# Diabetes self-management education interventions and self-management in low-resource settings; a mixed methods study

**DOI:** 10.1371/journal.pone.0286974

**Published:** 2023-07-14

**Authors:** Roberta Lamptey, Mary Amoakoh-Coleman, Babbel Djobalar, Diederick E. Grobbee, George Obeng Adjei, Kerstin Klipstein-Grobusch

**Affiliations:** 1 Polyclinic/ Family Medicine Department, Korle Bu Teaching Hospital, Accra, Ghana; 2 Department of Community Health, University of Ghana Medical School, Accra, Ghana; 3 Julius Global Health, Julius Center for Health Sciences and Primary Care, University Medical Center Utrecht, Utrecht University, Utrecht, The Netherlands; 4 Department of Epidemiology, Noguchi Memorial Institute for Medical Research, University of Ghana, Accra, Ghana; 5 Internal Medicine Department, Korle Bu Teaching Hospital, Accra, Ghana; 6 Centre for Tropical Clinical Pharmacology and Therapeutics, University of Ghana Medical School, Accra, Ghana; 7 Office of Research Innovation and Development, University of Ghana, Legon, Ghana; 8 Division of Epidemiology and Biostatistics, School of Public Health, Faculty of Health Sciences, University of the Witwatersrand, Johannesburg, South Africa; Monash University, AUSTRALIA

## Abstract

**Introduction:**

Diabetes is largely a self-managed disease; thus, care outcomes are closely linked to self-management behaviours. Structured self-management education (DSME) interventions are, however, largely unavailable in Africa.

**Aim:**

We sought to characterise DSME interventions in two urban low-resource primary settings; and to explore diabetes self-management knowledge and behaviours, of persons living with diabetes (PLD).

**Research design and methods:**

A convergent parallel mixed-methods study was conducted between January and February 2021 in Accra, Ghana. The sampling methods used for selecting participants were total enumeration, consecutive sampling, purposive and judgemental sampling. Multivariable regression models were used to study the association between diabetes self-management knowledge and behaviours. We employed inductive content analysis of informants’ experiences and context, to complement the quantitative findings.

**Results:**

In total, 425 PLD (70.1% (n = 298) females, mean age 58 years (SD 12), with a mean blood glucose of 9.4 mmol/l (SD 6.4)) participated in the quantitative study. Two managers, five professionals, two diabetes experts and 16 PLD participated in in-depth interviews. Finally, 24 PLD were involved in four focus group discussions. The median diabetes self-management knowledge score was 40% ((IQR 20–60). For every one unit increase in diabetes self-management knowledge, there were corresponding increases in the diet (5%;[95% CI: 2%-9%, *p<*0.05]), exercise (5%; [95% CI:2%-8%, *p*<0.05]) and glucose monitoring (4%;[95% CI:2%-5%, *p*<0.05]) domains of the diabetes self-care activities scale respectively. The DSME interventions studied, were unstructured and limited by resources. Financial constraints, conflicting messages, beliefs, and stigma were the themes underpinning self-management behaviour.

**Conclusions:**

The DSME interventions studied were under-resourced, and unstructured. Diabetes self-management knowledge though limited, was associated with self-management behaviour. DSME interventions in low resource settings should be culturally tailored and should incorporate sessions on mitigating financial constraints. Future studies should focus on creating structured DSME interventions suited to resource-constrained settings.

## 1. Introduction

Globally, 536 million people live with diabetes, and this number is projected to rise to 784 million by 2045 [1]. Eighty percent of these half a billion people live in low- and middle-income countries like Ghana [1]. Diabetes is a long-standing leading cause of morbidity and mortality [2] in Ghana, and among adults, the prevalence is 6.5% [3].

Diabetes self-management education (DSME), being a bedrock of optimal diabetes care can effectively improve glycaemic control and ameliorate the disease burden [4, 5]. DSME involves equipping patients, with knowledge for self-management. Several models for DSME interventions exist [4, 6]. Characteristics of DSME interventions include duration, cultural and linguistic tailoring, theoretical underpinnings, structure/ curriculum, mode of delivery, instructor characteristics, and intensity [6, 7]. Examples of theories that have been studied in relation to diabetes self-management include; the social cognitive theory and the empowerment theory [7, 8]. It is uncertain which of these characteristics of DSME interventions, account for effectiveness in improving glycaemic control and care outcomes [9]. Among a predominantly black population, Ryan et al reported an improvement in glycaemic control; specifically a difference in mean HbA1c, after a 6-month DSME intervention. They also found significant improvements in knowledge on complications and management of diabetes, glucose monitoring, and nutrition [10]. Similarly, a randomised control trial, that compared a culturally tailored DSME intervention in African-Americans, to usual care, reported significant reductions in HbA1c in the intervention arm at 6 months. However, at 12 and 18 months respectively, these differences were lost [11]. Contrastingly, a DSME intervention trial among African Americans, that emphasised the patient empowerment theory, reported significant improvements in self-care behaviours, quality of life and insulin use, even after 2 years [12]. Cunningham et al conducted a systematic review and meta-analysis of DSME intervention trials that were conducted exclusively African Americans. Contrary to the findings Ryan et al and Lynch et al, Cunningham et al reported a non-significant difference in mean HbA1c and no improvements in quality of life (QoL) between DSME intervention groups and usual care [6].

In Africa DSME interventions are not widely available; and studies on their effectiveness have likewise yielded conflicting results. An audit of interventions in S. Africa, found 27 DSME interventions. Five of these interventions offered structured education and the rest offered ad hoc education. Surprisingly, none of the interventions audited, had guidelines specifically dedicated to DSME [13]. Additionally, sustainability of facility-based structured DSME interventions is influenced by facility-, patient-, and provider level factors [14].

This limited availability of structured interventions in Africa, in particular, have consistently been reported in the literature [15, 16]. Likewise, the evidence on effectiveness of structured DSME interventions in Africa is sparse and inconclusive [15, 17]. Gathu et al conducted an RCT among 140 adults with diabetes attending a Family Medicine clinic in Kenya and reported no significant difference in mean A1c between groups. Gathu et al compared DSME delivered by certified diabetes educators to comprehensive care delivered by Family Physicians [18]. In contrast, an RCT comparing intensive structured DSME to conventional education in a facility in Nigeria showed a significant reduction in mean A1c at 6mo in the intervention arm [19]. To date, there are no structured DSME interventions in Ghana.

Structured DSME interventions for low-resource settings should be tailor-made for such settings. Such DSME interventions should take into consideration patient-, provider- and facility-level factors. Using a mixed methods design, we therefore sought to characterise DSME interventions in two urban low-resource primary settings, and to explore the (diabetes self-management) knowledge, and behaviours of persons living with diabetes (PLD).

## 2. Methods

### 2.1. Design

**Fig 1:**
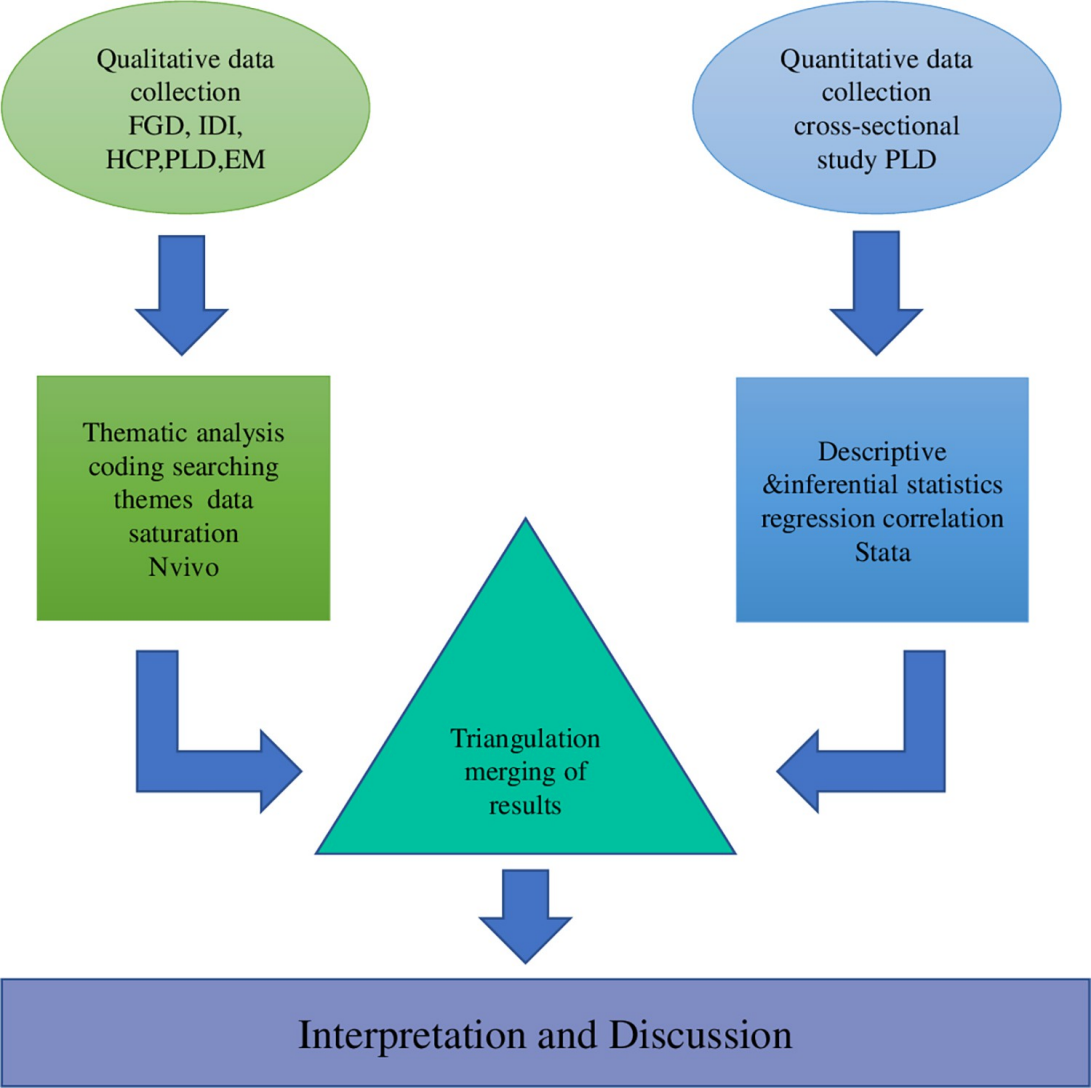
Convergent parallel mixed methods study design. Abbreviations: IDI- in-depth interview FGD-Focus Group Discussion HCP- healthcare professional EM-experts and managers.

A convergent parallel design [20] with triangulation was used. This design enabled collection of complementary data (quantitative and qualitative) concurrently (Fig 1). Thus, we merged the two research methods (quantitative and qualitative) to achieve our study aims. Data for the quantitative and qualitative studies were collected simultaneously; in parallel. Beyond data collection, the two methods converged at the point of analysing our results and interpretating our data. Specifically, we employed qualitative methods to deepen our understanding (of generalizable) outcomes from the quantitative study. In all the various aspects of this study, we placed equal emphasis on qualitative and quantitative data. Good Reporting of a Mixed-Methods Study (GRAMMS) [21] and Consolidated Criteria for Reporting Qualitative research (COREQ) [22] checklists were followed.

### 2.2. Setting

The study was conducted in, the Korle Bu Teaching Hospital polyclinic (KBTH) and Weija Gbawe Municipal hospital (WGMH), two public primary facilities located within the city of Accra, Ghana. KBTH is located in the Ablekumah South Metropolitan district and WGMH is in the Ga West Municipal district. We conducted one-on-one interviews and held focus group discussions with PLD in large open spaces at the study sites; Managers were also interviewed in-person on-site. Prescribed COVID-19 protocols were always observed. Experts were however, interviewed virtually.

### 2.3. Participant identification, study size and sampling

Participant recruitment and data collection occurred between January and February 2021. Using attendance records, a total enumeration of all eligible clients, seen at both study sites from December 2020 to January 2021, was done. These dates formed the frame and we included everyone within the frame, who met the eligibility criteria. The attendance records for each study site were used for retrieving relevant information on potential participants. Trained staff called all potential participants meeting eligibility criteria and invited them to participate. For each individual, three attempts were made to reach them. Interested participants received appointments for screening visits at the study sites, Subsequently, eligible participants underwent study procedures. Participants received reimbursement for travel costs and time. On average, each focus group discussion (FGD) lasted about an hour.

We assumed a 50% prevalence of diabetes self-management knowledge and 10% non-response rate [23, 24]. The level of significance was set at 5%. A sample size of 425 PLD was therefore required for the cross-sectional study. Recruitment for in-depth interviews (IDI) continued until saturation was reached and no new themes emerged.

For the qualitative study, PLD were identified through convenient sampling and snowballing. Managers and healthcare professionals (HCPs) were purposively sampled, but judgemental sampling was used for selecting experts.

#### 2.3.1 Eligibility criteria for PLD, HCP, managers, and experts

Participants had to meet all the following eligibility criteria and none of the exclusion criteria to be included. Experts were nationally recognised diabetologists. HCP and PLD were staff of the study sites and clinic attendants respectively. Managers were the facility heads. PLD were 18 years or older and ambulant at the time of recruitment. People known to have type 1 diabetes, or cognitive or psychiatric impairment were excluded.

### 2.4. Instrument development

We anticipated heterogeneity in the responses because of the case-mix variation and developed semi-structured interview guides to guide all interviews. RL and MAC, who both understood the local culture and norms, developed, and refined these interview guides. The interview questions were informed by the results of a literature review of DSME in low-resource settings, conducted by RL. Participant information guides on the purpose and methods of the study and anonymity was developed by RL and reviewed by MAC and KKG.

### 2.5. Data collection

This study was conducted in line with the principles of the Declaration of Helsinki [25]. Prior to any study procedures, each participant provided written informed consent. Participants who consented to taking part in FGDs, also signed non-disclosure statements. These statements were an assurance that information divulged by participants during the FGD would remain within the group and not shared outside the group. Since the sessions were audio taped and transcribed, participants were assigned pseudo-names. During the FGDs participants were referred to using these pseudo-names to maintain their confidentiality. Access to each facility was granted by the respective heads.

#### 2.5.1 Quantitative data collection

Diabetes self-management knowledge of PLD, the primary outcome variable, was measured on the spoken knowledge in low literacy persons with diabetes scale (SKILLD) [26]. SKILLD is a 10-item questionnaire with each option giving a score of either 0(0%) or 10(100%). Higher scores indicate better diabetes self-management knowledge.

The variables which were modelled as explanatory variables were anthropometric measures, sitting blood pressure, duration of diabetes, insulin use, random blood glucose, sex, family history of diabetes, income, educational level, occupation and the summary of diabetes self-care activities scores (SDSCA) [27].

*2*.*5*.*1*.*1 Measurement procedures*. We scrupulously followed standard recommended procedures for all measurements [28–30]. We used StatStrip Xpress glucometers (OneTouch, Taiwan) for measuring random blood glucose [29], and Omron M7 sphygmomanometers (Omron, Japan) for measuring sitting blood pressure [28]. An Omron digital scale, a stadiometer, and inelastic tape measures were used to take anthropometric measurements [30].

Duration of diabetes, insulin use, sex, family history of diabetes, income, educational level, and occupation were captured with a general questionnaire. The SKILLD and SDSCA instruments were interviewer administered.

#### 2.5.2 Qualitative data collection

**Fig 2:**
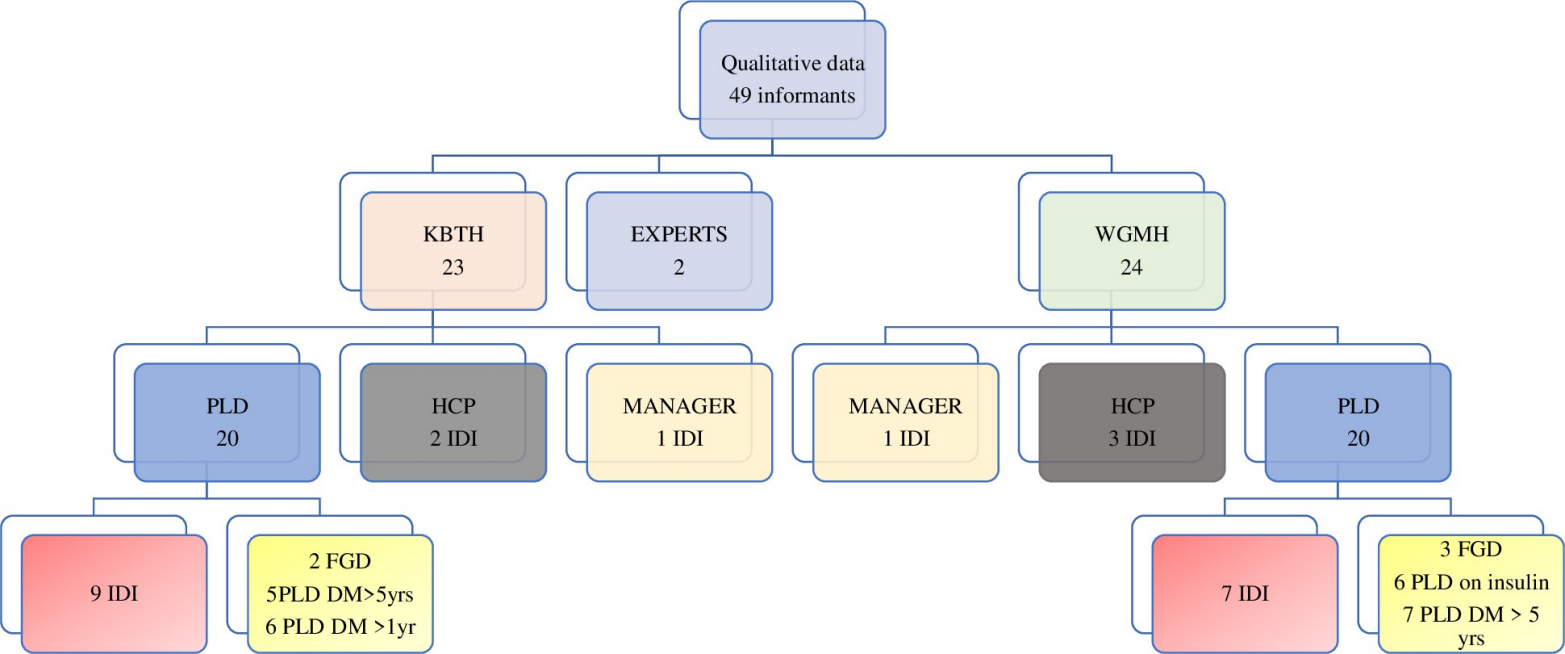
Qualitative data collection procedures and number of informants. Abbreviations: KBTH-Korle Bu Teaching Hospital; WGMH-Weija Gbawe Municipal Hospital IDI- in-depth interview FGD-Focus Group Discussion DM-duration of diabetes < less than > greater than yrs- years HCP-Health care professional PLD- person living with diabetes.

Fig 2 depicts the informants and qualitative procedures undertaken. RL and BB either conducted or coordinated the IDI and FGD. Interviews were conducted in in English, Twi, or Ga. Responses were audio-recorded digitally and handwritten field notes were taken. Some of the PLDs recruited from the KBTH study site might have known RL as a staff of that facility. All other PLD involved in the study did not have any prior relationship with the data collectors. Experts and Health Care Professionals were colleagues of RL. The roles of the researchers were to facilitate the FGDs and conduct the interviews.

### 2.6. Data management and analysis

#### 2.6.1 Quantitative analysis

Total SKILLD score (knowledge) was analysed both as a continuous and categorical variable. The individual SKILLD items were dichotomised into correct and incorrect responses and summarised using counts (percentage).

To test the strength of the association between the total SKILLD score and SDSCA sub-domains, the Pearson’s correlation coefficient was calculated. The appropriate regression tests involving ordinary least squares regression or quantiles regression were performed to assess the association between total SKILLD score) and clinically relevant variables. All analyses were conducted with Stata v16.1. We performed two-tailed hypotheses tests; statistical significance was set at 0.05. REDCap data management system was used for data capture.

#### 2.6.2 Qualitative analysis

Data was manually analysed independently, by RL, BB, and a research assistant, using an inductive thematic approach. Audio-recordings were transcribed verbatim. Transcription, initial coding, and thematic analysis were done concurrently with data collection. We extracted both latent and manifest content. Transcripts were line searched for recurring words and phrases. Concepts were then used to generate initial codes and further expanded by applying these initial codes to additional transcripts (open coding). Sub-themes were identified by reviewing the data for repeating patterns in participant’s responses. Sub-themes were merged into themes, ensuring themes closely described original content of transcripts. Emerging themes were categorized and compared across the various (informants) groups using colour coded comparative charts. Direct quotes were extracted. Our informants were fully engaged in all phases of our study. We selected participants who could best provide answers to our research question. Data saturation was reached when no new themes emerged. Subsequently, RL used NVivo (released March 2020) to organise the data.

MAC reviewed the themes against the final organisation of the data to ensure that there was agreement in the data collected and its final presentation. Discrepancies and suggestions for review were resolved through dialogue.

*2*.*6*.*2*.*1 Rigour*. Data, informant, and investigator triangulation was used to ensure rigor and comprehension of concepts. The transcripts and subsequently thematic analysis were shared with informants to check for accuracy and to provide feedback. Team meetings with co-investigators experienced in qualitative methods enhance the credibility of our data. The study procedures have been described to allow replicability. The use of NVivo improves transparency and reliability of the coding. Concurrent collection of quantitative and qualitative data improve internal validity.

### 2.7. Ethical approval

Ethical approval was granted by the Institutional Review Board of KBTH (STC/IRB/000175/2020) and the Ethics Review Committee of the Ghana Health Service (GHS-ERC 05/10/20). After ethical clearance had been obtained, the head of each facility granted permission for the study.

## 3. Results

The quantitative results are summarised in tables and the qualitative results are presented by themes. All the quantitative results are presented first followed by the qualitative results.

### 3.1. Quantitative results

#### 3.1.1 Participant’s flow and baseline characteristics

In total, 1202 participants out of 1735 potentially eligible clients were not included: 54 participants had travelled (zero from WGMH), 1029 were unreachable by telephone (544 from WGMH), 95 declined (one from WGMH), 25 were dead (one from WGMM). As 112 out of 533 eligible participants invited failed to report, four additional participants (0 from WGMH) were consecutively sampled. Finally, 425 participants were included in the analysis.

Participants’ baseline socio-demographic and clinical characteristics are shown in Table 1. Additionally, the mean body weight was 98kg (SD 16). The mean waist circumference for males was 94 cm (SD 16) and for females it was 98 cm (SD 16). The mean systolic and diastolic blood pressure were 133 mmHg (SD 21) and 81 mmHg (SD 12) respectively. The mean random blood glucose was 9.4 mmol/l (SD 6.4) mmol/l.

**Table 1:** Descriptive (socio-demographic and clinical) characteristics of participants.

Variable	Frequency	Percentage
Age(N = 425)		
≤39	26	6
40–49	77	18
50–59	132	31
60–69	120	28
70+	70	17
Mean (SD)	581(SD 12)	
Sex (N = 425)		
Female	298	70
Male	127	30
Educational level (N = 425)		
None	52	12
Primary and middle	194	46
Secondary and vocational	118	27
Tertiary	58	14
Other	3	0.7
Marital Status (N = 425)		
Married	245	58
Never married	24	5.7
Living together	1	0.2
Widowed	96	23
Divorced	59	14
Occupation (N = 425)		
Professionals with university degrees	36	8.5
Professionals without university degree	30	7
Clerks, motor vehicle drivers, mechanic	89	21
Cooks, barbers, domestic staff, gas staff	36	8.5
Labourers and petty traders	86	20
Apprentices, educated youth, unemployed	148	35
Ethnicity (N = 425)		
Akan	206	49
Ga/Adangbe	124	29
Ewe	53	13
Other	40	9.5
Religion (N = 425)		
Christian	380	89
Islam	42	9.9
Other	3	0.7
Size of your household (N = 412)		
1–2	91	22.09
3–4	136	33
5–6	116	28
6+	69	17
Min-Max	1–27	
Mean (SD)	5(3)	
Additional sources of income (N = 417)		
No	342	82
Yes	75	18
Years of diabetes illness (N = 416)		
≤1	48	12
2–3	95	23
4–9	138	33
10+	135	33
Min-Max	<1–45	
Mean (SD)	7.7 (0.3)	
Family history of diabetes (N = 418)		
No	179	43
Yes	239	57
Have any device for checking blood sugar at home (N = 418)		
No	252	60
Yes	166	40

Abbreviations; SD = Standard Deviation N = number of observations

#### 3.1.2 Diabetes self-management knowledge among PLD

The median SKILLD score was 40%(IQR 20–60). The results of the individual SKILLD items revealed significant deficits in diabetes self-management knowledge. Only 13 (3%) participants knew the normal HbA1c range and 162 (39%) knew the normal fasting glucose range. In total, 208 (50%) and 196 (40%) knew the signs of hyperglycaemia and hypoglycaemia, respectively. Only 227 (54%) knew how to treat hypoglycaemia. The importance of foot care was known by 135 (32%) and only126 (30%) participants knew the recommended frequency for foot examinations. The frequency of eye examinations and exercise was known by 176 (42%) and 199 (48%) respectively. Finally, 247 (59%) participants knew the long-term complications of diabetes.

#### 3.1.3 Factors associated with diabetes self-management knowledge

There was no association between SKILLD score and any of the baseline socio-demographic and clinical variables.

#### 3.1.4 Association between diabetes self-management knowledge and self-management behaviour

Pairwise corelations showed that SKILLD score was positively correlated with behaviour (SDSCA). The correlation coefficient was 0.22 (p<0.01) for diet, 0.19 (p<0.01) for medication, 0.14 for exercise (p<0.05), 0.39 (p<0.01) for glucose testing and 0.38 (p<0.01) for foot care.

#### 3.1.5 Influence of diabetes-self-management knowledge (SKILLD) on Diabetes Self-Care Activities Measure (SDSCA) sub-domains

The effect of total SKILLD on self-management behaviours (SDSCA sub-domains), adjusted for age, education, diabetes duration, family history of diabetes and ownership of a glucometer is displayed in Table 2.

**Table 2:** Influence of knowledge (spoken language in low literacy in diabetes scale) on diabetes self-care activities measure sub-domains.

Variable	Diabetes Self-Care Activities Measures				
	OLS		Quantile regression		
	Diet	Medication	Exercise	Blood testing	Foot
	aβ[95%CI]	aβ[95%CI]	aβ[95%CI]	aβ[95%CI]	aβ[95%CI]
SKILLED Knowledge	0.05[0.02–0.09]**	0.01[0.002–0.02]*	0.05[0.02–0.08]**	0.04[0.02–0.05]***	0.02[-0.02–0.05]
Age group					
≤39					
40–49	1.55[-2.39–5.48]	-0.73[-1.99–0.53]	1.00[-1.27–3.27]	1.07[-0.83–2.97]	0.33[-2.55–3.22]
50–59	1.21[-2.57–4.99]	0.37[-0.77–1.52]	2.00[-0.49–4.49]	0.93[-0.96–2.82]	0.17[-2.47–2.81]
60–69	1.03[-2.75–4.82]	0.20[-0.96–1.35]	1.00[-1.09–3.09]	1.07[-0.83–2.97]	0.33[-2.27–2.93]
70+	1.62[-2.46–5.70]	-0.03[-1.25–1.19]	0.50[-1.60–2.61]	2.07[-0.88–3.02]	0.33[-2.36–3.03]
Educational level					
None					
Primary	2.06[-0.93–5.05]	-0.96[-1.69- -0.24]**	1.22[-1.98–5.98]	-0.28[-0.94–0.37]	-0.17[-1.57–1.24]
Middle	1.77[-0.90–4.45]	-1.02[-1.59–0.45]***	-0.50[-2.33–1.33]	-0.50[-1.05–0.05]	-0.17[-1.46–1.13]
Secondary	3.19[0.33–6.04]*	-1.39[—2.07- -0.71]***	2.50[-0.20–5.20]	0.07[-1.32–1.46]	0.17[-1.48–1.81]
Vocational	2.97[-2.19–4.83]	-1.28[-2.02- -0.36]**	-2.22[-3.32–6.32]	0.78[-2.57–4.14]	0.17[-2.97–3.31]
Tertiary	1.21[-2.19–4.62]	-1.24[-1.98- -0.50]***	0.50[-2.16–3.16]	2.86[0.81–4.90]	1.00[-1.36–3.36]
Other	-2.54[-11.8–6.75]	0.08[-0.76–0.92]	10.0[-17.2–37.2]	7.07[2.85–11.3]***	7.00[1.98–12.0]**
Years of diabetes illness					
≤1					
2–3	0.38[-2.45–3.21]	0.99[-0.01–1.99]	2.66[-2.18–3.18]	-0.34[-1.01–0.29]	-0.17[-1.48–1.15]
4–9	0.34[-2.41–3.09]	0.93[-0.01–1.89]	3.11[-2.45–6.45]	-0.50[-1.15–0.15]	-0.33[-1.81–1.14]
10+	0.85[-1.98–3.68]	1.25[0.30–2.21]	0.50[-1.85–2.85]	-0.35[-1.19–0.48]	-0.17[-1.83–1.50]
Family history of diabetes					
No					
Yes	-1.06[-2.69–0.57]	0.13[-0.31–0.58]	-0.50[-1.92–0.92]	0.00[-0.48–0.49]	-5.55[-1.00–5.99]
Device for checking blood sugar					
No					
Yes	2.34[0.60–4.08]**	0.61[0.20–1.03]**	-1.00[-2.38–0.39]	1.00[0.32–1.67]**	0.17[-0.94–1.27]

NOTE: Abbreviation: SKILLED = Spoken Language in Low Literacy in Diabetes; OLS-ordinary least squares regression; aβ = adjusted Coefficient estimate. Covariates used age, education, duration of diabetes and family history. *P*-value Notation

*** p<0.01

** *p*<0.05

* *p*<0.1 type of test multiple linear regression

### 3.2. Qualitative results

#### 3.2.1 Participants

Fig 2 depicts the types of informants and data gathering techniques used.

#### 3.2.2 Emerging themes

**Fig 3:**
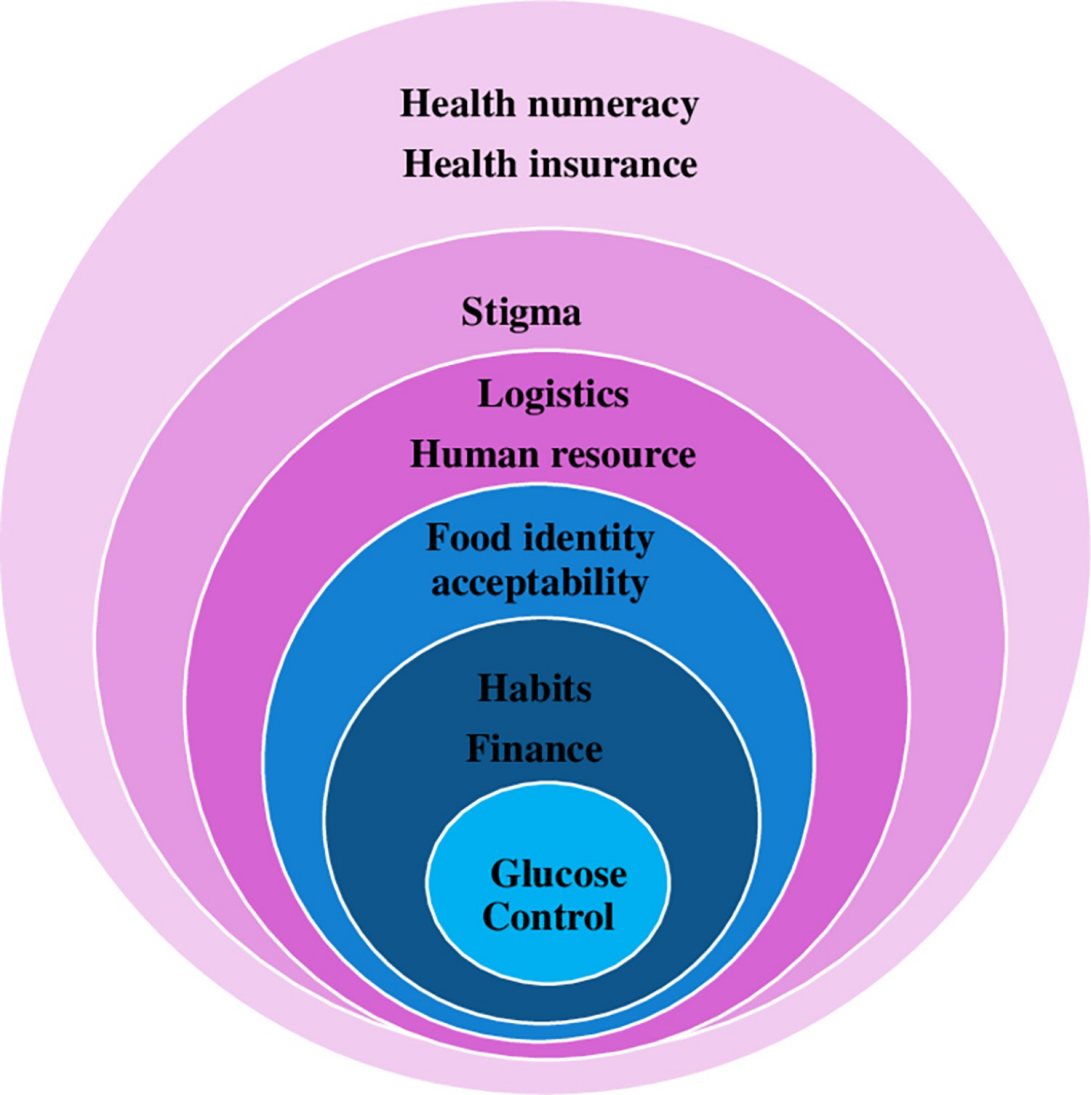
Thematic areas DSME needs in resource constrained settings.

The themes identified are displayed in Fig 3 and include health numeracy and financing, logistics and norms.

*3*.*2*.*2*.*1 DSME interventions*. We found that PLD received DSME from nurses, doctors, and or nutritionists. The education was un-structured, didactic, group-based and delivered in-person prior to consultations. Groups typically had about 20 PLD per group and sessions lasted for about 30 minutes, on average.

We observed that varied perceptions among informants, resulted in contrasting perspectives on existing DSME interventions. For example, PLD generally favoured group over individualised education, placing value on peer-to-peer learning. The consensus among PLD seemed to be that individualised education provided prior to a consultation was inadequate. They pointed out that, the group sessions inadvertently provided avenues for newly diagnosed persons to draw on the experience and diabetes self-management knowledge of their peers. All patient groups interviewed, recommended that peers, together with health workers should be used as diabetes educators.

PLD described existing DSME interventions as beneficial but reported that teaching aids were not culturally or linguistically adapted.

R5 FGD KBTH–“*often the books available on diabetes have examples of foods eaten abroad”*

R4 FGD WGMH*-“…*.*we have been given a book that teaches us how to manage diabetes*. *The book is normally read to me…*. *“*

R5 FGD WGMH: *“…*..*about the pamphlet*. *It sometimes contains foreign information which is their food and what they need to do in order to take care of themselves so I think they should be limited to our local activities”*.

R3 FGD WGMH: *“……*..*I prefer all the teachings in a leaflet form…*. *Those who can’t read the leaflet personally*, *can allow their children or friends to help them read”*

In contrast to PLD, providers and diabetes experts, thought that existing DSME interventions were at best parsimonious. Human resource constraints, lack of logistics, unavailability of academic courses, and lack of a policy direction were challenges identified. Except for the doctors, none of the other participant groups were familiar with structured DSME.

The unstructured nature of existing DSME interventions meant PLD continued with self-management education classes ad-infinitum. Our informants appreciated the knowledge reinforcement.

R IDI KBTH: “…[They] *are doing their best because the doctors really educate the patients on how they can manage the diabetes themselves*.*”*

PLD used DSME interchangeably with health education. They recommended that churches and other community spaces and mass media communication channels be used for DSME.

Most informants preferred the existing in-person format to virtual sessions.

*3*.*2*.*2*.*2 Diabetes self-management knowledge*. Knowledge on self-management was deficient and self-care practices among PLD were inadequate.

R4 FGD KBTH: “*I used to inject the insulin in the house but anytime I inject it*, *my sugar level rises*, *so a doctor friend of mine advised me that*, *the insulin should be injected in the hospital and by a doctor so for 5years now I have stop using the insulin*.”

PLDs echoed several myths as truths. Notwithstanding, PLD bemoaned the inconsistencies in nutritional recommendations.

*3*.*2*.*2*.*3 Self-management behaviours*. PLD knew more about the importance of medication use, self-blood glucose testing, meal planning, exercise, and routine reviews than about foot care. None of the PLD and HCPs mentioned foot care. Contrastingly, the experts mentioned foot care, routine investigations, and eye screening by as important components of self-management.

Several barriers to self-care, even when diabetes self-management knowledge was apparently adequate, were enumerated by all informants.

*3*.*2*.*2*.*4 Finance*. Among persons with low health numeracy in resource constrained settings, there’s very little choice in lifestyle-related matters. Poverty was the common pathway for restricted access to information, food, healthcare, and medication. PLD described dependence on literate relatives to access information contained in patient education leaflets.

PLDs and HCPs enumerated the cost constraints faced by PLD and how these influenced food consumption patterns. HCPs were empathetic, yet seemingly frustrated by the vicious cycle of high carbohydrate consumption and hyperglycaemia among PLD. PLD and HCPs both indicated that consumption of fresh produce was dependent on seasonality.

PLD described frequent stockout of medications covered by insurance. None of the PLD groups complained about costs associated with home glucose testing. The experts, however, noted that patient’s inability to afford home glucose monitoring, was a barrier to optimising glycaemic control.

*3*.*2*.*2*.*5 Norms and belief systems*. Finances were not the only determinants of meal patterns. PLD voiced the conflict between their intentions and actions. They recounted the difficulty of executing planned behaviour (such as portion control). They described nutritional recommendations as a deviation from cultural norms. PLD described wanting to ‘belong’ at social gatherings. HCP and PLD alike alluded to the fact that diabetes (especially among young persons) was stigmatised.

PLD said they receive conflicting messages from traditional herbal and alternative medicine practitioners, religious leaders, and HCPs. Furthermore, they expressed a belief in destiny and the existence of an external locus of control. These belief systems contributed to poor self-care.

## 4. Discussion

We sought to characterize DSME interventions and to explore the self-management knowledge and behaviours of persons living with diabetes. The interventions studied were unstructured, group-based and delivered in-person, mostly by nurses. Self-management knowledge and behaviours were sub-optimal. Self-management behaviours were influenced by financial constraints, culture, beliefs, stigma, and conflicting messaging.

### 4.1. Existing DSME interventions

The unstructured nature of the DSME interventions and use of group delivery methods, probably reflects an attempt to increase the accessibility of DSME, given the resource constraints. Building sustainability into DSME interventions for resource constrained settings, is key. The use of “non-internet” mass media to disseminate DSME interventions, as proposed by our informants, might be a sustainable option. Moreover, since most of our informants found repetition of content useful, mass media channels may be well patronised. Similar to our findings, the importance of the traditional media in disseminating DSME, was identified in another African study [31]. However, people living with long-standing diabetes in Iran reported that repetition of DSME content was not useful. A direct contrast to the views of the informants in our study. Importantly, the population studied in Iran had significantly higher literacy levels relative to our study population and this difference may account for the divergent views [32]. In Iran, health literacy has been shown to be positively correlated with health behaviours [33].

### 4.2. Diabetes self-management knowledge and it’s relation with self-management behaviours

Our findings of limited diabetes self-management knowledge, echo those of previous studies [34, 35]. The extremely low SKILLD scores, from our quantitative study, reflect the depth of lack of knowledge on self-care. The themes we identified in this study, provide some explanations for, and elaborate on the inadequate diabetes self-management knowledge among PLD. In particular, the low literacy levels and inconsistent messaging are plausible explanations for the low SKILLD scores.

Despite the seemingly insurmountable barriers to self-care expressed by PLD, our results show that, diabetes self-management knowledge is positively associated with several self-management behaviours. In congruence with our findings, a multi-centre cross-sectional study in Ghana found diabetes self-management knowledge to be a predictor of self-care: every 1 unit increase in knowledge was associated with 20 times the odds of higher SDSCA scores [36]. Although, the proportion of people with tertiary education was comparable to our study, the proportion of people with no education, was 50% higher, relative to our study population [36]. Efforts at improving self-management knowledge might therefore ultimately also translate into better self-care behaviours among PLD in low-resource settings.

Our findings suggest that formal education is not associated with self-management behaviours except for adherence to medication. In contrast, Rothman et al found that having tertiary education was associated with a 12% increase in SDSCA scores, indicating better self-care behaviours [26]. Surprisingly, a cross-sectional multi-centre study from Ethiopia, observed, that not having formal education was associated with increased odds of having good self-care behaviours (AOR = 2.6, 95% CI = 1.32–5.25) [37]. This estimate of the effect of formal education on self-management behaviour, could have been biased by the absence of a control group.

### 4.3. Diabetes self-management behaviours

Our findings of low scores across all domains of the SDSCA, parallel findings from a multi-centre study in the Northern region of Ghana [35]. The socio-demographic and clinical profiles of the participants in these two studies were similar except for diabetes duration. The duration of diabetes was longer in the study by Mogre et al. [35], however, despite having had diabetes for longer, the self-management behaviours were just as sub-optimal as in our study. The low SDSCA scores from the quantitative study and the qualitative results from the IDI and FGDs both indicate poor self-management among PLD. It is plausible that, poor self-care behaviours are fuelled by both factors within and beyond the individual’s control; particularly the financial constraints mentioned earlier. A cross-sectional study involving PLD in a specialist clinic of a tertiary teaching hospital in Nigeria, also echo our findings of low scores on all domains of SDSCA [38].

The alarmingly low knowledge scores on foot care, and correspondingly poor practice of foot care, in our study is disturbing. Our findings provide strong justification for emphasising foot care in DSME interventions. Curricula which emphasise the relation between amputations, glycaemic control, routines, and daily lifestyle choices would be beneficial. The qualitative results from our study provide further insight into the low scores in the domain of foot care and parallel findings from other sub-Saharan African countries [39] and other regions of Ghana [35]. Our findings also resonate with a qualitative facility-based study among a predominantly agricultural community [34]. Bossman et al reported deficits in diabetes self-management knowledge and self-care behaviours in the domains of nutrition, exercise, and foot care with foot care being the least known and practiced [34]. It is thus not surprising that, amputations are major causes of morbidity among PLD in Ghana and other sub-Saharan African countries [40].

Our findings indicate a high demand for diabetes self-management information, especially, culturally tailored information on nutrition therapy, albeit poor adherence to nutritional recommendations. Unfortunately, the edicts of self-care behaviours, particularly in the domain of nutrition; deviate from local cultural norms. This divergence of norms and recommendations could contribute to the poor adherence. Furthermore, Unavailability of formal training in DSME for providers, could contribute to inconsistent messaging on nutritional therapy. Our findings parallel those from, a study conducted in specialist clinic in Nigeria, that reported confusion about nutritional recommendations, and the unacceptability of nutritional recommendations [41].

We found that behaviour change seemed to be a hurdle that persisted, despite adequate diabetes self-management knowledge. Our results suggest that our informants’ capacity to modify established behaviours might be limited. Previous behaviour is a known predictor of adherence to self-care recommendations [42]. Incorporating education on behaviour change strategies, may therefore, be a useful addition to the existing DSME interventions.

### 4.4. Financial constraints

In this study, financial constraints transcend multiple aspects of diabetes self-management: adherence to self-management recommendations, keeping clinic appointments and purchasing medications. In particular, medications which were unavailable on the National Health Insurance were largely inaccessible. Likewise, for many of our informants, accessibility of vegetables was determined by their seasonality. Our findings collaborate previous findings from Ghana [43], and Benin [44]. de-Graft Aikins et al have previously shown that, cost is a major and important limiting factor in several domains of self-management [43].

### 4.5. Norms and belief systems

Some of our informants expressed the belief that the locus of control resides outside the individual. As reported widely in previous studies from Ghana [41, 43], Benin [44], Malawi and Mozambique [31], we also found a belief in “divinity” that influenced perceptions of diabetes and diseases in general. Potentially, these local belief systems could adversely affect attitudes to self-care and self-care behaviours. This suggests a need to include sessions on the locus of control, when designing DSME interventions for such settings.

### 4.6. Stigma

Hospital based DSME was more valued than community-based DSME, because of diabetes-related stigma. Our finding that diabetes is stigmatised, suggests that, having support persons as part of DSME interventions might be beneficial. Using peer educators may offer net-working opportunities for PLD and discussing disclosure may improve effectiveness of DSME interventions. The finding of stigma and lack of family support was also reported by Mogre et al. [45] Among Ghanaians, family non-support has been found to be negatively correlated with diabetes self-management behaviours [46]. Family support has a linear relation with self-care [47].

### 4.7. Strengths and limitations

Quantitative analysis enabled us to generate valid unbiased estimates of diabetes self-management knowledge, and behaviours. The mixed methods design provided additional qualitative data, and insights into the results of the quantitative study. The data was coded and analysed by researchers well accustomed to the Ghanaian culture. Data was generated from a variety of informants and study participants, managers, PLD, HCPs and experts.

The generalisability of the study to the Ghanaian population, however, is limited because the study was conducted only in two facilities within the Greater Accra region. However, the clientele of KBTH come from all over Ghana. Our findings may also not be generalisable to people known to have type 1 diabetes. Furthermore, the use of consecutive sampling may limit the representativeness of our sample.

## 5. Conclusion

The DSME interventions studied were under-resourced and were not structured. Our findings indicate very limited diabetes self-management knowledge and poor adherence to self-care recommendations. Barriers to self-care included cost constraints, cultural norms, stigma, and belief systems. DSME interventions should incorporate sessions on mitigating these barriers. DSME should be culturally tailored and linguistically modified for people with low literacy. This may improve self-management, ultimately reducing the difficulties of PLD in resource constrained settings. Future mixed-methods cohort studies should focus on elucidating factors associated with effectives of DSME interventions in low resource settings.

## Supporting information

S1 Checklist COREQ (COnsolidated criteria for REporting Qualitative research) checklist. (PDF)

S2 Checklist STROBE statement—Checklist of items that should be included in reports of *cross-sectional studies*. (DOCX)

S1 File Transcripts. (DOCX)

S2 File ZOOM in-depth interview with an expert-DR 1. (DOCX)

S3 File Discussion with prayer/policy makers. (DOCX)

S4 File Transcription from facility xxx. (DOCX)

S5 File Transcription from facility YYY. (DOCX)

S6 File Transcription on diabetes self-management education at facility xxx. (DOCX)

S7 File (DOCX)

S8 File (DOCX)

S9 File Facility yyy. (DOCX)

S10 File Transcription on diabetes self-management education at facility yyy. (DOCX)

S11 File (DOCX)

S12 File (DOCX)

S13 File IDI facility yyy 1. (DOCX)

S14 File Transcription on diabetes self-management education at facility xxx on x^st^ Feb 1957. (DOCX)

S15 File IDI patient facility xxx. (DOCX)

S1 Data (XLSX)

S2 Data (NVPX)
