# Supplementary material for: Diabetes self-management education interventions and self-management in low-resource settings; a mixed methods study
Source: PLoS One. 2023 Jul 14;18(7):e0286974. doi: 10.1371/journal.pone.0286974 (PMC10348576; doi:10.1371/journal.pone.0286974)
Supplement: S1 File — (DOCX) [file pone.0286974.s003.docx]

**Transcription at hospital A**

**I: Welcome.**

R: Thank You.

**I: How many years have you being living with diabetes?**

R: It started from last year 2020.

**I: Do you take the medicine orally or through injection.**

R: When I came to the hospital, they injected me with some of the drug and gave me some to drink.

**I: Do you now drink the medicine.**

R: Yes.

**I: Don’t you inject some of them.**

R: No please.

**I: What should patients living with diabetes do in order to take care of themselves?**

R: When you are diagnosed of the disease, you have to take the prescribed drugs to prevent complication. They should also be mindful about the choice of meals they take.

**I: What are the choices of food diabetic patients should consider eating and the ones they should desist from eating.**

R: We should not eat starchy food because it causes complication; examples of such food are yam, cassava, both sugar and butter bread. On the other hand food such as oats, cocoyam leafs, cabbage and plantain is very good for a diabetic patient**.**

**I: Who do you think should handle the delivery of education for a diabetic patient? Do you think it should be the doctor or someone living with diabetes?**

R: I think those who are living with the disease can educate those who have not being diagnosed of the disease. We can teach them the choice of meals and amount of sugar intake. I know a man who eats bread with sugary solution and I always advise him to refrain from it because it is a bad practice and can cause diabetes.

**I: Can doctors also involve themselves in the education.**

R: Yes, they can, by grouping us when we come to the hospital in order to educate us.

**I: Do you prefer the groupings or a one on one education.**

R: I prefer them grouping us because we all learn from each other on how to manage ourselves.

**I: Where do you prefer the education to be organized? Do you think it should be held in the hospital or different place?**

R: I think the church premises would be a good place to have the education because in my church for instance before the Christmas celebration, some doctors in our church educated us on how to eat well and take good care of ourselves and it really helped us a lot.

**I: In delivering the education do you think it should be delivered one day, on regular basis, every two weeks or every month. How do think it should be delivered.**

R: I think it should be organized monthly.

**I: Do you think the education should be delivered face to face or virtual (radio or internet)**

R: Some people may not get the chance to listen on the radio so I think us we come here and they see us face to face they can deliver the education to us.

**I: In hospital A do you think the education giving to you is beneficial.**

R: Yes it is.

**I: What are some of the things that prevent patients from obeying doctors’ advice despite the education being delivered to them?**

R: Some of them don’t have time for themselves to be doing what the doctor advices them to do.

**I: Would you say financial constraints is also part of the reasons patients don’t go according to what the doctor says.**

R: Yes it also a reason because one will not be able to purchase the right amount of food he or she needs in order to be healthy.

**I: What do you think would be important to be discussed during the education session?**

R: I think it should include ways by which the government can assist us financially because the treatment is very expensive. I usually go to sell when I was not diagnosed of the disease but now I can’t do that because of the situation so we need assistant from them.

**I: Thank you very much for your time.**
