# Supplementary material for: Diabetes self-management education interventions and self-management in low-resource settings; a mixed methods study
Source: PLoS One. 2023 Jul 14;18(7):e0286974. doi: 10.1371/journal.pone.0286974 (PMC10348576; doi:10.1371/journal.pone.0286974)
Supplement: S2 File — (DOCX) [file pone.0286974.s004.docx]

**ZOOM In-depth interview with an expert-DR 1**

**I: I want to first assure confidentiality that the meeting happening here will not go out. It’s solely for research. No names will be mentioned and everything will remain among the team and both of us. This is an in-depth interview with an expert on diabetes concerning diabetes self-management.**

**I: Please I will like to start by asking, what you know about diabetes self-management education.**

R: Diabetes self-management education is a way of ensuring that patients have enough knowledge, support and skills, and the necessary ability to manage their diabetes on their own. And in a way to alter behaviour so that the outcomes are encouraged.

**I: What is the minimum content that the DSME should have, if it’s to take place?**

R: You mean if you wanted to create one for Ghana?

**I: Yes please.**

R: I think it’s important that patients understand what diabetes and their treatment options are. Patients must be thought things like medical nutrition, therapy, how they can incorporate physical activities into their lifestyle. They should understand their medicine and how it works and their side effects. They should understand the need to monitor and also understand what I call survival skills such as how to manage emergencies like hypos and hypers. They must have some sort of target as part of their program and of course, they must know about misconceptions, gestational diabetes among others. Anything that ensure that patients have good outcomes for diabetes care is important and it must be culturally sensitive and individualised because no two individuals are the same.

**I: You talked about gestational diabetes, is there any other field in medicine or in practice apart from obstetric that could be involved in DSME?**

R: I guess if I get your question right, patients who have kidney problems also need to have some amount of glycerine , people with eye problems must know the kind of exercises they must do because that can predispose them. It should be individualised. Depending on the patient in front of you, you design a program that suits them. You can have the group structured education then after, give them individualised session if you can.

**I: In your expert opinion, how do you think the DSME should be delivered? Should it be in a structured way with a formal curriculum, or it should be ad hoc? And why?**

R: Certainly a structured education is better and there is a lot of evidence for that. Ad hoc certainly is not a good idea. When it is a structured curriculum, then you are assured that the patient will have an extensive and complete educational program which will benefit them. So definitely I prefer a structured educational program to ad hoc program. The two can never be comparable.

**I: What should the contact time be for those who undergo this program? How many session will be needed? And how long should each session last?**

R: Contact time should also be individualised. Some people can sit for longer hours others cannot. But if you look at the topics and the typical structured educational program then there has to several sessions. I doubt if you can deliver everything in one session. You have one session for diabetes another for lifestyle changes and another for medical nutrition. There should be several sessions but I don’t think I can give you the exact duration of each session. What I do know is that if you don’t have people who are qualified to deliver the education, people lose interest and so you must have people who can engage your patients so that even if it’s a long session, they keep interested in what is going on. Otherwise they may tune out and it will be a waste of everybody’s time especially in group settings. You should access the individual you are dealing with. If it a child, of course the duration may be shorter. With an adult or adolescent it may be longer. With adolescents apps and gadget may be employed, that is if they are literate. You tailor the education to each individual in a way that you think will be good for them. I don’t think there should be a specific duration for all, but you must have a way of accessing if your information flow is being understood.

**I: You talked about how the topics should be individualised and also mentioned group therapy. Which of these do you think will be better for DSME?**

R: Personally, I think a mixture of the two will be good. First you have the group session and teach them about the basics, what diabetes entails. But when it comes to what you want them to do I think individualised sessions will be better. So you evaluate the patient, set target for that particular patient, find out if the patient can monitor himself and the survival skills of the patient. But things like the description of the disease process and treatment options and complications of diabetes I think can be done in group sessions. Years ago one pharmaceutical company gave us the opportunity to sit patients down and teach them, about six or seven patients. At the end of the program, one patient came to me and was very excited about the fact that he learnt so much. Later, two other patients came around and thought it was a waste of their time. Everybody is different so I think you should really assess a patient and fine out what will be beneficial. By and large, a little bit of group and more of individual sessions will be preferred.

**I: With these suggestions, which is better? Live or virtual? Or a mixture of both?**

R: Before covid I probably would have said live will be better because you could access the patient and read the body language. But with covid everything has been switched to virtual. We know diabetes patients are high risk individuals so you want to desist from putting them together unless you can have a small group and see to it that the covid protocols are observed. But the other thing is you don’t want individuals being nervous so before you do such a thing you may want to find out from the patients what they want. I have patients who at the moment do not want to come to the hospital at all and that they prefer telecom but I explain to them that not everything can be done via telecom. If I say face to face is good and the patient feels uncomfortable, they will be in the group but all they will be wondering is if they are going to catch covid. I will say that at the moment, looking at everything that is going on, virtual will probably be a better idea. But you can find out from your patients what they want.

**I: If virtual should be employed in these times, which will be better? Text messages, video, radio**, **television or other means of communications?**

R: Any of those should work but you must ensure that your patients are really learning from it. Somehow you must ensure your patients are motivated enough. It will be good if you can have virtual but live interactions so that questions and answers can be exchanged.

**I: Which will be the best venue to host diabetes self-management education? Will it be the home, the community or the hospital?**

R: It depends on the individual. If the individual lives in a slam for example, am not sure it will be comfortable for everybody to have a session in their home. Everything must be individualised I guess, and If you want to have a small meeting in the clinic that is also possible. It’s difficult for me to give you an answer to that question, I think you must access your patients and find out what they want. But for example if you are designing a program for a national DSME program, then of course because it is going to be disseminated you want it to be uniform. You can decide to have it at the facility where they seek health care. It’s impossible to give you exactly where the best venue will be.

**I: With your experience, what can you say are some of the barriers to behavioural change after one has received DSME?**

R: If the expected changes is too much, I find that people even give up before they even start. A patient must be motivated and the provider of the educational program must ensure that, this will be dependent on their behaviour. We must make the experience enjoyable and make the patients understand that it is doable otherwise patients wouldn’t even bother. Also information overload can be a great barrier so as they say, keep it simple, and keep it short. Again the educational materials should be such that they can take it home.

**I: Please what is your assessment of the DSME administered in your facility?**

R: I would not say we are delivering standardised or structured diabetes self-management education but I would say we are doing in our facility xxx here . I know this from the feedback we get from patients and nurses and from the turn up for reviews on clinic days. It is probably because we have the specialists around, in the teaching hospital and in the polyclinic, and experience has also bolstered the effectiveness of our consultation.

**I: How can it be made better? And kindly share some of your experiences as a health care provider.**

R: (sighs for about 3 seconds) Well, I have specialised in diabetes for about 10 to 12 years, and it has been an amazing ride. I have been exposed to different presentations and complexities of the disease and I believe that more people have to be trained in administering healthcare in this field. It is very common in our country than we can think. I quite remember I had an encounter with one individual who had diabetes before hitting the twenties so, there has to be a strtegy that will disseminate education even beyond the diabetic population.

**ZOOM Interview with Diabetes Expert -DR 2**

**I: Can you tell me what you know about Diabetes Self-Management Education Support? What is the scope and what does it entail generally?**

R: We know that Diabetes is a chronic health illness or disease. Like any other disease, patients live with diabetes for a long time; hence, patients need to be educated on how to manage the diabetes by themselves because of the chronicity and nature of the diabetes. Therefore, the ability of the patient to manage his or her diabetes in his or own small way taking into consideration the discussion with the health care professional is very important in managing the diabetes. That is why the diabetes self-management education is key and useful elsewhere in helping patients to live better with the disease. The stage of diabetes health care education is underdeveloped and therefore needs to be scaled up.

**I: When it comes to diabetes health care education, which group of person do you think should administer the education to the diabetic patients? Should it be the health care professionals or people living with diabetes?**

R: I think there is no single answer. There are modules were peers have been able to deliver the education as well as the educators. You have to evaluate your peculiar circumstances and look at what works best for you. If you look at the peculiar circumstances of Ghana, which is under developed with limited scope, and lacks many educators. A combination of both is ok so that we will have core of diabetes educators, trainer or trainers who will now also educate peer support or peers of persons who have lived with diabetes so they become as an added layer of a way to administer the education. I think the combination will help because of our limited resources and limited nature of personnel and scope of education.

**I: Is lack of education the only challenge of Diabetes Self-Management Education? If it is, is there any ways to improve Diabetes Self-Management Education?**

R: I think most clinic or hospitals are not able to educate diabetic patients that visit their hospitals. Other hospitals do not also have structured educational program to educate diabetic patients. There are also limited educators to educate diabetic patients and there is lack of diversity in terms of personnel. There are also limited resources such as books, pamphlets, internet services, money and no policy redirection to support the education of diabetic patients. I think all these are challenges to the Diabetes Self-Management Education.

I also think we can improve Diabetes Self-Management Education by having a policy redirection, hence we should engage policy makers to understand that Diabetes Self-Management Education is key in managing diabetes. I think there should be an inbuilt lifestyle measures within the Diabetes Self-Management Education to help achieve a large success rate. The policy makers would also help in training more people as educators and trainers of trainers and also engage the training institutions to develop course contents. They also need to engage the university and other allied training institution to be interested in bringing out courses that trains persons to become diabetic educators. I also think the hospitals should petition to get more funding in order to buy more resources needed in the Diabetes Self-Management Education.

**I: What do you think should be the scope of Diabetes Self-Management Education and Support?**

R: I think the patients must understand what diabetes is and all the related signs and symptoms. Patients should be also able to educate other patients who may have similar symptoms. Patients must also understand that diabetes can give both accurate and chronic complications. They need to know how to manage the diabetes and understand that there can be referrals and they need to have eye check, legs check and other part of their body checked.

**I: What are some of the non-pharmacological education to diabetic patients?**

R:. Non-pharmacological education refers to mainly dietary habits and exercise therapy. It involves eating healthy balanced diets and exercising appropriately

**I: Do you think the face-to-face mode of education is better than the virtual mode delivery?**

R: I think the mode of delivery of the education depends on the client or patients. When the patients are highly educated, then the virtual delivery mode would be appropriate and helpful. When the patients are mostly illiterate, then the face to face would also be appropriate. However, the virtual education has become effective during this Covid-19 period.

**I: Do you think the education should be structured or unstructured?**

R: I think the education should be structured so that the deliverables can be set. I also think the education needs to be structured no matter the mode of delivery so that the message or information can be the same and the deliverables measured.

**I: Do you think all the education should be done at once or it should be held in bits (at least weekly or monthly)?**

R: I think the education should be held in bits so that the patients can sustain the information.

**I: What do you think are some of the challenges to patients adhering to Diabetes Self- Management Education?**

R: I think the challenges are multifaceted. One of the challenge may be inadequate information or education. When there is inadequate information, patients may not have the desired effect hence there would be poor in learning and non-compliance by the patient. Another challenge may be the lack of understanding or illiteracy on the part of the patients. Lack of financial resources on the part of patients and some socio-cultural factors that prevents patients from keeping appointment may also be challenges. Myths and other influences from the society may also be a challenge. There need to be an assessment mechanism to evaluate or test the learning and understanding of the patients.

**I: How has Covid-19 affected the Diabetes Self-Management Education in Ghana?**

R: I think Covid-19 has encouraged virtual education were appointment has been reschedule to limit the contact with patients. Most of the health care professional are running shift hence they may not be always available to deliver the education.

**I: Can you share some of your experiences in terms of Diabetes Self-Management Education and your interaction with the diabetic patients?**

R: I have not formally been involved in educating the patients. The nurses and dieticians normally administer the education transferring some of the unstructured information about diabetes to patients. Some of the patients usually complain and suggests that the doctors deliver the education.

**I: Have you had any formal education on Diabetes Self-Management Education?**

R: I have not had any formal education on Diabetes Self-Management Education but I did my MSc Project on Structured Education. I am also supervising a PhD student to do his project in Diabetes Self-Management Education.

**I: How long do you think one session of a structured Diabetes Self-Management Education should last? What are some of the logistics that are needed for the education?**

R: I think the session should last for at least 30minutes to one hour and some of the logistics needed for the education includes audiovisuals such as microphones, speakers, projectors and other materials like pamphlets.

**I: How would you assess the performance of the health professionals in terms of Diabetes Self-Management Education in facility xxx?**

R: I would say there isn’t anything special about the Diabetes Self-Management Education in Korle Bu. Most of the education is unstructured and I think we need to improve on it.

**I: Thank You**

R: Thank You.
