# Supplementary material for: Diabetes self-management education interventions and self-management in low-resource settings; a mixed methods study
Source: PLoS One. 2023 Jul 14;18(7):e0286974. doi: 10.1371/journal.pone.0286974 (PMC10348576; doi:10.1371/journal.pone.0286974)
Supplement: S3 File — (DOCX) [file pone.0286974.s005.docx]

**Discussion with Prayer/Policy Makers**

I: What are the most important current health care needs for those with cardio metabolic disease? What are your perspectives after accessing our health care systems about patients living with CMDs?

R5: I think they have their individual needs considering their financial status.

R2: I also think the issue would be how long I have to join queues to access healthcare services and how long I have to take a prescribed medicine. I know some have to take it morning and evening and they forget.

I: The long years you have to take the drugs is obviously a challenge.

R2: Yes, and if there is any side effect but the issues is when to stop taking the drug and also stop join long queues because anytime you come to the clinic you be worried because of this issues.

R5: I believe patients who have more money are able to receive good health care as compared to those who have little money. Patients who do not have enough money could even barely adhere to medication.

R3: I also think patients need motivation. That is what would really motivate them to even take their prescribed medication at the defined times. Some of these patients have more than one of these chronic diseases; hence, they need to be highly motivated to get health care.

I: Every day you have to think of your problems and if there is no money, it will obviously add to your stress.

R5: I would add that patients with these conditions also need social support. Patients basically have two needs namely; Physical and Psychological needs.

I: I will be more interested in social support, let’s go a bit further. What are some of the social support need of these patients that can help solve these challenges and manage their health conditions?

R4: The social supports ranges from support from families and friends. It is a chain of people.

I: For a typical Ghanaian along the shore example Bukom, shoko and korle Gorno. What do they have?

R2: I also think, ideally patients should visit the hospital with their relatives or other people. I don’t think patients are supposed to come for health care services alone so they could be supported by another person in the whole process. At facility xxx, we are using the card system and you need your card at each point. Some of them find it difficult to locate some of the department without the support of family and friends so they decide not to come to the hospital at their appointment dates. I think social support is very important.

I: We have now identify that family and close friends might be helpful. How about patients who may not have social support system from relatives and close friends? Is there anything the health care service providers can do at their level to meet these patients halfway?

R4: I think we need to adopt the biomedical - social model in helping patients. The health care givers need to be conscious of these things and develop a good attitude when meeting the needs of these patients. Health workers should empathize with them and respond positively to these patients. When patients sometimes come late to the hospital healthcare personnel should not be shouting on them but rather put themselves in their shoes and try to understand the reasons why he or she is late.

I: I think it is crucial because the response of the health workers can either sack our clients or determine how they feel welcomed into our system. Systemic or policy wise, is there anything that can be done to ensure that the health workers are more responsive or welcoming to these vulnerable clients who already have to go through so much before they end up at our doors but our attitude is sending them away. Is there any health systems or policy from the top that can be put in place to ensure that the healthcare workers are more responsive or welcoming to these vulnerable patients?

R6: I think we have these policies in place already. We only have to enforce it properly.

Continuation from second audio.

R4: There are a lot of polices in place, yet they aren’t being enforced because of so many barriers and factors. Most of the health workers are burned out always because they have to attend to more patients at a time. The ratio of health workers to patients’ ratio is lower. So, I think there should be policy on in-house training of health professionals on how to properly carry out their duties and the expected attitudes to be demonstrated in providing health care to patients.

I: Can we identify some of these policies that exists to ensure that health professionals give the best care to patients?

R1: The first one is Patients Charter. Most hospital have it pasted on their walls yet a lot of health professionals don’t follow them or read it.

I: We use the word policies to mean so many things. Policies could mean guidelines or instructions. We are looking at policy as a structure that that the health system have in place that empowers the health worker and the patients in their activities. These policies also reflect in the strategies of healthcare. The patients’ charter is a guide. Can we give any other policies?

R1: I would say the main health policy in Ghana. In relation to the cardio metabolic disease then we have the NCE policy.

I: Do we all know about the NCE policy?

R4: No please

I: it exists and we need to be taught about it. Do we also think there are specific health guidelines that in relation to the various health conditions?

R1: We have a lot of them. We have the standard treatment guidelines, diabetes, cardiovascular disease guidelines and a number of SoPs management developed within the various health facilities. Many facilities have developed their own specific guidelines for the various units. For example, when you visit the ICU, there are specific guidelines that one need to follow.

I: Looking at it from a perspective of what we see, how do you translate the policy, guidelines and SOPs to the care of the patients to make sure that most important health care needs of patients are really met?

R1: I think most of the health care workers are ignorant about what guidelines that are available or not. There need to do proper orientation of these current policies and intermittent training to health workers. As the reviews are done, we try to update them and apply current evidence and incorporate them into these documents. We also need to address the knowledge of the health workers into these policies, which could be helpful.

R4: I think immediately after the launch everybody forget about it. I also believe the facilities based SOPs among others can be more effective if only they are a reflection of what the real policies actually contains. This a way to decentralize it and get it work. Health workers need to be familiarized with the main policies.

I: So we should include them when drafting those policies.

R4: Yes.

I: How about pre-service training, how much of these policies are taught during our training? How much of these relevant health and administrative policies are considered?

R6: I think usually there is no proper feedback from patients in the hospitals. There are no suggestions boxes in the hospitals like there are in banks and hospitality centers. Patients who face hostile treatment from some health workers such as nurses and doctors dont know who to lodge their complaint to in the hospital. I think if these things are put into consideration it will help.

I: Practically, are you suggesting what is done in the banks? How best can we ensure that patient’s feedback is properly captured and they don’t feel legalized?

R2: At the Facility xxx, we have a Pro complaint desk. Patients usually visit the Pro complaint desk before they go to the laboratory for their lab test and they lodge all their complaints. The complaints are usually about their waiting time, and the health worker’s attitude towards them. They sometimes even record. There have being instances where a patients went to the consulting room and the doctor harshly told her that he has closed whiles it is not even 2:00pm to close so the PRO have to step in to solve the issues. The Pro receives all these reports and complaints and address them.

R4: This may be working well in the urban areas as compared to the rural areas. Normally, patients in the rural areas find it difficult to speak up because they see a doctor as a “dummy god”. They are afraid that, they might not get any care if they complain. The question now is how to make it practical so that it could get across all areas.

I: I believe the practice has to be intentional and needs to cut areas even in the rural areas, so they know it is important to the facility and patients do not feel victimized. We need to get people who would be held responsible to address these feedbacks. How best can we make this work in the rural areas especially?

R1: We tried a strategy in the maternal unit in Tema amidst challenges in electricity and water supply. We used to have an open forum for men whose partners would come and deliver. We called it a diagnostic clinic where moderators are rotated and feedbacks are recorded and reported by these patients. It was an open forum where we could pick up their challenges. The discussion where usually on Twi or Ga based on the moderator’s preference. That was a good way of picking sentiments because patients might choose not to write or call the toll free line. We have to hold a community forum where the community could share their ideas without the intervention of the health workers. Within the Ghana Health Service, when we want people to undertake a activity we have the performance review score where we put indicators for health workers to report on it. These numbers are compared annually so it is like a form of competition and for indicators, which are usually red; it means that the region would hunt them down, because the region is like a supervisor. We have a sort of scoring system based on the complaints and feedback from the patients, which can be negative or a complement. This is a way of making the health facilities feel the feedback and it can go a long way in improving the health service. It is a policy directive. Each districts have these indicators, which must be addressed annually and they are striving to ensure they are not red. .

I: How do people access health care- all possible options? We are talking about cardiometabolic disease in our environment. What are the various options available to people who have diabetics, hypertension etc?

R6: I think the closet health care facilities people access mostly are the hospitals and herbal clinics. From my experience, I know a lot of diabetic patients who usually prefer herbal clinics because they feel the herbal clinics cure the diseases rather than visiting the hospital where they usually manage the disease.

I: So you mean the patients want the cure and not the management.

R4: Patients normally visit the herbal clinics because of the regular advertisement made by them as compared to the hospitals where they manage the disease and regulate their medication. I think the legislators should take a stand on the advertisement of these herbal clinics, which has become like an epidemic in our part of the world, in the buses, marketplaces and in most areas of the community. Most of these advertisements are misleading and the most of the illiterates and vulnerable ones fall for them.

I: Is it not a form of education these herbal clinics make when selling their products? Do you think the hospitals can also do the same to educate patients about their healthcare services? Normally the herbal clinics do the advertisement without even meeting these patients, they look for every opportunity to sell themselves and products. The hospitals on the other hand, wait for these patients in the consulting room before they educate them notwithstanding the challenges faced by the hospitals. Hence, I think the hospitals should step up in the education because we understand the from the policy level that the first point of these disease is the knowledge and education This education could be done in different forms usually from the policy level. How much of this is supposed to be done knowing very well that hospitals wait for patients to visit before they educate the patients?

R4: I think the public health should focus on approaches that requires minimal efforts on the part of the patients. The hospital should change their approaches so that patients would see positive effects. Hospitals should also not be comfortable with the conventional old tools and realize that times have change; hence, they need to revise their syballus and ways of doing things. Hospitals need to meet the needs of patients to ensure proper feedback from them.

R2: I have my reservations about these advertisements of herbal clinics especially on the television. I believe that the hospitals do not have to wait for patients to visit them before they educate them. They can use public health workers to educate patients even before they visit the hospitals and the communities. I also suggest that the hospitals could also buy or get airtime on the various televisions stations to have health education programs like “Tele Nurse on Crystal TV” for patients. I think other television station should also do the same rather than showing telenovelas example GTV, TV3 and GHONE television.

R1: From the policy perspective, one main challenge, I have noticed is that funding for public health activities are very limited. There is almost zero funding, so health facilities figure out a way to send out public health workers for community health education. Most hospitals should be able to allocate some small funding to support public health education because it is crucial. They can get some big agencies such as the AGIs, or Telecoms to fully fund health promotional activities. It would be difficult for only the health system or hospitals to support these public health workers in the education of patients. Most of these public health workers are also limited in number and barely have enough time to come on the television to educate patients. But, I believe the hospitals could devise workable solutions. Residents for instance have outputs they are being measured and if they make it a requirement that as part of your output you should have giving a number of health talks to patients so that we can tie up value for patients. Practically, health professionals do not have the luxury of leaving the health facilities to sit on the television for a health talk, even though it is crucial, because they consider it as non-clinical. Most of these public health workers also need so much supports from our clinicians and specialists delivering their messages because they are few. [I: I think from the policy level, the public health nurses are supposed to be aware that they are trained to provide public health education to patients. We need people to be visiting the communities to educate people]. We need to prioritize public health aspects in Ghana.

R2: It is serious and from where am coming from, the Methodist church has received a communique from its authorities to build sick bays in all their societies. We have a group called the Social Services, which offer services and other things to the communities. A typical Methodist would not eat before coming for a Communion Service. Some have diabetes and hypertension and some would come to church early and would faint during church services. We sometimes start at 8:30am and close at 12pm so we need these public health educations to help solve these issues in our churches.

R4: When we consider a typical rural setting, we used to have people gathering in the various village squares. The hospitals could organize a public health education during these community gatherings if these public squares still exist.

R7: There are different divisions in the Ghana Health Service but I dont know of the teaching hospitals. The promotional health service is currently the 11^th^ division. We are gradually moving from the public health nurses running that function to the community nurses. I do not know how versatile they have gone through the system whether they are now part of the community or the CHIPS. We said we want to get to the people and we are already there but I think the problem is the caliber of people we send there. We use to train the CHIPS to distribute condoms and give us feedback. I was ask to go and talk about ENT when they were training HAG nurses in WA. The people who organized the training new how it will be useful. The community health nurses in Navrongo those days also started lectures on ENT so need to question the component and composition of the health system. There are more percentage of people who need the services than those who offer the services and it is an issue.

I: What community and healthcare resources currently exist (and which ones do you currently use) to support management of cardiometabolic disease?

R1: I think we need to empower the lower level of health care. There is a whole level for health care division that trickle down to our TRIPs and health care centers. I know within the districts levels, there are health promotional officers and public health nurses but then when we go down to the community, and there are either the CHO and community health nurses.

: Are we saying that the public health nurse aren’t supposed to do health promotion?

R1: No please, that is not what I actually meant.

R7: The public health nurses used to do the education until the health promotion became a division of the Ghana Health Service. However, I believe the public health nurse should work together with the health promotion division.

I: Who should take care of patients with the cardiometabolic diseases such as diabetes and hypertension at the community? Can we talk about it at the primary prevention, which is before patients get diagnosed and what services are available? At the secondary level, when patients have the disease, the source of management they need and at the tertiary level where the patients have had complications and they need care? What is available for them? What structures do we have?

R7: I think there is actually nothing in place. I do not think there are evidence of documents at the Ministry of Health that support most of things we have discussed. We are talking about cardio metabolic complex. Has it being raised in any of the documents we are taking about from the ministry level and has suggestion being giving as how players should fit in concerning the issues of cardio metabolic diseases. When it happens like that what will be the role of the Ghana Health Service in that document?

R1: Service delivery

R7: Service delivery is a statement so I think they should rundown and from there they can develop the guidelines like the STGs. The STGs have been able to provide minimum package for simple thing now. We need to also properly spell out the role of the CHIPs at managing this disease at that level. The District Director should know the minimum package at that level.

R1: Well, I think a lot of people at the Ministry level are ignorant about the policies, strategies and guidelines about the guidelines that have been developed. Dr. Doku and his people in Ghana launched these guidelines two years ago. We need to link the various levels and ensure that when each policy is created but the question is, how do the people know about it and what their various roles on all the things that have been outlined in the documents.

R7: We know that the most of the expertise are in the teaching hospitals. We should ask how we have attempted to tap in and use those expertise, so that teaching hospitals will take that policy and they try to let it reflect on how they do things at the teaching hospitals .How do we get the clinical component of the Ghana Health Service not to be dependent on the expertise of these teaching hospitals. They need to be expertise transfer or sharing because most of the times, they would say it has being launched and published but it sitting in the teaching hospitals. [I: Not only that but it is also in the regional hospitals such as Ridge Hospital and the Koforidua Hospitals]. I don’t know about any regional hospitals who are part of these policy awareness. Usually, when someone comes around and may want to highlight these polices and create awareness, he only invites a few regional representatives like Ridge, Koforidua and sometimes Ho regional hospitals.

R1: I believe we need to get these policies also to the districts and in the dynamics of the rural areas. Within the region, the structure should be carry out at the district levels and they should ensure that the districts are engaged. It is very possible to engage them.

R7: Please, who would ensure all these things would be supervised and monitored?

R1: The regional health directorate and the district health directorate would be held responsible. The training is for the CHIPs and they are given supervisory role. The regional directorate are the ones who develop the guidelines for the districts.

I: One of the challenges I also see is that, when we take the Ghana Card into consideration for instance, we could examine the policy. This policy was sponsored by the World Bank to a large extend. In translating it into strategies and guidelines, it was supported by a donor. These donor want global recognition and expect that we bring the policy down to the low level.

R1: Ultimately, the country is not contributing to this process, so we end up meeting our own human interest. From my work experience, since I joined the unit GoG allocation was GHc200.00 and the last time GoG allocation in the GHS came to the health workers was around 2011. The top leadership should be able to disperse resources to the various levels. I don’t think it is fair for the regional staff of the GHS, whether the clinics or the public health are left to find their own way of keeping their regions healthy. Meanwhile people are eating healthy diets, they are taking the alcohol, and they are not exercising, and are not checking their health status and they visit the hospitals to overwhelm the health workers. [R7: I think some hospital are being given money from the NHIA]. Well, ultimately, how much would that end the hospital through to the outreach we are hoping they establish? This is because the hospital is 80percent clinical so they would ultimately depend on the money that are coming from the clinical bit. Hence, the hospitals would have to prioritize the community-based service, which is a pro bono. Most hospitals have to look at the transportation and allowances to be given to public health nurses who would be sent for the community outreaches. Hospitals may find it difficult to sustain that service if they do not get extra support and funding.

I: Practically with CREATE, we want to propose something that is workable. We have been able to identify and talk about some of the challenges, but we want to propose something that is workable that even if we can do it in every places, we can test it in communities to see if it will improve self-care and care outcomes for cardio metabolic diseases. What are some of the things we think we can do to make living with cardio metabolic disease a death sentence, taking it from where the people live to the highest level of care?

R7: We could start from the highest level to the lower level.

I: I want us to start from the bottom line. Where do you come from?

R7: Sunyani.

I: I mean your hometown.

R7: Nsokor.

I: The market women at Nsokor who are happily trading and coming to buy their foodstuffs and living their lives. How can we have a system that makes sure that they are healthy, and if they are developing diabetes or hypertension, there is a system that picks it up quickly and takes care of them in such a way that they do not develop complications? In addition, even if they develop complications, there would be adequate support for them so that they would not have later issues?

R7: I think there may be disparity in the system at the ground level where most of the solutions would be effected. This is because what may work in one community may not be able to work in another community. Therefore, I believe all the things we need to do from the ground level must come from the top, which is why there is leadership. I also want us to know that the main sector responsible for health is the Ministry of Health. There would be other collaborators and other linkages like quick side government health facilities, the military, the police and the VRA who also do certain health. However, I believe all their health deliverables would come into the mainstream GHS that will be championing them. If the ministry has a policy as we have being made aware now and we have found out the component that relate to CMDs, then we should look for it, open it and try to see what in it, know the HN components of that policy, the strategies, the technical areas and task sharing. Obviously, not everyone may understand the psychology and pathos of these CMDs. Is that when we are going to pick middle levels that’s Pas and push them to the community. It is not enough to think that people must mess up with basic things that could have been corrected from the very beginning, before they bring the issues to the health centers, it would have already been messed up. I strongly believe we need to spell out clearly the human resource, training, risk, and minimum package. We also need to state what we expect CHIPs compound to have, and if they have it how often should they replenish the consumables of the strips.

I: So beyond the spelling out, what else can we do? We need resources.

R7: Well, we have a policy and the policy just like a law seems so big for the lower and middle level to understand. We have to break it down and develop strategies or guidelines, protocols, SoPs that would be bolder. When we are done am sure at each point we will get what each levels are supposed to consume. We should also critically ensure that people are doing what they ought to do. In addition, dont forget there is already an existing structure, so you will only reinforce the structure and make sure that players there play their roles to success.

I: How do we ensure that they are doing what they are supposed to do?

R7: We should put in M&E that is monitoring, supportive supervision and proper feedback.

I: I don’t know why you aren’t talking about the resources. Can we talk about what resources they need and how to give it to them?

R2: I think the resources should come because if I do not have tools and materials to work with, then how evaluation would be done?

R7: I believe I mentioned some of the resources as well. However, because of donor factors, you really have to make a point that you need these resources and provide reasons on how you would judiciously use these resources. In the past, there used to be so much money pumped into the hospitals yet nothing was been done with such money. None thought about IGF, until NHIA appeared. [I: Yes, the NHIA is another resource.They made sure every level, the community; district and the regional levels have it]

R2: I think some communities are doing well. In Chorkor, we had Stephen Appiah coming to encourage the people to register for the NHIS free.

R7: I don’t think the NHIS is the problem actually.

R2: Well, I also think the NHIS is also important because you can be able to attend the hospital.

R7: I have been working with the NHIA for some time now as a clinical compliant audit. We have been to all the regions in Ghana and yet there is no private or public hospital in Ghana that is not doing co-payment. So for me, even renewing my health insurance is not even motivating because no matter what it is, I would definitely have to make additional payments when I visit the hospital. I believe we are not really supposed to make any payment when we visit the hospitals. [I: I don’t think you should believe you don’t have to make any payment because it depends on what you go to the hospital for and the benefit package for that condition.]. According to the international health insurance backed by law, there was not supposed to be co-payment. I think facility xxx wants to justify it because they are doing it.

All respondents: No

R1: Practically, which countries in the world comparatively practice what you are saying? The NHIS selects services that they cover.

R7: I think you are going to the extreme. I even think most of the cardiometabolic diseases are covered by the NHIS.

R1: The NHIS do not cover all the services in the hospitals. There are some laboratory tests that need to be paid for in the hospitals.

R2: Yes, that is true because at Facility xxx, the NHIS only cover selected services for patients like the full blood count etc.

R7: I believe this would not have been an issue, if the law formulators had considered and highlighted what services should be covered by the NHIS. Most people are at the district level and a good package could have been received by the people there. At the regional level, there could be a consideration of half package or benefits of the NHIS because we may have the Regional Coordinators or the Regional MCEs visiting health treatment from the regional hospitals. Then, at the teaching hospitals, there could be at least 20percent of benefits, so that we know that 80percent would be out of private insurance or money to be paid for health care services. I don’t agree that 100percent at the national level is the best practice and co-payment shouldn’t be at all levels.

R2: The whole NHIA has a little issues because of its political agenda. The realities of our patients is that they go to the hospitals and they pay for services. Usually, some of the patients are not even able to access a service at the hospital. Usually, what is bringing down a few pilot is the GHC5 stripes that people cannot even afford. So they are screening BP free, for a lot of patients, their BP are monitored yet the blood sugar test is less. We cannot disregard the finance, it is a key especially considering the chronic nature of the disease and that is why the hospital resort other alternatives.

I: Most of the patients are visiting the herbal clinics and even the churches. The license herbal shop are treating diseases they are not supposed to treat and dispensing medicines they aren’t supposed to give. The question is, should we and if we should how can, we bring these people on board to help us care for people with CMD.

R7: When you saw the policy and the role of CMD, did the policy tell you the role of the private sector, town, traditional areas, CHAD, or the local or quasi government in managing the CDMs. I think they should just copy from that and practice it because that is the starting point

I: The Alternate sources of Health care for the people with CMD. How can we bring them on board to propose something that would work for taking care of people with CDMs? As I said not when they are sick but from the preventive order as we spoke about the herbalist advertising on the radio Can we intervene in any way to make sure that the formation is modified to meeting up with their profit.

R6: We cannot disregard the fact that some of this herbal medicine works, some do work. So I believe that there should be a legislation or legal medical documents or regulation backing these groups.

R7: I think there is a regulation but it needs to be enforced. These herbal clinics are being licensed.

R6: I think as far as health is concerned, everyone is supposed to be regulated when we are talking about health.

I: There was a press conference by GMA especially at the time where people were claiming to cure many things and calling cancer patient to stop taking medicines from the hospitals. So we brought the media, tried to educate these groups. So we took it from the scientific – technical view and side of the law and we did a lot of stakeholder’s engagements yet we are still at where we are.

R4: I think the regulation needs to be bold. There is a country in Africa where they ban these religious groups. Most of these religious groups are also part of the problems. A lot of them are in for business. Most of them take advantage of the vulnerability of the patients even though the public needs their spiritual enlightenment and nourishment. They mislead them to make themselves rich. The regulations exist and when we implement it, they will all stop. We can’t deny that we need these people but we need to identify the right ones and sanction the ‘fake’ ones to serve as deterrent to others.

R5: I think in dealing with these chemical sellers who are supposed to sell only Class C drugs but they sell Class A and Class B drugs, we need to solve the issue by enforcing the regulation so that patients would not be able to even get the Class A and Class B drugs at the chemical shops. When you go to villages there is a policy that within a particular kilometer, there has to be a health facility. Yet when you visit some communities, after some series of meters, you would not find any health facility. Hence patients with this chronic disease would have no choice than to visit these chemical shops to buy drugs without any clinical consultation or investigation about the intake of the type and amount of drugs to take. In order to address all these issues then we need to solve the problems and ensure that the regulation would work.

I: If these chemical shops are not licensed to sell some particular drugs yet they sell them, is there a way for example, we can say that before you give the medicine to these patients, then you would have to test their blood or sugar levels?

R5: I do not think they have the technical know how to do the test. They are not well equipped. There are a lot of herbalist who sell drugs with no education but has more experience. In order to address this, there should be health facilities within short distance so that the patients can also visit. Otherwise, it would be very difficult to stop herbalist from selling their drugs and even stop patients from buying from these chemical shops.

I: Some year back we claimed that maternal mortality is worrying us so we decided to train TPAs We spent a lot of donor money to train them yet the mortality was still high. Sometimes, it is comforting that there is an alternative, but the alternatives must be a safe alternative.

R5: I think the nation should arise, address the problems of the community, and provide health facilities for community with a large population and a lot of households. The Food and Drug board can also help in handling this issue of drugs sold by the chemical shops and herbalists. I think when we all work in building the blocks; we would be able to address these issues.

R3: I also think no matter the regulation or legislation, over the counter will not stopped totally, because these people are after the profit no matter how they make them. Instead of the patients going to see the doctor they will go to the pharmacists or chemical shop. He or she will explain everything to the pharmacist and instead of them advising them to see the doctor, they rather give them drugs thinking it will help them so no matter the policy or the legislation, over the counter will not stop even though there will be education with rules guiding them.

R2: There was an incident in Abokobi were a pregnant woman was feeling lower abdominal pain and went to over the counter medicine providers. They decided to give a prescription and apparently she had an abruption and passed away together with the baby after taking drugs from a chemical shop. The health directorate took advantage of the situation and made the NCE and the district assembly aware of the problems caused by these chemical shops in the communities. We need to strategize so that we can solve most of these issues. There are security agency like the police and immigration officers at the district assembly who could be used for the enforcement of the regulations concerning the chemical shops and furnish the districts with updates. I think the district health directorates should hold series of meetings with these chemical sellers as well as these pharmacies because they dont even know that they have laws backing them. There is a limit to the kind of things they can do. We had a program where these chemical shop attendants and herbalists were invited to sell condoms and take the blood samples of the patients around and they were very happy about the program because they learnt a lot and people started referring to them as doctors. Even though, this was a way to empower them, we were able to monitor them as well. Every month, the district health directorate sends personnel to check the operations and performance of these chemical shops. When we tried this, the chemical shops started referring patients with this chronic case who were not attending the clinics. Hence, I strongly believe some of the collaborations at the local level is very possible. We need to check our approaches and speak to the minds of these herbalists using the law. Therefore, we do not have to wait for an issue to occur before we can attend to them. We need to let the herbalist know that the hospitals and health workers are easily approachable. At Pastor Obinim’s place near the Tema General Hospital, he was camping an eclampsia and pre-eclampsia pregnant woman in his church, which was not the best action to take so we had to send police to the church. We made it known to him that religious leaders can pray for patients when they visit them and should encourage the pregnant women to visit the hospital regularly instead of holding them in the church. On top of that, his small pastors came to the ward and shred paper funs and the women were chewing the funs instead of taking the anti-hypertensive. We told him we dont mind if he will come and stand at the gate and pray for them after their CS. In addition, he should not hoard them and let them fit before bringing them to the hospital. I also suggest we should try and approach these alternative providers who may not be the main streams and not just wait till issues come up before we try and solved them.

I: We taking about trying to do things a bit different, how open we are to these new ideas from the policy mainstream and how to trigger it.

R2: My brother spoke about the regulation in the religious sector so we have to go by that. I took lessons from something one of our Rev. Minister did during his encounter with one of the patients. He advised her to visit the hospital even whilst we were praying for her. We need to look at the holistic approach and integrate the praying and fasting aspects as we encourage our members to visit the hospitals whenever they are unhealthy. The patients also need to be educated and enlightened as to what they need to do. As part of our pastoral care and counselling, and as a Chaplain at the Facility xxx, we visit our patients and pray with them. We should encourage our church member to visit the hospitals and take their medications as well then we could continue with the prayer and fasting for our patients.

I: It seems very easier for the religious body to support in managing the CDMs but the issues has to do more with the herbalists and chemical shops. What do you think we can do to address these issues?

R2: Unfortunately, these herbalists are seeing themselves as Pastors because of the spirituality they add in selling their drugs.

I: How do we deal with the component of the herbal and the healthcare medication?

R4: There seem to be a gap between what takes place in industry and what takes place in academia. We know that now knust are training herbal pharmacist and we have the research center at Mampong. I believe we need to bridge this gap to help solve the issue.

R7: I think there should be collaboration with these herbal facilities.

R4: I also think we need to be empowered to make informed decisions on a personal level.What is working in other jurisdiction could also work in our country.

R1: I believe it is possible when we are being educated and empowered. This is because throughout the Covid 19 period, there was no record of Cholera in Accra because people were adhering to the mandatory safety protocols of healthy living. They were been forced to use sanitizers and wash their hands with soap and water regularly. The health education would become part of our lives when we frequently do it. There are now health education happening in schools and our children are being taught how to keep good health, which have become part of them.

I: The truth is that when we do the education, we may not see the returns immediately, so people tend to relax or give up on it. However, it is the one sure thing we need to do. Sometimes, we all need motivation or a reminder.

R1: I also believe with the education, we can use patients as health educators so that the other patients with the same condition can also relate with the issues better in the diabetic clinic. Unfortunately, in our culture, we barely talk about our disease with other people because of fear of been stigmatized. However if patients could stand out and make this education, this would go a long way to help manage the disease.

R7: This is true but hasn’t been part of us in a long time. We are now learning from the HIV/ADIS and its management.

R1: It has been difficult getting a survivor of stroke to do a one-minute video for it. It has been two years now. Sometimes, we do get some diabetic patients willingly to share their experiences.

I: What self-care deliberate strategy and management would work? A lot of patient’s management issues are outside the consulting rooms, how well should we deliver these self-care messages to the patients and what are best way to deal with it.

R5: I think it should be a collaborative effort from the community to the hospital based. We need to use all the stakeholders, the use of CIC where patients would be encouraged to visit the hospitals regularly. At the facility level too, the health promotional could be use and the telecommunication messages could be sent to people. We also need to exhaust all the areas as much as possible. This is because the self-management approach is not a one time event, it involves a series of management coming together to create an outcome. If we are using one approach, it will not work but when we use series of alternatives, it will work.

I: Thank you all.
