# Supplementary material for: Diabetes self-management education interventions and self-management in low-resource settings; a mixed methods study
Source: PLoS One. 2023 Jul 14;18(7):e0286974. doi: 10.1371/journal.pone.0286974 (PMC10348576; doi:10.1371/journal.pone.0286974)
Supplement: S4 File — (DOCX) [file pone.0286974.s006.docx]

**Transcription from facility xxx**

I: **Today we are at facility xxx to interview a manager**

**I: Please you are welcome.**

R: Thank you.

**I: Please I would like to know something brief concerning diabetes self-management education you know.**

R: It is the standardize form of education for patient with diabetes. They take the patients through the various areas of diabetes they are supposed to know which will help them achieve their target. I have not read the full document but am assuming they will talk about education, management of the disease that is how the diseases is brought about, the care for diabetes patients in terms of knowing how to measure their own glucose, the essence of having their gluco meter, checking sugar and managing their food. There should be a component about how you eat, choosing your food, foot care, eye care and recognizing emergencies of diabetes either hypos or hypers and things that will worsening the disease.

I don’t know whether there is supposed to be a component about complication of the disease or not but if it have to do with education am assuming it will cover what I mentioned.

Is your question specifically about that standardize document or diabetes education in general?

**I: The standardize document.**

R: I don’t know much about that particular document but when it comes to diabetes education, I do.

**I: When it comes to diabetes self-management education, which health care professionals do you think should deliver this education?**

R: I don’t think it should be only one category of health care professional to deliver the education. First, whoever is diagnosing the patient must start educating on what exactly the disease entity is about because I believe that education is key and if you educate the patient well irrespective of the disease condition, they make informed choices. If you let the person know the consequences and he or she decide to go in another direction, that is an informed choice because the person becomes aware of the complication he or she will get perusing that direction. I believe it is the responsibility of the doctor doing the diagnosing to educate the patient but because the clinic are busy any other health care professional can support but if the nurses are enough at a place one can be detailed to support with the education.

We also have health educators who can also deliver if they are well educated. I think it is a team work not just one particular health care professional but just to make sure the information is standardize and all at the same page.

**I: What would be the ideal form of delivering this education? Should it be virtual or face-to-face? (**R: In the contest of covid**). We will come to the contest of covid.**

R: In the ordinary contest.

**I: Yes.**

R: In the ordinary contest, I believe most people prefer face-to-face because if you look at their educational background, majority of the people we have would prefer to be there and listen. I do many health talks in churches etc and you realize that they always prefer you being there so they can ask question and aslo hoping to get answers but because it’s a finite period and the group being large, the bold ones are the ones that come out with the questions. I believe the group and face-to-face are good but the sizes of the group should be taking into consideration because if it is large, few people will come on board but I don’t know exactly the style of this DSME but I think face-to-face is definitely better than the virtual looking at the clientele we have here. The group will also be better for the people to realize that they are not the only ones going through the hustle of not being able to afford their drugs. This shared community help them emotionally in addition with the one on one with the doctor. Support groups have been proven helpful with other diseases and hence must also be welcomed

**I: In the contest of Covid 19, do you think it should be done virtually, face-to-face, in groups or one to one.**

R: The question we need to ask about the clientele is whether they are conversant with zoom and Microsoft themes. I spoke with another consultant yesterday, she was telling me about how they tried to do telephone consultation, and the challenge was that most of them were not having their phones around them when they being called. We need to find ways of doing things safely because there is a new normal and the covid have also come to stay. If it’s face to face that majority wants it has to be done in a safe way and ensure that people derive the benefit. In addition, groups can be done were social distancing and the wearing of mask are observed. On the other hand, in a different setting where there are well-educated people, online held. An example is the CDC online program, which is purely done online in the United States when the covid started and most people have signed up to that, but don’t know if it can be easily replicated here.

**I: Where do you think such education should take place? Should it take place in the hospital or hiring of a conference room (R: A conference room outside the hospital) Yes, or maybe go to the communities. Where is the ideal place to educate patient on diabetes self-management.**

R: If you say an ideal place then it means you want one answer **(I: Yes)** if I were to rank I would say the health facility is an Ideal place because that is the central meeting point. If you even go into the community there still have to be a central meeting point where you gather people who are having diabetes and get someone who will bring the people together, you can’t just go there and say you are doing diabetes self-management education. It is most likely to certainly start from the health facility but in village or community where the health facility don’t have a big space to accommodate them and they have a big church where all the patients can meet, then it ok to have the group session there. In terms of meeting point, it should be easily accessible central meeting point for the particular clientele in discussion, so for us here we have a lot of open place on the compound and corridors where it can be held but for other groups different places might be considered.

**I: How would you want the education to be structured? Would you want it in a day with the patient or in bits, probably twice or once in a month? Which one would you think would be better?**

R: There are several topic and areas to be covered. When someone have the diagnosis, it is a journey depending the control of the person. He or she might be asked to come in a month, two or three month time, so within those times that the come for the review and we realize they are stable we quickly sort them out for the doctor, and who ever will be handling the education bit can then go in and join. If it is a group base, somebody might have heard that aspect already but I think it becomes reinforcement, so certainly not everything at ones.

It must be structured in such a way that if you had educational bit on the eye, the next time you might be doing food and if at the end of the session you give an opportunity for them to discuss both the eye and the food then you can always feedback on what they have learnt previously. I don’t think it will be good to bombard them with everything at a sitting, perhaps an overall at the first meeting and subsequently you zoom in for each of the aspect of diabetes management.

**I: When we come to facility xxx, how would you access diabetes and diabetes health management here? Are you doing well or not?**

R: It will be difficult to do an assessment objectively because we have various category of staff here that are medical officers, senior medical officers, specialist and consultant. It also depend on how the patient receives the information. I believe that elsewhere limiting factors are cut out but here they are just too many, we have the herbalist, pastors and people not being enrolled on the insurance. These are all factors but elsewhere almost everybody is on the insurance where they will get the insulin and their food being labelled and you can easily calculate how much you need to take. We need to find a way of doing things here because if am to educate a patient I will do that but not as deep as a group session and will find out if he or she has bought the glucometer. Mostly their responds are that they are waiting for funds to purchase the glucometer so they are giving glucometer from outside were the trips are not available in Ghana because they only bring 30 strips after that they have to get another glucometer so the issue is, how the person will be able to monitor his or her sugar. In measuring their education, it’s possible that one will know a lot if he or she have being there for a long time but translating what they have known into better outcomes, there are so many confounding factors which will determine it.

**I: What do you think impedes behavioral change when it comes to diabetes self-management? What do you think prevent patient from doing what they have been told to do despite the fact that it have been hammed on several times.**

R: I will say culture, because they are likely to listen to whomever they hold prominent in their life as compared to the doctors. Example, a patient is more likely to adhere to what the doctors says when he or she thinks that is preeminent over what the pastors says, but on the contrary, somebody who thinks what the herbalist says is more important in his or her life won’t listen to what the doctor says. If you decide to even donate the glucometer and give them the medication, they will still listen to the pastor. When he even instruct them to desist from taking their medicine on the purpose of been healed spiritual, they will adhere to it. There are educated people who will still listen to what the pastor, herbalist and traditional priest says, so I think culture is an issue of discussion.

**I: Are there any other limiting factor (R: There are several, I thought you wanted me to rank it) No.**

R: In health, we have what is termed as health numeracy, which talks about people being able to understand what the disease is about so then **educational level** becomes a limiting factor. **finances** is also a limiting factor because it indicate whether the person is able to afford their glucometer, strips, needles, both recommend, cheaper and effective drugs and if the person have insurance. Another factor is the will power of the person because some of them decide to take in soft drinks at funeral occasion’s whiles they are not supposed to drink them due to their condition; they do this because of pressure from others.

**I: What do you think we can do to improve upon diabetes self-management education in this facility? What can we do to make things better?**

R: As I told you my interest have being about prevention. Somewhere 2017, we always had a crowd of people waiting to be attended to in the hospital. This people included patients and their relative. The medical officers, residents and specialist then take the opportunity to educate them on several issues, it’s also an avenue where people can be around and have a piece of diabetes education. Mostly it is centered towards those who have already being diagnosed but I think we can also target those who have not being diagnosed and teach them about the prevention aspect. In addition, there should be a screen at the OPD where video sessions are showed repeatedly. I also think there should be a well-structured diabetes educational session for our clients here. It is not something that is new to us here because in times past the nurses started a few group session where they educated the clients. Dr xxx started a few group sessions.

**I: As a head of department, what are some of the logistics necessary to help improve diabetes self-management education in this facility?**

R: I think this particular facility need chairs and rooms because we are heavily constraint for space for consultation and the patients. If something of that sort is to be done, I will certainly say it should defer to the afternoon where there is a lighter load and fewer people on the ground. We will also need dedicated chairs so that there will not be the struggle for chairs with the patients who have come for consultation. Also, they should be particular about selecting the time to organize the session so that there isn’t too much pressure on the department and other logistics. In the afternoon if, they need some chairs they can get it because by then there will be fewer people on the ground and they can have about hundred (100) chairs if they want but in the morning, it might be a challenge to have that logistics for them. If it is done on, monthly basis then certainly that can be accommodated because everyone will then be aware and the necessary logistics will be put in place.

**I: It is mainly the space to organize such an education sessions.**

R: We have the space but the problem is the chairs. The chairs here were brought because of the study and as soon as the study is over they will be moved to their various places but the space will be available. If we have dedicated chairs, we can easily rearrange them for another study and later pack it up when the meeting is over so focus should be on the chairs.

**I: Are there other logistics that you think will be necessary (R: For the education) Yes.**

R: I don’t know whether you are asking from the departmental point of view or from the educational point of view because if it’s about education you certainly need things that you will use to educate the patient like samples of the needles, pens, food items among others.

**I: Yes about the educational point of view.**

R: You will need things that the patient can easily understand and read. In addition, audio visuals will help educate them because they can be learning from the watching of movies in the local language.

**I: Thank very much for your time. We have come to end of the interview.**

R: Thank you.
