# Supplementary material for: Diabetes self-management education interventions and self-management in low-resource settings; a mixed methods study
Source: PLoS One. 2023 Jul 14;18(7):e0286974. doi: 10.1371/journal.pone.0286974 (PMC10348576; doi:10.1371/journal.pone.0286974)
Supplement: S5 File — (DOCX) [file pone.0286974.s007.docx]

**Transcription from FACILITY YYY**

**I: Good afternoon. Today we are at FACILITY YYY hospital to interview one of the doctors on diabetes self-management education.**

**I: Hello, good afternoon.**

R: Good afternoon.

**I: Please what do you understand by diabetes self-management education? What does it entail?**

R: I think it's about educating diabetic patients on how to take their medications and also take note of any side or adverse effects. For example, if they are going through hypoglycaemia, they should be able to identify those things and then report to hospital in case of any problems.

**I: Thank you.**

**I: Who should give diabetes self-management education? Which health care professionals are to deliver diabetes self-management education?**

R: The doctors and the nurses should see to that.

**I: The doctors and the nurses?**

R: Yes.

**I: How do you think diabetes self-management education should be delivered? Do you think it should be delivered in a structured form or an informal way? How do you think this education should be done?**

R: I don't know what you mean by structured.

**I: By structured, I mean, you have a sort of template that you follow in given the education. Or you just deliver the education informally in an ad hoc manner more or less.**

R: I give it depending on the patient's medications. So if someone is on Metformin, am not likely to go into probably hypoglycaemia, because normally they don’t go into hypoglycaemia. I just talk to them about the side effects of Metformin. If they are having any other problems with the medications, they can come back and discuss, but if someone is other hypoglycaemic agents, then I tell them about the hypoglycaemia. The signs, the danger signs and what to do if they feel like they are going into hypoglycaemia.

**I: How do you think diabetes self-management education should be delivered? Do you think it should be delivered virtually or on a one to one basis?**

R: I think it should be a one on one basis.

**I: When it comes to facility yyy, how do you think diabetes self-management education is like generally?**

R: Do you mean how effective it is?

**I: Yes, how effective it is.**

R: We do get a few people who brought into the emergency with hypoglycaemia and other, but more often than not, the patients are able to monitor themselves. And if they identify any of the problems with their medications, they are able to know. They sometimes tell you when they take their medications they feel uneasy or they have dizziness, so I tell them teach them how to identify the type of medication causing it and if it likely to be the hypoglycaemic agents. They can stop that for a day, see the responds, and give me feedback about it. If they stop it and they are still feeling dizzy, then I will know it's either the other medications or it's another problem that is coming up with the condition.

**I: Despite the fact that patients are being educated on diabetes self-management, some patients are still not compliant. What do you think are the barriers to diabetes self-management education with respect to patients?**

R: Well, the problem we have is with their diets. Some patients have difficulty sticking to the diet. Some will come in and tell you they have been taking sugary drinks. They come in with high blood sugar and when you ask them what they ate the previous day, they will tell you they took Milo in the evening whiles they are aware Milo contains sugar. They will tell you that is what they enjoy taking and how they have been taking it for a long time and very difficult to stop. So it's usually with the diet. That's where we have difficulty with some of the patients. Some will tell you they feel uncomfortable eating the prescribed diet while others have to eat the food available in their homes because of financial constraints. If you instruct them not to eat Fufu and Fufu is the only food available in the house, it means they will have to take the Fufu or not eat at all.

**I: Finally, what would be the ideal way of delivering diabetes self-management education? Is it as a group, would you want to group individuals in one place and educate them or you would like to do it on a one to one basis.**

R: I prefer one on one.

**I: Thank you very much for your time.**

R: Thank you for having me.
