# Supplementary material for: Diabetes self-management education interventions and self-management in low-resource settings; a mixed methods study
Source: PLoS One. 2023 Jul 14;18(7):e0286974. doi: 10.1371/journal.pone.0286974 (PMC10348576; doi:10.1371/journal.pone.0286974)
Supplement: S6 File — (DOCX) [file pone.0286974.s008.docx]

Transcription on Diabetes Self-Management Education at facility xxx

**I: What do you know about diabetes self-management education?**

R: Diabetic patients can manage diabetes by taking their medication as prescribed on time. They should also eat healthy foods such as oats or wheat in the morning, waakye, rice and salad in the afternoon and exercise regularly at least 30 to 40minutes.

**I: Which group do you think should deliver the education to diabetic patients?**

R: I think doctors and patients living with diabetes for a period should deliver the education.

**I: How should the education be done? Should it be face to face or virtual (over the radio, TV or internet?)**

R: I would prefer the face-to-face education.

**I: Do you think the education should be done on a one on one basis or in a group? Which one would you prefer?**

R: I would prefer that the education should be done in a group.

**I: Where do you recommend the education should be done? Should it be done in the hospital, in the community or a hired place?**

R: I recommend the education should be done in the hospital because healthcare and treatment is accessible in the hospital.

**I: How often should the education be done? Should it be delivered at once or held at different time schedule?**

R: I think the education should be done on different time schedules because the number of diabetes patients who visit the hospital is huge, hence the education shouldn’t be done at once.

**I: How would you evaluate the education of diabetes in terms of performance in the facility xxx?**

R: I would say the hospital are doing their best because the doctors really educate the patients on how they can manage the diabetes themselves.

**I: In your opinion, what do you think are the barriers to behavioral change in patients despite the fact that they have been given diabetes self-management education?**

R: I think that in most of the cases, financial challenges and indiscipline on the part of the patients are barriers to their behavioral changes.

**I: What particular education should health professionals give to diabetic patients when they visit the hospital?**

R: I think doctors should particularly educate diabetic patients on how to take prescribed medication on time and the kind of foods to eat to stay healthy.

**I: Do you have any other thing you would want to say?**

R: No please

**I: Thank You for your time**

R: Thank You
