# Supplementary material for: Diabetes self-management education interventions and self-management in low-resource settings; a mixed methods study
Source: PLoS One. 2023 Jul 14;18(7):e0286974. doi: 10.1371/journal.pone.0286974 (PMC10348576; doi:10.1371/journal.pone.0286974)
Supplement: S7 File — (DOCX) [file pone.0286974.s009.docx]

**I: Good morning**

**Morning.** (All respondents).

**I: We want to discuss about how diabetic patients manage themselves.**

**I: What should patients living with diabetes do in order to manage themselves?**

R1: When you are diagnosed with diabetes it result in rise of sugar level so there is the need to make sure the sugar level reduces and this can be accomplished by checking the choice of meals to take also exercising every 30 minutes at least three times a week.

R3: I agree with R1 because we need to be cautious about the choice of meals and practice the habit of eating fruit. Exercising regular is also very important and needs to be done regular. In addition, the dietitians should also help us to be able to know the right food to be taking in order to be health.

R4: We should exercise regular by walking at least thirty minutes every hour and check, our diet by eating more of cocoyam leaves and enough salad.

R5: When you are diagnosed with diabetes, you should not be taking starchy food like fufu and banku. We should also include vegetables in our diet and take in a little bit of rice if we prefer because it contains starchy food.

R1: We should not also be eating late in the night because it may not be able to digest before we sleep.

**I: Who do think should deliver the education, should it be the doctor, nurses or those who are living with the diabetes.**

R4: When I was diagnosed with the disease, I was advised to attend to the hospital for medical care. The doctor then gave me some drugs and advised me on the type of food to eat and what to avoid.

**I: It means the doctor should be the one to deliver the education.**

**R4: Yes.**

R1: I think the doctor should deliver the education. However, those who have the experience and living with the disease should also help in the delivery because they have the experience.

R3: I will agree with R5 because most of our meals are starchy, hence we can add soya to it to be able to reduce the starch.

**I: I: How should the education be done? Should it be face-to-face or virtual (over the radio, TV or internet?)**

R3: I think face-to-face is better because most people don’t get the chance to listen to radio. It is also good because the patients get the chance to speak to the doctor directly and address his or her issues.

R1: I also think the face to face is appropriate.

**I: Do you all think the face to face is good?**

**(All respondent) Yes.**

**I: How do you want the education to be done? Do you prefer it one on one or in groups.**

R4: I prefer it to be in groups than face to face because some of the patients’ needs to learn from others in order to take care of themselves.

R1: I would have agreed with R4 but we are not in normal times. People can be affected with the Covid so I think the face to face with the doctor will be a good option.

**I: Where do you think this education should be delivered? Would you prefer the hospital, communities or we should hire a place? What is the ideal place for the education?**

R3: I think it should be done in the hospital because most of them will not be able, to attend if it is organized somewhere else but because he or she goes to the hospital for the drugs, he or she will receive the education.

R1: I suggest it will be held in the community to create awareness for those who have no idea about the disease.

**I: How do you want the education to be held? Do you prefer on in a day example six hours within the day, daily basis or be monthly.**

R1: I don’t have the strength or time to be coming everyday so I think once a month would be better.

**(All respondent) We prefer once in a month.**

**I: Don’t you prefer a day, were you would be taught everything outright.**

R3: Some of us are absent minded and may not be able to capture what is being taught when its done in a day so I think monthly will be ok because we can be able to learn something.

**I: What do you think hinder people for not adhering to the doctor’s advice despite the education delivered to them always?**

R1: I will talk about the issue of finance and use myself as an example, I don’t work, my husband is the only one that takes care of the house. If I don’t get financial support else where I cannot go according to the doctors advice. I have to buy my drugs and check the type of meals I eat. If am not financially stable, I can do it.

R4: Most of us don’t work and as a result we do enough thinking hence, we are unable to cater for ourselves.

R1: I think it’s based on personal choice not to adhere doctor doctors’ advice.

R3: lack of funds.

**I: How is the performance of this hospital with regard to education for diabetic patients?**

R1: I think their working very hard with regard to education because the doctor who attended to me when I was referred here assured me that I would be fine when I take my drugs seriously and also instructed me to adhere to the advices of the health professional.

R4: I support R1 because the doctors are very good here. The take good care of us, gives us emotional support and advices us in order to live a health life.

**I: What are the things that you expect to be taught during the education sessions?**

R3: I think we should be taught the importance of the medication so that we would be compelled to adhere to them. Also taking the drugs on time must also be a priority during the session.

R1: They should emphasize on the choice of meal we eat.

R4: They should also teach us to be cautious about eating very late in the night.

**I: Is there any question.**

R3: I want to know the reasons why we sometimes feel pains in our legs.

**I: The doctor will explain the reasons to you after we are done with the discussion.**

**I: We will end our discussion here, thank all very much.**
