# Supplementary material for: Diabetes self-management education interventions and self-management in low-resource settings; a mixed methods study
Source: PLoS One. 2023 Jul 14;18(7):e0286974. doi: 10.1371/journal.pone.0286974 (PMC10348576; doi:10.1371/journal.pone.0286974)
Supplement: S8 File — (DOCX) [file pone.0286974.s010.docx]

**I: This is the focus group discussion for facility yyy. These people have diabetes for more than 5years. I introduce to you R1, R2, R3, R4, R5, R6 and R7.**

**I: How many years have you had diabetes.**

R1: 17years.

R2: 34 years.

R3: 7years.

R4: 11 years.

R5: 14 years.

R6: 7 years.

R7: 24 years.

**I: Thank you all.**

**I: What are the education you know about diabetes and how are you going to apply as educate others?**

R4: What I know about diabetes is that, it is not curable when you contract it. I think we can manage it by checking our diet, practice regular taking of the drugs, visiting the hospital, regular exercising and preventing of late night eating.

**I: Thank you very much, is there more you have on education.**

R3: I think what R4 said is very true; we have to take our drugs regularly and visit the doctor as scheduled for us. Some people go for the drugs but do not take them. I know a woman who have also contracted the disease, I tried to advise her to be taking the drugs regularly but she refuses, one day she just feel down and died so I think we should take good care of ourselves by not taking some meals not good for our health.

R2: I think we should not overeat and when eating we should make sure the food is well balanced with vegetables. We should always take in fruits and after that take the drugs, prescribed for us.

R6: In the morning after taking care of yourself, we have to take the Dallin drug, thirty minutes time after eating we take the Medford and the other prescribed drugs. We should be cautious of taking in sugary food because when we don’t abide to that we end up not able to control ourselves when we want to urinate and hence soiling ourselves. Also in the evening, we should also take our drugs and adhere to what have being taught in the hospital to help us desist from getting complication.

R7: We should not overeat because we end up overburden ourselves when we do that. I didn’t know that until I encountered some complications where I had to lie down on cement floor for thirty minutes before I will be relieved, It help me a lot when I desisted from overeating. Also I think we should be aware of the time intervals we eat and make sure it’s a balanced diet.

R1: What my colleagues have said is very true but what I will add is that if we able to practice the habit of reading about the nature of the disease and what it entails that is very important.

R2: I will also talk about the time intervals of eating. It will be ok when by 8:30 we have already taking our breakfast and the prescribed drugs and 3:00 our supper so that the supper might not extend beyond 6:00 o’clock, which will bring about complication. In case you feel hungry due to sleeping very late, you can take in a little bit of porridge or oats.

R3: Diabetes occurs because of the failure of the system in our body to absorb the sugary substance in our diet so I think we should eat thirty minutes after taking the drugs. Let me set an example with myself, I have insulin in my body and if I decide not to eat thirty minutes after taking my drugs I will encounter problems because I have to eat so that the insulin will absorb the sugar out of my system. When the insulin does not function, that is when I can teste sugar in my urine, which causes problems.

**I: You have talked a lot about the quantity of food we need to take, the time intervals for eating, the prescribed drugs to take, exercising the body, and acquiring knowledge about the disease by reading but I want to know the minimum education we will all give to people who have diabetes.**

R1: I will educate him or her to be particular about the choice of food.

R2: They have to be educated about the causes of sugar in their urine and be cautious about their choice of food. They should also ensure they take their drugs frequently and be eating regularly to prevent complication.

R7: They should also practice the habit of keeping their drugs in their bag so that they can be able to take them when it needed.

R6: A diabetic patients should desist from drinking and smoking.

**I: Do you all accept that the answers provided above is the minimum education you will give to a diabetic patient.**

R7: We should also let them know what they will be exposing themselves into when they don’t abide by the doctor’s advice.

R2: I will also educate them to be cautious when they are either peeling vegetables or removing their fingernails because they might end up being cut by the blade or knife and when this happens, it may be difficult to heal because of the disease.

R6: When you are a diabetic patient and you discover that there is a cut on your legs, you have to immediately visit the hospital and seek medical treatment. I witnessed an instance of that sort when the person refused to go to the hospital when he had cuts on the leg and he later died of the complication 3 months’ time.

R2: in addition to what I said, a diabetic patient should always wipe the surface of his or her saw with Dettol anytime he or she experiences that before visiting the hospital.

I**: Thank you very much.**

**I: In educating diabetic patient, which form would you suggest, is it in the form of schooling or on daily base. Which form should it be organized?**

R2: Where I attend diabetic clinic, they normally take the first 30 minutes to teach us the dos and don’t we need to know about the disease before they start to attend to us.

I: You think teaching on daily basis is better than the form of schooling structure.

R1: I think we should adopt the integrated marketing communication where airtime on diabetic education will be purchased for advertisement on both the radio and television. Also a well-trained team should embark on education for people to really understand issues pertaining to the disease. The doctors and nurses must be trained on the disease.

R2: There should be mass testing in the clinic for people to check their sugar level and BP because most people have the disease and they are not aware of it. If they are aware of the disease, they will then seek medical care to prevent complication leading to death.

**I: Who do think should help in the delivering of diabetes self-management education. Do you prefer the nurses, doctor or someone who have experience with the disease?**

R3: Some people prefer to go to the churches rather than visiting the hospital.

R7: I agree with R1 because doctors and nurses can organize both the diabetic and non-diabetic patient to educate them on self-management so that they will be able to prevent complication.

R4: Nurses, doctors and someone who have the experience with the disease can deliver the education to others. To use myself as an example, I have taking it upon myself to educate my family members and sometimes assist outsiders who also need some information about the disease, so think they are all in the right position to deliver the education.

**I: In delivering diabetes self-management education, do you think it must be held within a day, in bits and in sessions and if so how many sessions would you prefer?**

R2: I think it should be held every day for 30minutes before they start attending to us when we come to the clinic.

R1: I think everything should be discussed at a daily session, and must not be in bits in order for the patient to get an understanding of what is being taught.

R5: I think we should get an educational material to read and understand the disease.

R1: I also think education should be done on daily basis because people might not be attending the session’s frequently and hence may not understand what is being taught.

**I: Do you think the education should be delivered in groups or one on one.**

R1: It should be organized in groups because the issue confidentiality and people feeling very shy because of the diseases is no more.

**I: Do you think it should be face-to-face or virtually organized.**

R1: I think with the virtual, most of the target group are uneducated so they cannot participate when using that channel.

**I: Do you think we should use all channels.**

R: Yes please (All respondent)

**I: Where would you think would be the best place to have this education on diabetes self-management. Is it the hospital or the community?**

R2: It can be done everywhere, example the hospital or the church.

**I: Where would be the place to organize it?**

R2: I think the church and the hospital will be the right place because in the hospital the doctor can organize them during their visit to the place.

R1: We have groups in church, which are also target groups. These groups have particular days of meeting so I think they can agree with them so that meeting can be held during that period.

R5: The church is a good place because in my church there times we organize rallies and sometimes invite lawyers and doctors to educate us, so I think that period is also best for the education.

R3: I also support the church place because it was through church service meetings where a doctor was invited to educate and perform checkups, and it was through that I was diagnosed of the disease.

I: **I: What do you think impedes behavioral change when it comes to diabetes self-management despite the fact that it have been taught several times?**

R2: I think it is the personal behaviors of the individuals. An example is a woman in my area who is diagnosed of the disease but saw her in drinking spot drinking alcoholic beverages so I stopped her and advised her to be taking the drugs.

R1: I think people have the notion that when they stop eating some meals that are not good for their health death still awaits them so they will decide to eat them despite their condition.

R2: To back what R1 said it very important not to take in substance that will cause complication in other to have a long live span. In my family, we were four siblings diagnosed of this disease and my twin brother just died of the disease because he was not complying with the advice of the doctors not to eat certain meals.

I: **What are other behavior that impedes diabetes self-management despite the fact that it have taught several times?**

R1: I think it is an attitude and minds set of the individual because I had a friend whose father was a medical doctor but he had the habit of drinking a lot and due to that, both of his legs were amputated because he was diagnosed of the disease but because of his attitude he could not stop drinking.

R4: I think most of them depend on effectiveness of their medicine and hence be eating all kinds of meals, which may affect them positively.

**I: Do you think people have important reasons that impede diabetes self-management despite the fact that it have taught several times?**

R2: No I think is their behavior and attitude.

**I: Do you have any impression about education delivered here in Facility yyy? What can you say is your experience on the education delivered here in Facility yyy?**

R2: It has really helped me a lot because I was at the Cardio center at korle bu and referred here. When I came here, I was sent to the diabetic center, during my visits, we had dietition coming every two weeks to educate us on our diet and the nurses teaches us on how to take care of ourselves. I have being able to learn about self-management through the education.

R4: They also gave us pamphlet freely to be reading on why to take care of themselves.

**I: Is there any more experience you have had about Facility yyy education on diabetes.**

R1: I was attending a private hospital and I once passed by, saw the signpost of diabetic center here so I decide to come here. When I came here, it was Dr Kusi who was educating us when we come in the morning. Moreover, the nurses come around to teach us but for about six month now, I think they have stopped doing that (R2: It’s due to the transfer of the dietitian but a woman was posted here recently). I also think the doctors should also explain to us the work of the medicine when we take them. In addition, I think the doctors should not allow other herbal medicine sellers to be around the diabetic center in the hospital selling their herbal drugs because some patients resort to those drugs and they will later get complication when they take them. We don’t also get our medicines when we come to the hospital and they are prescribed for us to buy them outside.

R5: I will advise that during our education the doctors should caution us on the herbal medicine because some of the herbalist tries to convince the patients on curing them when they take the medicine. I had an encounter with a herbalist trying to persuade me to buy the drug on notion of it curing the disease so I think it should be an issue of concern because the rural areas will be a target.

R3: In Facility yyy diabetic center, we don’t always get all our drugs when we come here, we sometimes get half of it and later buy the rest outside as compare to the zzz teaching hospital where you are giving all your drugs anytime you are scheduled for an appointment. This is an issue of concern and hence needs to be addressed because patients do not attend the hospital because of these reasons.

R1: I think we should be educated on the side effect of the drugs because some of the drugs affect the man by causing dysfunction of the male organ. When you visit facility xxx, they give drugs that will prevent the side effect. Some people end up having side effects because they don’t have the finances to purchase drugs that will be good for them, so I also suggest the NHIS should cover some the important drugs.

**I: Thank you all very much.**
