# Supplementary material for: Diabetes self-management education interventions and self-management in low-resource settings; a mixed methods study
Source: PLoS One. 2023 Jul 14;18(7):e0286974. doi: 10.1371/journal.pone.0286974 (PMC10348576; doi:10.1371/journal.pone.0286974)
Supplement: S9 File — (DOCX) [file pone.0286974.s011.docx]

**Facility yyy**

**I: Good afternoon.**

Nurse: Good afternoon

**I: We are at the Facility yyy. And we are here to interview one of the nurses involved in the management of patients living with diabetes. I want to find out what you know about diabetes self-management.**

R: it has to do with how the clients are able to manage themselves in terms of their diet, in terms of the amount of exercise they engage in, checking their blood sugar level, adhering to their regimen among others.

**I: Please, which healthcare professionals do you think should deliver diabetes self-management education?**

R: I think the nurses, doctors, pharmacist and nutritionist can all deliver.

**I: How do you think it should be done? Should it be done face to face or virtually? Virtually I mean by posters, messages and all that? How best can we have this education?**

R: I think the best way is doing it one on one.

**I: You mean Face to face.**

R: Yes face-to-face because some patients will not be able to read read but once you do it one on one or the face-to-face interaction, it helps.

**I: Do you think it should be delivered on a group basis or it should be done on a one on one basis?**

R: I think in both cases, it's good. Here for instance, when the person is diagnosed for the first time we do it one on one. We normally have the group sessions in the morning. However, we do it one on one, the person is able to express himself and ask questions because some are shy and wouldn’t be able to ask questions even when they do not understand in group settings, but can do so in a one on one setting.

**I: Where do you think this education should be done? Should it be done in the hospital settings? In the communities? Alternatively, maybe we should hire a place and educate the patients? Where do you think is ideal for such education to take place?**

R: The diabetic class?

**I: Yes the diabetes class**

R: Any of the places is good example the radio, the television stations, the hospital setting. They all help.

**I: When you come to Facility yyy hospital, how would you access the diabetes self-management here in this facility?**

R: I think we are doing our best. We are doing so much at the diabetic unit. They do refer some of the clients to us and we are sometimes called to the wards to give education to patients. So, yes Facility yyy hospital is doing so much and we even have clients coming from far places. Patients tell us they prefer this hospital when we try to transfer them to other facilities.

**I: What do you think can be done to make diabetes self-management education in Facility yyy better?**

R: I think when patients are waiting to be attended to in the waiting area, we can get some educative short videos in the local language, they can understand. I think it will help them relax as they wait. It will also educate them on some complication they will encounter if they fail to adhere to the advice of the professionals. In general, it will help educate them on diabetes. So these are some of the things I think we can do to help.

**I: What do you think are some of the barriers to behavioural change on the side of the patients? Despite the fact that they have been given diabetes self-management education, what do you think hinders some of the patients from adhering to this education?**

R: I think support from the family and friends will go a long way to help them. If relatives and friends stop looking at diabetic patients to be ‘’different’’ because of the new way of life that comes with the condition but rather accept, encourage, support and help these patients to adhere to doctor’s advice, it will really help these patients a lot. Also if patients get to understand the condition and the financial burden it places on family, I think it will help change their behaviour. Change is difficult, and for some patients switching from old habits to new ones is not easy for them. But if relatives can help, I think gradually it will be better.

**I: Is there any other thing that you think hinders patients from adhering?**

R: For some it has to do with money. Some can't afford their medication. Over here, I don't believe any of the medication is free. However, I know that even if it is covered by insurance, they still pay a little. Some don't even have money to attend to the clinic in the first place for their medications. So these are some of the challenges. Some are willing and are ready to adhere to treatment but they just can’t afford it.

**I: All right. Thank you very much for your time.**
