# Supplementary material for: Diabetes self-management education interventions and self-management in low-resource settings; a mixed methods study
Source: PLoS One. 2023 Jul 14;18(7):e0286974. doi: 10.1371/journal.pone.0286974 (PMC10348576; doi:10.1371/journal.pone.0286974)
Supplement: S10 File — (DOCX) [file pone.0286974.s012.docx]

Transcription on Diabetes Self-Management Education at facility yyy

**I: Please I want to find out what you know about Diabetes Self-Management Education**.

R: Diabetes Self-Management Education talks about educating the patients on how to plan their diet or meal, exercising regularly, taking their medication as prescribed as well as checking their FBS and RBS at regular intervals.

**I: Which health care professionals do you think should deliver the Diabetes Self Management Education**?

R: Doctors, Nurses, Pharmacists, and even Lab technicians

**I: How do you think the education should be? Should it be face-to-face or virtual?**

R: It can be face to face because that is more interactive.

**I: Do you think diabetes self-management education** **should be delivered in a group by gathering a group of patients living with diabetes or you think it should be done on a one to one basis?**

R: Depending on the number of patients we have, it can be done either one to one or in a group. If the number is huge, they can be grouped throughout the education. However, when it comes to normal counseling session, one to one is more appropriate.

**I: Where do you think this education should be done? Should it be done in the clinics, in the homes or in the communities? Where exactly do you think is the ideal place to have the diabetes education?**

R: In my point of view, it can be done in the clinic or in the community where people without fair knowledge about it can get to know more about the diabetes and how to manage the it themselves.

**I: So how would you assess diabetes self-management education in terms of performance in your hospital, facility yyy?**

R: I would say we are doing our best in educating our clients. We do one to one counseling and group sessions here when the patients come on their appointment dates. We group them and talk to them about how they can manage the diabetes themselves. When cases are also referred to us, we talk to the patients’ one on one on how to manage it themselves. Therefore, with the assessment I would say we are doing our best.

**How can diabetes self-management be made better or improved in your hospital?**

R: We can group the patients so that each person would be in a group when they are booked for an appointment, this will help them to be familiar with each other.

**I: In your opinion, what do you think are the barriers to behavioral change in patients despite the fact that they have been given diabetes self-management education?**

R: In most cases, finance and non-support from the family are barriers to consider. Most of the patients claim they don’t have anyone to support them with money or care for them hence they keep recording high sugar levels

**I: Thank You very much for your time**

R: Thank you too.
