# Supplementary material for: Diabetes self-management education interventions and self-management in low-resource settings; a mixed methods study
Source: PLoS One. 2023 Jul 14;18(7):e0286974. doi: 10.1371/journal.pone.0286974 (PMC10348576; doi:10.1371/journal.pone.0286974)
Supplement: S11 File — (DOCX) [file pone.0286974.s013.docx]

**I: What education do you think should be given to diabetic patients? On the other hand, what are some of the things we need to do to manage diabetes in patients?**

R3: Diabetics is a deadly disease so the patients need to take their medications on time. They also need to know the kind of food to eat and the time to eat. We have been given a leaflet that shows the type of foods we need to eat. When we are able to do these things, we would be healthy. We are also supposed to regularly check our sugar levels to know whether we need more or less sugar in our body. We should also have enough medication to treat the diabetics anytime we have high or low sugar levels. We need to take in more soupy foods to stay healthy. Most importantly, we need to abide by all the directives we are being given to manage the diabetes.

R5: I always visit Akawey hospital for treatment since I had diabetes for the past six years. I usually take medication to reduce my blood pressure. I had complications and the doctor realized the medication wasn’t helpful. My doctor put me on insulin to maintain my sugar and blood levels. He recommended regular exercise and advised that I take my meals and medication on time and avoid oily foods. I ate banku during my complications at the hospital and I was able to reduce my sugar levels because I tried to regulate the food intake.

**I: What was your sugar level, after taking the small amount of banku?**

R5: My sugar level was initially 8.1 but after eating the small amount banku, my sugar level was 21.2.

**I: Would you agree that we need to educate diabetic patients about the kind of food they need to eat and the time to eat such foods?**

R5: Yes please. They also need to be taught how to exercise regularly.

**I: What other education should be given to diabetic patients apart from the ones that have been stated already?**

R4: We need to be discipline and follow all the educations on how to manage diabetes ourselves as we are being taught about them

**I: How would you advise a new diabetic patient?**

R4: I would advise the patient to be discipline and adhere to all the self-management education on diabetes.

**I: How do you think we can educate patients about managing the diabetes themselves? Do you think the education should be done through books? Or by health professionals? What method would you prefer?**

R4: I prefer the education through books because we have been given a book that teaches us how to manage the diabetes. The book is normally read out to me and I try to assess whether I have been able to follow the education in the book.

**I: To arrange on the method or way of education to diabetic patients? Which methods would you prefer?**

R3: I think we need to consider the kind of medication to take and the time to be taking those. It could be before or after meals. I prefer all the teachings in a leaflet form and given to us to read. For those who can’t read the leaflet personally, they can allow our children or friends to help them read

**I: Should the education be done on a face-to-face basis or virtual (over the internet). Which one would be helpful or preferable?**

R1: The virtual education is easy and helpful, however older people would not be able to use internet for the education. Therefore, I think the education should be done on face-to-face basis as patients are grouped to visit the hospital on a specific date or day.

R6: I would also prefer face-to-face education.

**I: How long do you want the education to last when diabetic patients are grouped to visit the hospital on specific days?**

R2: It should last for at least 30mins.

I: **Do you prefer to have the education for at least 30mins or you prefer to visit the hospital a couple of times, let’s say 5minutes or 10minutes on a regular basis for the education?**

R4: I prefer diabetic patients would be grouped to visit the hospital at least on specified days or dates. For example, they can be grouped so that some would come on Mondays, others on Tuesdays or any other days.

R6: I also prefer the grouping of the diabetic patients to come to the hospital on specific days.

I: **Where do you think the education should be done? Should it be done in the hospital or in the community or a hired place? Which place would be appropriate for the education?**

R: I think the education should be done in the hospital.

**I: What do you think are the barriers to behavioral changes in patients despite the fact that they have been educated on how to manage the diabetes themselves?**

R1: I think it is the attitude and indiscipline behavior of diabetic patients.

R4: I also think it is the mindset and complacency of the patients

R3: I think when we are indiscipline in taking meals and medication.

R6: I also think discouragement from people act as barrier to our behavioral change

**I: Apart from what you have already said, do you still think there are genuine reasons or barriers to the behavioral changes in diabetic patients?**

R6: I think we need to be compliant and follow all the education.

**I: Has the Covid-19 affected you? If yes, how has it affected you?**

R6: I have been able to take care of myself during the Covid-19 pandemic. It hasn’t really affected me.

R4: I experienced severe pains in my knee.

R3: We needed to control our drinking of alcohol habits.

**I: Do you think financial challenge is one of the barriers to behavioral changes in diabetic patients or the inability of patients to follow the education?**

R4: I face some financial challenges but I have learnt how to manage the little money I have to buy any suitable meal.

R1: I don’t think finances is a problem because I have health insurance so I can afford to pay the little money required for treatment,

R3: I have financial challenges in getting some of my medications.

I: **How would you assess the Akawey Hospital’s performance in relations to the education and treatment given to diabetic patients who visit the hospital? Would you say it is beneficial?**

R1: The education from the hospital has been helpful and best so far and the doctor encourages us to follow the instructions.

R6: I think the hospital has been helpful. For the first time, you would be scared to see the doctor especially when you are indiscipline but with time, the doctors would educate you on how to self-manage the diabetes

R4: The doctor encourages us to take our medication on time.

**I: Which group do you think should deliver the education on diabetes? Is it the health professionals or diabetics patients?**

R4: I believe our doctors

R3: I recommend nurses and persons who have suffered diabetics for a long time.

**I: Thank you very much**
