# Supplementary material for: Diabetes self-management education interventions and self-management in low-resource settings; a mixed methods study
Source: PLoS One. 2023 Jul 14;18(7):e0286974. doi: 10.1371/journal.pone.0286974 (PMC10348576; doi:10.1371/journal.pone.0286974)
Supplement: S12 File — (DOCX) [file pone.0286974.s014.docx]

**FACILITY XXX/FDG/PLD LESS THAN 1 YEAR**

**FGD FACILITY XXX Patients with DM for LESS THAN 1 YR**

**I: Good morning.**

**Morning.** (All respondents).

**I: We want to discuss about how diabetic patients manage themselves.**

**I: What should patients living with diabetes do in order to manage themselves?**

R1: When you are diagnosed with diabetes it result in rise of sugar level so there is the need to make sure the sugar level reduces and this can be accomplished by checking the choice of meals to take also exercising every 30 minutes at least three times a week.

R3: I agree with R1 because we need to be cautious about the choice of meals and practice the habit of eating fruit. Exercising regular is also very important and needs to be done regular. In addition, the dietitians should also help us to be able to know the right food to be taking in order to be health.

R4: We should exercise regular by walking at least thirty minutes every hour and check, our diet by eating more of cocoyam leafs and enough salad.

R5: When you are diagnosed with diabetes, you should not be taking starchy food like fufu and banku. We should also include vegetables in our diet and take in a little bit of rice if we prefer because it contains starchy food.

R1: We should not also be eating late in the night because it may not be able to digest before we sleep.

**I: Who do think should deliver the education, should it be the doctor, nurses or those who are living with the diabetes.**

R4: When I was diagnosed with the disease, I was advised to attend to the hospital for medical care. The doctor then gave me some drugs and advised me on the type of food to eat and what to avoid.

**I: It means the doctor should be the one to deliver the education.**

**R4: Yes.**

R1: I think the doctor should deliver the education. However, those who have the experience and living with the disease should also help in the delivery because If we are able to get information from them, it will be good because I would not have experienced what they have being though so we can get more information from them to take care of ourselves.

R3: I will agree with R5 because most of our meals are starchy, hence we can add soya to it to be able to reduce the starch.

**I: I: How should the education be done? Should it be face-to-face or virtual (over the radio, TV or internet?)**

R3: I think face-to-face is better because most people don’t get the chance to listen to radio. It is also good because the patients get the chance to speak to the doctor directly and address his or her issues and the doctor direct him or her on what to do.

R1: I also think the face to face is appropriate.

**I: Do you all think the face to face is good?**

**(All respondent) Yes.**

**I: How do you want the education to be done? Do you prefer it one on one or in groups.**

R4: I prefer it to be in groups than face to face because some of the patients’ needs to learn from others in order to take care of themselves. Some have the diabetes for more than 5years but dont know how to take care of themselves.

R1: I would have agreed with R4 but we are not in normal times People can be affected with the Covid when they come into contact with affected persons so I think the face to face with the doctor will not be a good option and you ca get any information from the doctor.

**I: Where do you think this education should be delivered? Would you prefer the hospital, communities or we should hire a place? What is the ideal place for the education?**

R3: I think it should be done in the hospital because most of them will not be able, to attend if it is organized somewhere else but because he or she goes to the hospital for the drugs, he or she will receive the education.

R1: I suggest it will be held in the community to create awareness for those who have no idea about the disease, how it occurs and brought about.

**I: How do you want the education to be held? Do you prefer on in a day example six hours within the day, daily basis or be monthly.**

R1: I don’t have the strength or time to be coming everyday so I think once a month would be better.

**(All respondent) We prefer once in a month.**

**I: Don’t you prefer a day, were you would be taught everything outright.**

R3: Some of us are absent minded and may not be able to capture what is being taught when its done in a day so I think monthly will be ok because we can be able to learn something.

**I: What do you think hinder people for not adhering to the doctor’s advice despite the education delivered to them always?**

R1: I will talk about the issue of finance and use myself as an example, I don’t work, my husband is the only one that takes care of the house. If I don’t get financial support else where I cannot go according to the doctors advice. I have to buy my drugs and check the type of meals I eat. If am not financially stable, I can do it.

R4: Most of us don’t work and as a result we do enough thinking hence, we are unable to cater for ourselves.

R1: I think it’s based on personal choice not to adhere doctor doctors’ advice.

R3: lack of funds.

**I: How is the performance of this hospital with regard to education for diabetic patients?**

R1: I think their working very hard with regard to education because the doctor who attended to me when I was referred here assured me that I would be fine when I take my drugs seriously and also instructed me to adhere to the advices of the health professional.

R4: I support R1 because the doctors are very good here. The take good care of us, gives us emotional support and advices us in order to live a health life.

**I: What are the things that you expect to be taught during the education sessions?**

R3: I think we should be taught the importance of the medication so that we would be compelled to adhere to them. Also taking the drugs on time must also be a priority during the session.

R1: They should emphasize on the choice of meal we eat.

R4: They should also teach us to be cautious about eating very late in the night.

**I: Is there any question.**

R3: I want to know the reasons why we sometimes feel pains in our legs.

**I: The doctor will explain the reasons to you after we are done with the discussion.**

**I: We will end our discussion here, thank all very much.**

**FGD-DMSE RESEARCH -FGD patients living with dm for OVER 5 YRS -KBTH site**

**I: This study is to find ways by which people living with diabetes can be able to take care of themselves. I am Dr Lamptey.**

**I: We want to know the things people living with diabetes can do to take care of themselves.**

R3: People living with diabetes should be cautious about the choice meals.

R5: I also think the time we take our meals is also very important. We should give intervals between our breakfast, lunch and supper.

R2: I will add to what R5 said about the time, we should be cautious about the time intervals we take our medications and reduce the intake of carbohydrate specifically kenkey and protein because it takes a longer period for it to digest. We should rather increase the intake of vegetables.

R4: Concerning the choice of meals, I think we can eat six times a day in a sense that we can divide every food we will be eating within the day twice so we eat them in bits. When we eat at 10: 00, we ca then take the rest at 12: 00.

R3: W should not be eating at any time; we must set intervals between our meals. Example I have regular times I take my meals within this 30 years period living with diabetes and am 85 years now which means practicing that have helped me to avoid complications.

**I: R3 Please talk about the intake of the kenkey.**

R3: I was born into a family that prepares kenkey almost every day and I was eating kenkey always but since I had diabetes for 2 years now , I have stopped eating kenkey because it takes time to digest. Those who will like to eat kenkey should take a little.

R2: People living with diabetes can take in kenkey because it contains fiber that enhances digestion. They should take a little bit of it because taking in so much will also cause complication. Living with diabetes does not mean we can’t eat every meal; it depends on the quantity we take in.

R5: I take in kenkey and fufu but I do that early before sunset so that by the time I want to sleep and feels like eating I would take in some fruits. We also need to exercise regularly which will also help us to manage the diabetes.

R1: In the morning when I wake up I take in many vegetables, I later take in oats and oranges. In the afternoon around 12 o’clock, I take some heavy food and in the evening, around 4 o’clock I take in some plantain.

R5: I will agree with R1 but for me when I take in heavy food before I sleep I realize that my sugar level rise upon waking up the morning. What I do is that I eat vegetables before I sleep.

R3: I dont eat after 5 o’clock in the evening and what I do is, in the morning I take my breakfast with a lot **“Dandelion**” vegetables because I have planted it in my house.

**I: Apart from the regular exercise and making the right choices with the kind of meals to eat, what other things should diabetic patients do in other to manage his or herself?**

R3: I think our sugar rises because of thinking a lot. Diabetes also brings about high blood pressure and hypertension so we should not be thinking a lot.

**I: What are the factors that causes behavioral change within diabetic patients despite the doctors teaching them on the dos and dont?**

R3: It is because of lack of finances. We cannot eat the kind of food you suppose to eat if you dont get regular sources of finance. We can’t even buy our medication regular if we dont get regular flow of financing, this makes us disobey the doctors advice.

R4: I think self-discipline is also a factor because if we dont discipline ourselves to obey the doctors advice we end up doing otherwise. Another factor is the kind of work we do because some of us we dont have time to even eat in the evening so we end up eating late in the evening.

R3: I think it is depends on the kind of lifestyle we live because we might be taking in more protein and drinks when we were not diagnosed of diabetes hence we find it difficult to obey doctors advice to avoid them when we are living with the disease.

R2: I also think it’s due to the ineffectiveness of the drugs prescribed by the doctors. We sometimes take drugs for a longer period and we dont see the effectiveness of the drug hence we decided not obey the doctors next time he or she instruct us.

R4: I used to inject the insulin in the house but anytime I inject it, my sugar level rises so a doctor friend of mine advised me that the insulin should be injected in the hospital and by a doctor so for 5years now I have stop using the insulin.

R3: We should also eat within the correct intervals, which will help us, a lot. I always experience hypo (sugar level becomes very low) in the evening and will be shivering when I dont eat well during the day. When this happens, I have to get some soft drink and will be ok.

R5: I have a friend who was also diagnosed with diabetes but the doctor have now taking him off his drugs because he was very conscious about self-management. He always eats the right meal and exercise regular, so I think when we obey the doctor’s advice and do as they say we will also be taking off the drugs because some of the original drugs are very expensive.

R2: Lifestyle also causes behavioral change because we sometimes forget to take our drugs during work hours because we might not have eating within these periods. We then decide to eat heavily in the evening to enable us take our drugs but we should be concern about the digestion of the food. We should wait for at least 30 mins before we sleep or in the evening, we can choose to take in lighter food such as cream crackers and some tea, which will help us.

**I: Which form do you prefer the diabetes self-management education to be, do you want it one on one or in groups.**

R3: I prefer the one on one.

R1: I also prefer the one on one

R2: One on one will be ok for me.

R7: I will go for all the options depending on the doctor’s preference.

R5: I would also prefer one on one but will also talk about the pamphlet giving to us. It sometimes contain foreign information which is their food and what they need to do in order to take care of themselves so think they should be limited to our local activities. In addition, visuals displayed to us should be translated into pamphlet and frequently distributed to us so that we can be reading at home.

R4: I would also prefer the one on one.

**I: What should be the duration of the education? Do you prefer every month, half a year, daily or hours?**

R3: I would prefer an hour.

**Majority of R**: We will prefer it within shorter period (in hours).

**I: Do you prefer we teach all the topics under diabetes self-management one day or we should take the entire topic in bits. Example choice of meals, exercise and the drugs.**

R2: I want it to be ones.

R4: I also think we should take it one at a time so we will be able to understand and considering the time factor we cannot take all at ones.

**I: Which group do you prefer should embark on this education? Do you prefer the doctor, nurses or people living with diabetes?**

R3: I think the doctors are capable to embark on the education.

R6: I also prefer the doctors.

R4: Doctors have their responsibilities so I would prefer the dietitians to take us through the choice of meals we should take as diabetes patients.

R1: I think the dietitians always demand money in order to attend to us so its always difficult see them.

**I: In your opinion, how would you assess the diabetes self-management education delivered to you infacility xxx?**

R3: They are very good.

R1: They are very good because they take their time to educate us well on how to manage ourselves.

R5: I used to becoming for diabetes clinic at the main hospital but I stopped because I have to be waking up early in the morning around 2:00 to form a queue and leave around 11: 00 and it had effect on my work.

R2: It is not encouraging because you have wait for longer periods before you will be able to see the doctor. This is due to the results from the lab that takes longer periods.

**I: I thank you all for the contribution made towards education on diabetes self-management but I will also accept advice from you that will also help us in this education.**

R3: I think this all tailored to the choice of meals, regular exercise and taking in many vegetables in order to self-manage ourselves.

**I: Thank you all very much.**

**FGD-Patients on Insulin-weija site**

**I: What education do you think should be given to diabetic patients? On the other hand, what are some of the things we need to do to manage diabetes in patients?**

R3: Diabetics is a deadly disease so the patients need to take their medications on time. They also need to know the kind of food to eat and the time to eat. We have been given a leaflet that shows the type of foods we need to eat. When we are able to do these things, we would be healthy. We are also supposed to regularly check our sugar levels to know whether we need more or less sugar in our body. We should also have enough medication to treat the diabetics anytime we have high or low sugar levels. We need to take in more soupy foods to stay healthy. Most importantly, we need to abide by all the directives we are being given to manage the diabetes.

R5: I always visit Akawey hospital for treatment since I had diabetes for the past six years. I usually take medication to reduce my blood pressure. I had complications and the doctor realized the medication wasn’t helpful. My doctor put me on insulin to maintain my sugar and blood levels. He recommended regular exercise and advised that I take my meals and medication on time and avoid oily foods. I ate banku during my complications at the hospital and I was able to reduce my sugar levels because I tried to regulate the food intake.

**I: What was your sugar level, after taking the small amount of banku?**

R5: My sugar level was initially 8.1 but after eating the small amount banku, my sugar level was 21.2.

**I: Would you agree that we need to educate diabetic patients about the kind of food they need to eat and the time to eat such foods?**

R5: Yes please. They also need to be taught how to exercise regularly.

**I: What other education should be given to diabetic patients apart from the ones that have been stated already?**

R4: We need to be discipline and follow all the educations on how to manage diabetes ourselves as we are being taught about them

**I: How would you advise a new diabetic patient?**

R4: I would advise the patient to be discipline and adhere to all the self-management education on diabetes.

**I: How do you think we can educate patients about managing the diabetes themselves? Do you think the education should be done through books? Or by health professionals? What method would you prefer?**

R4: I prefer the education through books because we have been given a book that teaches us how to manage the diabetes. The book is normally read out to me and I try to assess whether I have been able to follow the education in the book.

**I: To arrange on the method or way of education to diabetic patients? Which methods would you prefer?**

R3: I think we need to consider the kind of medication to take and the time to be taking those. It could be before or after meals. I prefer all the teachings in a leaflet form and given to us to read. For those who can’t read the leaflet personally, they can allow our children or friends to help them read

**I: Should the education be done on a face-to-face basis or virtual (over the internet). Which one would be helpful or preferable?**

R1: The virtual education is easy and helpful, however older people would not be able to use internet for the education. Therefore, I think the education should be done on face-to-face basis as patients are grouped to visit the hospital on a specific date or day.

R6: I would also prefer face-to-face education.

**I: How long do you want the education to last when diabetic patients are grouped to visit the hospital on specific days?**

R2: It should last for at least 30mins.

I: **Do you prefer to have the education for at least 30mins or you prefer to visit the hospital a couple of times, let’s say 5minutes or 10minutes on a regular basis for the education?**

R4: I prefer diabetic patients would be grouped to visit the hospital at least on specified days or dates. For example, they can be grouped so that some would come on Mondays, others on Tuesdays or any other days.

R6: I also prefer the grouping of the diabetic patients to come to the hospital on specific days.

I: **Where do you think the education should be done? Should it be done in the hospital or in the community or a hired place? Which place would be appropriate for the education?**

R: I think the education should be done in the hospital.

**I: What do you think are the barriers to behavioral changes in patients despite the fact that they have been educated on how to manage the diabetes themselves?**

R1: I think it is the attitude and indiscipline behavior of diabetic patients.

R4: I also think it is the mindset and complacency of the patients

R3: I think when we are indiscipline in taking meals and medication.

R6: I also think discouragement from people act as barrier to our behavioral change

**I: Apart from what you have already said, do you still think there are genuine reasons or barriers to the behavioral changes in diabetic patients?**

R6: I think we need to be compliant and follow all the education.

**I: Has the Covid-19 affected you? If yes, how has it affected you?**

R6: I have been able to take care of myself during the Covid-19 pandemic. It hasn’t really affected me.

R4: I experienced severe pains in my knee.

R3: We needed to control our drinking of alcohol habits.

**I: Do you think financial challenge is one of the barriers to behavioral changes in diabetic patients or the inability of patients to follow the education?**

R4: I face some financial challenges but I have learnt how to manage the little money I have to buy any suitable meal.

R1: I don’t think finances is a problem because I have health insurance so I can afford to pay the little money required for treatment,

R3: I have financial challenges in getting some of my medications.

I: **How would you assess the Akawey Hospital’s performance in relations to the education and treatment given to diabetic patients who visit the hospital? Would you say it is beneficial?**

R1: The education from the hospital has been helpful and best so far and the doctor encourages us to follow the instructions.

R6: I think the hospital has been helpful. For the first time, you would be scared to see the doctor especially when you are indiscipline but with time, the doctors would educate you on how to self-manage the diabetes

R4: The doctor encourages us to take our medication on time.

**I: Which group do you think should deliver the education on diabetes? Is it the health professionals or diabetics patients?**

R4: I believe our doctors

R3: I recommend nurses and persons who have suffered diabetics for a long time.

**I: Thank you very much**

**FGD PATIENT WITH DM FOR MORE THAN 5 YEARS Weija**

**I: This is the focus group discussion for weija gbawe municipal hospital. These people have diabetes for more than 5years. I introduce to you R1, R2, R3, R4, R5, R6 and R7.**

**I: How many years have you had diabetes.**

R1: 17years.

R2: 34 years.

R3: 7years.

R4: 11 years.

R5: 14 years.

R6: 7 years.

R7: 24 years.

**I: Thank you all.**

**I: What are the education you know about diabetes and how are you going to apply as educate others?**

R4: What I know about diabetes is that, it is not curable when you contract it. I think we can manage it by checking our diet, practice regular taking of the drugs, visiting the hospital, regular exercising and preventing of late night eating.

**I: Thank you very much, is there more you have on education.**

R3: I think what R4 said is very true; we have to take our drugs regularly and visit the doctor as scheduled for us. Some people go for the drugs but do not take them. I know a woman who have also contracted the disease, I tried to advise her to be taking the drugs regularly but she refuses, one day she just feel down and died so I think we should take good care of ourselves by not taking some meals not good for our health.

R2: I think we should not overeat and when eating we should make sure the food is well balanced with vegetables. We should always take in fruits and after that take the drugs, prescribed for us.

R6: In the morning after taking care of yourself, we have to take the Dallin drug, thirty minutes time after eating we take the Medford and the other prescribed drugs. We should be cautious of taking in sugary food because when we don’t abide to that we end up not able to control ourselves when we want to urinate and hence soiling ourselves. Also in the evening, we should also take our drugs and adhere to what have being taught in the hospital to help us desist from getting complication.

R7: We should not overeat because we end up overburden ourselves when we do that. I didn’t know that until I encountered some complications where I had to lie down on cement floor for thirty minutes before I will be relieved, It help me a lot when I desisted from overeating. Also I think we should be aware of the time intervals we eat and make sure it’s a balanced diet.

R1: What my colleagues have said is very true but what I will add is that if we able to practice the habit of reading about the nature of the disease and what it entails that is very important.

R2: I will also talk about the time intervals of eating. It will be ok when by 8:30 we have already taking our breakfast and the prescribed drugs and 3:00 our supper so that the supper might not extend beyond 6:00 o’clock, which will bring about complication. In case you feel hungry due to sleeping very late, you can take in a little bit of porridge or oats.

R3: Diabetes occurs because of the failure of the system in our body to absorb the sugary substance in our diet so I think we should eat thirty minutes after taking the drugs. Let me set an example with myself, I have insulin in my body and if I decide not to eat thirty minutes after taking my drugs I will encounter problems because I have to eat so that the insulin will absorb the sugar out of my system. When the insulin does not function, that is when I can teste sugar in my urine, which causes problems.

**I: You have talked a lot about the quantity of food we need to take, the time intervals for eating, the prescribed drugs to take, exercising the body, and acquiring knowledge about the disease by reading but I want to know the minimum education we will all give to people who have diabetes.**

R1: I will educate him or her to be particular about the choice of food.

R2: They have to be educated about the causes of sugar in their urine and be cautious about their choice of food. They should also ensure they take their drugs frequently and be eating regularly to prevent complication.

R7: They should also practice the habit of keeping their drugs in their bag so that they can be able to take them when it needed.

R6: A diabetic patients should desist from drinking and smoking.

**I: Do you all accept that the answers provided above is the minimum education you will give to a diabetic patient.**

R7: We should also let them know what they will be exposing themselves into when they don’t abide by the doctor’s advice.

R2: I will also educate them to be cautious when they are either peeling vegetables or removing their fingernails because they might end up being cut by the blade or knife and when this happens, it may be difficult to heal because of the disease.

R6: When you are a diabetic patient and you discover that there is a cut on your legs, you have to immediately visit the hospital and seek medical treatment. I witnessed an instance of that sort when the person refused to go to the hospital when he had cuts on the leg and he later died of the complication 3 months’ time.

R2: in addition to what I said, a diabetic patient should always wipe the surface of his or her saw with Dettol anytime he or she experiences that before visiting the hospital.

I**: Thank you very much.**

**I: In educating diabetic patient, which form would you suggest, is it in the form of schooling or on daily base. Which form should it be organized?**

R2: Where I attend diabetic clinic, they normally take the first 30 minutes to teach us the dos and don’t we need to know about the disease before they start to attend to us.

I: You think teaching on daily basis is better than the form of schooling structure.

R1: I think we should adopt the integrated marketing communication where airtime on diabetic education will be purchased for advertisement on both the radio and television. Also a well-trained team should embark on education for people to really understand issues pertaining to the disease. The doctors and nurses must be trained on the disease.

R2: There should be mass testing in the clinic for people to check their sugar level and BP because most people have the disease and they are not aware of it. If they are aware of the disease, they will then seek medical care to prevent complication leading to death.

**I: Who do think should help in the delivering of diabetes self-management education. Do you prefer the nurses, doctor or someone who have experience with the disease?**

R3: Some people prefer to go to the churches rather than visiting the hospital.

R7: I agree with R1 because doctors and nurses can organize both the diabetic and non-diabetic patient to educate them on self-management so that they will be able to prevent complication.

R4: Nurses, doctors and someone who have the experience with the disease can deliver the education to others. To use myself as an example, I have taking it upon myself to educate my family members and sometimes assist outsiders who also need some information about the disease, so think they are all in the right position to deliver the education.

**I: In delivering diabetes self-management education, do you think it must be held within a day, in bits and in sessions and if so how many sessions would you prefer?**

R2: I think it should be held every day for 30minutes before they start attending to us when we come to the clinic.

R1: I think everything should be discussed at a daily session, and must not be in bits in order for the patient to get an understanding of what is being taught.

R5: I think we should get an educational material to read and understand the disease.

R1: I also think education should be done on daily basis because people might not be attending the session’s frequently and hence may not understand what is being taught.

**I: Do you think the education should be delivered in groups or one on one.**

R1: It should be organized in groups because the issue confidentiality and people feeling very shy because of the diseases is no more.

**I: Do you think it should be face-to-face or virtually organized.**

R1: I think with the virtual, most of the target group are uneducated so they cannot participate when using that channel.

**I: Do you think we should use all channels.**

R: Yes please (All respondent)

**I: Where would you think would be the best place to have this education on diabetes self-management. Is it the hospital or the community?**

R2: It can be done everywhere, example the hospital or the church.

**I: Where would be the place to organize it?**

R2: I think the church and the hospital will be the right place because in the hospital the doctor can organize them during their visit to the place.

R1: We have groups in church, which are also target groups. These groups have particular days of meeting so I think they can agree with them so that meeting can be held during that period.

R5: The church is a good place because in my church there times we organize rallies and sometimes invite lawyers and doctors to educate us, so I think that period is also best for the education.

R3: I also support the church place because it was through church service meetings where a doctor was invited to educate and perform checkups, and it was through that I was diagnosed of the disease.

I: **I: What do you think impedes behavioral change when it comes to diabetes self-management despite the fact that it have been taught several times?**

R2: I think it is the personal behaviors of the individuals. An example is a woman in my area who is diagnosed of the disease but saw her in drinking spot drinking alcoholic beverages so I stopped her and advised her to be taking the drugs.

R1: I think people have the notion that when they stop eating some meals that are not good for their health death still awaits them so they will decide to eat them despite their condition.

R2: To back what R1 said it very important not to take in substance that will cause complication in other to have a long live span. In my family, we were four siblings diagnosed of this disease and my twin brother just died of the disease because he was not complying with the advice of the doctors not to eat certain meals.

I: **What are other behavior that impedes diabetes self-management despite the fact that it have taught several times?**

R1: I think it is an attitude and minds set of the individual because I had a friend whose father was a medical doctor but he had the habit of drinking a lot and due to that, both of his legs were amputated because he was diagnosed of the disease but because of his attitude he could not stop drinking.

R4: I think most of them depend on effectiveness of their medicine and hence be eating all kinds of meals, which may affect them positively.

**I: Do you think people have important reasons that impede diabetes self-management despite the fact that it have taught several times?**

R2: No I think is their behavior and attitude.

**I: Do you have any impression about education delivered here in Akawey? What can you say is your experience on the education delivered here in Akawey?**

R2: It has really helped me a lot because I was at the Cardio center at facility xxx and referred here. When I came here, I was sent to the diabetic center, during my visits, we had dietition coming every two weeks to educate us on our diet and the nurses teaches us on how to take care of ourselves. I have being able to learn about self-management through the education.

R4: They also gave us pamphlet freely to be reading on why to take care of themselves.

**I: Is there any more experience you have had about Akawey education on diabetes.**

R1: I was attending a private hospital and I once passed by, saw the signpost of diabetic center here so I decide to come here. When I came here, it was Dr x123x who was educating us when we come in the morning. Moreover, the nurses come around to teach us but for about six month now, I think they have stopped doing that (R2: It’s due to the transfer of the dietitian but a woman was posted here recently). I also think the doctors should also explain to us the work of the medicine when we take them. In addition, I think the doctors should not allow other herbal medicine sellers to be around the diabetic center in the hospital selling their herbal drugs because some patients resort to those drugs and they will later get complication when they take them. We don’t also get our medicines when we come to the hospital and they are prescribed for us to buy them outside.

R5: I will advise that during our education the doctors should caution us on the herbal medicine because some of the herbalist tries to convince the patients on curing them when they take the medicine. I had an encounter with a herbalist trying to persuade me to buy the drug on notion of it curing the disease so I think it should be an issue of concern because the rural areas will be a target.

R3: In Akawey diabetic center, we don’t always get all our drugs when we come here, we sometimes get half of it and later buy the rest outside as compare to the Okomfo Anokye teaching hospital where you are giving all your drugs anytime you are scheduled for an appointment. This is an issue of concern and hence needs to be addressed because patients do not attend the hospital because of these reasons.

R1: I think we should be educated on the side effect of the drugs because some of the drugs affect the man by causing dysfunction of the male organ. When you visit korle bu, they give drugs that will prevent the side effect. Some people end up having side effects because they don’t have the finances to purchase drugs that will be good for them, so I also suggest the NHIS should cover some the important drugs.

**I: Thank you all very much.**
