# Supplementary material for: Diabetes self-management education interventions and self-management in low-resource settings; a mixed methods study
Source: PLoS One. 2023 Jul 14;18(7):e0286974. doi: 10.1371/journal.pone.0286974 (PMC10348576; doi:10.1371/journal.pone.0286974)
Supplement: S13 File — (DOCX) [file pone.0286974.s015.docx]

**IDI facility yyy 1**

**I: Today is Friday, xxth of January and we have madam A with us at facility yyy. I am going to have an interview with Madame A.**

**I: Madam A, how long have you had diabetes?**

Madam A: One year and five months.

**I: Madam A how old are you.**

Madam A: adult year old

**I: I want you to tell me what you know about self-management education. When we say diabetes self-management, what does it mean to you?**

Madam A: The little I know is that, you reduce the intake of carbohydrates, exercise, taking in a lot of water and having enough rest.

**I: Madam A, what do you think must be the minimum thing that we must teach people with diabetes diabetes? The minimum education that somebody who is newly diagnose must know.**

Madam A: They have to know about their choice of food, take in more water and minimize the intake of sugar.

**I: Do you think the teaching session should be face-to-face or virtual? And why?**

Madam A: Face to face.

**I: Why?**

Madam A: With the virtual most people will not understand. The patient may not also get the chance to ask questions so he or she can understand. On the other hand, with the face to face, the patient get the chance to interact with the interviewer and can ask question.

**I: What about COVID, do you think it has affected diabetes care in any way.**

Madam A: Diabetic patient are still able to move around, and this make them susceptible to covid.

**I: If we were to use the internet to deliver diabetes education, do you think it would work?**

Madam A: I don’t think so because some patients do not have access to the internet so virtual education will not benefit these people.

**I: So where do you think we have to do self-management education. Do you think we have to do it in the communities or in the hospital?**

Madam A: I think it should be done in the communities because some people in the community are not even aware that they have diabetes. Taking the education to the communities will make these people aware of the condition, unlike the hospitals where almost all people that visit know their status.

**I: Who do you think is the best person to give the education? The health worker, the patient or nurse.**

Madam A: The health worker.

**I: Which group do you think it should be, nurses or doctors?**

Madam A: Both.

**I: Who do you think will be in the best position to do the teaching?**

Madam A: The nurses.

**I: Do you think the education should be one time? Teaching everything from morning to evening or it should be done only when a patient comes to the hospital. Which will be better?**

Madam A: I don’t think it will be helpful to teach everything at once. It will make people forget. But when done in bits, the patient’s knowledge on diabetes develop incrementally.

**I: We teach the people about their choices of food and the quantity they needed but they don’t abide by it. What do you think is responsible for that?**

Madam A: What I can say is that, if someone checks his or her blood sugar and it tends to be normal. It make the person feels he or she is getting better and therefore returns to old habits.

**I: What else prevent people from adhering to the education that we give?**

Madam A: Most diabetic patients find it difficult to go by the strict diet prescribed to them. Some patients do not even have the gadget to check their blood sugar at home which make it impossible for them to know how their diet is affecting them. They only become aware when they visit the hospital.

**I: In facility yyy, how would you rate their self-management education? Do you think when it comes to education they do well or they don’t do well? What has been your experience with them?**

Madam A: For me, as I said I was only diagnosed a year and some months ago and sometimes the nurses do give some education before they take our vitals. It helps because there are some things they teach that am not aware. The teachings helps me learn certain things.

**I: Thank you very much.**

Madam A: You are welcome

**I: This will be the end of our interview.**
