# Supplementary material for: Diabetes self-management education interventions and self-management in low-resource settings; a mixed methods study
Source: PLoS One. 2023 Jul 14;18(7):e0286974. doi: 10.1371/journal.pone.0286974 (PMC10348576; doi:10.1371/journal.pone.0286974)
Supplement: S14 File — (DOCX) [file pone.0286974.s016.docx]

**Transcription on Diabetes Self- Management Education at facility xxx on x^st^ Feb 1957**

**I: How long have you been living with diabetes?**

R: I will be several years in March this year since I was diagnosed of Diabetes.

**I: Do you take prescribed medication or insulin injection to manage the diabetes?**

R: I take the medication as prescribed. I haven’t been given an insulin injection since I was diagnosed.

**I: What are the things diabetic patients supposed to do to self-manage the diabetes?**

R: I think diabetic patients need to take their prescribed medication on time. They also need to exercise regularly and eat healthy foods to manage the diabetes. Patients need to adhere to all the instructions relation to managing the diabetes and visit the doctor on a regular basis.

**I: What kind of foods are diabetic patients supposed to eat?**

R: According to the leaflet I was given at the hospital, diabetic patients are supposed to eat some kind of foods in a proportion so that they can stay healthy and strong. Some of the foods we can eat include soupy foods, vegetables and less sugary foods.

**I: Which group do you think should deliver the education to diabetic patients?**

R: I think the doctors, nurses and patients living with the diabetes for a long period should educate the diabetic patients on how to manage themselves.

**I: How should the education be done? Would you prefer face to face or virtual (over the internet, TV or radio) delivery of the education?**

R: I would prefer the virtual delivery of the education. The virtual education over the TV or radio can reach many people in a short time as compared to the face-to-face education.

**I: How often should the education be done? Should it be delivered at once or held at different time schedule for patients in a group?**

R: I would prefer that the education should be done based on different time schedule for patients in a group. It could be in on a weekly or monthly basis.

**I: Where do you think the education should be held? Should it be in the hospital, in the community or at a hired place?**

R: I think the education should be held in the clinics or hospitals. The education can be done in the communities when the patients giving prior notice in the various communities.

**I: In your opinion, what do you think are the barriers to behavioral change in patients despite the fact that they have been given diabetes self-management education?**

R: I think the indiscipline behavior of patients and lack of support from others can be considered as a barrier to behavioral change in patients. I also think most patients are not able to afford the medication.

**I: How would you evaluate the education of diabetes in terms of performance in facility xxx?**

R: I would say the hospital is performing well in educating patients on how to manage diabetes.

**I: What particular education should health professionals give to diabetic patients when they visit the hospital?**

R: I think doctors should particularly educate diabetic patients on how to take prescribed medication on time and their choice foods and the time to take them in order to stay healthy.

**I: Thanks for your time.**

R: Thanks.
