# Supplementary material for: Diabetes self-management education interventions and self-management in low-resource settings; a mixed methods study
Source: PLoS One. 2023 Jul 14;18(7):e0286974. doi: 10.1371/journal.pone.0286974 (PMC10348576; doi:10.1371/journal.pone.0286974)
Supplement: S15 File — (DOCX) [file pone.0286974.s017.docx]

**IDI Patient facility xxx**

**I: Today is February xth , we are in facility xxx. Am doing an in-depth interview with one patient, and he is going to give us insight into diabetes.**

**I:  Madam you are welcome.**

R:  Thank you.

**I:  The reason we are doing this is that, we've seen our patience with diabetes doesn't do well. And people who are younger than forty have a long life expectancy so it's critical that we get it right.  We want to understand from you the best way to go about educating, especially people with diabetes below forty years. When diabetes starts early, you really have to get it correct because the person has a long life ahead. I want to know in your own opinion, when it comes to educating someone with diabetes, what is the minimum that we have to teach them?**

R:   We should educate them on diets and encourage them to take their medications daily and regularly without missing because I know when you miss medications, it does not work as it should.

**I: Thank you very much.**

**I:  How do you think the education should be done? Classroom setting or ad hoc? Should it be in a structured form or the health professionals teach according to their understanding?**

R: I think if possible the education can be done on radio or television or whenever they visit the clinic, a minute or two education on diabetes will be better for us.

**I: You mean it shouldn't be like a classroom but as and when the patient comes to the hospital, it should be delivered in short durations.**

R: Yes.

**I:  You mentioned one on one, do you think that will be better than in group settings.**

R:  I think grouping will be better.

**I:  You think the group setting will be better. But should it be done face to face with the instructor or virtual via the internet?**

R:  With the internet, most of us will not be able to get access due to their kind of phone or their level of incomes. However, if it can be organized for the diabetic patients to meet maybe once or twice a month to learn from each other. Each diabetic patient has different symptoms and challenges and if they can share their experiences with each other, it will help encourage others and give hope

**I:  Has the covid affected you in any way?**

R: Yes, I have to be careful now about the distance between others and myself when am interacting with them.

**I:  What about the ability to do the things necessary to take care of yourself as a diabetic patient? Have you had any challenges with getting food and your medications due to covid?**

R:  Things are a little bit expensive now.

**I:  Is it the food or the medication?**

R: The food. Vegetables, fruits and things like that.

**I:  Should the education be done in the hospital, the community, or at a special venue.**

R:  I think doing it in the hospital will help.

**I:  How?**

R:  It will be less expensive to host it in a hospital than to rent a place elsewhere which I think is not sustainable.

**I:  Despite the fact that we educate patients, they do not change. What reasons prevent people from changing?**

R:  Lack of education on the part of family and friends is a factor. Lack of encouragement and uninformed rhetoric’s can really influence patients. Family and friends sometimes misinform the patients by telling them to do otherwise by not going contrary to the doctors advice and they end up listening to them.

**I:   Are there any areas of diabetes self-management that is confusing to you and may need clarification?**

R:  I was diagnosed recently so am now learning.

**I:  What about here? Have you had any education from this facility?**

R:  No, please.

**I:  How long have you had diabetes?**

R:  Four months.

**I:  Is there anything else that you want to tell us?**

R:  I hope that I get to learn more about how to deal with this condition and take care of myself.

**I:  Thank you very much.**

**IDI Patient xxx**

**In-dept. Interview 8 Facility xxx 01-02-1957**

**XXX/IDI/PLD-002**

**I: You are welcome daddy.**

R: Thank you.

**I: How many years have you being living with diabetes?**

R: Six (6) years.

**I: Please do you take the drug orally or inject it.**

**R: I inject it when I was admitted here.**

**I: How long did you inject the drugs?**

R: One and half years because currently, I dont have refrigerator to store the drug.

**I: Which means you now take them orally.**

R: Yes, I take the metformin.

**I: What do you think people living with diabetes needs to do in order to take care of himself or herself?**

R: They should desist from the following habits, eating late in the evening, eating of fresh fish, taking in alcohol, smoking and over eating, but must adopt the habit of exercising regular.

**I: Please is there any other**.

R: Overeating (I: Can you explain) Sometimes people over eat even if they are satisfied which is also not help. It depends on the time you take breakfast. In the morning when you eat breakfast by five hours’ time you eat a little and in the evening you take in something little before six o’clock which is ok.

**I: Which group of people do you think should help in the delivering of this education? Do you prefer the doctors, nurses or people living with diabetes?**

R: I think the doctors and nurses are the best people to help deliver the education.

**I: Do you think the education should be done face to face or virtually (television, radio and phones)**

R: I think face to face will be best because most of us, dont have time to watch the television. The information centers should also involve themselves in the education from time to time.

**I: Do prefer it in groups or one on one with the doctors.**

R: I prefer the one on one.

**I: Do you think it should be delivered in the hospital or the communities.**

R: I think the hospital is better because in the communities or the rural areas they will still prefer to be near the hospital even if they are gathered somewhere else.

**I: Do you think the doctors here are doing well with regard to diabetes self-management.**

R: The doctors are very good here especially the one who takes care of me, he is very good and delivers proper healthcare to me.

**I: What are the factors that hinders patients from doing what they are told to do by the doctors?**

R: Patients who are not discipline by nature will not heed to doctors advice because they will end up eating food they are not supposed to eat. I usually eat very late in the evening when I was not diagnosed of these diseases. I used to smoke a lot but I took the decision not to smoke again, I was not giving any medication but I managed to stop because I stopped buying. In addition, lack of finances is also a factor.

**I: Do you think the education should be organized one-day, every week or monthly.**

R: One day is not advisable and wont help patients because some of them can mis most the session because of other schedules. Example if it being delivered on the radio one can easily mis the programme so it should be consistent because it is a deadly disease.

**I: What do you think the doctors should emphasis on during the education sessions?**

R: They should stress on our choice of meals we take, the excessive intake of sugar, over eating, late eating in the evening and the effect of taking in fatty food.

**I: Thank you for your time.**

**IDI patient XXX**

**XXX/IDI/PLD-003**

**Transcription on Diabetes Self- Management Education at Facility xxx Polyclinic on 1^st^ Feb 1957**

**I: How long have you been living with diabetes?**

R: I will be 18 years in March this year since I was diagnosed of Diabetes.

**I: Do you take prescribed medication or insulin injection to manage the diabetes?**

R: I take the medication as prescribed. I haven’t been given an insulin injection since I was diagnosed.

**I: What are the things diabetic patients supposed to do to self-manage the diabetes?**

R: I think diabetic patients need to take their prescribed medication on time. They also need to exercise regularly and eat healthy foods to manage the diabetes. Patients need to adhere to all the instructions relation to managing the diabetes and visit the doctor on a regular basis.

**I: What kind of foods are diabetic patients supposed to eat?**

R: According to the leaflet I was given at the hospital, diabetic patients are supposed to eat some kind of foods in a proportion so that they can stay healthy and strong. Some of the foods we can eat include soupy foods, vegetables and less sugary foods. Example when you want take in kenkey we have sizes that you are supposed to take and if you prefer to take fufu you have to know how to prepare it and with soup we have types that you can be taken enough whiles other soups can be taken in small portion example light soup can be taken throughout the week but with palm nut and groundnut soup you cant take it twice a week, if you want to take it you have take all the oil from it.

**I: Which group do you think should deliver the education to diabetic patients?**

R: I think the doctors, nurses and patients living with the diabetes for a long period should educate the diabetic patients on how to manage themselves.

**I: How should the education be done? Would you prefer face to face or virtual (over the internet, TV or radio) delivery of the education?**

R: I would prefer the virtual delivery of the education. The virtual education over the TV or radio can reach many people in a short time as compared to the face-to-face education.

**I: How often should the education be done? Should it be delivered at once or held at different time schedule for patients in a group?**

R: I would prefer that the education should be done based on different time schedule for patients in a group. It could be in on a weekly or monthly basis.

**I: Where do you think the education should be held? Should it be in the hospital, in the community or at a hired place?**

R: I think the education should be held in the clinics or hospitals. The education can be done in the communities when the patients giving prior notice in the various communities.

**I: In your opinion, what do you think are the barriers to behavioral change in patients despite the fact that they have been given diabetes self-management education?**

R: I think the indiscipline behavior of patients and lack of support from others can be considered as a barrier to behavioral change in patients. I also think most patients are not able to afford the medication.

**I: How would you evaluate the education of diabetes in terms of performance in the Facility xxx Polyclinic?**

R: I would say the hospital is performing well in educating patients on how to manage diabetes.

**I: What particular education should health professionals give to diabetic patients when they visit the hospital?**

R: I think doctors should particularly educate diabetic patients on how to take prescribed medication on time and their choice foods and the time to take them in order to stay healthy.

**I: Thanks for your time.**

R: Thanks.

**IDI patient xxx.**

**XXX/IDI/PLD-004**

**TRANSCRIPTION AT FACILITY XXX POLYCLINIC – IN-DEPTH INTERVIEW 6 0N 1^ST^ FEBRUARY, 1957**

**I: How long have you had diabetes?**

R: I have had diabetes for the past nine (9) years.

**I: Are you on insulin or you are on oral medication?**

R: I am on insulin. I only take Mix tag.

**I: So how long have you been using the insulin?**

R: I have been using insulin since I was diagnosed of diabetes for the past nine years.

**I: What do you understand by Diabetes Self-Management?**

R: Diabetes Self-management is all about how a diabetic patient is able to take care of themselves by controlling their diets, exercising regularly, and taking prescribed medication as directed by the doctor. Diabetes is brought about high increase of sugar in the body therefore; patients have to control their diets by taking food with low or no sugar.

**I: How should the Diabetes Self-Management Education be delivered? Should it be face to face or virtual (over radio, or internet)? Which one do you think is best for the patient?**

R: I think the face to face education would be an appropriate method of educating the patients since a lot of the patients would not be able to use or access the virtual method of education.

**I: Should the education be done on a one on one basis or in a group? Which one would you recommend or prefer?**

R: I think the education should be done in a group. Patients should be gathered so that they can be taught like students in a school.

**I: Do you think there should be a structure or a syllabus for the education? Or should patients be told what to do without a structure?**

R: I think the education should be done step by step so that every patients can know and understand what is being taught.

**I: Who do you think should give this education? Do you think the health care professionals alone should give the education or patients living with diabetes can also play a role?**

R: I think both the health professionals and patients living with diabetes for a period can educate patients about the diabetes.

**I: Where do you think the education should be done? Should it be done in the hospital, or in the community or a hired place?**

R: I think the education can be done in both the hospital and the community once there is an opportunity to gather diabetes patients.

**I: Do you think the education be done at once or should be done in bits or at a different time schedule? Which one would be ideal?**

R: I think all the education should be given to the diabetic patients at one time when there is a chance to do so.

**I: What do you think are some of the barriers to behavioral changes diabetic patients despite all the education on diabetes self-management?**

R: I think most patients are indiscipline.

**I: What particular education should patients be taught on diabetes self-management? What key areas should be taught to patients?**

R: I think the keys areas that the patients need to be educated on is taking the right kind of diet, exercising regularly and taking medication.

**I: How would you assess the education in the hospital in terms of performance?**

R: I would say the doctors are doing their best to educate the patients on how to manage the diabetes very well.

**I: What do you think the doctors can improve upon in the Diabetes Self-Management Education?**

R: I think the doctors in the hospital should recommend prescribed medication that would last longer and can be bought in the hospital. I also think there should be frequent visit to the doctor.

**IDI patient xxx**

**In-dept. Interview 5 Facility xxx 01-02-1957**

**XXX/IDI/PLD-005**

**I: Good morning**

R: Morning.

**I: Am Dr s123s, this is Dr s101s. This is a session where we want to find out what patients know and about diabetes and how they are going to manage it. We are doing this research to see how visible the new treatment will be for patient so they ca live better.**

**I: Please how long have you had diabetes?**

R: Fourteen years (14)

**I: Please are you on insulin.**

R: Yes.

**I: Please how long have you been on insulin.**

R: About 3 or 4 years.

**I: Pleas what do you know about diabetes and what are the measure, ways or lifestyles you have put in place to make sure that you leave well with this condition.**

R: I know diabetes is a defect of the pancreas that impairs the absorption of sugar in the body. We have to eat before six o’clock, eat in modulation, minimize intake of carbohydrate and exercise regularly.

**I: Do you prefer the diabetes self-management education in a structured form or you want it the way its being done in the hospital.**

R I am very open to however manner the education would be delivered. What does the structure entails.

**I: The structured one is more of a curriculum that’s have details of the various sessions that will be taught during the education.**

**I: Would you prefer a face-to-face education or a virtual education.**

I would prefer face to face because patients can have personal interaction with one another as compared to the virtual education

**I: Do you prefer it in a hospital, home or community setting.**

R: I would prefer the community setting because it could bring about social support from people in the community that may also have the diabetes.

**I: Do you prefer it in a group session or one on one session.**

R: Am okay with all the options.

**I: How will you rate those who have educating you on diabetes self-management. Are they doing well in the hospital?**

R: They are doing well.

**I: Would you prefer it in a day, week or monthly.**

R: I think it is a matter of time so if they prefer to do it in a day I will accept it.

**I: What are some of the barriers that impedes behavioral change for patients to adhere to what the doctors have taught them?**

R: I think it is the indiscipline of the individual to conform to the doctors’ advice. In addition, the approach used by the person delivering the education should be cordial and friendly atmosphere edging the people to do as they say.

**I: Who do you think should deliver the education, the nurses or the doctors?**

R: In my case, the doctors are the first people I usually talk to and the nurses follow suit.

**I: Is there anything that can be done to improve the delivering of the education.**

R: I think there should be visual presentation for patients to remember what have being taught.

**I: What are some of the things the doctors should emphasize when delivering the education.**

R: The choice of food and their self-discipline because it is self-management

**I: Thank you so much.**

R: I have a challenge with the constant injection at a particular place, which is a problem, and need to be an issue of concern.

**IDI PATIENT XXX**

**Transcription for Facility xxx Polyclinic 01/02/1957**

**XXX/IDI/PLD-006**

**I: What do you know about diabetes and some of the measures they can adopt in order to manage diabetes.**

R: To my understanding, diabetes is the high amount of sugar in the system. It comes with different complications if not treated very early. I have also realized that I should take in more vegetables and fruit and minimize the intake of carbohydrate. It has really helped me a lot since I had this idea about the disease.

**I: When were you diagnosed with diabetes?**

R: Three (3) months ago.

**I: Are you on insulin.**

R: Yes

**I: How long have you being on insulin.**

R: Three (3) month.

**I: Do you want this education to be delivered in a structured form or the normal way it’s done at the diabetes clinic.**

R: I believe it will be much beneficial if it is structured, they will be able to track the progress.

**I: How long would you prefer these teachings to be, would you prefer a one-day affair, long period or you like it in bits.**

R: I would prefer it to be in monthly basis.

**I: I: Do you think the education should be delivered face to face or virtual (radio or internet)**

R: I would prefer face to face because you will be able to express your taught and feelings better than **being** done virtually.

**I: Where would you also prefer it to be held, in a hospital, home or community setting?**

R: Community setting would be better because there are lots people are likely to be prone to diabetes **and** have not received any education. People without diabetes can also learn as well and acquire some knowledge about diabetes.

**I: Would you prefer it to be a one on one session or in a group.**

R: I think the group will be very effective because we can learn from each other despite the fact that we are being taught by the health facilitators.

**I: Which group of people would you prefer to help disseminate this information to the patients?**

R: The doctors.

**I: Why do you think it’s the doctors?**

R: They have better understanding and can provide better answers to all the questions provided during the education session.

**I: Are there things the doctors can do to make the teaching sessions understandable for people taking the study.**

R: I think the approach used in the discussion must be taking in to consideration because of the nature of the disease and it effect to the patients.

**I: Have anyone taking you through diabetes education.**

R: Yes, that was when I was admitted at this hospital and after that, I did some researches on it. I also watch documentary on television about diabetes, which helped me contain and manage it.

**I: How would you rate the way they manage or educate patients on diabetes here, are they doing well or not.**

R: They have been doing well because the doctor who took care of me from time to time calls me to enquire if am going through other complications.

**I: Are there other things they should improve on.**

R: I think they are doing their best, which I personally appreciate.

**I: What do you think impedes behavioral change when it comes to diabetes self-management?**

R: I think it is a lifestyle they were living before and adopting to new ones becomes very difficult especially their choices of food, the daily injection of insulin and medication.

**I: Is there any other thing that make people do otherwise after them being educated about self-management.**

R: Lack of education or knowledge they have on diabetes.

**I: Thank you for your time.**

**IDI patient xxx**

**Transcription on In-dept. Interview 3 Facility xxx 01-02-1957.**

**XXX/IDI/PLD-007**

**I: When did you realized you had diabetes?**

R: About 10 years ago.

**I: Do you take insulin.**

R: Yes.

**I: When did you start using the insulin?**

R: About 10 years.

**I: How can a patient with diabetes manage it?**

R: A patients can manage diabetes by being cautious of the food they eat. They should not also eat late in the evening at least by 4:00 to 5:00 they should have finish eating. The types of food they should be eating is plantain and they should desist from taking in alcoholic drinks.

**I: How would you want the education to be delivered, in a day, on daily basis, weeks or months?**

R: I would prefer it on daily basis.

**I: Would you prefer it will be done in the hospital, house or in the community.**

R: I prefer it in the hospital.

**I: How would you want the education to be organized? Do you want it in group or face-to-face?**

R: I would like it face to face.

**I: In delivering the education what topics do you think the doctors or the professionals should put** emphasis on.

R: I think they should emphasize on the choice of meals we should eat and the importance of the drugs giving to us.

**I: How would you rate the level of education delivered to patients in this hospital in the aspect of** **diabetes self-management. Do you think it better or not?**

R: They are very good in delivering the services.

**I: Why do you think patients do not obey what they are being taught by the doctors?**

R: I think it lack of finances because patients don’t have the money to purchase food even after they have taking their drugs, so they intend skipping some of their drugs so that when they think there is food available, they will take them. Finances are some of the barriers that prevents patients from doing what they are told to do in other to manage diabetes.

**I: Thank you very much for you time.**

**Transcription on In-dept. Interview 2 Facility xxx Polyclinic 01-02-1957**

**XXX/IDI/PLD-008**

**I: How long have you had diabetes?**

R: Nine (9) years.

**I: Are you on insulin or oral medication.**

R: Am on insulin and oral medication.

**I: Please how long have you being on insulin.**

R: Eight (8) years.

**I: What do you know about diabetes self-management? What patients needs to do in order to manage their diabetes well**.

R: Diabetes is not about high sugar but the way you manage yourself, how you control it by eating the food you are supposed to eat example cocoyam leaves, vegetables and avoid the intake of less carbohydrate. Most of the people living with diabetes dont have much idea about it and they dont have people to control them on what to eat and what to avoid. My mother had diabetes before I did so she was the one directing me on the dos and dont.

**I: If we are to deliver education on diabetes self-management, do you want it face to face or virtually.**

R: I would say we should do it face to face because some are not much educated. Those uneducated will understand better if they sit with the doctor face to face.

**I: You think the face is ideal than the virtual.**

R: Yes.

**I: How do you think this education should be delivered, by a structured form, by structured I mean it having a curriculum or we should just do it the way we do in the consulting room by just talking to you without any structure.**

R: The structured form is better.

**I: Do you think the education be done one on one or in groups.**

R: I think the groups will be better than one on one.

**I: Any reason why you think the group is better.**

R: Am the shy type and cant express my feelings when it one on one but in a group am able to ask questions when others do it.

**I: You feel free to talk in a group than one on one.**

R: Yes.

**I: Which people should deliver the health care education? Do you think it should be limited to only health care professionals or other patients living with diabetes?**

R: I think the healthcare professionals are better.

**I: Why do you think they are better?**

R: They know how to talk us for us to understand.

**I: How should the education be delivered, do you think it should be done every week, monthly or** **daily.**

R: it should be every week.

**I: Any reason why you prefer it weekly.**

R: I prefer it weekly because people easily forget things when we tell them ones but when it is repeated every week they will always remember and practice it.

**I: How would you rate the self-management education delivered to you personally in this facility?**

R: It is not bad.

**I: What are the barriers that hinder patients to practice what he have been taught?**

R: At times, I feel bad using the insulin.

**I: Is it because of the stigma attached to the taking of insulin.**

**IDI patient xxx final**

**XXX/IDI/PLD-009**

**I: Today, we are at the Facility xxx polyclinic. It's the 4th of February and we are with one of our patients who has been diagnosed with diabetes in less than a year. We are going to have an interaction with her.**

**I:  Welcome madam.**

R:  Thank you.

**I:  Please what are some of the things that a diabetic patient needs to be taught?**

R:  They need to know that the intake of excessive sugary foods causes diabetes. Likewise, the intake of things that are considered harmful to the human body. Finally, they need to know that diabetes can cause one's legs to be amputated.

**I:  Are you saying that the least a diabetic patient needs to be taught is food and lifestyle choices that cause diabetes and what they can do to manage the condition?**

R:  Yes, please.

**I:  Where do you think this education should be delivered? The hospital, the church? Where exactly do you think this education should be taught?**

R:  I believe it can be taught anywhere people are convened. It could be the market, football fields, and the church, etc.

**I: Is it better to do this education at these locations than at the hospital?**

R:  No. But it should be delivered anywhere people are assembled.

**I:  I will like to ask, if you have been taught about diabetes self-management education at this hospital?**

R:  I have not been taught, but a woman who sat beside me today helped me understand a few things.

**I:  We are aware that we need to push for the education of diabetes self-management, but which group of people do you think should deliver the education? Should it be the doctors, nurses, or people living with diabetes? Which group of people should deliver the education?**

R:  In our homes, people living with diabetic have many experiences that they can share with fellow patients. They are in the best position to educate other patients on the right meals to take since they have gone through the process themselves. The nurses can also help with the education when we come to the hospital.

**I:  How do you think the education should be done? Should it be face to face? Or it should be done online or through other mediums like the radio and television?**

R:  Educating on radio and television will help a lot.

**I:  Do you know about the internet? WhatsApp and the likes?**

R: I have only heard of it but don't know much about it and therefore would not help me. Radio and television on the other hand are the best mediums through which diabetes education can be conveyed.

**I:  And would you say face to face education will help?**

R: Yes.

**I:  Do you think the education should be done in groups or it should be one on one?**

R:  I think both scenarios help.

**I:  But which one do you prefer?**

R:   I prefer the group.

**I:   Do you think the education should be one time? Teaching everything from maybe morning to evening or it should be done in bits.  Which will be better?**

R:  People may not take the education seriously and may even forget if done at a go.  So I think it should be done in bits.

**I: In dividing the education into bits, how long do you think it should last?**

R:  Having it once a week with a duration of four hours will be perfectly fine.

**I:  We've talked a lot about diabetes. Is there any other thing that you will like to add? Anything that will be useful to the diabetes community.**

R: I will entreat people to visit the hospital regularly for check-ups. If I had not come to the hospital for a check-up, I would not have known to have diabetes.

**I: Thank you very much, madam.**

R: It's my pleasure.

**IDI**

**I: Good morning.**

**R: Morning.**

**I: Please when did you realize you have diabetes?**

R: I realized I had diabetes when I was pregnant and visited the hospital for antenatal and upon the check up the doctor drew my attention that I have diabetes hence he will admit me and he started to inject me with the insulin until I had the caesarian section and they took the baby out and the doctor started the treatment. He then told me that if any member of my family have been diagnosed with diabetes in the past years then it would be possible for me to acquire it. I had a brother who was amputated and he died because he did not take care of himself well, so I needed to take very good care of myself so that it wouldn’t happen to me.

**I: It means is being 14 years since you had the diabetes.**

R: Yes.

**I: Do you take insulin.**

R: No, I take tablet.

**I: Do you want the education to be in a curriculum form or like how doctors attend to patients during hospital hours.**

R: I want it to be in a curriculum form.

**I: Do you want it to be done in one day or in bits let’s say twice or three times a week.**

R: I would prefer it to be done every month because we work daily and can’t afford to mis our work, which is also important.

**I: Do you also prefer we do it in hourly basis or for a long period.**

R: I would prefer it organized in hourly basis or in bits because we have other activities to undertake.

**I: Do you prefer the education to be organize face to face or virtually that is on the phone, television, or the computer**.

R: I think the face to face is better than virtual. Most of us would prefer that form because the use of the device virtually will be a problem and hence would like it to be face to face with the doctor.

**I: Would you prefer it’s organized in groups or one on one sessions.**

R: The group session will be the best because we can share ideas and learn from each other.

**I: Do you think the community, homes or hospitals would be better for the education to be organized.**

R: I think the community would be better because some of them dont like going to the hospital hence if it is held in the community most people will be able to attend and understand the importance of taking good care of the themselves.

**I: What are the factors that prevents patients from adhering to doctors advice?**

R: Some of the factors are the kind of lifestyles patients adopt. The intend taking in meals that needs to be avoided.

**I: What do you think the doctors can adopt to enhance the diabetes self-management education?**

R: I think they should give intervals between patients visit to the hospitals so that they can be able to take all their drugs before the next the visit.

**I: What are main topics needed to be taught by the doctors about diabetes self-management for patients to understand and desist from not obeying their advice.**

R: I think they should emphasize on the regular intake of drugs, which is of paramount important for patients to avoid experiencing complication.

**I: Who do you think should help in the delivery of diabetes self-management education? Do you think it the doctors, nurses or dietitians?**

R: I think they can all help in the delivery because they have the knowledge about it.

**I: Do the doctors here offer good services to patients pertaining to diabetes self-management education.**

R: Yes, they do and are very good.

**I: Thank very much for your time.**

**IDI-Transcription at facility yyy interview five (5).02/02/1957**

**I: Good afternoon.**

**I: How many month or year have you being living with diabetes.**

R: Its almost a year now.

**I: Do you inject the drugs or you them orally.**

R: I take them orally.

**I: What do you have to do to take care of yourself when you have diabetes?**

R: We don’t have to be eating in late in the evening. We also have to avoid the intake of sugary food and eat many vegetables.

**I: What else do you need to do apart from being cautious about your meals to take care of yourself?**

R: We need to practice regular exercise, avoid take in alcoholic drinks and desist from smoking.

**I: Who do you think should deliver the education on self-management to patients with diabetes?**

R: I think the doctors and those who are already living with the diabetes can help in the education.

**I: Where do you think the education should be delivered in the community, hospital or houses?**

R: I prefer all the options, in the community, houses and hospitals.

**I: How do you prefer it to be done, in-groups or on one on one.**

R: I prefer the one on one because it help us a lot.

**I: Why do you prefer one on one?**

R: It has made me understand diabetes well enough since I came here.

**I: Are the doctors here good in educating you on diabetes self-management.**

R: Yes, they are very good because anytime I come here; they teach me the type of food to eat and how to manage myself.

**I: Do you think it should be done face-to-face or virtual that is the use of radio, television or phone.**

R: I prefer the face to face.

**I: Why do you prefer the face to face?**

R: Most of us don’t have time to use the internet.

I: **I: What do you think prevents people from adhering to what the doctors teach on diabetes self-management and do otherwise?**

R: I think it is because of disobedience because some people go further to drink alcohol and smoke despite the education giving to them to desist from them.

**I: Do you think it because of lack of finances that will make people go contrary to what the doctors teach them.**

R: I don’t think so because some of them use the money to buy alcoholic drinks rather than taking it to the hospital to buy their drugs. Diabetes drugs don’t cost that much and insurance covers some of them.

**I: What are the most important things doctors should prioritize during the education for diabetes to patients?**

R: I think emphasis should be placed on regular exercising, eating of vegetables and fruits.

**I: Are there some things the doctors here needs to do to encourage people with diabetes.**

R: I think they should always continue to teach us because some of us dont even come to the hospital on appointed date giving by the nurse or doctors.

**I: We have come to the end of our discussion thank you.**

**IDI-Transcription facility yyy 2^nd^ ___ 1957.**

**I: Please good Afternoon.**

**I: When did you realize you had diabetes?**

R: Last year July.

**I: Do you take insulin.**

R: No please.

**I: What have you being doing to manage yourself since you had the diseases?**

R: I take my medications regular and eat very early at least before 5 o’clock in the evening.

**I: How should the education be delivered, should be structured or as its done in the clinic.**

R: I prefer it to be done as it is in the hospital.

**I: Would you prefer the education done within one a day, monthly, every week or on daily basis.**

R: I think monthly will be ok because I have children and have to cater for them so cant make it every week or every day.

**I: How would you want it to be done? Do you want it face to face, or virtually (television, radio or phone).**

R: I don’t have the gadget that would be used virtually so I prefer face to face with the doctor.

**I: Would you prefer the education organized in groups or one on one**

R: I would be available for all the options whether in groups or one on one with the doctor.

**I: Where do you think the education should be organized, in community or the hospital**?

I: I would be available if it is organized anywhere, whether in the community or the hospital.

**I: What do you think impedes behavioral change when it comes to diabetes self-management after patients have received education?**

R: I think it is because of lack of funds.

I: Whom would you prefer to deliver self-management education, is it the doctor, nurse, dietitian or the psychologist.

R: Any of them would be fine with me.

I: **Are the doctors and nurses doing well taking care of you here as in helping you to manage your diabetes.**

R: They are doing well because when you come here they will attend to you and prescribe some drugs for you to purchase.

**I: What should the doctors adopt to improve the education giving to diabetes patient?**

R: I think they should improve on the aspect of communication.

**I: Thank you very much.**

I: We have ended our discussion. Thank you.

**I: Today is Friday, 29th of January and we have madam A with us at Facility yyy. I am going to have an interview with Madame A.**

**I: Madam A, how long have you had diabetes?**

Madam A: One year and five months.

**I: Madam A how old are you.**

Madam A: 25 years

**I: I want you to tell me what you know about self-management education. When we say diabetes self-management, what does it mean to you?**

Madam A: The little I know is that, you reduce the intake of carbohydrates, exercise, taking in a lot of water and having enough rest.

**I: Madam A, what do you think must be the minimum thing that we must teach people with diabetes diabetes? The minimum education that somebody who is newly diagnose must know.**

Madam A: They have to know about their choice of food, take in more water and minimize the intake of sugar.

**I: Do you think the teaching session should be face-to-face or virtual? And why?**

Madam A: Face to face.

**I: Why?**

Madam A: With the virtual most people will not understand. The patient may not also get the chance to ask questions so he or she can understand. On the other hand, with the face to face, the patient get the chance to interact with the interviewer and can ask question.

**I: What about COVID, do you think it has affected diabetes care in any way.**

Madam A: Diabetic patient are still able to move around, and this make them susceptible to covid.

**I: If we were to use the internet to deliver diabetes education, do you think it would work?**

Madam A: I don’t think so because some patients do not have access to the internet so virtual education will not benefit these people.

**I: So where do you think we have to do self-management education. Do you think we have to do it in the communities or in the hospital?**

Madam A: I think it should be done in the communities because some people in the community are not even aware that they have diabetes. Taking the education to the communities will make these people aware of the condition, unlike the hospitals where almost all people that visit know their status.

**I: Who do you think is the best person to give the education? The health worker, the patient or nurse.**

Madam A: The health worker.

**I: Which group do you think it should be, nurses or doctors?**

Madam A: Both.

**I: Who do you think will be in the best position to do the teaching?**

Madam A: The nurses.

**I: Do you think the education should be one time? Teaching everything from morning to evening or it should be done only when a patient comes to the hospital. Which will be better?**

Madam A: I don’t think it will be helpful to teach everything at once. It will make people forget. But when done in bits, the patient’s knowledge on diabetes develop incrementally.

**I: We teach the people about their choices of food and the quantity they needed but they don’t abide by it. What do you think is responsible for that?**

Madam A: What I can say is that, if someone checks his or her blood sugar and it tends to be normal. It make the person feels he or she is getting better and therefore returns to old habits.

**I: What else prevent people from adhering to the education that we give?**

Madam A: Most diabetic patients find it difficult to go by the strict diet prescribed to them. Some patients do not even have the gadget to check their blood sugar at home which make it impossible for them to know how their diet is affecting them. They only become aware when they visit the hospital.

**I: In facility yyy, how would you rate their self-management education? Do you think when it comes to education they do well or they don’t do well? What has been your experience with them?**

Madam A: For me, as I said I was only diagnosed a year and some months ago and sometimes the nurses do give some education before they take our vitals. It helps because there are some things they teach that am not aware. The teachings helps me learn certain things.

**I: Thank you very much.**

Madam A: You are welcome

**I: This will be the end of our interview.**

**IDI PATIENT FACILITY YYY**

**TRANSCRIPTION ON DIABETES SELF-MANAGEMENT EDUCATION- AN INTERVIEW WITH AN ELDERLY MAN AT AKAWEY HOSPITAL ON 1^ST^ FEBRUARY, 1957**

**I: Please how old are you?**

R: I am 68years old.

**I: How long have you been living with diabetes?**

R: I have had diabetes for over 10years now.

**I: Do you take insulin or tablet? Or do you take both?**

R: I take tablets. I don’t use insulin.

**I: What education should be given to diabetic patients?**

R: I think diabetic patients should be educated on the kind of food to eat and the time of eating, and things that patients are not supposed to do.

**I: What minimum education do you think should be given to patients living with diabetes?**

R: I would say patients should be educated particularly on taking their prescribed medication and the time of taking medication. They should also be educated on the kind of food to take on regular basis.

**I: How should the education be done? Should it be face-to-face or virtual? Which method would be helpful or preferable?**

R: I think the education should be face to face because it is more interactive and easier.

**I: Do you think the virtual education would be helpful because it is beneficial to everyone?**

R: I think face-to-face education would be the best because only few people would be able to access and use the internet with virtual form of education.

**I: Do you still think the face-to-face education is better even in the Covid-19 era?**

R: I think the face-to-face education is better than the virtual education.

**I: Has the Covid-19 affected your ability to self-manage the diabetes in any way?**

R: I haven’t really been affected by the Covid-19 but I have been careful to observe all the protocols. We are been educated to wash our hands regularly, wear facemask, and avoid crowded places.

**I: Should the education be held at once or at a different time schedules when patients visit the hospital? Which one would be helpful and preferably?**

R: I suggest the education should be held on different time schedule or bit by bit because when all the education is done at once, the patients can easily forget what they have been taught,

**I: Which group do you think should deliver the education to diabetic patients? Is it the health professionals or patients who have had diabetes for a long time?**

R: I think the nurses are the best group to deliver the education because they are able to interact more with the patients.

**I: Where do you think the education should be held? Should it be held in the hospital, in the churches, in the communities or at a hired place?**

R: I think the education should be held in the hospital because everyone can visit the hospital on any day for treatment.

**I: How long do you think each education session should last?**

R: In my opinion, each education session should last for about 20 to 30mins depending on the time the doctor would report.

**I: What are some of the things you think would be needed by the hospital for an effective education?**

R: I think the hospital may need insulin pins, needles, and the glucometer for an effective education.

**I: Do you think the education should be done on a one on one basis or in a group? Which one would you prefer?**

R: I would prefer that the education should be done in a group so that the diabetic patients can learn from one another.

**I: How long have you been coming to this facility for your treatment?**

R: I have been visiting this hospital for the past 5years for medical treatment.

**I: How would you assess the self-management education program of the hospital? Is it good or is helpful? How can they make it better?**

R: I think the education at the hospital is helpful and good depending on the one who delivers it. The education they deliver is very good, understandable and helps us to practice what they have taught us.

**I: What are the barriers to behavioral changes despite the self-management education to diabetes patients?**

R: I think some are indiscipline because they end up going contrary to what they have been taught by the doctors, example taking in food they are not supposed to eat. In addition, lack of finances also causes behavioral change because they can’t afford to eat frequently and eat the right type of meal.

**I: What advice would you give to diabetic patients?**

R: I would advise diabetic patients to take prescribed medication on time. They should also eat healthy foods such as fruits and vegetables and eat on time.

**IDI patient Facility yyy 1**

I: When did you realize you have Diabetes?

R: I was diagnosed of Diabetes last year

I: Which means its being a year now since you had diabetes. It has been a year now.

R: Yes.

I: Are you on insulin?

R: No please, I am not on insulin.

I: What are some of the things you do to self manage diabetes?

R: I have been eating healthy food like plantain with cocoyam leaves stew; I take fruit in the morning and eats on time eating to avoid eating late in the night.

I: Do you think the Self-Management education should be structured (with syllabus) or unstructured?

R: I think the education should be unstructured. It should still be done like the way it is done in the hospitals.

I: How should the education be done. Do you prefer it at once or in bits?

R: I think the education should be done at once so that patients would know how to manage the diabetes when we visit the hospital.

I: Do you think the education should be held at the hospital or in the community?

R: I think the education should be held in the hospital.

I: Should the education be done on a one on one basis or in a group?

R: It should be done in a group so that patients can learn from one another

I: Do you think the education should be face-to-face or virtual?

R: I think the education should be done live or face to face because it is easier and faster

I: What are some of the barriers to behavioral changes despite diabetes self-management education being delivered?

R: Some of the barriers to behavioral changes could be the lifestyle of the patients, lack of understanding by the patients and financial challenges.

I: Which group of people should deliver the education?

R: I think the health care professionals should deliver the education because they have in-depth knowledge of the diabetes.

I: What are some of the logistics that are needed for the education?

R: There should be communication devices to help in delivering their speeches

I: How would you grade or assess the education from the hospital in terms of performance?

R: The doctors are doing well.

I: What should be the focus of the education?

R: The education should focus on our dieting, drinking habits and our medication.

I: Thank You for your time

R: Thank you too

**IDI patient facility yyy**

**I: Today is the 2^nd^ of February 1957 and we are at the facility yyy. Today we are going to have a conversation with one of our patients.**

**I: You are welcome madam.**

R: Thank you.

**I: How many years have you had diabetes?**

R: Four years.

**I: How old are you?**

R: I am 38 years old.

**I: You are not yet 40?**

R: Yes.

**I: What are some of the thing that diabetic patients needs to be thought and needs to understand?**

R: Most importantly, they need to know what to eat. When you are diagnosed of diabetes, you cannot eat as you used to. If you used to take two balls of banku, now you will have to take a ball. Excessive eating of meat needs to be stopped and substituted for dry fish. Sugary drinks needs to be substituted for natural fruit juice. When your parents have diabetes, you need to be careful because the disease is genetic. Diabetes was known to be a disease of the old but now a days, year old babies are being diagnosed with it therefore care needs to be taken. I will appeal to the younger generation to be careful with what they consume, the sugary foods and all that would not help. We have the freedom to eat whatever we like but not all is good for our health. I am only 38 years old and have diabetes, care therefore needs to be taken. I used to have a sweet tooth but when I got to know that diabetes is what killed my parents and other family members on my mom’s side, I forego I a lot of things I used to do. I will continue to appeal to the younger generation, those with diabetes and otherwise to be careful of what they eat. It is unfortunate but one can get the disease irrespective of whether their parents had it or not. If you already have diabetes, you need to heed to the advice of your doctor. Things have changed and we need to be careful because anyone can get the disease.

**I: In short, what you are saying is we need to hammer on the importance of good diet and also the possibility of getting the disease whether it is in the family or not. In addition, patients’ needs to also heed to the advice of doctors.**

R: Exactly. I will also plead with you guys to give us free medications. Not everyone can afford their medications. The medications are very expensive considering that most people need it to survive. I will channel my grievance through you to the government to provide our medications for free.

**I: You talked about money and diet. We have seen that sometimes we educate patients on what to eat and also how to take their medication but some patients do not heed. What are some of the things that prevent patients from heeding to these advice?**

R: Some patients cannot afford the medications prescribed to them and only visits the facility when their blood sugar is out of the roof. The patients are willing and are ready to follow the doctor’s advice but lack of finance on their side and lack of support on the part of government render them incapable.

**I: You mentioned support and money, and that lack of these two can make it difficult for some patients to manage their condition. Can you elaborate on how lack of support can make self-care difficult?**

R: Financial support from friends for example can be of great help.

**I: Is there any other support that can help patients?**

R: Friends can put patients on their toes by rebuking them when they fail to follow the doctor’s advice. Friend can remind patients about the consequences of their bad behaviours.

**I: Would it be beneficial for health professionals to ask patients to come along to the facility with other people? Or it will be better to ask patients to come alone? Which would you prefer?**

R: Most diabetes patients feel reluctant to come to the hospital. I will plead with your guys to educate patients at each and every diabetic centre. Some people are very shy and wouldn’t want anyone to know they have diabetes so I think going to their centre will make them feel more comfortable. None of my friend know me to have diabetes because I can’t trust them to keep my secret. Before I know it, people will be pointing at me in the streets. People can stigmatise against you just for having diabetes.

**I: You feel that the education should be done at diabetes centres but not necessarily home and in the communities.**

R: The education can be done in churches because a lot of people are in need of help. I for example, I didn’t know I had diabetes until I attended a program. At the program they checked our blood sugar for free and that is when I got to know that my blood sugar was at 18 and over. It was a few weeks after I had giving birth. You can go to churches to educate the congregants. Someone may have diabetes but may not know how to get help and may resort to herbal medicine.

**I: So you think that taking the education to churches and the communities will be helpful?**

R: Yes.

**I: What about renting a venue? Will that help?**

R: No, it wouldn’t help. Myself I wouldn’t come to such a place because I would not want to meet someone I know. When you take the education to the churches for example, it will encourage people to come to your facility, but will be difficult for them to come forward in public places.

**I: Why don’t you want anyone to know you have diabetes? Are you scared of being stigmatized?**

R: People like to gossip. When people get to know that you have diabetes, they begin to gossip about how sick and frail you are and that can demoralize you when you get to know about it.

**I: Are you saying that renting a place will be a waste of money and that not everyone will attend?**

R: Maybe the elderly, but the younger ones like myself will not attend for fear of stigmatization.

**I: Which group of people do you think is best suited to deliver the education? Should it doctors, nurses or other patients living with diabetes?**

R: Doctors, dieticians and diabetic patients with experience are all qualified to deliver the education. Some people have lived with the disease for over 40 years but are still alive. Younger patients can therefor get valuable knowledge from these people in order to live long.

**I: Let’s focus on this facility, Facility yyy where you come for support. How do you see their diabetes education? What can they do to improve in their work?**

R: We are not always taking through diabetes self-management training when we come here. It will be good if they can do it every day. We need to be always reminded on how dangerous the condition and the need to take our medication and be cautious about the choice of meals. Sometimes it takes over a month for someone to meet with us. In addition, we are ask to run some lab test but when the results come in, the doctors don’t tell us anything about the results. They just input the result onto their system without letting us know how it affects our condition hence we cannot give feedback to our relatives when they ask about the result of the test so I think they should endeavor to relay information to us after the lab test..

**I: How often do you think the education should take place? Should everything about diabetes self-management be thought in a day? How should it be done?**

R: Doing everything in a day wouldn’t help us. There are a lot of things we deal with and it will be difficult to remember if it’s all done in a day? What will help is when the education is delivered whenever we come to the hospital

**I: For how long do you think the education must last? Is it 30 minutes or maybe an hour?**

R: An hour will do considering the amount of question and answers that will be exchanged. Thirty minutes for question and another thirty for answers.

**I: Considering covid, do you think the education should be virtual or face to face? Which method will be best?**

R: Face to face is better. I may be on WhatsApp but might not know how to read. But if we can’t meet because of covid, messages can be conveyed in audio like a voice note. We can listen to it from time to time.

**I: You’ve said something that no one has mentioned before. Do you think patients will listen to voice messages?**

R: Of course we will listen.

**I: How many times should the voice messages be sent to you? Should we send it daily? How do you think it must be done for patients to listen?**

R: You can send it maybe once in a week, and it should be a long audio. When it is long, we wouldn’t be in a rush to listen to everything. We will take our time and will be ready and in anticipation to receive the following weeks voice message. The voice messages can be made in several local languages and this will make it possible for all to understand.

**I: Would it help if we use the internet?**

R: I don’t visit the internet. I only use WhatsApp.

**I: Are you saying the internet wouldn’t help?**

R: It will help some people but not all.

**I: Has covid affected you in anyway?**

R: Yes it has affected me.

**I: How has it affected you? Is it in taking care of yourself financially or in managing the diabetes?**

R: It has affected my finances.

**I: What do you do?**

R: I am a trader. I was fortunate to get help from relatives abroad in setting up a business because you can’t move around a lot when you have this condition. I used to collapse from time to time when I first got diabetes and will eat but go hungry in just a little while. Business is not good as a result of the covid.

**I: You’ve said a lot, and it’s not going to help not just you, but other diabetic patients. Is there any other thing that you would like to add?**

R: We the ladies, the condition causes our private part to itch. Sometimes I scratch till it blisters. If you can educate us on what to do and which medications to use when such a situation arises, it will be helpful to us.

**I: Thank you very much.**

R: Happy to be here.
