# Supplementary material for: Diabetes self-management education interventions and self-management in low-resource settings; a mixed methods study
Source: PLoS One. 2023 Jul 14;18(7):e0286974. doi: 10.1371/journal.pone.0286974 (PMC10348576; doi:10.1371/journal.pone.0286974)
Supplement: Attachment — Submitted filename: PONE-D-22-15096_R1.pdf [file pone.0286974.s022.pdf]

PLOS ONE

Diabetes self-management education interventions and self-management in low-resource settings; a mixed methods study

--Manuscript Draft--

**Manuscript Number:**	PONE-D-22-15096R1
**Article Type:**	Research Article
**Full Title:**	Diabetes self-management education interventions and self-management in low-resource settings; a mixed methods study
**Short Title:**	Running title: Structured? No; Literate? No; Pamphlets? Yes!; DSME in resource-constrained settings
**Corresponding Author:**	Roberta Lamptey<br>Korle Bu Teaching Hospital<br>Accra, GHANA
**Keywords:**	Diabetes; self-management; Self-Care; education; Health Resources; Ghana
**Abstract:**	Introduction<br>Diabetes is largely a self-managed disease and thus care outcomes are closely linked to self-management behaviours. However, structured self-management education (DSME) interventions are largely unavailable in Africa.<br>Aim<br>We sought to characterise DSME interventions in two urban low-resource primary settings, and to explore diabetes self-management knowledge and behaviours of persons living with diabetes (PLD).<br>Research design and Methods<br>A convergent parallel mixed-methods study was conducted between January to February 2021 in Accra, Ghana. A total enumeration was done for the cross-sectional study whilst purposive or judgemental sampling was used in selecting participants for the qualitative study. Multivariable regression models were used to study the association between diabetes self-management knowledge and behaviours. We employed inductive content analysis of informants' experiences and context to complement the quantitative findings.<br>Results<br>In total 425 PLD (70.1 % (n=298) females, mean age 58 years (SD 12), mean blood glucose 9.4 mmol/l (SD 6.4)) participated in the quantitative study. Two managers, five professionals, two diabetes experts and 16 PLD participated in in-depth interviews. Finally, 24 PLD were involved in four focus group discussions.<br>Median diabetes self-management knowledge score was 40 % ((IQR 20-60). Every 1 unit increase in diabetes self-management knowledge was associated with increased scores on diet (5%;[95% CI: 2%-9%, p<0.05]), exercise (5%; [95% CI:2%-8%, p<0.05]) and glucose monitoring (4%;[95% CI:2%-5%, p<0.05]) domains of the diabetes self-care activities scale.<br>The DSME interventions were unstructured and limited by resources. Financial constraints, conflicting messages, beliefs, and stigma were themes underpinning behaviours.<br>Conclusions<br>The DSME interventions were under-resourced and unstructured. Diabetes self-management knowledge was limited and associated with self-management behaviour. DSME interventions in low resource settings should be culturally tailored and incorporate sessions on mitigating financial constraints. Future studies should focus on creating structured DSME interventions suitable for resource-constrained settings.
**Order of Authors:**	Roberta Lamptey
	Mary Amoakoh-Coleman
	Babbel Djobalar
	Diederick E. Grobbee
	George Obeng Adjei

Powered by Editorial Manager® and ProduXion Manager® from Aries Systems Corporation

	Kerstin Klipstein-Grobusch
**Response to Reviewers:**	We are highly indebted to the reviewers for their time. We deeply appreciate the time they have already committed to helping us improve our paper. We have uploaded a file with our point by point response in tabular form. We provide here a summary only.<br><br>We have reviewed the entire manuscript for clarity in communicating our processes and procedures. We were meticulous in our methods and we believe our work is technically sound.<br><br>We believe our analyses have been conducted appropriately and with rigour.<br>We have reviewed the entire manuscript to ensure that the language used is suitable for a scientific paper. We have endeavoured to correct all language use errors.<br>In our original submission, we addressed these issues. Please find our itemised responses below:<br><br>1. We stated that we used a convergent parallel design, a recognised type of mixed methods design for which we provided a reference. We also depicted the design graphically with Fig 1.<br><br>To explain the method in more detail we have now added this statement :<br>“Thus, we merged the two research methods (quantitative and qualitative) to answer our research questions and achieve our study aims. In addition, the two methods converged at the point of analysing the results, and interpretating the data. Data for the quantitative study and qualitative study were collected simultaneously, in parallel. Moreover, we placed equal emphasis on qualitative and quantitative data in all aspects of the study.”<br>We believe this increases the reproducibility of our method. Thank you.<br><br>2. In the abstract we stated that “we employed inductive content analysis of informants’ experiences and context”.<br><br>In the main manuscript we explained further by stating “Data was analysed independently by RL, BB and a research assistant using an inductive thematic approach manually”-These explain the qualitative method<br><br>2.In the abstract we stated, “Financial constraints, conflicting messages, beliefs, and stigma were themes underpinning behaviours.”- These themes are our quantitative results<br><br>We then went on to describe the qualitative results in detail in the main manuscript.<br>Thank you. In our original submission, we addressed these issues. Please find our itemised responses below:<br>:<br>Type of study<br>1. A convergent parallel mixed-methods study was conducted as earlier described<br><br>Sample size<br>2. sample size: In total 425 PLD…..<br>Two managers, five healthcare professionals, two diabetes experts and 16 PLD participated in in-depth interviews. Finally, 24 PLD participated in four FGD<br><br>Sampling strategy<br>3. sampling strategy: we stated the following in our original submission in the abstract “A total enumeration was done for the cross-sectional study whilst purposive or judgemental sampling was used in selecting participants for the qualitative study”.<br><br>4.date and country of the study<br>Thank you we have now included this “January to February 2021 in Accra, Ghana”<br><br>Thank you. We have re-written the entire introduction section.

Powered by Editorial Manager® and ProduXion Manager® from Aries Systems Corporation

We have modified our title to ensure that the title, new introduction, aims, and conclusions are congruent
Thank you. In our original submission, we provided an explanation for our choice. We stated that “We employed qualitative methods to deepen our understanding (of generalizable) outcomes from the quantitative study”
Thank you. In our original submission, we addressed these issues. Please find our itemised responses below:

1.where we collected samples for the study : We stated that “The study was conducted in Korle Bu Teaching Hospital polyclinic (KBTH) and Weija Gbawe Municipal hospital (WGMH),

2. we stated that these two facilities were public primary care facilities located in Accra, Ghana.

Interviews were conducted at the study sites either in offices or large open spaces whilst observing prescribed COVID-19 protocols. Experts were interviewed virtually.
”
3. year study conducted
We also stated that “Participant recruitment and data collection occurred between January and February 2021”
4.
exclusion and inclusion criteria: We also stated that “HCP and PLD were staff and attendants at the study sites respectively. Managers were the respective heads. PLD were 18 years or older, not known to have type 1 diabetes, cognitive or psychiatric impairment and ambulant.
” This section is now labelled clearly.

Thank you. In our original submission, we addressed these issues. Please find our itemised responses below:

We stated that “a total enumeration of all eligible clients seen at both study sites from December 2020 to January 2021 was done.” Thus the sampling strategy for the qualitative section was total enumeration.

We further explained that “Trained staff called all potential participants meeting eligibility criteria and invited them to participate.” This was how we accessed the sampling frame

We also stated that “PLD were identified through convenient sampling and snowballing for the qualitative study. Managers and healthcare professionals (HCPs) were sampled purposively, and judgemental sampling were used in identifying experts”. This explains the sampling method for the qualitative study.

Thank you. In our original submission, we addressed these issues. Please find our itemised responses below:

We stated that “The study was conducted in Korle Bu Teaching Hospital polyclinic (KBTH) and Weija Gbawe Municipal hospital (WGMH), two public primary facilities located in Accra, Ghana”. Thus, the facilities were government primary care facilities. They were not referral facilities.
In our original submission, we addressed these issues. Please find our itemised responses below:
Thank you.

We mentioned in our original submission that discrepancies were resolved through dialogue.
For the qualitative study, the interviews were one-on -one and for the FGD we had more than one facilitator per group including field note takers.

Response rate
Thank you. we have now included the non-response rate “21%”

Powered by Editorial Manager® and ProduXion Manager® from Aries Systems Corporation

	Permissions<br>We had stated the following in our original submission" The head of each facility granted permission for the study after having obtained ethical clearance"<br><br>Participant recruitment<br>We stated the following in our original submission "Trained staff called all potential participants meeting eligibility criteria and invited them to participate. For each individual, three attempts were made to reach them."<br><br>In our original submission, we addressed these issues. Please find our itemised responses below:<br>Thank you.<br><br>We stated in our original submission that "The Good Reporting of a Mixed-Methods Study (GRAMMS)(5) and Consolidated Criteria for REporting Qualitative research (COREQ)(6) checklists were followed.<br>"<br>In response to the reviewer's comment, we now have added the COREQ checklist as supporting material." Thank you<br>In our original submission, we addressed these issues. Please find our itemised responses below:<br>Thank you.<br><br><br><br>We stated the following and provided details of our method<br>1."using an inductive content analysis"<br><br>2."using an inductive thematic approach manually"<br>Thank you. The following statements have now been included "Our informants were fully engaged in all phases of our study. We selected participants who could best provide answers to our research question."<br>Thank you . The following statement has been added "Some of the PLD recruited from the KBTH study site might have known RL as a staff of that facility. All other PLD involved in the study did not have any prior relationship with the data collectors. Experts and Health Care Professionals were colleagues of RL. The roles of the researchers were to facilitate the FGD and conduct the interviews"<br>Our original discussion section included 14 references 9 which were published within the last 3 years and all the 14 references were published within the last 7 years.<br><br>We have in addition significantly increased the number of references in the introduction. Thank you.<br>2<br>We tried to rephrase the sentence to accommodate this suggestion however the sentence did not read well . We have therefore maintained the original sentence as is.<br>We have increased the number of references in the introduction thank you.<br><br>The entire introduction has been re-written form clarity thank you.<br>Additional background information has been provided in the introduction for clarity. We have also replaced self-management program with self-management intervention.<br>Thank you<br>Thank you this has now been corrected by quoting a 6.5% prevalence.<br>Thank you. This entire section has been re-written<br>The title, study aim and conclusions have been re-written for clarity. Our manuscript is now more focused and congruent. Thank you<br>There is a fine line between various types of diabetes with some overlap, and often it is difficult to clinically distinguish between them. For example making a distinction between latent autoimmune diabetes in adults and type 2 diabetes or between type 1 diabetes in an adult and type 2 diabetes which is burnt out. Given that we did not do formal diagnostic testing e.g autoantibodies, c-peptide etc, we decided it was best to avoid classifying patients as type 2 diabetes.<br><br>The inclusion criteria was self-reported diabetes and we excluded those known to have type 1 diabetes. We have now included this statement in our limitation "our findings

Powered by Editorial Manager® and ProduXion Manager® from Aries Systems Corporation

	may not be generalised to people known to have type 1 diabetes"<br>Thank you we have replaced DSME program with DSME intervention throughout the manuscript..<br>Thank-you. This statement has been modified We have now specified facility based DSME interventions.<br>"Additionally, sustainability of facility-based structured DSME interventions are influenced by facility-, patient-, and provider level factors.[13]"<br>Thank you<br>We have re-written the entire concluding paragraph . Those findings are limited to the two study sites.<br>"The DSME interventions studied were under-resourced and were not structured"<br>The aim has been re-stated for clarity and the study location included in the Abstract<br>"We sought to characterise DSME interventions in two urban low-resource primary settings, and to explore diabetes self-management knowledge and behaviours of persons living with diabetes (PLD).<br>"<br><br>The entire introduction has been re-written for clarity and to improve congruency with the other sections of the manuscript thank you.<br>The aim has been re-stated at the end of the introduction<br><br>Our aim was rather to describe and characterise the existing DSME interventions<br><br>The location within the city has been stated. "KBTH is located within the Ablekumah South Metropolitan district and WGMH is located in Ga West Municipal district."<br><br>Our aim was rather to describe and characterise the existing DSME interventions. The aim has been re-written. Thank you<br><br>The abbreviations in the Figures have been corrected. Thank you.<br>The section on eligibility criteria has now been clearly labelled.<br><br>Figure 1 has been reorganised as suggested<br>Figure 2- Abbreviations have been corrected<br>Figure 3 has been re-drawn; the major theme in that circle is stigma<br>The section on quantitative analysis has been titled to maintain formatting with the subsequent section<br><br>Analysis has been changes to analyses<br><br>The number of included participants have been corrected to 425. Thank you<br><br>Table 1 has been re-formatted for clarity. The variables are now readily identifiable<br><br>T in table 2 has been capitalised<br>We have updated our funding statement . Our amended funding statement is as follows:<br>"This study was funded in part by the UMC Utrecht Global Health Support PhD program. It had no role in the study design, collection, analysis, interpretation of data, writing of the report or decision to submit the article for publication.<br>"
**Additional Information:**	
**Question**	**Response**
**Financial Disclosure**<br><br>Enter a financial disclosure statement that describes the sources of funding for the work included in this submission. Review	The author(s) received no specific funding for this work.

the submission guidelines for detailed requirements. View published research articles from *PLOS ONE* for specific examples.<br><br>This statement is required for submission and **will appear in the published article** if the submission is accepted. Please make sure it is accurate.<br><br>**Unfunded studies**<br>Enter: *The author(s) received no specific funding for this work.*<br><br>**Funded studies**<br>Enter a statement with the following details:<br>• Initials of the authors who received each award<br>• Grant numbers awarded to each author<br>• The full name of each funder<br>• URL of each funder website<br>• Did the sponsors or funders play any role in the study design, data collection and analysis, decision to publish, or preparation of the manuscript?<br>• **NO** - Include this sentence at the end of your statement: *The funders had no role in study design, data collection and analysis, decision to publish, or preparation of the manuscript.*<br>• **YES** - Specify the role(s) played.<br><br>* typeset	
**Competing Interests**<br><br>Use the instructions below to enter a competing interest statement for this submission. On behalf of all authors, disclose any competing interests that could be perceived to bias this work—acknowledging all financial support and any other relevant financial or non-financial competing interests.<br><br>This statement is required for submission and **will appear in the published article** if the submission is accepted. Please make sure it is accurate and that any funding sources listed in your Funding Information later in the submission form are also	The authors have declared that no competing interests exist.

declared in your Financial Disclosure statement.<br><br>View published research articles from *PLOS ONE* for specific examples.<br><br>**NO authors have competing interests**<br><br>Enter: *The authors have declared that no competing interests exist.*<br><br>**Authors with competing interests**<br><br>Enter competing interest details beginning with this statement:<br><br>*I have read the journal's policy and the authors of this manuscript have the following competing interests: [insert competing interests here]*<br><br>* typeset	
**Ethics Statement**<br><br>Enter an ethics statement for this submission. This statement is required if the study involved:<br><br>• Human participants<br>• Human specimens or tissue<br>• Vertebrate animals or cephalopods<br>• Vertebrate embryos or tissues<br>• Field research<br><br>Write "N/A" if the submission does not require an ethics statement.<br><br>General guidance is provided below. Consult the submission guidelines for detailed instructions. **Make sure that all information entered here is included in the Methods section of the manuscript.**	Ethical approval was granted by the Institutional Review Board of KBTH (STC/IRB/000175/2020) and the Ethics Review Committee of the Ghana Health Service (GHS-ERC 05/10/20).

Powered by Editorial Manager® and ProduXion Manager® from Aries Systems Corporation

**Format for specific study types**<br><br>**Human Subject Research (involving human participants and/or tissue)**<br>• Give the name of the institutional review board or ethics committee that approved the study<br>• Include the approval number and/or a statement indicating approval of this research<br>• Indicate the form of consent obtained (written/oral) or the reason that consent was not obtained (e.g. the data were analyzed anonymously)<br><br>**Animal Research (involving vertebrate animals, embryos or tissues)**<br>• Provide the name of the Institutional Animal Care and Use Committee (IACUC) or other relevant ethics board that reviewed the study protocol, and indicate whether they approved this research or granted a formal waiver of ethical approval<br>• Include an approval number if one was obtained<br>• If the study involved *non-human primates*, add *additional details* about animal welfare and steps taken to ameliorate suffering<br>• If anesthesia, euthanasia, or any kind of animal sacrifice is part of the study, include briefly which substances and/or methods were applied<br><br>**Field Research**<br><br>Include the following details if this study involves the collection of plant, animal, or other materials from a natural setting:<br>• Field permit number<br>• Name of the institution or relevant body that granted permission	
**Data Availability**<br><br>Authors are required to make all data underlying the findings described fully available, without restriction, and from the time of publication. PLOS allows rare exceptions to address legal and ethical concerns. See the PLOS Data Policy and FAQ for detailed information.	Yes - all data are fully available without restriction

A Data Availability Statement describing where the data can be found is required at submission. Your answers to this question constitute the Data Availability Statement and **will be published in the article**, if accepted.<br><br>**Important:** Stating 'data available on request from the author' is not sufficient. If your data are only available upon request, select 'No' for the first question and explain your exceptional situation in the text box.<br><br>Do the authors confirm that all data underlying the findings described in their manuscript are fully available without restriction?	
**Describe where the data may be found in full sentences. If you are copying our sample text, replace any instances of XXX with the appropriate details.**<br><br>• If the data are **held or will be held in a public repository**, include URLs, accession numbers or DOIs. If this information will only be available after acceptance, indicate this by ticking the box below. For example: *All XXX files are available from the XXX database (accession number(s) XXX, XXX.*).<br>• If the data are all contained **within the manuscript and/or Supporting Information files**, enter the following: *All relevant data are within the manuscript and its Supporting Information files.*<br>• If neither of these applies but you are able to provide **details of access elsewhere**, with or without limitations, please do so. For example:<br><br>*Data cannot be shared publicly because of [XXX]. Data are available from the XXX Institutional Data Access / Ethics Committee (contact via XXX) for researchers who meet the criteria for access to confidential data.*<br><br>*The data underlying the results presented in the study are available from (include the name of the third party*	Data is has been uploaded and is available.

Powered by Editorial Manager® and ProduXion Manager® from Aries Systems Corporation

and contact information or URL).<br>• This text is appropriate if the data are owned by a third party and authors do not have permission to share the data.<br><br>* typeset	
Additional data availability information:	Tick here if your circumstances are not covered by the questions above and you need the journal's help to make your data available.

Powered by Editorial Manager® and ProduXion Manager® from Aries Systems Corporation

Manuscript

Click here to access/download;Manuscript;15-11 Lamptey et al no boldened sections .docx

Diabetes self-management education interventions and self-management in low-resource settings; a mixed methods study

Running title: Structured? No; Literate? No; Pamphlets? Yes!; DSME in resource-constrained settings

Roberta Lamptey[1,2,3*], Mary Amoakoh-Coleman[3,4], Babbel Djobalar[5], Diederick E. Grobbee[3], George Obeng Adjei[6,7], Kerstin Klipstein-Grobusch[3,8]

[1]Polyclinic/ Family Medicine Department, Korle Bu Teaching Hospital, Accra, Ghana.

[2]Department of Community Health, University of Ghana Medical School, Accra, Ghana.

[3]Julius Global Health, Julius Center for Health Sciences and Primary Care, University Medical Center Utrecht, Utrecht University, Utrecht, The Netherlands.

[4]Department of Epidemiology, Noguchi Memorial Institute for Medical Research, University of Ghana, Accra, Ghana.

[5]Internal Medicine Department, Korle Bu Teaching Hospital, Accra, Ghana.

[6]Centre for Tropical Clinical Pharmacology and Therapeutics, University of Ghana Medical School, Accra, Ghana

[7]Office of Research Innovation and Development, University of Ghana, Legon, Ghana

[8]Division of Epidemiology and Biostatistics, School of Public Health, Faculty of Health Sciences, University of the Witwatersrand, Johannesburg, South Africa.

[*]Corresponding author:

Email: r.lamptey@umcutrecht.nl

Abstract

Introduction

Diabetes is largely a self-managed disease and thus care outcomes are closely linked to self-management behaviours. However, structured self-management education (DSME) interventions are largely unavailable in Africa.

Aim

We sought to characterise DSME interventions in two urban low-resource primary settings, and to explore diabetes self-management knowledge and behaviours of persons living with diabetes (PLD).

Research design and Methods

A convergent parallel mixed-methods study was conducted between January to February 2021 in Accra, Ghana. A total enumeration in addition to consecutive sampling was done for the cross-sectional study whilst purposive or judgemental sampling was used in selecting participants for the qualitative study. Multivariable regression models were used to study the association between diabetes self-management knowledge and behaviours. We employed inductive content analysis of informants' experiences and context to complement the quantitative findings.

Results

In total 425 PLD (70.1 % (n=298) females, mean age 58 years (SD 12), mean blood glucose 9.4 mmol/l (SD 6.4)) participated in the quantitative study. Two managers, five professionals, two diabetes experts and 16 PLD participated in in-depth interviews. Finally, 24 PLD were involved in four focus group discussions.

Median diabetes self-management knowledge score was 40 % ((IQR 20-60). Every 1 unit increase in diabetes self-management knowledge was associated with increased scores on diet (5%;[95% CI: 2%-9%, $p<0.05$]), exercise (5%; [95% CI:2%-8%, $p<0.05$]) and glucose monitoring (4%;[95% CI:2%-5%, $p<0.05$]) domains of the diabetes self-care activities scale.

The DSME interventions studied were unstructured and limited by resources. Financial constraints, conflicting messages, beliefs, and stigma were themes underpinning behaviours.

Conclusions

The DSME interventions studied were under-resourced and unstructured. Diabetes self-management knowledge was limited and associated with self-management behaviour. DSME interventions in low resource settings should be culturally tailored and incorporate sessions on mitigating financial constraints. Future studies should focus on creating structured DSME interventions suitable for resource-constrained settings.

Key words

Diabetes, self-management, self-care, education, health resources, Ghana

Introduction

Globally 536 million people live with diabetes, and this number is projected to rise to 784 million by 2045.[1] Eighty percent of these half a billion people live in low- and middle-income countries like Ghana.[1] Diabetes is a long-standing leading cause of morbidity and mortality[2] in Ghana and among adults the prevalence is 6.5%.[3]

Diabetes self-management education (DSME), being a bedrock of optimal diabetes care can effectively improve glycaemic control and ameliorate the disease burden. [4, 5] DSME involves equipping patients with knowledge for self-management and several models for DSME interventions exist.[4, 6] Characteristics of DSME interventions include duration, cultural and linguistic tailoring, theoretical underpinnings, structure/ curriculum, mode of delivery, instructor characteristics, and intensity.[6, 7] Examples of theories which have been studied in relation to diabetes self-management include the social cognitive theory and empowerment theory.[7, 8] It is uncertain which of these characteristics of DSME interventions account for effectiveness in improving glycaemic control and care outcomes.[9]

Ryan et al reported an improvement in glycaemic control, specifically a difference in mean HbA1c, following a 6-month DSME intervention among a predominantly black population. They also found significant improvements in knowledge in glucose monitoring, nutrition, complications, and management of diabetes.[10] Similarly, a randomised control trial comparing a culturally tailored DSME intervention in African-Americans to usual care reported significant reductions in HbA1c in the intervention arm at 6 months. However, at 12 and 18 months respectively, this difference was lost.[11] A DSME intervention trial among African Americans which emphasised patient empowerment theory reported significant improvements in self-care behaviours, quality of life and insulin use even after 2 years.[12] Cunningham et al conducted a systematic review and meta-analysis of DSME intervention trials conducted exclusively African Americans. Contrary to the findings Ryan et al and Lynch et al, Cunningham et al reported a non-significant difference in mean HbA1c and no improvements in quality of life (QoL) between DSME intervention groups and usual care.[6]

In Africa DSME interventions are not widely available and studies on their effectiveness have likewise yielded conflicting results. An audit of interventions in S. Africa found 27 DSME interventions with five of these interventions offering structured education and the rest offering ad hoc education. Surprisingly, none of the interventions audited had guidelines specifically dedicated to DSME.[13] Additionally, sustainability of facility-based structured DSME interventions is influenced by facility-, patient-, and provider level factors.[14]

This limited availability of structured interventions in Africa, in particular, have consistently been reported in the literature.[15, 16] Likewise, the evidence on effectiveness of structured DSME interventions in Africa is sparse and inconclusive.[15, 17] Gathu et al conducted an RCT among 140 adults with diabetes attending a Family Medicine clinic in Kenya and reported no significant difference in mean A1c between groups. Gathu et al compared DSME delivered by certified diabetes educators to comprehensive care delivered by Family Physicians.[18] In contrast, an RCT comparing intensive structured DSME to conventional education in a facility in Nigeria showed a significant reduction in mean A1c at 6mo in the intervention arm.[19] To date, there are no structured DSME interventions in Ghana.

Structured DSME interventions for low-resource settings should be tailor-made for such settings. Such DSME interventions should take into consideration patient-, provider- and facility-level factors. Using a mixed methods design, we therefore sought to characterise DSME interventions in two urban low-

resource primary settings, and to explore the (diabetes self-management) knowledge, and behaviours of persons living with diabetes (PLD).

2.Methods

2.1. Design

A convergent parallel design[20] with triangulation was used; enabling collection of complementary data (quantitative and qualitative) concurrently (Fig 1). Thus, we merged the two research methods (quantitative and qualitative) to achieve our study aims. Data for the quantitative study and qualitative study were collected simultaneously, in parallel. Beyond data collection, the two methods converged at the point of analysing our results and interpretating our data. Specifically, we employed qualitative methods to deepen our understanding (of generalizable) outcomes from the quantitative study. In all the various aspects of the study, we placed equal emphasis on qualitative and quantitative data. Good Reporting of a Mixed-Methods Study (GRAMMS)[21] and Consolidated Criteria for Reporting Qualitative research (COREQ)[22] checklists were followed.

2.2. Setting

The study was conducted at the Korle Bu Teaching Hospital polyclinic (KBTH) and Weija Gbawe Municipal hospital (WGMH), two public primary facilities located in the city of Accra, Ghana. KBTH is located within the Ablekumah South Metropolitan district and WGMH is located in Ga West Municipal district. We conducted one-on-one interviews and held focus group discussions with PLD in large open spaces at the study sites; Managers were also interviewed in-person on-site. Prescribed COVID-19 protocols were observed at all times. Experts were however, interviewed virtually.

2.3.Participant identification, study size and sampling

Participant recruitment and data collection occurred between January and February 2021. Using attendance records, a total enumeration of all eligible clients seen at both study sites from December 2020 to January 2021 was done. These dates formed the frame and we included everyone within the frame, who met the eligibility criteria. The attendance records of each study site were used in retrieving the relevant information on potential participants. Trained staff called all potential participants meeting eligibility criteria and invited them to participate. For each individual, three attempts were made to reach them. Interested participants were given appointments for a screening visit at the study sites and to undergo study procedures. Participants received reimbursement for travel costs and time. On average, each focus group discussion (FGD) lasted about an hour.

We assumed a 50% prevalence of diabetes self-management knowledge and 10% non-response rate. [23, 24] The level of significance was set at 5%. A sample size of 425 PLD was therefore required for the cross-sectional study. Recruitment for in-depth interviews (IDI) continued until saturation was reached and no new themes emerged.

PLD were identified through convenient sampling and snowballing for the qualitative study. Managers and healthcare professionals (HCPs) were sampled purposively, and judgemental sampling were used in identifying experts.

Fig 1. Convergent parallel mixed methods study design.

Abbreviations: IDI- in-depth interview FGD-Focus Group Discussion HCP- healthcare professional EM- experts and managers

Eligibility criteria for PLD, HCP, managers and experts

Participants had to meet all the following eligibility criteria and none of the exclusion criteria to be included. Experts were nationally recognised diabetologists. HCP and PLD were staff and attendants at the study sites respectively. Managers were the respective heads. PLD were 18 years or older and ambulant at the time of recruitment. People known to have type 1 diabetes, or cognitive or psychiatric impairment were excluded.

2.4. Instrument development

As we anticipated heterogeneity in responses, because of the case-mix variation, we developed semi-structured interview guides to guide all interviews. RL and MAC, who both understand the local culture and norms, developed and refined these interview guides. The questions were informed by results of a literature review of DSME in low-resource settings conducted by RL. Participant information guides on the purpose and methods of the study and anonymity was developed by RL and reviewed by MAC and KKG.

2.5. Data collection

The study was conducted in line with the principles of the Declaration of Helsinki.[25] Prior to any study procedures, each participant provided written informed consent. Participants who consented to take part in FGD, also signed non-disclosure statements. These statements were an assurance that information divulged by participants during the FGD would remain within the group and not shared outside the group. Since the sessions were audio taped and transcribed, participants were assigned codes names. Participants were referred to by their code names rather than their real names to maintain their confidentiality during the FGDs. Access to each facility was granted by their respective heads.

2.5.1. Quantitative data collection

Diabetes self-management knowledge of PLD, the primary outcome variable, was measured on the spoken knowledge in low literacy persons with diabetes scale (SKILLD).[26] SKILLD is a 10-item questionnaire with each option giving a score of either 0(0%) or 10(100%). Higher scores indicate better diabetes self-management knowledge.

The variables which were modelled as explanatory variables were anthropometric measures, sitting blood pressure, duration of diabetes, insulin use, random blood glucose ,sex, family history of diabetes, income, educational level, occupation and the summary of diabetes self-care activities scores (SDSCA).[27]

Measurement procedures

We scrupulously followed standard recommended procedures for all measurements.[28-30] We used StatStrip Xpress glucometer(Onetouch, Taiwan) to measure random blood glucose[29], and Omron

M7 (Omron, Japan) to measure sitting blood pressure[28]. Omron digital scale, stadiometer, and inelastic tape measure were used to take anthropometric measurements.[30]

Duration of diabetes, insulin use, sex, family history of diabetes, income, educational level, and occupation were captured with a general questionnaire. The SKILLD and SDSCA instruments were interviewer administered.

Fig 2.Qualitative data collection procedures and number of informants.

Abbreviations: KBTH-Korle Bu Teaching Hospital; WGMH-Weija Gbawe Municipal Hospital IDI- in-depth interview FGD-Focus Group Discussion DM-duration of diabetes < less than > greater than yrs- years HCP-Health care professional PLD- person living with diabetes

2.5.2. Qualitative data collection

Fig 2 depicts the informants and qualitative procedures undertaken. RL and BB either conducted or coordinated the IDI and FGD. Interviews were conducted in in English, Twi, or Ga. Responses were audio-recorded digitally and handwritten field notes were taken. Some of the PLD recruited from the KBTH study site might have known RL as a staff of that facility. All other PLD involved in the study did not have any prior relationship with the data collectors. Experts and Health Care Professionals were colleagues of RL. The roles of the researchers were to facilitate the FGD and conduct the interviews.

2.6. Data management and analysis

2.6.1. Quantitative analysis

Total SKILLD score (knowledge) was analysed both as a continuous and categorical variable. The individual SKILLD items were dichotomised into correct and incorrect responses and summarised using counts (percentage).

To test the strength of the association between the total SKILLD score and SDSCA sub-domains, the Pearson's correlation was employed. The appropriate regression tests involving ordinary least squares regression or quantiles regression were performed to assess the association between total SKILLD score) and clinically relevant variables. All analyses were conducted with Stata v16.1. Statistical significance was set at a two-sided p-value < 0.05. REDCap data management system was used for data capture.

2.6.2. Qualitative analysis

Data was analysed independently by RL, BB and a research assistant using an inductive thematic approach manually. Audio-recordings were transcribed verbatim. Transcription, initial coding, and thematic analysis were done manually concurrently with data collection. We extracted both latent and manifest content. Transcripts were line searched for recurring words and phrases. Concepts were then used to generate initial codes and further expanded by applying the codes to additional transcripts (open coding). Sub-themes were identified by reviewing the data for repeating patterns in participant's responses. Sub-themes were merged into themes, ensuring themes closely described original content of transcripts. Emerging themes were categorized and compared across the various (informants) groups

using colour coded comparative charts. Direct quotes were extracted. Our informants were fully engaged in all phases of our study. We selected participants who could best provide answers to our research question. Data saturation was reached when no new themes emerged. Subsequently RL used Nvivo (released March 2020) to organise the data.

MAC reviewed the themes against the final organisation of the data to ensure that there was agreement in the data collected and its final presentation. Discrepancies and suggestions for review were resolved through dialogue.

Rigour

Data, informant, and investigator triangulation was used to ensure rigor and comprehension of concepts. The transcripts and subsequently thematic analysis were shared with informants to check for accuracy and to provide feedback. Team meetings with co-investigators experienced in qualitative methods enhance credibility of the data. Procedures have been described to allow replicability. Use of Nvivo improves transparency and reliability of the coding. Concurrent collection of quantitative and qualitative data improve internal validity.

2.7.Ethical approval

Ethical approval was granted by the Institutional Review Board of KBTH (STC/IRB/000175/2020) and the Ethics Review Committee of the Ghana Health Service (GHS-ERC 05/10/20). The head of each facility granted permission for the study after ethical clearance had been obtained.

3. Results

The quantitative results are summarised in tables and the qualitative results are presented by themes. All the quantitative results are presented first followed by the qualitative results.

3.1.Quantitative results

3.1.1.Participant’s flow and baseline characteristics

In total, 1202 participants out of 1735 potentially eligible clients were not included. Reasons for this were as follows: 54 participants had travelled (zero from WGMH), 1029 were unreachable by telephone (544 from WGMH), 95 declined (one from WGMH), 25 were dead (one from WGMM). As 112 out of 533 eligible participants invited failed to report, four additional participants (0 from WGMH) were consecutively sampled. Finally, 425 participants were included in the analysis.

Participants’ baseline socio-demographic and clinical characteristics are shown in Table 1. Additionally, the mean body weight was 98kg (SD 16). The mean waist circumference for males was 94 cm (SD 16) and for females it was 98 cm (SD 16). The mean systolic and diastolic blood pressure were 133 mmHg (SD 21) and 81 mmHg (SD 12) respectively. The mean random blood glucose was 9.4 mmol/l (SD 6.4) mmol/l.

Table 1. Descriptive (socio-demographic and clinical) characteristics of participants

Variable	Frequency	Percentage
Age(N=425)		
≤39	26	6
40-49	77	18
50-59	132	31
60-69	120	28
70+	70	17
Mean (SD)	581(SD 12)	
Sex (N=425)		
Female	298	70
Male	127	30
Educational level (N=425)		
None	52	12
Primary and middle	194	46
Secondary and vocational	118	27
Tertiary	58	14
Other	3	0.7
Marital Status (N=425)		
Married	245	58
Never married	24	5.7
Living together	1	0.2
Widowed	96	23
Divorced	59	14
Occupation (N=425)		
Professionals with university degrees	36	8.5
Professionals without university degree	30	7
Clerks, motor vehicle drivers, mechanic	89	21
Cooks, barbers, domestic staff, gas staff	36	8.5
Labourers and petty traders	86	20
Apprentices, educated youth, unemployed	148	35

Abbreviations; SD =Standard Deviation N=number of observations

Table 1 (continued). Descriptive (socio-demographic and clinical) characteristics of participants

Variable	Frequency	Percentage
Ethnicity (N=425)		
Akan	206	49
Ga/Adangbe	124	29
Ewe	53	13
Other	40	9.5
Religion (N=425)		
Christian	380	89
Islam	42	9.9
Other	3	0.7
Size of your household (N=412)		
1-2	91	22.09
3-4	136	33
5-6	116	28
6+	69	17
Min-Max	1-27	
Mean (SD)	5(3)	
Additional sources of income (N=417)		
No	342	82
Yes	75	18
Years of diabetes illness (N=416)		
≤1	48	12
2-3	95	23
4-9	138	33
10+	135	33
Min-Max	<1-45	
Mean (SD)	7.7 (0.3)	
Family history of diabetes (N=418)		
No	179	43
Yes	239	57
Have any device for checking blood sugar at home (N=418)		
No	252	60
Yes	166	40

Abbreviations; SD =Standard Deviation N=number of observations

3.1.2. Diabetes self-management knowledge among PLD

The median SKILLD score was 40 %(IQR 20-60). The results of the individual SKILLD items revealed significant deficits in diabetes self-management knowledge. Only 13 (3%) participants knew the normal HbA1c range and 162 (39%) knew the normal fasting glucose range. In total, 208 (50%) and 196 (40%) knew the signs of hyperglycaemia and hypoglycaemia, respectively. Only 227 (54%) knew how to treat hypoglycaemia. The importance of foot care was known by 135 (32%) and only126 (30%) participants knew the recommended frequency for foot examinations. The frequency of eye examinations and exercise was known by 176 (42%) and 199 (48%) respectively. Finally, 247 (59%) participants knew the long-term complications of diabetes.

3.1.3. Factors associated with diabetes self-management knowledge

There was no association between SKILLD score and any of the baseline socio-demographic and clinical variables.

3.1.4. Association between diabetes self-management knowledge and self-management behaviour

Pairwise corelations showed that SKILLD score was positively correlated with behaviour (SDSCA). The correlation coefficient was 0.22 ($p<0.01$) for diet, 0.19 ($p<0.01$) for medication, 0.14 for exercise ($p<0.05$), 0.39 ($p<0.01$) for glucose testing and 0.38 ($p<0.01$) for foot care.

3.1.5. Influence of diabetes-self-management knowledge (SKILLD) on Diabetes Self-Care Activities Measure(SDSCA) sub-domains

The effect of total SKILLD on self-management behaviours (SDSCA sub-domains), adjusted for age, education, diabetes duration, family history of diabetes and ownership of a glucometer is displayed in table 2.

Table 2. Influence of Knowledge (spoken language in Low Literacy in Diabetes scale) on Diabetes Self-Care Activities Measure sub-domains

Variable	Diabetes Self-Care Activities Measures				
	OLS		Quantile regression		
	Diet	Medication	Exercise	Blood testing	Foot
	aβ[95%CI]	aβ[95%CI]	aβ[95%CI]	aβ[95%CI]	aβ[95%CI]
SKILLED Knowledge	0.05[0.02-0.09]**	0.01[0.002-0.02]*	0.05[0.02-0.08]**	0.04[0.02-0.05]***	0.02[-0.02-0.05]
Age group					
≤39					
40-49	1.55[-2.39-5.48]	-0.73[-1.99-0.53]	1.00[-1.27-3.27]	1.07[-0.83-2.97]	0.33[-2.55-3.22]
50-59	1.21[-2.57-4.99]	0.37[-0.77-1.52]	2.00[-0.49-4.49]	0.93[-0.96-2.82]	0.17[-2.47-2.81]
60-69	1.03[-2.75-4.82]	0.20[-0.96-1.35]	1.00[-1.09-3.09]	1.07[-0.83-2.97]	0.33[-2.27-2.93]
70+	1.62[-2.46-5.70]	-0.03[-1.25-1.19]	0.50[-1.60-2.61]	2.07[-0.88-3.02]	0.33[-2.36-3.03]
Educational level					
None					
Primary	2.06[-0.93-5.05]	-0.96[-1.69- -0.24]**	1.22[-1.98-5.98]	-0.28[-0.94-0.37]	-0.17[-1.57-1.24]
Middle	1.77[-0.90-4.45]	-1.02[-1.59 -0.45]***	-0.50[-2.33-1.33]	-0.50[-1.05-0.05]	-0.17[-1.46-1.13]
Secondary	3.19[0.33-6.04]*	-1.39[--2.07- -0.71]***	2.50[-0.20-5.20]	0.07[-1.32-1.46]	0.17[-1.48-1.81]
Vocational	2.97[-2.19-4.83]	-1.28[-2.02- -0.36]**	-2.22[-3.32-6.32]	0.78[-2.57-4.14]	0.17[-2.97-3.31]
Tertiary	1.21[-2.19-4.62]	-1.24[-1.98- -0.50]***	0.50[-2.16-3.16]	2.86[0.81-4.90]	1.00[-1.36-3.36]
Other	-2.54[-11.8-6.75]	0.08[-0.76-0.92]	10.0[-17.2-37.2]	7.07[2.85-11.3]***	7.00[1.98-12.0]**
Years of diabetes illness					
≤1					
2-3	0.38[-2.45-3.21]	0.99[-0.01-1.99]	2.66[-2.18-3.18]	-0.34[-1.01-0.29]	-0.17[-1.48-1.15]
4-9	0.34[-2.41-3.09]	0.93[-0.01-1.89]	3.11[-2.45-6.45]	-0.50[-1.15-0.15]	-0.33[-1.81-1.14]
10+	0.85[-1.98-3.68]	1.25[0.30-2.21]	0.50[-1.85-2.85]	-0.35[-1.19-0.48]	-0.17[-1.83-1.50]
Family history of diabetes					
No					
Yes	-1.06[-2.69-0.57]	0.13[-0.31-0.58]	-0.50[-1.92-0.92]	0.00[-0.48-0.49]	-5.55[-1.00-5.99]
Device for checking blood sugar					
No					
Yes	2.34[0.60-4.08]**	0.61[0.20-1.03]**	-1.00[-2.38-0.39]	1.00[0.32-1.67]**	0.17[-0.94-1.27]

NOTE: Abbreviation: SKILLED= Spoken Language in Low Literacy in Diabetes; OLS-ordinary least squares regression; aβ= adjusted Coefficient estimate. Covariates used age, education, duration of diabetes and family history. *P*-value Notation; *** $p<0.01$, ** $p<0.05$, * $p<0.1$ type of test multiple linear regression

3.2.Qualitative results

3.2.1.Participants

Fig 2 depicts the types of informants and data gathering techniques used.

3.2.2.Emerging themes

The themes identified are displayed in Fig 3 and include health numeracy and financing, logistics and norms.

Fig 3. Thematic areas DSME needs in resource constrained settings.

i. DSME interventions

We found that PLD received DSME from nurses, doctors, and or nutritionists. The education was unstructured, didactic, group-based and delivered in-person prior to consultations. Groups typically had about 20 PLD per group and sessions lasted for about 30 minutes, on average.

We observed that varied perceptions among informants resulted in contrasting perspectives on existing DSME interventions. For example, PLD generally favoured group over individualised education, placing value on peer-to-peer learning. The consensus among PLD seemed to be that individualised education provided prior to a consultation was inadequate. They pointed out that the group sessions inadvertently provided avenues for newly diagnosed persons to draw on the experience and diabetes self-management knowledge of their peers. All patient groups interviewed, recommended that peers, together with health workers should be used as diabetes educators.

PLD described existing DSME interventions as beneficial but reported that teaching aids were not culturally or linguistically adapted.

R5 FGD KBTH –*"often the books available on diabetes have examples of foods eaten abroad"*

R4 FGD WGMH-*"….we have been given a book that teaches us how to manage diabetes. The book is normally read to me…. "*

R5 FGD WGMH*: ".....about the pamphlet. It sometimes contains foreign information which is their food and what they need to do in order to take care of themselves so I think they should be limited to our local activities"*

R3 FGD WGMH: *"……..I prefer all the teachings in a leaflet form…. Those who can't read the leaflet personally, can allow their children or friends to help them read"*

In contrast to PLD, providers and diabetes experts thought existing DSME interventions were at best parsimonious. Human resource constraints, lack of logistics, unavailability of academic courses, and a policy direction were challenges identified. Except for the doctors, none of the other participant groups were familiar with structured DSME.

The unstructured nature of existing DSME interventions meant PLD continued with self-management education classes ad-infinitum. Our informants appreciated the knowledge reinforcement.

R IDI KBTH: “…[They] *are doing their best because the doctors really educate the patients on how they can manage the diabetes themselves.”*

PLD used DSME interchangeably with health education. They recommended that churches and other community spaces and mass media communication channels be used for DSME.

Most informants preferred the existing in-person format to virtual sessions.

ii. Diabetes self-management knowledge

Knowledge on self-management was deficient and self-care practices among PLD were inadequate.

R4 FGD KBTH: “*I used to inject the insulin in the house but anytime I inject it, my sugar level rises so a doctor friend of mine advised me that the insulin should be injected in the hospital and by a doctor so for 5years now I have stop using the insulin*.”

PLDs echoed several myths as truths. Notwithstanding, PLD bemoaned the inconsistencies in nutritional recommendations.

iii. Self-management behaviours

PLD knew more about the importance of medication use, self-blood glucose testing, meal planning, exercise, and routine reviews than about foot care. None of the PLD and HCPs mentioned foot care. Contrastingly, foot care, routine investigations and eye screening were mentioned by the experts as being important components of self-management.

Several barriers to self-care, even when diabetes self-management knowledge was apparently adequate, were enumerated by all informants.

iv. Finance

Among persons with low health numeracy in resource constrained settings there’s little choice in lifestyle. Poverty is the common pathway for restricted access to information, food, care, and medication. PLD described dependence on literate relatives to access useful information contained in patient education leaflets.

PLDs and HCPs enumerated the cost constraints faced by PLD and how those influenced food consumption patterns. HCPs were empathetic and yet seemingly frustrated by the vicious cycle of high carbohydrate consumption and hyperglycaemia among PLD. PLD and HCPs both indicated that consumption of fresh produce was dependent on seasonality.

PLD described frequent stockout of medications covered by insurance. None of the PLD groups complained about costs associated with home glucose testing. The experts however noted that patient’s inability to afford home glucose monitoring was a barrier to optimising glycaemic control.

v. Norms and belief systems

Finances were not the only determinants of meal patterns. PLD voiced the conflict between their intentions and actions. They recounted the difficulty of executing planned behaviour (such as portion control). They described nutritional recommendations as a deviation from cultural norms. PLD described wanting to ‘belong’ at social gatherings. HCP and PLD alike alluded to the fact that diabetes (especially among young persons) was stigmatised.

PLD said they received conflicting messages from traditional herbal and alternative medicine practitioners, religious leaders, and HCPs. Furthermore, they expressed a belief in destiny and the existence of an external locus of control. These belief systems contributed to poor self-care.

4.Discussion

We sought to characterize DSME interventions and to explore the self-management knowledge and behaviours of persons living with diabetes. The interventions studied were unstructured, group-based and delivered in-person mostly by nurses. Self-management knowledge and behaviours were sub-optimal and influenced by conflicting messaging, financial constraints, culture, beliefs, and stigma.

Existing DSME interventions

The unstructured nature of the DSME interventions and use of group delivery methods probably reflects an attempt to increase the accessibility of DSME despite resource constraints. Building sustainability into DSME interventions for resource constrained settings, is key. The use of "non-internet" mass media to disseminate DSME interventions, as proposed by our informants might be a sustainable option. Moreover, since most of our informants found repetition of content useful, mass media channels may be well patronised. Similar to our findings, the importance of the traditional media in disseminating DSME was identified in another African study.[31] However, people living with long-standing diabetes in Iran reported that repetition of DSME content was not useful. A direct contrast to the views of informants in our study. Importantly, the population studied in Iran had significantly higher literacy levels relative to our study population and this difference may account for the disparities.[32] In Iran, health literacy has been shown to be positively correlated with health behaviours.[33]

Diabetes self-management knowledge and it's relation with self-management behaviours

Our findings of limited diabetes self-management knowledge echo those of previous studies.[34, 35] The extremely low SKILLD scores from our quantitative study reflect the depth of lack of knowledge on self-care. The themes we identified in this study provide some explanations for and elaborate on the inadequate diabetes self-management knowledge among PLD. In particular, the low literacy levels and inconsistent messaging are plausible explanations for the low SKILLD scores.

Despite the seemingly insurmountable barriers to self-care expressed by PLD, our results show that diabetes self-management knowledge is positively associated with several self-management behaviours. In congruence with our findings, a multi-centre cross-sectional study in Ghana found diabetes self-management knowledge to be a predictor of self-care: every 1 unit increase in knowledge was associated with 20 times the odds of higher SDSCA scores.[36] Although, the proportion of people with tertiary education was comparable to our study, the proportion of people with no education, was 50% higher relative to our study population.[36] Efforts at improving self-management knowledge might therefore ultimately also translate into better self-care behaviours among PLD in low-resource settings.

Our findings suggest, formal education is not associated with self-management behaviours except for adherence to medication. In contrast, Rothman et al found that having tertiary education was associated

with a 12% increase in SDSCA scores, indicating better self-care behaviours.[26] Surprisingly, a cross-sectional multi-centre study from Ethiopia, observed, that not having formal education was associated with increased odds of having good self-care behaviours (AOR = 2.6, 95% CI = 1.32-5.25).[37] This estimate of the effect of formal education on self-management behaviour could have been biased by the absence of a control group.

Diabetes self-management behaviours

Our findings of low scores across all domains of the SDSCA parallel findings from a multi-centre study in the Northern region of Ghana. [35] The socio-demographic and clinical profiles of the participants in these two studies were similar except for diabetes duration. The duration of diabetes was longer in the study by Mogre et al. [35], however, despite having had diabetes for longer, the self-management behaviours were just as sub-optimal as in our study. The low SDSCA scores from the quantitative study and the qualitative results from the IDI and FGDs both indicate poor self-management among PLD. It is plausible poor self-care behaviours are fuelled by both factors within and beyond the individual's control; particularly the financial challenges enumerated earlier. A cross-sectional study involving PLD in a specialist clinic of a tertiary teaching hospital in Nigeria also echo our findings of low scores on all domains of SDSCA.[38]

The alarmingly low knowledge scores on foot care, and correspondingly poor practice of foot care, in our study is disturbing. Our findings provide strong justification for emphasising foot care in DSME interventions. Curricula which emphasise the relation between amputations, glycaemic control, routines, and daily lifestyle choices would be beneficial. The qualitative results from our study provide further insight into the low scores in the domain of foot care and parallel findings from other sub-Saharan African countries[39] and other regions of Ghana.[35] Our findings also resonate with a qualitative facility-based study among a predominantly agricultural community.[34] Bossman et al reported deficits in diabetes self-management knowledge and self-care behaviours in the domains of nutrition, exercise, and foot care with foot care being the least known and practiced.[34] It is thus not surprising that, amputations are major causes of morbidity among PLD in Ghana and other sub-Saharan African countries.[40]

Our findings indicate a high demand for diabetes self-management information, especially, culturally tailored information on nutrition therapy albeit poor adherence to nutritional recommendations. Unfortunately, the edicts of self-care behaviours particularly in the domain of nutrition deviate from local cultural norms and this could contribute to the poor adherence. Furthermore, Unavailability of formal training in DSME for providers, could contribute to inconsistent messaging on nutritional therapy. Our findings parallel those from a study conducted in specialist clinic in Nigeria which reported confusion about nutritional recommendations, and the unacceptability of nutritional recommendations.[41]

We found that behaviour change seemed to be a hurdle that persisted despite adequate diabetes self-management knowledge. Our results suggest that our informants' capacity to modify established behaviours might be limited. Previous behaviour is a known predictor of adherence to self-care recommendations.[42] Incorporating education on behaviour change strategies may therefore be a useful addition to the existing DSME interventions.

Financial constraints

In this study, financial constraints transcend multiple aspects of diabetes self-management: adherence to self-management recommendations, keeping clinic appointments and purchasing medications. In particular, medications which were unavailable on the National Health Insurance were largely inaccessible. Likewise, for many of our informants, accessibility of vegetables was determined by their seasonality. Our findings collaborate previous findings from Ghana [43], and Benin.[44] de-Graft Aikins et al have previously shown that cost was a major and important limiting factor in several domains of self-management. [43]

Norms and belief systems

Some of our informants expressed the belief that the locus of control resides outside the individual. We found a belief in “divinity” which influenced perceptions of diabetes and diseases in general as reported widely in previous studies from Ghana[41, 43], Benin[44], Malawi and Mozambique.[31] Potentially, the local beliefs systems could adversely affect attitudes to self-care and self-care behaviours. This indicates a need to include sessions on the locus of control when designing DSME interventions for such settings.

Stigma

Hospital based DSME was more valued than community-based DSME because of diabetes-related stigma. Our finding that diabetes is stigmatised suggests that, having support persons as part of DSME interventions might be beneficial. Using peer educators may offer net-working opportunities for PLD and discussing disclosure may improve effectiveness of DSME interventions. The finding of stigma and lack of family support was also reported by Mogre et al. [45] Among Ghanaians, family non-support has been found to be negatively correlated with diabetes self-management behaviours.[46] Family support has a linear relation with self-care.[47]

Strengths and limitations

Quantitative analysis enabled us to generate valid unbiased estimates of diabetes self-management knowledge, and behaviours. The mixed methods design provided additional qualitative data and insights into the results of the quantitative study. The data was coded and analysed by researchers well accustomed to the Ghanaian culture. Data was generated from a variety of informants and study participants, managers, PLD, HCPs and experts.

The generalisability of the study to the Ghanaian population, however, is limited because the study was conducted only in two facilities within the Greater Accra region. However, the clientele of KBTH come from all over Ghana. Our findings may also not be generalised to people known to have type 1 diabetes. Furthermore, the use of consecutive sampling may limit the representativeness of our sample.

Conclusion

The DSME interventions studied were under-resourced and were not structured. Our findings indicate very limited diabetes self-management knowledge and poor adherence to self-care recommendations. Barriers to self-care included cost constraints, cultural norms, stigma and belief systems. DSME interventions should incorporate sessions on mitigating these barriers. They should be culturally tailored

and linguistically modified for people with low literacy. This may improve self-management, ultimately reducing the difficulties of PLD in resource constrained settings. Future mixed-methods cohort studies should focus on elucidating factors associated with effectives of DSME interventions in low resource settings.

Acknowledgements

We are thankful to Mr John Tetteh for assistance with running the statistical analysis.

References

1. IDF. International diabetes federation. IDF Diabetes Atlas, 10th edn Brussels, Belgium: International Diabetes Federation. 2021.

2. Owusu AY, Kushitor SB, Ofosu AA, Kushitor MK, Ayi A, Awoonor-Williams JK. Institutional mortality rate and cause of death at health facilities in Ghana between 2014 and 2018. PLoS One. 2021;16(9):e0256515. Epub 20210908. doi: 10.1371/journal.pone.0256515. PubMed PMID: 34496000; PubMed Central PMCID: PMCPMC8425528.

3. Asamoah-Boaheng M, Sarfo-Kantanka O, Tuffour AB, Eghan B, Mbanya JC. Prevalence and risk factors for diabetes mellitus among adults in Ghana: a systematic review and meta-analysis. International health. 2019;11(2):83-92.

4. Powers MA, Bardsley JK, Cypress M, Funnell MM, Harms D, Hess-Fischl A, et al. Diabetes self-management education and support in adults with type 2 diabetes: a consensus report of the American Diabetes Association, the Association of Diabetes Care & Education Specialists, the Academy of Nutrition and Dietetics, the American Academy of Family Physicians, the American Academy of PAs, the American Association of Nurse Practitioners, and the American Pharmacists Association. Diabetes Care. 2020;43(7):1636-49.

5. Chrvala CA, Sherr D, Lipman RD. Diabetes self-management education for adults with type 2 diabetes mellitus: A systematic review of the effect on glycemic control. Patient Educ Couns. 2016;99(6):926-43. Epub 2015/12/15. doi: 10.1016/j.pec.2015.11.003. PubMed PMID: 26658704.

6. Cunningham AT, Crittendon DR, White N, Mills GD, Diaz V, LaNoue MD. The effect of diabetes self-management education on HbA1c and quality of life in African-Americans: a systematic review and meta-analysis. BMC Health Serv Res. 2018;18(1):367. Epub 20180516. doi: 10.1186/s12913-018-3186-7. PubMed PMID: 29769078; PubMed Central PMCID: PMCPMC5956958.

7. Powers MA, Bardsley J, Cypress M, Duker P, Funnell MM, Fischl AH, et al. Diabetes Self-management Education and Support in Type 2 Diabetes. Diabetes Educ. 2017;43(1):40-53. doi: 10.1177/0145721716689694. PubMed PMID: 28118121.

8. Reisi M, Fazeli H, Mahmoodi M. Application of the social cognitive theory to predict self-care behavior among type 2 diabetes patients with limited health literacy. Journal of health litaracy. 2021;6(2):21-32.

9. Othman MM, Khudadad H, Dughmosh R, Furuya-Kanamori L, Abou-Samra AB, Doi SAR. Towards a better understanding of self-management interventions in type 2 diabetes: A concept analysis. Prim Care Diabetes. 2021. Epub 20210920. doi: 10.1016/j.pcd.2021.09.001. PubMed PMID: 34556438.

10. Ryan JG, Jennings T, Vittoria I, Fedders M. Short and long-term outcomes from a multisession diabetes education program targeting low-income minority patients: a six-month follow up. Clinical therapeutics. 2013;35(1):A43-A53.

11. Lynch EB, Mack L, Avery E, Wang Y, Dawar R, Richardson D, et al. Randomized trial of a lifestyle intervention for urban low-income African Americans with type 2 diabetes. J Gen Intern Med. 2019;34(7):1174-83.

12. Tang TS, Funnell MM, Noorulla S, Oh M, Brown MB. Sustaining short-term improvements over the long-term: results from a 2-year diabetes self-management support

(DSMS) intervention. Diabetes Res Clin Pract. 2012;95(1):85-92. Epub 20110827. doi: 10.1016/j.diabres.2011.04.003. PubMed PMID: 21872962; PubMed Central PMCID: PMCPMC3783218.

13. Dube L, Van den Broucke S, Dhoore W, Kalweit K, Housiaux M. An audit of diabetes self-management education programs in South Africa. Journal of public health research. 2015;4(3):jphr. 2015.581.

14. Mash R, Kroukamp R, Gaziano T, Levitt N. Cost-effectiveness of a diabetes group education program delivered by health promoters with a guiding style in underserved communities in Cape Town, South Africa. Patient Educ Couns. 2015;98(5):622-6. Epub 2015/02/03. doi: 10.1016/j.pec.2015.01.005. PubMed PMID: 25641665.

15. Lamptey R, Robben MP, Amoakoh-Coleman M, Boateng D, Grobbee DE, Davies MJ, et al. Structured diabetes self-management education and glycaemic control in low- and middle-income countries: A systematic review. Diabet Med. 2022:e14812. Epub 20220218. doi: 10.1111/dme.14812. PubMed PMID: 35179792.

16. Bekele BB, Negash S, Bogale B, Tesfaye M, Getachew D, Weldekidan F, et al. Effect of diabetes self-management education (DSME) on glycated hemoglobin (HbA1c) level among patients with T2DM: Systematic review and meta-analysis of randomized controlled trials. Diabetes Metab Syndr. 2021;15(1):177-85. Epub 20201219. doi: 10.1016/j.dsx.2020.12.030. PubMed PMID: 33360516.

17. Kumah E, Otchere G, Ankomah SE, Fusheini A, Kokuro C, Aduo-Adjei K, et al. Diabetes self-management education interventions in the WHO African Region: A scoping review. PLoS One. 2021;16(8):e0256123. Epub 2021/08/18. doi: 10.1371/journal.pone.0256123. PubMed PMID: 34403455; PubMed Central PMCID: PMCPMC8370626.

18. Gathu CW, Shabani J, Kunyiha N, Ratansi R. Effect of diabetes self-management education on glycaemic control among type 2 diabetic patients at a family medicine clinic in Kenya: A randomised controlled trial. Afr J Prim Health Care Fam Med. 2018;10(1):e1-e9. Epub 2018/11/21. doi: 10.4102/phcfm.v10i1.1762. PubMed PMID: 30456964; PubMed Central PMCID: PMCPMC6244221.

19. Essien O, Otu A, Umoh V, Enang O, Hicks JP, Walley J. Intensive Patient Education Improves Glycaemic Control in Diabetes Compared to Conventional Education: A Randomised Controlled Trial in a Nigerian Tertiary Care Hospital. PLoS One. 2017;12(1):e0168835. Epub 20170103. doi: 10.1371/journal.pone.0168835. PubMed PMID: 28045979; PubMed Central PMCID: PMCPMC5207750.

20. Klassen AC, Creswell J, Plano Clark VL, Smith KC, Meissner HI. Best practices in mixed methods for quality of life research. Qual Life Res. 2012;21(3):377-80. Epub 20120204. doi: 10.1007/s11136-012-0122-x. PubMed PMID: 22311251.

21. O'Cathain A, Murphy E, Nicholl J. The quality of mixed methods studies in health services research. J Health Serv Res Policy. 2008;13(2):92-8. doi: 10.1258/jhsrp.2007.007074. PubMed PMID: 18416914.

22. Tong A, Sainsbury P, Craig J. Consolidated criteria for reporting qualitative research (COREQ): a 32-item checklist for interviews and focus groups. Int J Qual Health Care. 2007;19(6):349-57. Epub 20070914. doi: 10.1093/intqhc/mzm042. PubMed PMID: 17872937.

23. Zowgar AM, Siddiqui MI, Alattas KM. Level of diabetes knowledge among adult patients with diabetes using diabetes knowledge test. Saudi Med J. 2018;39(2):161-8. doi: 10.15537/smj.2017.2.21343. PubMed PMID: 29436565; PubMed Central PMCID: PMCPMC5885093.

24. Muninarayana C, Balachandra G, Hiremath SG, Iyengar K, Anil NS. Prevalence and awareness regarding diabetes mellitus in rural Tamaka, Kolar. Int J Diabetes Dev Ctries. 2010;30(1):18-21. doi: 10.4103/0973-3930.60005. PubMed PMID: 20431801; PubMed Central PMCID: PMCPMC2859279.

25. World Medical A. World Medical Association Declaration of Helsinki: ethical principles for medical research involving human subjects. JAMA. 2013;310(20):2191-4. doi: 10.1001/jama.2013.281053. PubMed PMID: 24141714.

26. Rothman RL, Malone R, Bryant B, Wolfe C, Padgett P, DeWalt DA, et al. The spoken knowledge in low literacy in diabetes scale: a diabetes knowledge scale for vulnerable patients. Diabetes Educ. 2005;31(2):215-24. doi: 10.1177/0145721705275002. PubMed PMID: 15797850.

27. Toobert DJ, Hampson SE, Glasgow RE. The summary of diabetes self-care activities measure: results from 7 studies and a revised scale. Diabetes Care. 2000;23(7):943-50. doi: 10.2337/diacare.23.7.943. PubMed PMID: 10895844.

28. Umemura S, Arima H, Arima S, Asayama K, Dohi Y, Hirooka Y, et al. The Japanese Society of Hypertension guidelines for the management of hypertension (JSH 2019). Hypertension Research. 2019;42(9):1235-481.

29. Sagkal Midilli T, Ergin E, Baysal E, Ari Z. Comparison of glucose values of blood samples taken in three different Ways. Clin Nurs Res. 2019;28(4):436-55. Epub 20170706. doi: 10.1177/1054773817719379. PubMed PMID: 28679284.

30. Sellen D. Physical Status: The Use and Interpretation of Anthropometry. Report of a WHO Expert Committee. WHO Technical Report Series No. 854. Pp. 452.(WHO, Geneva, 1995.) Swiss Fr 71.00. Journal of Biosocial Science. 1998;30(1):135-44.

31. Bamuya C, Correia JC, Brady EM, Beran D, Harrington D, Damasceno A, et al. Use of the socio-ecological model to explore factors that influence the implementation of a diabetes structured education programme (EXTEND project) inLilongwe, Malawi and Maputo, Mozambique: a qualitative study. BMC Public Health. 2021;21(1):1355. Epub 20210708. doi: 10.1186/s12889-021-11338-y. PubMed PMID: 34238258; PubMed Central PMCID: PMCPMC8268266.

32. Mardanian Dehkordi L, Abdoli S. Diabetes self-management education; experience of people with diabetes. J Caring Sci. 2017;6(2):111-8. Epub 20170601. doi: 10.15171/jcs.2017.011. PubMed PMID: 28680865; PubMed Central PMCID: PMCPMC5488666.

33. Mirsamiyazdi N, Jafaripour F, Taqvaeinasab H, Masoudiyekta L, Amiri R, Komeilifar Z. The relationship between health literacy and health promoting behaviors in patients with type2 diabetes. Journal of Health Literacy. 2021;6(3):24-31.

34. Bossman IF, Dare S, Oduro BA, Baffour PK, Hinneh TK, Nally JE. Patients' knowledge of diabetes foot complications and self-management practices in Ghana: A phenomenological study. PLoS One. 2021;16(8):e0256417. Epub 20210825. doi: 10.1371/journal.pone.0256417. PubMed PMID: 34432838; PubMed Central PMCID: PMCPMC8386847.

35. Mogre V, Abanga ZO, Tzelepis F, Johnson NA, Paul C. Adherence to and factors associated with self-care behaviours in type 2 diabetes patients in Ghana. BMC Endocr Disord. 2017;17(1):20. Epub 20170324. doi: 10.1186/s12902-017-0169-3. PubMed PMID: 28340613; PubMed Central PMCID: PMCPMC5366118.

36. Afaya RA, Bam V, Azongo TB, Afaya A, Kusi-Amponsah A, Ajusiyine JM, et al. Medication adherence and self-care behaviours among patients with type 2 diabetes

mellitus in Ghana. PLoS One. 2020;15(8):e0237710. Epub 20200821. doi: 10.1371/journal.pone.0237710. PubMed PMID: 32822381; PubMed Central PMCID: PMCPMC7446850.

37. Molalign Takele G, Weharei MA, Kidanu HT, Gebrekidan KG, Gebregiorgis BG. Diabetes self-care practice and associated factors among type 2 diabetic patients in public hospitals of Tigray regional state, Ethiopia: A multicenter study. PLoS One. 2021;16(4):e0250462. Epub 20210421. doi: 10.1371/journal.pone.0250462. PubMed PMID: 33882117; PubMed Central PMCID: PMCPMC8059799.

38. Jackson IL, Onung SI, Oiwoh EP. Self-care activities, glycaemic control and health-related quality of life of patients with type 2 diabetes in a tertiary hospital in Nigeria. Diabetes Metab Syndr. 2021;15(1):137-43. Epub 20201214. doi: 10.1016/j.dsx.2020.12.027. PubMed PMID: 33340873.

39. Stephani V, Opoku D, Beran D. Self-management of diabetes in sub-Saharan Africa: A systematic review. BMC Public Health. 2018;18(1):1148. Epub 20180929. doi: 10.1186/s12889-018-6050-0. PubMed PMID: 30268115; PubMed Central PMCID: PMCPMC6162903.

40. Boateng D, Ayellah BB, Adjei DN, Agyemang C. Contribution of diabetes to amputations in sub-Sahara Africa: A systematic review and meta-analysis. Primary Care Diabetes. 2022.

41. Hushie M. Exploring the barriers and facilitators of dietary self-care for type 2 diabetes: a qualitative study in Ghana. Health Promot Perspect. 2019;9(3):223-32. Epub 20190806. doi: 10.15171/hpp.2019.31. PubMed PMID: 31508343; PubMed Central PMCID: PMCPMC6717922.

42. Jannuzzi FF, Cornelio ME, Sao-Joao TM, Gallani MC, Godin G, Rodrigues RCM. Psychosocial determinants of adherence to oral antidiabetic medication among people with type 2 diabetes. J Clin Nurs. 2020;29(5-6):909-21. Epub 20200106. doi: 10.1111/jocn.15149. PubMed PMID: 31856319.

43. de-Graft Aikins A, Awuah RB, Pera TA, Mendez M, Ogedegbe G. Explanatory models of diabetes in urban poor communities in Accra, Ghana. Ethn Health. 2015;20(4):391-408. Epub 20140722. doi: 10.1080/13557858.2014.921896. PubMed PMID: 25048822.

44. Alaofe H, Yeo S, Okechukwu A, Magrath P, Amoussa Hounkpatin W, Ehiri J, et al. Cultural considerations for the adaptation of a diabetes Self-management education program in Cotonou, Benin: Lessons learned from a qualitative study. Int J Environ Res Public Health. 2021;18(16). Epub 20210807. doi: 10.3390/ijerph18168376. PubMed PMID: 34444125; PubMed Central PMCID: PMCPMC8393923.

45. Mogre V, Johnson NA, Tzelepis F, Paul C. Barriers to diabetic self-care: A qualitative study of patients' and healthcare providers' perspectives. J Clin Nurs. 2019;28(11-12):2296-308. Epub 20190306. doi: 10.1111/jocn.14835. PubMed PMID: 30791160.

46. Amankwah-Poku M, Amoah AGB, Sefa-Dedeh A, Akpalu J. Psychosocial distress, clinical variables and self-management activities associated with type 2 diabetes: a study in Ghana. Clin Diabetes Endocrinol. 2020;6:14. Epub 20200714. doi: 10.1186/s40842-020-00102-7. PubMed PMID: 32685189; PubMed Central PMCID: PMCPMC7362489.

47. Gunggu A, Thon CC, Whye Lian C. Predictors of diabetes self-management among type 2 diabetes patients. J Diabetes Res. 2016;2016:9158943. Epub 20160803. doi: 10.1155/2016/9158943. PubMed PMID: 27563681; PubMed Central PMCID: PMCPMC4987486.

Fig 1.

Click here to access/download;Figure;Fig 1.tif

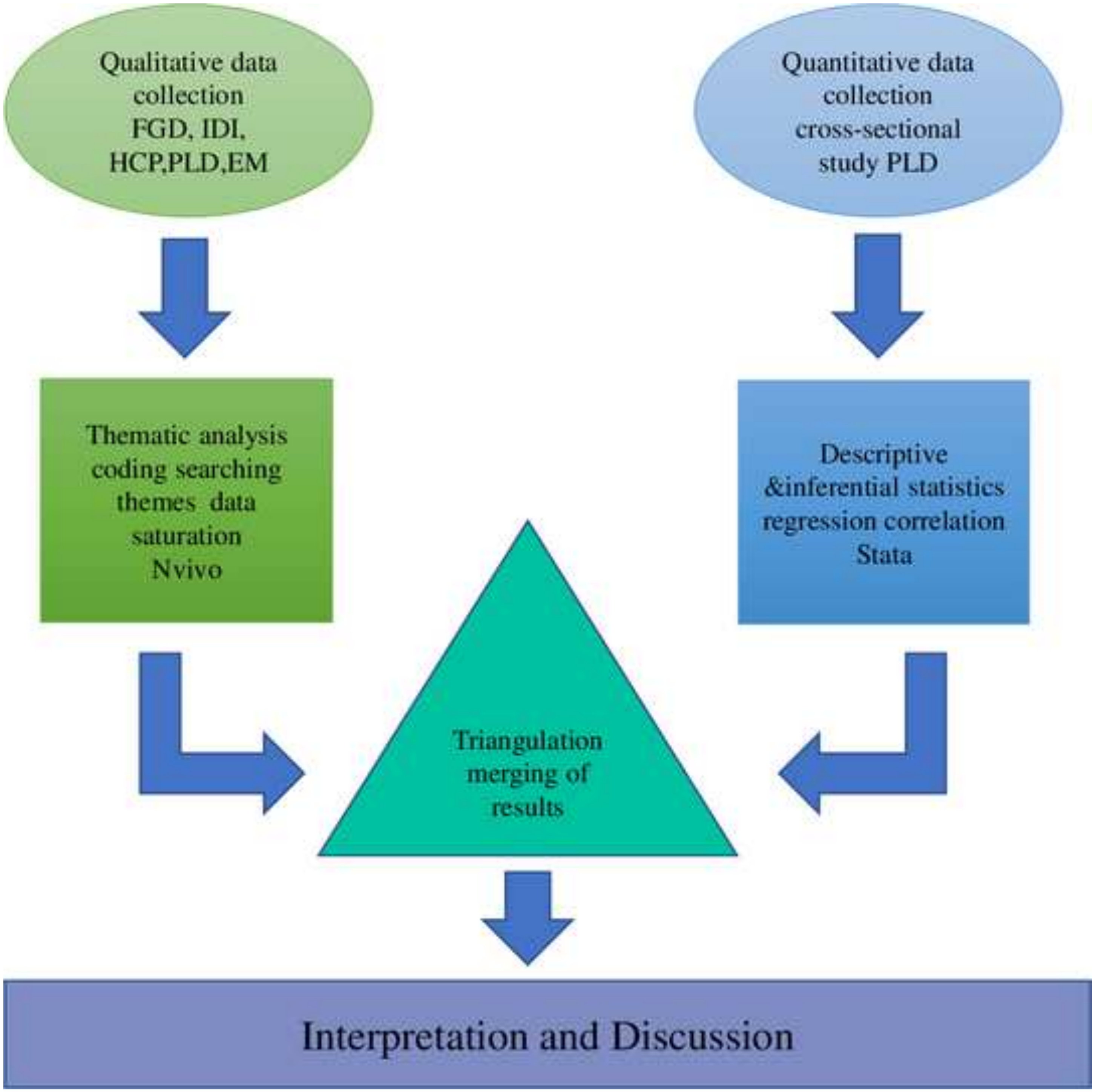

Fig 3.

Click here to access/download;Figure;Fig 3.tif

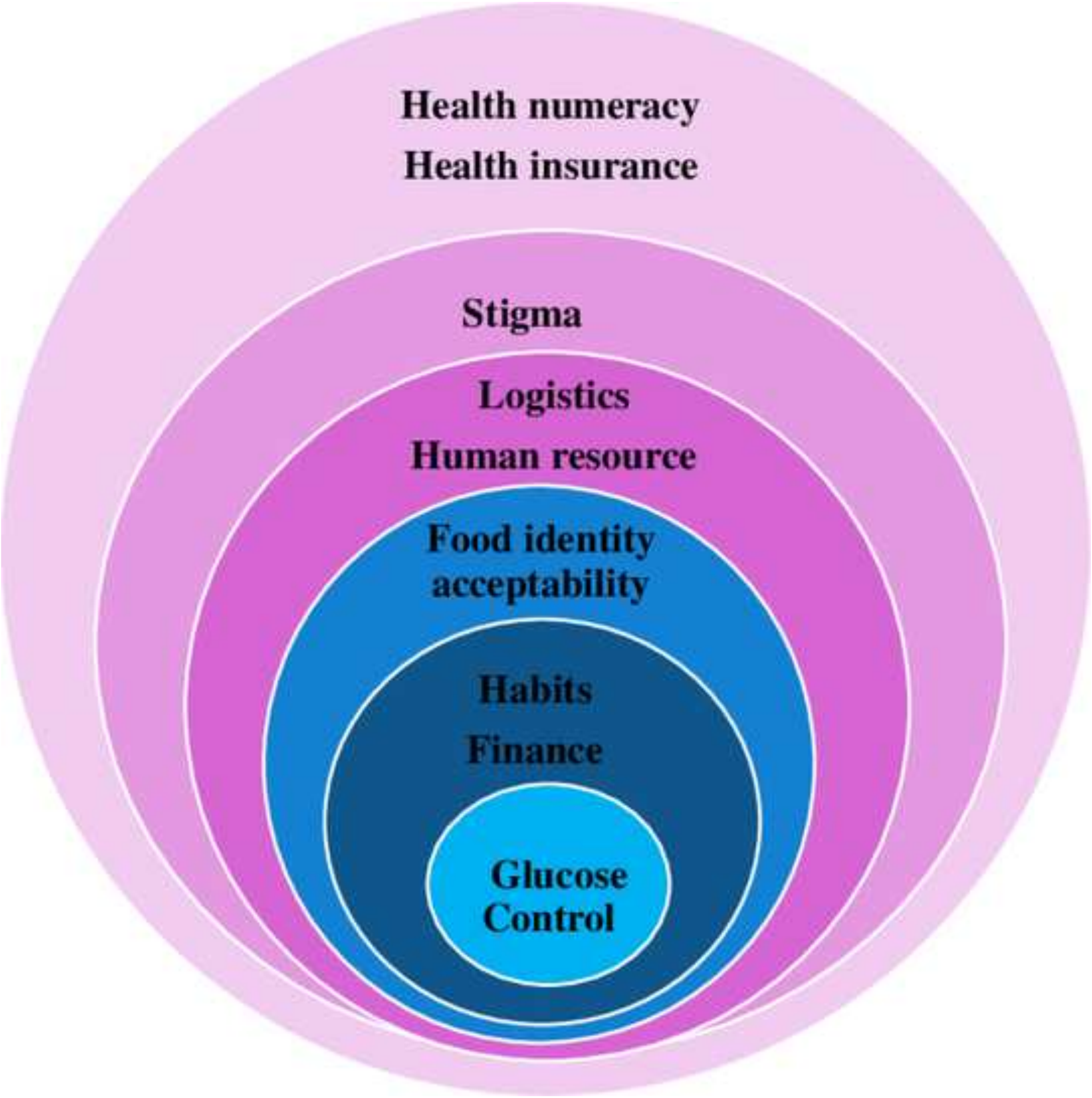

Fig 2

Click here to access/download;Figure;Lamptey et al Fig 2.tiff

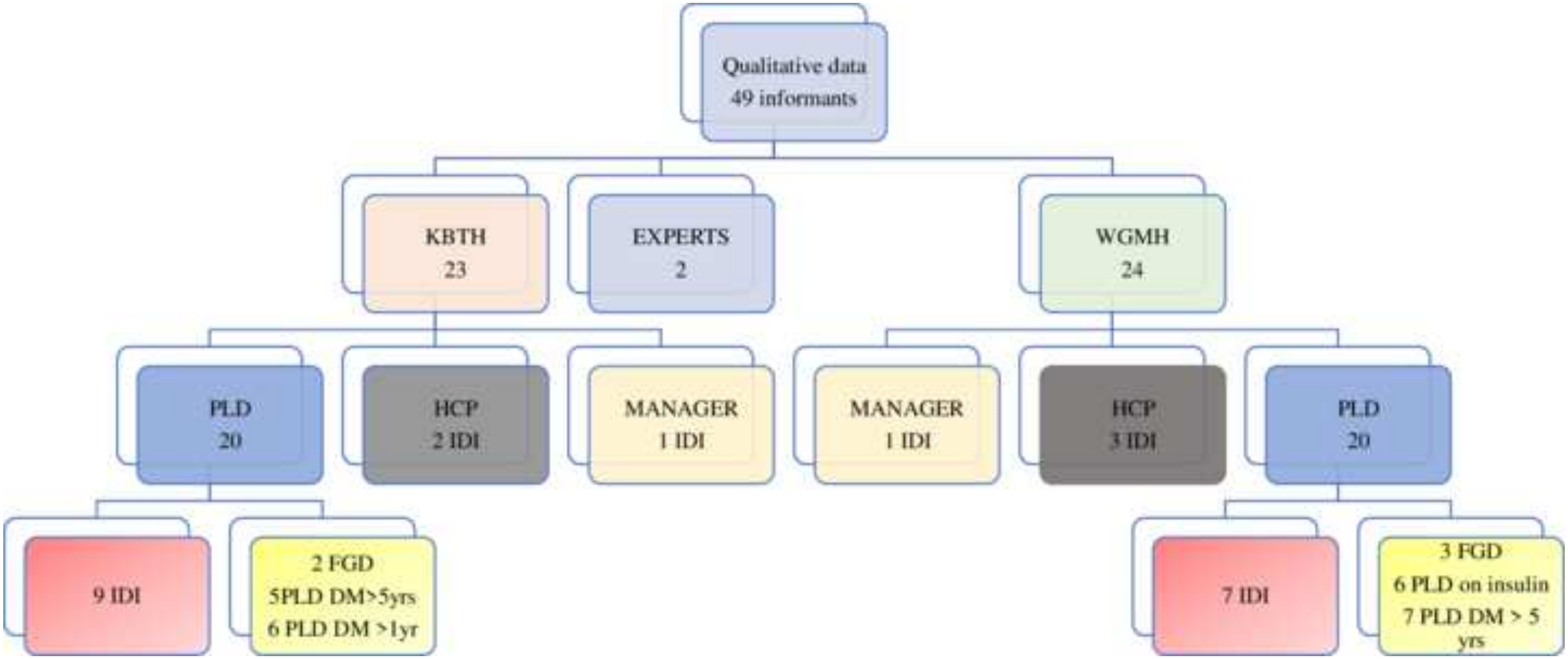

Click here to access/download
Supporting Information
Lamptey et al STROBE_checklist_cross-sectional.docx

Click here to access/download
Supporting Information
31-10 COREQ CHECKLIST.pdf

Click here to access/download

Supporting Information - Compressed/ZIP File Archive

Mixed methods DATA.qdpx

Click here to access/download
Supporting Information - Compressed/ZIP File Archive
Diabetes Data-Update-3.xlsx

Diabetes self-management education interventions and self-management in low-resource settings; a mixed methods study

Running title: Structured? No; Literate? No; Pamphlets? Yes!; DSME in resource-constrained settings

Roberta Lamptey[1,2,3*], Mary Amoakoh-Coleman[3,4], Babbel Djobalar[5], Diederick E. Grobbee[3], George Obeng Adjei[6,7], Kerstin Klipstein-Grobusch[3,8]

[1]Polyclinic/ Family Medicine Department, Korle Bu Teaching Hospital, Accra, Ghana.

[2]Department of Community Health, University of Ghana Medical School, Accra, Ghana.

[3]Julius Global Health, Julius Center for Health Sciences and Primary Care, University Medical Center Utrecht, Utrecht University, Utrecht, The Netherlands.

[4]Department of Epidemiology, Noguchi Memorial Institute for Medical Research, University of Ghana, Accra, Ghana.

[5]Internal Medicine Department, Korle Bu Teaching Hospital, Accra, Ghana.

[6]Centre for Tropical Clinical Pharmacology and Therapeutics, University of Ghana Medical School, Accra, Ghana

[7]Office of Research Innovation and Development, University of Ghana, Legon, Ghana

[8]Division of Epidemiology and Biostatistics, School of Public Health, Faculty of Health Sciences, University of the Witwatersrand, Johannesburg, South Africa.

[*]Corresponding author:

Email: r.lamptey@umcutrecht.nl

Abstract

Introduction

Diabetes is largely a self-managed disease and thus care outcomes are closely linked to self-management behaviours. However, structured self-management education (DSME) interventions are largely unavailable in Africa.

Aim

We sought to characterise DSME interventions in two urban low-resource primary settings, and to explore diabetes self-management knowledge and behaviours of persons living with diabetes (PLD).

Research design and Methods

A convergent parallel mixed-methods study was conducted between **January to February 2021 in Accra, Ghana**. A total enumeration **in addition to consecutive sampling** was done for the cross-sectional study whilst purposive or judgemental sampling was used in selecting participants for the qualitative study. Multivariable regression models were used to study the association between diabetes self-management knowledge and behaviours. We employed inductive content analysis of informants' experiences and context to complement the quantitative findings.

Results

In total 425 PLD (70.1 % (n=298) females, mean age 58 years (SD 12), mean blood glucose 9.4 mmol/l (SD 6.4)) participated in the quantitative study. Two managers, five professionals, two diabetes experts and 16 PLD participated in in-depth interviews. Finally, 24 PLD were involved in four focus group discussions.

Median diabetes self-management knowledge score was 40 % ((IQR 20-60). Every 1 unit increase in diabetes self-management knowledge was associated with increased scores on diet (5%;[95% CI: 2%-9%, p<0.05]), exercise (5%; [95% CI:2%-8%, p<0.05]) and glucose monitoring (4%;[95% CI:2%-5%, p<0.05]) domains of the diabetes self-care activities scale.

The DSME interventions studied were unstructured and limited by resources. Financial constraints, conflicting messages, beliefs, and stigma were themes underpinning behaviours.

Conclusions

The DSME interventions studied were under-resourced and unstructured. Diabetes self-management knowledge was limited and associated with self-management behaviour. DSME interventions in low resource settings should be culturally tailored and incorporate sessions on mitigating financial constraints. Future studies should focus on creating structured DSME interventions suitable for resource-constrained settings.

Key words

Diabetes, self-management, self-care, education, health resources, Ghana

Introduction

Globally 536 million people live with diabetes, and this number is projected to rise to 784 million by 2045.[1] Eighty percent of these half a billion people live in low- and middle-income countries like Ghana.[1] Diabetes is a long-standing leading cause of morbidity and mortality[2] in Ghana and among adults the prevalence is 6.5%.[3]

Diabetes self-management education (DSME), being a bedrock of optimal diabetes care can effectively improve glycaemic control and ameliorate the disease burden. [4, 5] DSME involves equipping patients with knowledge for self-management and several models for DSME interventions exist.[4, 6] Characteristics of DSME interventions include duration, cultural and linguistic tailoring, theoretical underpinnings, structure/ curriculum, mode of delivery, instructor characteristics, and intensity.[6, 7] Examples of theories which have been studied in relation to diabetes self-management include the social cognitive theory and empowerment theory.[7, 8] It is uncertain which of these characteristics of DSME interventions account for effectiveness in improving glycaemic control and care outcomes.[9]

Ryan et al reported an improvement in glycaemic control, specifically a difference in mean HbA1c, following a 6-month DSME intervention among a predominantly black population. They also found significant improvements in knowledge in glucose monitoring, nutrition, complications, and management of diabetes.[10] Similarly, a randomised control trial comparing a culturally tailored DSME intervention in African-Americans to usual care reported significant reductions in HbA1c in the intervention arm at 6 months. However, at 12 and 18 months respectively, this difference was lost.[11] A DSME intervention trial among African Americans which emphasised patient empowerment theory reported significant improvements in self-care behaviours, quality of life and insulin use even after 2 years.[12] Cunningham et al conducted a systematic review and meta-analysis of DSME intervention trials conducted exclusively African Americans. Contrary to the findings Ryan et al and Lynch et al, Cunningham et al reported a non-significant difference in mean HbA1c and no improvements in quality of life (QoL) between DSME intervention groups and usual care.[6]

In Africa DSME interventions are not widely available and studies on their effectiveness have likewise yielded conflicting results. An audit of interventions in S. Africa found 27 DSME interventions with five of these interventions offering structured education and the rest offering ad hoc education. Surprisingly, none of the interventions audited had guidelines specifically dedicated to DSME.[13] Additionally, sustainability of facility-based structured DSME interventions is influenced by facility-, patient-, and provider level factors.[14]

This limited availability of structured interventions in Africa, in particular, have consistently been reported in the literature.[15, 16] Likewise, the evidence on effectiveness of structured DSME interventions in Africa is sparse and inconclusive.[15, 17] Gathu et al conducted an RCT among 140 adults with diabetes attending a Family Medicine clinic in Kenya and reported no significant difference in mean A1c between groups. Gathu et al compared DSME delivered by certified diabetes educators to comprehensive care delivered by Family Physicians.[18] In contrast, an RCT comparing intensive structured DSME to conventional education in a facility in Nigeria showed a significant reduction in mean A1c at 6mo in the intervention arm.[19] To date, there are no structured DSME interventions in Ghana.

Structured DSME interventions for low-resource settings should be tailor-made for such settings. Such DSME interventions should take into consideration patient-, provider- and facility-level factors. Using a mixed methods design, we therefore sought to characterise DSME interventions in two urban low-resource primary settings, and to explore the (diabetes self-management) knowledge, and behaviours of persons living with diabetes (PLD).

2.Methods

2.1. Design

A convergent parallel design[20] with triangulation was used; enabling collection of complementary data (quantitative and qualitative) concurrently (Fig 1). **Thus, we merged the two research methods (quantitative and qualitative) to achieve our study aims. Data for the quantitative study and qualitative study were collected simultaneously, in parallel. Beyond data collection, the two methods converged at the point of analysing our results and interpretating our data. Specifically, we employed qualitative methods to deepen our understanding (of generalizable) outcomes from the quantitative study. In all the various aspects of the study, we placed equal emphasis on qualitative and quantitative data.** Good Reporting of a Mixed-Methods Study (GRAMMS)[21] and Consolidated Criteria for Reporting Qualitative research (COREQ)[22] checklists were followed.

2.2. Setting

The study was conducted at the Korle Bu Teaching Hospital polyclinic (KBTH) and Weija Gbawe Municipal hospital (WGMH), two public primary facilities located in the city of Accra, Ghana. **KBTH is located within the Ablekumah South Metropolitan district and WGMH is located in Ga West Municipal district.** We conducted **one-on-one** interviews and held focus group discussions with PLD in large open spaces at the study sites; Managers were also interviewed in-person on-site. Prescribed COVID-19 protocols were observed at all times. Experts were however, interviewed virtually.

2.3.Participant identification, study size and sampling

Participant recruitment and data collection occurred between January and February 2021. Using attendance records, a total enumeration of all eligible clients seen at both study sites from December 2020 to January 2021 was done. **These dates formed the frame and we included everyone within the frame, who met the eligibility criteria. The attendance records of each study site were used in retrieving the relevant information on potential participants.** Trained staff called all potential participants meeting eligibility criteria and invited them to participate. For each individual, three attempts were made to reach them. Interested participants were given appointments for a screening visit at the study sites and to undergo study procedures. Participants received reimbursement for travel costs and time. On average, each focus group discussion (FGD) lasted about an hour.

We assumed a 50% prevalence of diabetes self-management knowledge and 10% non-response rate. [23, 24] The level of significance was set at 5%. A sample size of 425 PLD was therefore required for the cross-sectional study. Recruitment for in-depth interviews (IDI) continued until saturation was reached and no new themes emerged.

PLD were identified through convenient sampling and snowballing for the qualitative study. Managers and healthcare professionals (HCPs) were sampled purposively, and judgemental sampling were used in identifying experts.

Fig 1. Convergent parallel mixed methods study design.

Abbreviations: IDI- in-depth interview FGD-Focus Group Discussion HCP- healthcare professional EM-experts and managers

Eligibility criteria for PLD, HCP, managers and experts

Participants had to meet all the following eligibility criteria and none of the exclusion criteria to be included. Experts were nationally recognised diabetologists. HCP and PLD were staff and attendants at the study sites respectively. Managers were the respective heads. PLD were 18 years or older and ambulant at the time of recruitment. People known to have type 1 diabetes, or cognitive or psychiatric impairment were excluded.

2.4. Instrument development

As we anticipated heterogeneity in responses, because of the case-mix variation, we developed semi-structured interview guides to guide all interviews. RL and MAC, who both understand the local culture and norms, developed and refined these interview guides. The questions were informed by results of a literature review of DSME in low-resource settings conducted by RL. Participant information guides on the purpose and methods of the study and anonymity was developed by RL and reviewed by MAC and KKG.

2.5. Data collection

The study was conducted in line with the principles of the Declaration of Helsinki.[25] Prior to any study procedures, each participant provided written informed consent. Participants who consented to take part in FGD, also signed non-disclosure statements. These statements were an assurance that information divulged by participants during the FGD would remain within the group and not shared outside the group. Since the sessions were audio taped and transcribed, participants were assigned codes names. Participants were referred to by their code names rather than their real names to maintain their confidentiality during the FGDs. Access to each facility was granted by their respective heads.

2.5.1. Quantitative data collection

Diabetes self-management knowledge of PLD, the primary outcome variable, was measured on the spoken knowledge in low literacy persons with diabetes scale (SKILLD).[26] SKILLD is a 10-item questionnaire with each option giving a score of either 0(0%) or 10(100%). Higher scores indicate better diabetes self-management knowledge.

The variables which were modelled as explanatory variables were anthropometric measures, sitting blood pressure, duration of diabetes, insulin use, random blood glucose ,sex, family history of diabetes, income, educational level, occupation and the summary of diabetes self-care activities scores (SDSCA).[27]

Measurement procedures

We scrupulously followed standard recommended procedures for all measurements.[28-30] We used StatStrip Xpress glucometer(Onetouch, Taiwan) to measure random blood glucose[29], and Omron M7 (Omron, Japan) to measure sitting blood pressure[28]. Omron digital scale, stadiometer, and inelastic tape measure were used to take anthropometric measurements.[30]

Duration of diabetes, insulin use, sex, family history of diabetes, income, educational level, and occupation were captured with a general questionnaire. The SKILLD and SDSCA instruments were interviewer administered.

Fig 2.Qualitative data collection procedures and number of informants.

Abbreviations: KBTH-Korle Bu Teaching Hospital; WGMH-Weija Gbawe Municipal Hospital IDI- in-depth interview FGD-Focus Group Discussion DM-duration of diabetes < less than > greater than yrs- years HCP-Health care professional PLD- person living with diabetes

2.5.2. Qualitative data collection

Fig 2 depicts the informants and qualitative procedures undertaken. RL and BB either conducted or coordinated the IDI and FGD. Interviews were conducted in in English, Twi, or Ga. Responses were audio-recorded digitally and handwritten field notes were taken. Some of the PLD recruited from the KBTH study site might have known RL as a staff of that facility. All other PLD involved in the study did not have any prior relationship with the data collectors. Experts and Health Care Professionals were colleagues of RL. The roles of the researchers were to facilitate the FGD and conduct the interviews.

2.6. Data management and analysis

2.6.1. Quantitative analysis

Total SKILLD score (knowledge) was analysed both as a continuous and categorical variable. The individual SKILLD items were dichotomised into correct and incorrect responses and summarised using counts (percentage).

To test the strength of the association between the total SKILLD score and SDSCA sub-domains, the Pearson’s correlation was employed. The appropriate regression tests involving ordinary least squares regression or quantiles regression were performed to assess the association between total SKILLD score) and clinically relevant variables. All analyses were conducted with Stata v16.1. Statistical significance was set at a two-sided p-value < 0.05. REDCap data management system was used for data capture.

2.6.2. Qualitative analysis

Data was analysed independently by RL, BB and a research assistant using an inductive thematic approach manually. Audio-recordings were transcribed verbatim. Transcription, initial coding, and thematic analysis were done manually concurrently with data collection. We extracted both latent and manifest content. Transcripts were line searched for recurring words and phrases. Concepts were then used to generate initial codes and further expanded by applying the codes to additional transcripts (open coding). Sub-themes were identified by reviewing the data for repeating patterns in participant’s

responses. Sub-themes were merged into themes, ensuring themes closely described original content of transcripts. Emerging themes were categorized and compared across the various (informants) groups using colour coded comparative charts. Direct quotes were extracted. Our informants were fully engaged in all phases of our study. We selected participants who could best provide answers to our research question. Data saturation was reached when no new themes emerged. Subsequently RL used Nvivo (released March 2020) to organise the data.

MAC reviewed the themes against the final organisation of the data to ensure that there was agreement in the data collected and its final presentation. Discrepancies and suggestions for review were resolved through dialogue.

Rigour

Data, informant, and investigator triangulation was used to ensure rigor and comprehension of concepts. The transcripts and subsequently thematic analysis were shared with informants to check for accuracy and to provide feedback. Team meetings with co-investigators experienced in qualitative methods enhance credibility of the data. Procedures have been described to allow replicability. Use of Nvivo improves transparency and reliability of the coding. Concurrent collection of quantitative and qualitative data improve internal validity.

2.7.Ethical approval

Ethical approval was granted by the Institutional Review Board of KBTH (STC/IRB/000175/2020) and the Ethics Review Committee of the Ghana Health Service (GHS-ERC 05/10/20). The head of each facility granted permission for the study after ethical clearance had been obtained.

3. Results

The quantitative results are summarised in tables and the qualitative results are presented by themes. All the quantitative results are presented first followed by the qualitative results.

3.1.Quantitative results

3.1.1.Participant's flow and baseline characteristics

In total, 1202 participants out of 1735 potentially eligible clients were not included. Reasons for this were as follows: 54 participants had travelled (zero from WGMH), 1029 were unreachable by telephone (544 from WGMH), 95 declined (one from WGMH), 25 were dead (one from WGMM). As 112 out of 533 eligible participants invited failed to report, four additional participants (0 from WGMH) were consecutively sampled. Finally, 425 participants were included in the analysis.

Participants' baseline socio-demographic and clinical characteristics are shown in Table 1. Additionally, the mean body weight was 98kg (SD 16). The mean waist circumference for males was 94 cm (SD 16) and for females it was 98 cm (SD 16). The mean systolic and diastolic blood pressure were 133 mmHg (SD 21) and 81 mmHg (SD 12) respectively. The mean random blood glucose was 9.4 mmol/l (SD 6.4) mmol/l.

Table 1. Descriptive (socio-demographic and clinical) characteristics of participants

Variable	Frequency	Percentage
Age(N=425)		
≤39	26	6
40-49	77	18
50-59	132	31
60-69	120	28
70+	70	17
Mean (SD)	581(SD 12)	
Sex (N=425)		
Female	298	70
Male	127	30
Educational level (N=425)		
None	52	12
Primary and middle	194	46
Secondary and vocational	118	27
Tertiary	58	14
Other	3	0.7
Marital Status (N=425)		
Married	245	58
Never married	24	5.7
Living together	1	0.2
Widowed	96	23
Divorced	59	14
Occupation (N=425)		
Professionals with university degrees	36	8.5
Professionals without university degree	30	7
Clerks, motor vehicle drivers, mechanic	89	21
Cooks, barbers, domestic staff, gas staff	36	8.5
Labourers and petty traders	86	20
Apprentices, educated youth, unemployed	148	35

Abbreviations; SD =Standard Deviation N=number of observations

Table 1 (continued). Descriptive (socio-demographic and clinical) characteristics of participants

Variable	Frequency	Percentage
Ethnicity (N=425)		
Akan	206	49
Ga/Adangbe	124	29
Ewe	53	13
Other	40	9.5
Religion (N=425)		
Christian	380	89
Islam	42	9.9
Other	3	0.7
Size of your household (N=412)		
1-2	91	22.09
3-4	136	33
5-6	116	28
6+	69	17
Min-Max	1-27	
Mean (SD)	5(3)	
Additional sources of income (N=417)		
No	342	82
Yes	75	18
Years of diabetes illness (N=416)		
≤1	48	12
2-3	95	23
4-9	138	33
10+	135	33
Min-Max	<1-45	
Mean (SD)	7.7 (0.3)	
Family history of diabetes (N=418)		
No	179	43
Yes	239	57
Have any device for checking blood sugar at home (N=418)		
No	252	60
Yes	166	40

Abbreviations; SD =Standard Deviation N=number of observations

3.1.2. Diabetes self-management knowledge among PLD

The median SKILLD score was 40 %(IQR 20-60). The results of the individual SKILLD items revealed significant deficits in diabetes self-management knowledge. Only 13 (3%) participants knew the normal HbA1c range and 162 (39%) knew the normal fasting glucose range. In total, 208 (50%) and 196 (40%) knew the signs of hyperglycaemia and hypoglycaemia, respectively. Only 227 (54%) knew how to treat hypoglycaemia. The importance of foot care was known by 135 (32%) and only126 (30%) participants knew the recommended frequency for foot examinations. The frequency of eye examinations and exercise was known by 176 (42%) and 199 (48%) respectively. Finally, 247 (59%) participants knew the long-term complications of diabetes.

3.1.3. Factors associated with diabetes self-management knowledge

There was no association between SKILLD score and any of the baseline socio-demographic and clinical variables.

3.1.4. Association between diabetes self-management knowledge and self-management behaviour

Pairwise corelations showed that SKILLD score was positively correlated with behaviour (SDSCA). The correlation coefficient was 0.22 ($p<0.01$) for diet, 0.19 ($p<0.01$) for medication, 0.14 for exercise ($p<0.05$), 0.39 ($p<0.01$) for glucose testing and 0.38 ($p<0.01$) for foot care.

3.1.5. Influence of diabetes-self-management knowledge (SKILLD) on Diabetes Self-Care Activities Measure(SDSCA) sub-domains

The effect of total SKILLD on self-management behaviours (SDSCA sub-domains), adjusted for age, education, diabetes duration, family history of diabetes and ownership of a glucometer is displayed in table 2.

Table 2. Influence of Knowledge (spoken language in Low Literacy in Diabetes scale) on Diabetes Self-Care Activities Measure sub-domains

Variable	Diabetes Self-Care Activities Measures				
	OLS		Quantile regression		
	Diet	Medication	Exercise	Blood testing	Foot
	aβ[95%CI]	aβ[95%CI]	aβ[95%CI]	aβ[95%CI]	aβ[95%CI]
SKILLED Knowledge	0.05[0.02-0.09]**	0.01[0.002-0.02]*	0.05[0.02-0.08]**	0.04[0.02-0.05]***	0.02[-0.02-0.05]
Age group					
≤39					
40-49	1.55[-2.39-5.48]	-0.73[-1.99-0.53]	1.00[-1.27-3.27]	1.07[-0.83-2.97]	0.33[-2.55-3.22]
50-59	1.21[-2.57-4.99]	0.37[-0.77-1.52]	2.00[-0.49-4.49]	0.93[-0.96-2.82]	0.17[-2.47-2.81]
60-69	1.03[-2.75-4.82]	0.20[-0.96-1.35]	1.00[-1.09-3.09]	1.07[-0.83-2.97]	0.33[-2.27-2.93]
70+	1.62[-2.46-5.70]	-0.03[-1.25-1.19]	0.50[-1.60-2.61]	2.07[-0.88-3.02]	0.33[-2.36-3.03]
Educational level					
None					
Primary	2.06[-0.93-5.05]	-0.96[-1.69- -0.24]**	1.22[-1.98-5.98]	-0.28[-0.94-0.37]	-0.17[-1.57-1.24]
Middle	1.77[-0.90-4.45]	-1.02[-1.59 -0.45]***	-0.50[-2.33-1.33]	-0.50[-1.05-0.05]	-0.17[-1.46-1.13]
Secondary	3.19[0.33-6.04]*	-1.39[--2.07- -0.71]***	2.50[-0.20-5.20]	0.07[-1.32-1.46]	0.17[-1.48-1.81]
Vocational	2.97[-2.19-4.83]	-1.28[-2.02- -0.36]**	-2.22[-3.32-6.32]	0.78[-2.57-4.14]	0.17[-2.97-3.31]
Tertiary	1.21[-2.19-4.62]	-1.24[-1.98- -0.50]***	0.50[-2.16-3.16]	2.86[0.81-4.90]	1.00[-1.36-3.36]
Other	-2.54[-11.8-6.75]	0.08[-0.76-0.92]	10.0[-17.2-37.2]	7.07[2.85-11.3]***	7.00[1.98-12.0]**
Years of diabetes illness					
≤1					
2-3	0.38[-2.45-3.21]	0.99[-0.01-1.99]	2.66[-2.18-3.18]	-0.34[-1.01-0.29]	-0.17[-1.48-1.15]
4-9	0.34[-2.41-3.09]	0.93[-0.01-1.89]	3.11[-2.45-6.45]	-0.50[-1.15-0.15]	-0.33[-1.81-1.14]
10+	0.85[-1.98-3.68]	1.25[0.30-2.21]	0.50[-1.85-2.85]	-0.35[-1.19-0.48]	-0.17[-1.83-1.50]
Family history of diabetes					
No					
Yes	-1.06[-2.69-0.57]	0.13[-0.31-0.58]	-0.50[-1.92-0.92]	0.00[-0.48-0.49]	-5.55[-1.00-5.99]
Device for checking blood sugar					
No					
Yes	2.34[0.60-4.08]**	0.61[0.20-1.03]**	-1.00[-2.38-0.39]	1.00[0.32-1.67]**	0.17[-0.94-1.27]

NOTE: Abbreviation: SKILLED= Spoken Language in Low Literacy in Diabetes; OLS-ordinary least squares regression; aβ= adjusted Coefficient estimate. Covariates used age, education, duration of diabetes and family history. *P*-value Notation; *** p<0.01, ** *p*<0.05, * *p*<0.1 type of test multiple linear regression

3.2.Qualitative results

3.2.1.Participants

Fig 2 depicts the types of informants and data gathering techniques used.

3.2.2.Emerging themes

The themes identified are displayed in Fig 3 and include health numeracy and financing, logistics and norms.

Fig 3. Thematic areas DSME needs in resource constrained settings.

i. DSME interventions

We found that PLD received DSME from nurses, doctors, and or nutritionists. The education was unstructured, didactic, group-based and delivered in-person prior to consultations. Groups typically had about 20 PLD per group and sessions lasted for about 30 minutes, on average.

We observed that varied perceptions among informants resulted in contrasting perspectives on existing DSME interventions. For example, PLD generally favoured group over individualised education, placing value on peer-to-peer learning. The consensus among PLD seemed to be that individualised education provided prior to a consultation was inadequate. They pointed out that the group sessions inadvertently provided avenues for newly diagnosed persons to draw on the experience and diabetes self-management knowledge of their peers. All patient groups interviewed, recommended that peers, together with health workers should be used as diabetes educators.

PLD described existing DSME interventions as beneficial but reported that teaching aids were not culturally or linguistically adapted.

R5 FGD KBTH –*"often the books available on diabetes have examples of foods eaten abroad"*

R4 FGD WGMH-*"….we have been given a book that teaches us how to manage diabetes. The book is normally read to me…. "*

R5 FGD WGMH*: "…..about the pamphlet. It sometimes contains foreign information which is their food and what they need to do in order to take care of themselves so I think they should be limited to our local activities"*

R3 FGD WGMH: *"……..I prefer all the teachings in a leaflet form…. Those who can't read the leaflet personally, can allow their children or friends to help them read"*

In contrast to PLD, providers and diabetes experts thought existing DSME interventions were at best parsimonious. Human resource constraints, lack of logistics, unavailability of academic courses, and a policy direction were challenges identified. Except for the doctors, none of the other participant groups were familiar with structured DSME.

The unstructured nature of existing DSME interventions meant PLD continued with self-management education classes ad-infinitum. Our informants appreciated the knowledge reinforcement.

R IDI KBTH: "…[They] *are doing their best because the doctors really educate the patients on how they can manage the diabetes themselves."*

PLD used DSME interchangeably with health education. They recommended that churches and other community spaces and mass media communication channels be used for DSME.

Most informants preferred the existing in-person format to virtual sessions.

ii. Diabetes self-management knowledge

Knowledge on self-management was deficient and self-care practices among PLD were inadequate.

R4 FGD KBTH: "*I used to inject the insulin in the house but anytime I inject it, my sugar level rises so a doctor friend of mine advised me that the insulin should be injected in the hospital and by a doctor so for 5years now I have stop using the insulin*."

PLDs echoed several myths as truths. Notwithstanding, PLD bemoaned the inconsistencies in nutritional recommendations.

iii. Self-management behaviours

PLD knew more about the importance of medication use, self-blood glucose testing, meal planning, exercise, and routine reviews than about foot care. None of the PLD and HCPs mentioned foot care. Contrastingly, foot care, routine investigations and eye screening were mentioned by the experts as being important components of self-management.

Several barriers to self-care, even when diabetes self-management knowledge was apparently adequate, were enumerated by all informants.

iv. Finance

Among persons with low health numeracy in resource constrained settings there's little choice in lifestyle. Poverty is the common pathway for restricted access to information, food, care, and medication. PLD described dependence on literate relatives to access useful information contained in patient education leaflets.

PLDs and HCPs enumerated the cost constraints faced by PLD and how those influenced food consumption patterns. HCPs were empathetic and yet seemingly frustrated by the vicious cycle of high carbohydrate consumption and hyperglycaemia among PLD. PLD and HCPs both indicated that consumption of fresh produce was dependent on seasonality.

PLD described frequent stockout of medications covered by insurance. None of the PLD groups complained about costs associated with home glucose testing. The experts however noted that patient's inability to afford home glucose monitoring was a barrier to optimising glycaemic control.

v. Norms and belief systems

Finances were not the only determinants of meal patterns. PLD voiced the conflict between their intentions and actions. They recounted the difficulty of executing planned behaviour (such as portion control). They described nutritional recommendations as a deviation from cultural norms. PLD described wanting to 'belong' at social gatherings. HCP and PLD alike alluded to the fact that diabetes (especially among young persons) was stigmatised.

PLD said they received conflicting messages from traditional herbal and alternative medicine practitioners, religious leaders, and HCPs. Furthermore, they expressed a belief in destiny and the existence of an external locus of control. These belief systems contributed to poor self-care.

4.Discussion

We sought to characterize DSME interventions and to explore the self-management knowledge and behaviours of persons living with diabetes. The interventions studied were unstructured, group-based and delivered in-person mostly by nurses. Self-management knowledge and behaviours were sub-optimal and influenced by conflicting messaging, financial constraints, culture, beliefs, and stigma.

Existing DSME interventions

The unstructured nature of the DSME interventions and use of group delivery methods probably reflects an attempt to increase the accessibility of DSME despite resource constraints. Building sustainability into DSME interventions for resource constrained settings, is key. The use of "non-internet" mass media to disseminate DSME interventions, as proposed by our informants might be a sustainable option. Moreover, since most of our informants found repetition of content useful, mass media channels may be well patronised. Similar to our findings, the importance of the traditional media in disseminating DSME was identified in another African study.[31] However, people living with long-standing diabetes in Iran reported that repetition of DSME content was not useful. A direct contrast to the views of informants in our study. Importantly, the population studied in Iran had significantly higher literacy levels relative to our study population and this difference may account for the disparities.[32] In Iran, health literacy has been shown to be positively correlated with health behaviours.[33]

Diabetes self-management knowledge and it's relation with self-management behaviours

Our findings of limited diabetes self-management knowledge echo those of previous studies.[34, 35] The extremely low SKILLD scores from our quantitative study reflect the depth of lack of knowledge on self-care. The themes we identified in this study provide some explanations for and elaborate on the inadequate diabetes self-management knowledge among PLD. In particular, the low literacy levels and inconsistent messaging are plausible explanations for the low SKILLD scores.

Despite the seemingly insurmountable barriers to self-care expressed by PLD, our results show that diabetes self-management knowledge is positively associated with several self-management behaviours. In congruence with our findings, a multi-centre cross-sectional study in Ghana found diabetes self-management knowledge to be a predictor of self-care: every 1 unit increase in knowledge was associated with 20 times the odds of higher SDSCA scores.[36] Although, the proportion of people with tertiary education was comparable to our study, the proportion of people with no education, was 50% higher relative to our study population.[36] Efforts at improving self-management knowledge might therefore ultimately also translate into better self-care behaviours among PLD in low-resource settings.

Our findings suggest, formal education is not associated with self-management behaviours except for adherence to medication. In contrast, Rothman et al found that having tertiary education was associated

with a 12% increase in SDSCA scores, indicating better self-care behaviours.[26] Surprisingly, a cross-sectional multi-centre study from Ethiopia, observed, that not having formal education was associated with increased odds of having good self-care behaviours (AOR = 2.6, 95% CI = 1.32-5.25).[37] This estimate of the effect of formal education on self-management behaviour could have been biased by the absence of a control group.

Diabetes self-management behaviours

Our findings of low scores across all domains of the SDSCA parallel findings from a multi-centre study in the Northern region of Ghana. [35] The socio-demographic and clinical profiles of the participants in these two studies were similar except for diabetes duration. The duration of diabetes was longer in the study by Mogre et al. [35], however, despite having had diabetes for longer, the self-management behaviours were just as sub-optimal as in our study. The low SDSCA scores from the quantitative study and the qualitative results from the IDI and FGDs both indicate poor self-management among PLD. It is plausible poor self-care behaviours are fuelled by both factors within and beyond the individual's control; particularly the financial challenges enumerated earlier. A cross-sectional study involving PLD in a specialist clinic of a tertiary teaching hospital in Nigeria also echo our findings of low scores on all domains of SDSCA.[38]

The alarmingly low knowledge scores on foot care, and correspondingly poor practice of foot care, in our study is disturbing. Our findings provide strong justification for emphasising foot care in DSME interventions. Curricula which emphasise the relation between amputations, glycaemic control, routines, and daily lifestyle choices would be beneficial. The qualitative results from our study provide further insight into the low scores in the domain of foot care and parallel findings from other sub-Saharan African countries[39] and other regions of Ghana.[35] Our findings also resonate with a qualitative facility-based study among a predominantly agricultural community.[34] Bossman et al reported deficits in diabetes self-management knowledge and self-care behaviours in the domains of nutrition, exercise, and foot care with foot care being the least known and practiced.[34] It is thus not surprising that, amputations are major causes of morbidity among PLD in Ghana and other sub-Saharan African countries.[40]

Our findings indicate a high demand for diabetes self-management information, especially, culturally tailored information on nutrition therapy albeit poor adherence to nutritional recommendations. Unfortunately, the edicts of self-care behaviours particularly in the domain of nutrition deviate from local cultural norms and this could contribute to the poor adherence. Furthermore, Unavailability of formal training in DSME for providers, could contribute to inconsistent messaging on nutritional therapy. Our findings parallel those from a study conducted in specialist clinic in Nigeria which reported confusion about nutritional recommendations, and the unacceptability of nutritional recommendations.[41]

We found that behaviour change seemed to be a hurdle that persisted despite adequate diabetes self-management knowledge. Our results suggest that our informants' capacity to modify established behaviours might be limited. Previous behaviour is a known predictor of adherence to self-care recommendations.[42] Incorporating education on behaviour change strategies may therefore be a useful addition to the existing DSME interventions.

Financial constraints

In this study, financial constraints transcend multiple aspects of diabetes self-management: adherence to self-management recommendations, keeping clinic appointments and purchasing medications. In particular, medications which were unavailable on the National Health Insurance were largely inaccessible. Likewise, for many of our informants, accessibility of vegetables was determined by their seasonality. Our findings collaborate previous findings from Ghana [43], and Benin.[44] de-Graft Aikins et al have previously shown that cost was a major and important limiting factor in several domains of self-management. [43]

Norms and belief systems

Some of our informants expressed the belief that the locus of control resides outside the individual. We found a belief in "divinity" which influenced perceptions of diabetes and diseases in general as reported widely in previous studies from Ghana[41, 43], Benin[44], Malawi and Mozambique.[31] Potentially, the local beliefs systems could adversely affect attitudes to self-care and self-care behaviours. This indicates a need to include sessions on the locus of control when designing DSME interventions for such settings.

Stigma

Hospital based DSME was more valued than community-based DSME because of diabetes-related stigma. Our finding that diabetes is stigmatised suggests that, having support persons as part of DSME interventions might be beneficial. Using peer educators may offer net-working opportunities for PLD and discussing disclosure may improve effectiveness of DSME interventions. The finding of stigma and lack of family support was also reported by Mogre et al. [45] Among Ghanaians, family non-support has been found to be negatively correlated with diabetes self-management behaviours.[46] Family support has a linear relation with self-care.[47]

Strengths and limitations

Quantitative analysis enabled us to generate valid unbiased estimates of diabetes self-management knowledge, and behaviours. The mixed methods design provided additional qualitative data and insights into the results of the quantitative study. The data was coded and analysed by researchers well accustomed to the Ghanaian culture. Data was generated from a variety of informants and study participants, managers, PLD, HCPs and experts.

The generalisability of the study to the Ghanaian population, however, is limited because the study was conducted only in two facilities within the Greater Accra region. However, the clientele of KBTH come from all over Ghana. **Our findings may also not be generalised to people known to have type 1 diabetes**. **Furthermore, the use of consecutive sampling may limit the representativeness of our sample.**

Conclusion

The DSME interventions studied were under-resourced and were not structured. Our findings indicate very limited diabetes self-management knowledge and poor adherence to self-care recommendations. Barriers to self-care included cost constraints, cultural norms, stigma and belief systems. DSME

interventions should incorporate sessions on mitigating these barriers. They should be culturally tailored and linguistically modified for people with low literacy. This may improve self-management, ultimately reducing the difficulties of PLD in resource constrained settings. Future mixed-methods cohort studies should focus on elucidating factors associated with effectives of DSME interventions in low resource settings.

Acknowledgements

We are thankful to Mr John Tetteh for assistance with running the statistical analysis.

References

1. IDF. International diabetes federation. IDF Diabetes Atlas, 10th edn Brussels, Belgium: International Diabetes Federation. 2021.

2. Owusu AY, Kushitor SB, Ofosu AA, Kushitor MK, Ayi A, Awoonor-Williams JK. Institutional mortality rate and cause of death at health facilities in Ghana between 2014 and 2018. PLoS One. 2021;16(9):e0256515. Epub 20210908. doi: 10.1371/journal.pone.0256515. PubMed PMID: 34496000; PubMed Central PMCID: PMCPMC8425528.

3. Asamoah-Boaheng M, Sarfo-Kantanka O, Tuffour AB, Eghan B, Mbanya JC. Prevalence and risk factors for diabetes mellitus among adults in Ghana: a systematic review and meta-analysis. International health. 2019;11(2):83-92.

4. Powers MA, Bardsley JK, Cypress M, Funnell MM, Harms D, Hess-Fischl A, et al. Diabetes self-management education and support in adults with type 2 diabetes: a consensus report of the American Diabetes Association, the Association of Diabetes Care & Education Specialists, the Academy of Nutrition and Dietetics, the American Academy of Family Physicians, the American Academy of PAs, the American Association of Nurse Practitioners, and the American Pharmacists Association. Diabetes Care. 2020;43(7):1636-49.

5. Chrvala CA, Sherr D, Lipman RD. Diabetes self-management education for adults with type 2 diabetes mellitus: A systematic review of the effect on glycemic control. Patient Educ Couns. 2016;99(6):926-43. Epub 2015/12/15. doi: 10.1016/j.pec.2015.11.003. PubMed PMID: 26658704.

6. Cunningham AT, Crittendon DR, White N, Mills GD, Diaz V, LaNoue MD. The effect of diabetes self-management education on HbA1c and quality of life in African-Americans: a systematic review and meta-analysis. BMC Health Serv Res. 2018;18(1):367. Epub 20180516. doi: 10.1186/s12913-018-3186-7. PubMed PMID: 29769078; PubMed Central PMCID: PMCPMC5956958.

7. Powers MA, Bardsley J, Cypress M, Duker P, Funnell MM, Fischl AH, et al. Diabetes Self-management Education and Support in Type 2 Diabetes. Diabetes Educ. 2017;43(1):40-53. doi: 10.1177/0145721716689694. PubMed PMID: 28118121.

8. Reisi M, Fazeli H, Mahmoodi M. Application of the social cognitive theory to predict self-care behavior among type 2 diabetes patients with limited health literacy. Journal of health litaracy. 2021;6(2):21-32.

9. Othman MM, Khudadad H, Dughmosh R, Furuya-Kanamori L, Abou-Samra AB, Doi SAR. Towards a better understanding of self-management interventions in type 2 diabetes: A concept analysis. Prim Care Diabetes. 2021. Epub 20210920. doi: 10.1016/j.pcd.2021.09.001. PubMed PMID: 34556438.

10. Ryan JG, Jennings T, Vittoria I, Fedders M. Short and long-term outcomes from a multisession diabetes education program targeting low-income minority patients: a six-month follow up. Clinical therapeutics. 2013;35(1):A43-A53.

11. Lynch EB, Mack L, Avery E, Wang Y, Dawar R, Richardson D, et al. Randomized trial of a lifestyle intervention for urban low-income African Americans with type 2 diabetes. J Gen Intern Med. 2019;34(7):1174-83.

12. Tang TS, Funnell MM, Noorulla S, Oh M, Brown MB. Sustaining short-term improvements over the long-term: results from a 2-year diabetes self-management support

(DSMS) intervention. Diabetes Res Clin Pract. 2012;95(1):85-92. Epub 20110827. doi: 10.1016/j.diabres.2011.04.003. PubMed PMID: 21872962; PubMed Central PMCID: PMCPMC3783218.

13. Dube L, Van den Broucke S, Dhoore W, Kalweit K, Housiaux M. An audit of diabetes self-management education programs in South Africa. Journal of public health research. 2015;4(3):jphr. 2015.581.

14. Mash R, Kroukamp R, Gaziano T, Levitt N. Cost-effectiveness of a diabetes group education program delivered by health promoters with a guiding style in underserved communities in Cape Town, South Africa. Patient Educ Couns. 2015;98(5):622-6. Epub 2015/02/03. doi: 10.1016/j.pec.2015.01.005. PubMed PMID: 25641665.

15. Lamptey R, Robben MP, Amoakoh-Coleman M, Boateng D, Grobbee DE, Davies MJ, et al. Structured diabetes self-management education and glycaemic control in low- and middle-income countries: A systematic review. Diabet Med. 2022:e14812. Epub 20220218. doi: 10.1111/dme.14812. PubMed PMID: 35179792.

16. Bekele BB, Negash S, Bogale B, Tesfaye M, Getachew D, Weldekidan F, et al. Effect of diabetes self-management education (DSME) on glycated hemoglobin (HbA1c) level among patients with T2DM: Systematic review and meta-analysis of randomized controlled trials. Diabetes Metab Syndr. 2021;15(1):177-85. Epub 20201219. doi: 10.1016/j.dsx.2020.12.030. PubMed PMID: 33360516.

17. Kumah E, Otchere G, Ankomah SE, Fusheini A, Kokuro C, Aduo-Adjei K, et al. Diabetes self-management education interventions in the WHO African Region: A scoping review. PLoS One. 2021;16(8):e0256123. Epub 2021/08/18. doi: 10.1371/journal.pone.0256123. PubMed PMID: 34403455; PubMed Central PMCID: PMCPMC8370626.

18. Gathu CW, Shabani J, Kunyiha N, Ratansi R. Effect of diabetes self-management education on glycaemic control among type 2 diabetic patients at a family medicine clinic in Kenya: A randomised controlled trial. Afr J Prim Health Care Fam Med. 2018;10(1):e1-e9. Epub 2018/11/21. doi: 10.4102/phcfm.v10i1.1762. PubMed PMID: 30456964; PubMed Central PMCID: PMCPMC6244221.

19. Essien O, Otu A, Umoh V, Enang O, Hicks JP, Walley J. Intensive Patient Education Improves Glycaemic Control in Diabetes Compared to Conventional Education: A Randomised Controlled Trial in a Nigerian Tertiary Care Hospital. PLoS One. 2017;12(1):e0168835. Epub 20170103. doi: 10.1371/journal.pone.0168835. PubMed PMID: 28045979; PubMed Central PMCID: PMCPMC5207750.

20. Klassen AC, Creswell J, Plano Clark VL, Smith KC, Meissner HI. Best practices in mixed methods for quality of life research. Qual Life Res. 2012;21(3):377-80. Epub 20120204. doi: 10.1007/s11136-012-0122-x. PubMed PMID: 22311251.

21. O'Cathain A, Murphy E, Nicholl J. The quality of mixed methods studies in health services research. J Health Serv Res Policy. 2008;13(2):92-8. doi: 10.1258/jhsrp.2007.007074. PubMed PMID: 18416914.

22. Tong A, Sainsbury P, Craig J. Consolidated criteria for reporting qualitative research (COREQ): a 32-item checklist for interviews and focus groups. Int J Qual Health Care. 2007;19(6):349-57. Epub 20070914. doi: 10.1093/intqhc/mzm042. PubMed PMID: 17872937.

23. Zowgar AM, Siddiqui MI, Alattas KM. Level of diabetes knowledge among adult patients with diabetes using diabetes knowledge test. Saudi Med J. 2018;39(2):161-8. doi: 10.15537/smj.2017.2.21343. PubMed PMID: 29436565; PubMed Central PMCID: PMCPMC5885093.

24. Muninarayana C, Balachandra G, Hiremath SG, Iyengar K, Anil NS. Prevalence and awareness regarding diabetes mellitus in rural Tamaka, Kolar. Int J Diabetes Dev Ctries. 2010;30(1):18-21. doi: 10.4103/0973-3930.60005. PubMed PMID: 20431801; PubMed Central PMCID: PMCPMC2859279.

25. World Medical A. World Medical Association Declaration of Helsinki: ethical principles for medical research involving human subjects. JAMA. 2013;310(20):2191-4. doi: 10.1001/jama.2013.281053. PubMed PMID: 24141714.

26. Rothman RL, Malone R, Bryant B, Wolfe C, Padgett P, DeWalt DA, et al. The spoken knowledge in low literacy in diabetes scale: a diabetes knowledge scale for vulnerable patients. Diabetes Educ. 2005;31(2):215-24. doi: 10.1177/0145721705275002. PubMed PMID: 15797850.

27. Toobert DJ, Hampson SE, Glasgow RE. The summary of diabetes self-care activities measure: results from 7 studies and a revised scale. Diabetes Care. 2000;23(7):943-50. doi: 10.2337/diacare.23.7.943. PubMed PMID: 10895844.

28. Umemura S, Arima H, Arima S, Asayama K, Dohi Y, Hirooka Y, et al. The Japanese Society of Hypertension guidelines for the management of hypertension (JSH 2019). Hypertension Research. 2019;42(9):1235-481.

29. Sagkal Midilli T, Ergin E, Baysal E, Ari Z. Comparison of glucose values of blood samples taken in three different Ways. Clin Nurs Res. 2019;28(4):436-55. Epub 20170706. doi: 10.1177/1054773817719379. PubMed PMID: 28679284.

30. Sellen D. Physical Status: The Use and Interpretation of Anthropometry. Report of a WHO Expert Committee. WHO Technical Report Series No. 854. Pp. 452.(WHO, Geneva, 1995.) Swiss Fr 71.00. Journal of Biosocial Science. 1998;30(1):135-44.

31. Bamuya C, Correia JC, Brady EM, Beran D, Harrington D, Damasceno A, et al. Use of the socio-ecological model to explore factors that influence the implementation of a diabetes structured education programme (EXTEND project) inLilongwe, Malawi and Maputo, Mozambique: a qualitative study. BMC Public Health. 2021;21(1):1355. Epub 20210708. doi: 10.1186/s12889-021-11338-y. PubMed PMID: 34238258; PubMed Central PMCID: PMCPMC8268266.

32. Mardanian Dehkordi L, Abdoli S. Diabetes self-management education; experience of people with diabetes. J Caring Sci. 2017;6(2):111-8. Epub 20170601. doi: 10.15171/jcs.2017.011. PubMed PMID: 28680865; PubMed Central PMCID: PMCPMC5488666.

33. Mirsamiyazdi N, Jafaripour F, Taqvaeinasab H, Masoudiyekta L, Amiri R, Komeilifar Z. The relationship between health literacy and health promoting behaviors in patients with type2 diabetes. Journal of Health Literacy. 2021;6(3):24-31.

34. Bossman IF, Dare S, Oduro BA, Baffour PK, Hinneh TK, Nally JE. Patients' knowledge of diabetes foot complications and self-management practices in Ghana: A phenomenological study. PLoS One. 2021;16(8):e0256417. Epub 20210825. doi: 10.1371/journal.pone.0256417. PubMed PMID: 34432838; PubMed Central PMCID: PMCPMC8386847.

35. Mogre V, Abanga ZO, Tzelepis F, Johnson NA, Paul C. Adherence to and factors associated with self-care behaviours in type 2 diabetes patients in Ghana. BMC Endocr Disord. 2017;17(1):20. Epub 20170324. doi: 10.1186/s12902-017-0169-3. PubMed PMID: 28340613; PubMed Central PMCID: PMCPMC5366118.

36. Afaya RA, Bam V, Azongo TB, Afaya A, Kusi-Amponsah A, Ajusiyine JM, et al. Medication adherence and self-care behaviours among patients with type 2 diabetes

mellitus in Ghana. PLoS One. 2020;15(8):e0237710. Epub 20200821. doi: 10.1371/journal.pone.0237710. PubMed PMID: 32822381; PubMed Central PMCID: PMCPMC7446850.

37. Molalign Takele G, Weharei MA, Kidanu HT, Gebrekidan KG, Gebregiorgis BG. Diabetes self-care practice and associated factors among type 2 diabetic patients in public hospitals of Tigray regional state, Ethiopia: A multicenter study. PLoS One. 2021;16(4):e0250462. Epub 20210421. doi: 10.1371/journal.pone.0250462. PubMed PMID: 33882117; PubMed Central PMCID: PMCPMC8059799.

38. Jackson IL, Onung SI, Oiwoh EP. Self-care activities, glycaemic control and health-related quality of life of patients with type 2 diabetes in a tertiary hospital in Nigeria. Diabetes Metab Syndr. 2021;15(1):137-43. Epub 20201214. doi: 10.1016/j.dsx.2020.12.027. PubMed PMID: 33340873.

39. Stephani V, Opoku D, Beran D. Self-management of diabetes in sub-Saharan Africa: A systematic review. BMC Public Health. 2018;18(1):1148. Epub 20180929. doi: 10.1186/s12889-018-6050-0. PubMed PMID: 30268115; PubMed Central PMCID: PMCPMC6162903.

40. Boateng D, Ayellah BB, Adjei DN, Agyemang C. Contribution of diabetes to amputations in sub-Sahara Africa: A systematic review and meta-analysis. Primary Care Diabetes. 2022.

41. Hushie M. Exploring the barriers and facilitators of dietary self-care for type 2 diabetes: a qualitative study in Ghana. Health Promot Perspect. 2019;9(3):223-32. Epub 20190806. doi: 10.15171/hpp.2019.31. PubMed PMID: 31508343; PubMed Central PMCID: PMCPMC6717922.

42. Jannuzzi FF, Cornelio ME, Sao-Joao TM, Gallani MC, Godin G, Rodrigues RCM. Psychosocial determinants of adherence to oral antidiabetic medication among people with type 2 diabetes. J Clin Nurs. 2020;29(5-6):909-21. Epub 20200106. doi: 10.1111/jocn.15149. PubMed PMID: 31856319.

43. de-Graft Aikins A, Awuah RB, Pera TA, Mendez M, Ogedegbe G. Explanatory models of diabetes in urban poor communities in Accra, Ghana. Ethn Health. 2015;20(4):391-408. Epub 20140722. doi: 10.1080/13557858.2014.921896. PubMed PMID: 25048822.

44. Alaofe H, Yeo S, Okechukwu A, Magrath P, Amoussa Hounkpatin W, Ehiri J, et al. Cultural considerations for the adaptation of a diabetes Self-management education program in Cotonou, Benin: Lessons learned from a qualitative study. Int J Environ Res Public Health. 2021;18(16). Epub 20210807. doi: 10.3390/ijerph18168376. PubMed PMID: 34444125; PubMed Central PMCID: PMCPMC8393923.

45. Mogre V, Johnson NA, Tzelepis F, Paul C. Barriers to diabetic self-care: A qualitative study of patients' and healthcare providers' perspectives. J Clin Nurs. 2019;28(11-12):2296-308. Epub 20190306. doi: 10.1111/jocn.14835. PubMed PMID: 30791160.

46. Amankwah-Poku M, Amoah AGB, Sefa-Dedeh A, Akpalu J. Psychosocial distress, clinical variables and self-management activities associated with type 2 diabetes: a study in Ghana. Clin Diabetes Endocrinol. 2020;6:14. Epub 20200714. doi: 10.1186/s40842-020-00102-7. PubMed PMID: 32685189; PubMed Central PMCID: PMCPMC7362489.

47. Gunggu A, Thon CC, Whye Lian C. Predictors of diabetes self-management among type 2 diabetes patients. J Diabetes Res. 2016;2016:9158943. Epub 20160803. doi: 10.1155/2016/9158943. PubMed PMID: 27563681; PubMed Central PMCID: PMCPMC4987486.

31st October, 2022

The Editorial Office
PLOS ONE

Dear Jamie Males,

RESPONSE TO REVIEWERS' COMMENTS

PONE-D-22-15096: DIABETES SELF-MANAGEMENT EDUCATION IN LOW-RESOURCE SETTINGS: A MIXED METHODS NEEDS ASSESSMENT OF PROVIDERS AND PEOPLE LIVING WITH DIABETES

We are deeply indebted to the reviewers for their time. We highly appreciate the time you and the team of editors have already committed to helping us improve our paper. Please find a point-by-point response to each comment in the table below. Our paper has been revised with these responses.

We have also modified the funding statement as follows:

Funding

The study was funded in part by the UMC Utrecht Global Health Support PhD program. It had no role in the study design, collection, analysis, interpretation of data, writing of the report or decision to submit the article for publication.

No	Reviewer's comment	Response to reviewer's comment
	1. Is the manuscript technically sound, and do the data support the conclusions?<br><br>The manuscript must describe a technically sound piece of scientific research with data that supports the conclusions. Experiments must have been conducted rigorously, with appropriate controls, replication, and sample sizes. The conclusions must be drawn appropriately based on the data presented.<br><br>Reviewer #1: No	Thank you.<br>We have reviewed the entire manuscript for clarity in communicating our processes and procedures. We were meticulous in following research methods, and we believe our work is technically sound.

	Reviewer #2: Partly<br><br>Reviewer #3: Partly	
	2. Has the statistical analysis been performed appropriately and rigorously?<br><br>Reviewer #1: I Don't Know<br><br>Reviewer #2: Yes<br><br>Reviewer #3: I Don't Know	We believe our analyses have been conducted appropriately and with rigour.
	3. Have the authors made all data underlying the findings in their manuscript fully available?<br><br>The PLOS Data policy requires authors to make all data underlying the findings described in their manuscript fully available without restriction, with rare exception (please refer to the Data Availability Statement in the manuscript PDF file). The data should be provided as part of the manuscript or its supporting information, or deposited to a public repository. For example, in addition to summary statistics, the data points behind means, medians and variance measures should be available. If there are restrictions on publicly sharing data—e.g. participant privacy or use of data from a third party—those must be specified.<br><br>Reviewer #1: Yes<br><br>Reviewer #2: Yes<br><br>Reviewer #3: Yes	--
	4. Is the manuscript presented in an intelligible fashion and written in standard English?	In response to reviewer 1, We have further reviewed the entire manuscript to ensure

	PLOS ONE does not copyedit accepted manuscripts, so the language in submitted articles must be clear, correct, and unambiguous. Any typographical or grammatical errors should be corrected at revision, so please note any specific errors here.<br><br>Reviewer #1: No<br><br>Reviewer #2: Yes<br><br>Reviewer #3: Yes	that the language used is suitable for a scientific paper. We have endeavoured to correct all language use errors.
	Reviewer 1	
	Reviewer #1: a. I applaud the authors for conducting an interesting article. However, is still needed to improve the quality of this paper. Please revise the manuscript to address the expressed concerns. After thorough review, I am recommending some revisions. In this regard, kindly address the following comments and suggestions to further improve your manuscript	Thank you.
	b. There are some spelling and grammatical errors in the text. Please correct them	We have reviewed the manuscript and endeavoured to correct all language use errors.
	c. Explain about the qualitative method in the abstract method section and mention the important qualitative results in the abstract results section	These were previously done. In our original submission we stated the following:<br><br>1.A convergent parallel—This explains the mixed method both qualitative and quantitative. In response to the reviewer's comment, in addition to the reference already provided, and Figure 1, we have modified the section

		2.1 design and included this further explanation:<br><br>"Thus, we merged the two research methods (quantitative and qualitative) to answer our research questions and achieve our study aims. In addition, the two methods converged at the point of analysing the results and interpretating the data. Data for the quantitative study and qualitative study were collected simultaneously, in parallel. Moreover, we placed equal emphasis on qualitative and quantitative data in all aspects of the study."<br>We believe this increases the reproducibility of our method. Thank you.<br><br>2. In the abstract we stated that "we employed inductive content analysis of informants' experiences and context".<br><br>In the main manuscript we explained further by stating "Data was analysed independently by RL, BB and a research assistant using an inductive thematic approach manually"-These explain the qualitative method<br><br>2.In the abstract we stated, "Financial constraints, conflicting messages, beliefs, and stigma were themes underpinning behaviours."-These are the quantitative results

		We then went on to describe the qualitative results in detail in the main manuscript.
	d. Please write the type of study, sample size, sampling strategy and date and country of study in abstract	These were previously done. We stated the following in our original submission:<br><br>1. A convergent parallel mixed-methods study was conducted . We went on to describe this method further in the main manuscript. In response to the reviewers comment we have provided further and better particulars of this type of study in addition to the reference already provided<br><br>2. sample size: In total 425 PLD…..<br>Two managers, five professionals, two diabetes experts and 16 PLD participated in in-depth interviews. Finally, 24 PLD<br><br>3. We have now provided further clarity to our sampling strategy.<br><br>, a total enumeration of all eligible clients seen at both study sites from December 2020 to January 2021 was done. **These dates formed the frame and we included everyone within the frame, who met the eligibility criteria. The attendance records of each study site were used in retrieving the relevant**

		**information on potential participants.** "<br><br>"PLD were identified through convenient sampling and snowballing for the qualitative study. Managers and healthcare professionals (HCPs) were sampled purposively, and judgemental sampling were used in identifying experts."<br><br><br>4.date and country of the study Thank you we have included this "January to February 2021 in Accra, Ghana"
	e. The introduction section need some revision. You could summarize this section a bit more for readers. Write about the problems, the novelty of your study, the limitations of prior research might also be mentioned by the authors as further support for their present investigation and your study goals within the introduction. In this section, you can use the following articles:<br>1- " Application of the Social Cognitive Theory to Predict Self-Care Behavior among Type 2 Diabetes Patients with Limited Health Literacy"<br>2- "The Relationship Between Health Literacy and Health Promoting Behaviors in Patients with Type2 Diabetes"	We have re-written the entire introduction section.<br><br>We have modified our title to ensure that the title, new introduction, aims, and conclusions are congruent<br><br>We have found it useful to include the suggested references in the introduction and discussion sections of the manuscript respectively.<br><br>Thank you for helping us improve our manuscript.
	f. In the introduction, you should fully explain why you used the qualitative method?	This was previously done.<br>We stated that "We employed qualitative methods to deepen our understanding (of generalizable) outcomes from the quantitative study"
	g. The materials & methods section is relatively immature. You could expand it a bit more clearly for readers. For example, Where have	These were previously done<br><br>1. Name and place where we did the study : We stated that

	you collected samples? Write the year and the name of place in which you had done this survey. Furthermore, write about all applied exclusion and inclusion criteria a bit more clearly by which you selected samples for this survey.	"The study was conducted in Korle Bu Teaching Hospital polyclinic (KBTH) and Weija Gbawe Municipal hospital (WGMH),<br><br>2.where samples were collected: two public primary facilities located in Accra, Ghana.<br><br>Interviews were conducted at the study sites either in offices or large open spaces whilst observing prescribed COVID-19 protocols. Experts were interviewed virtually.<br>"<br>3. year study conducted: We also stated that "Participant recruitment and data collection occurred between January and February 2021"<br>4. applied exclusion and inclusion criteria: We also stated that "HCP and PLD were staff and attendants at the study sites respectively. Managers were the respective heads. PLD were 18 years or older, not known to have type 1 diabetes, cognitive or psychiatric impairment and ambulant.<br>"
	h. Discuss more about your sampling strategy in both qualitative and quantitative section? The structure of your sampling is so vague and understandable. Did you have sampling frame? how did you access to this frame	This was previously done.<br>We stated that "a total enumeration of all eligible clients seen at both study sites from December 2020 to January 2021 was done." For clarity we have added this sentence: "These dates formed the frame and we included

		everyone within the frame, who met the eligibility criteria "
	What are the data extract's center characteristics? is it governmental or private, is it referral or not referral and so on, discuss more about it	This was previously done.<br><br>We stated that "The study was conducted in Korle Bu Teaching Hospital polyclinic (KBTH) and Weija Gbawe Municipal hospital (WGMH), two public primary facilities located in Accra, Ghana". The facilities were government primary care facilities
	j. How many observers did you have? if you had more than one observer, you must mention agreement	Thank you. We mentioned in our original submission that discrepancies were resolved through dialogue. We had 3 observers engaging the PLD . One observer engaged both managers<br>2 observers engaged the HCW.<br>2 observers engaged the Experts.
	k. The methods need to be improved by providing more detail information related to participant's selection (e.g. respond rate; necessary permissions from who? How did the researcher contact the potential participants?)	Response rate: We have now included the non-response rate "21%"<br><br>Permissions: We had stated the following in our original submission"The head of each facility granted permission for the study after having obtained ethical clearance"<br><br>We previously stated the following in our original submission "Trained staff called all potential participants meeting eligibility criteria and invited them to participate. For each individual, three attempts were made to reach them."
	l. Please prepare a method section	This was previously done . We stated in our original

	based on consolidated criteria for reporting qualitative research (COREQ) guidelines	submission that "The Good Reporting of a Mixed-Methods Study (GRAMMS)(5) and Consolidated Criteria for REporting Qualitative research (COREQ)(6) checklists were followed.<br>"<br>In response to the reviewer, we now have added the COREQ checklist as supporting material." Thank you
	m. Please write the type of Qualitative study (for example Grounded Theory, Content analysis, …) sample size, sampling strategy in qualitative method section	This was previously done in the abstract as well as in the main manuscript.<br>We stated the following and provided details of our method<br>1."using an inductive content analysis"<br><br>2."using an inductive thematic approach manually"
	n. Sampling in qualitative section was done until redundancy in data was reached? the types and levels of participation of the participants should also be described	Thank you. The following statements have now been included "Our informants were fully engaged in all phases of our study. We selected participants who could best provide answers to our research question."
	o. The researcher's role(s), level of participation and relationship with participants also needs to be described in qualitative section, as they can influence the findings	Thank you . The following statement has been added "Some of the PLD recruited from the KBTH study site might have known RL as a staff of that facility. All other PLD involved in the study did not have any prior relationship with the data collectors. Experts and Health Care Professionals were colleagues of RL. The roles of the researchers were to facilitate the FGD and conduct the interviews"
	p. You could increase the number of more recently studies in the reference section. You should have comprehensive and reliable comparisons between your findings	Our original discussion section included 14 references 9 which were published within the last 3 years and all the 14

	with the other previous studies. In the discussion, you did not include related previous studies in relation to the findings of the current study. Please search and cite related studies and include them in your discussion	references were published within the last 7 years.<br><br>We have in addition significantly increased the number of references in the introduction. Thank you.
	q. In the discussion section, more interpretations are needed	This has been done . Thank you. We have done a thorough review of the entire discussion section
	Reviewer #2: Thank you for the opportunity to review this paper. It is a very interesting and well written paper. It addresses a very important topic.<br>I provide my suggestions below which the authors may use for further improvement.	Thank you
	1. Abstract: Lines 50-52, the authors may revise the statement to include the percentage increase in the sentence, then the CI and the range are put in parentheses.	We tried to rephrase the sentence to accommodate this suggestion however the sentence did not read very well<br>.
	2. The introduction is not sufficiently grounded in the literature. The authors have not demonstrated their knowledge of what has been done already on the topic. Also, they have not provided enough justification for a need for the study. What is the research gap? And why is the study important for policy and practice.	We have increased the number of references in the introduction thank you.<br><br>The introduction has been re-written form clarity thank you.
	Self-Management Education is a behavior change intervention that is lees known and implemented in Africa. Authors should therefore provide sufficient information so readers will not confuse it with the normal self-management education that is provided to patients with diabetes when they seek routine clinical care. The topic is about diabetes self-management education (DSME). What is this DSME? Such background information is necessary.	Additional background information has been provided in the introduction for clarity. We have also replaced self-management program with self-management intervention. Thank you
	3. The diabetes prevalence rate of 26% among the adult population in Ghana	Thank you this has now been corrected to 6.5% prevalence.

	attributed to Jie Li et al., 2018 (lines 68 and 69) as the source may be incorrect. Jie Li et al. were talking about the prevalence of cardiovascular disease (CVD) risk factors and 26.1% was attributed to diabetes mellitus. They were not talking about diabetes prevalence among adult population in Ghana. They were talking about diabetes mellitus being a risk factor of CVD.	
	4. Lines 70-78, the entire two paragraphs have no single citation. Are the assertions based on the authors opinion? If the answer is no, then they need to credit the sources.	Thank you. This entire section has been re-written
	5. I am a little bit confused about the study aim and the conclusion drawn. The aim of the study is to understand DSME needs of patients and care providers. The conclusion is that "Diabetes self-management education tailored to resource-constrained settings are needed". What exact DSME needs of patients and providers did they find? It is already a known fact that DSMEs are needed for patients so they could self-manage their conditions. If the authors want to justify the need of DSME for patients, then the study aim has to be reconstructed. Also, what is understanding DSME needs of providers? Knowledge? resources? Some clarity is needed. It should also be noted that self-management needs are not the same as self-management education needs. Self-management education is an intervention provided to patients to build their capacity to self-manage their disease or engage in effective self-care behaviors.	The title, study aim, and conclusions have been re-written for clarity and is now more focused and congruent. Thank you
	6. Authors need to be specific about the type of diabetes patients being studied. People living with diabetes (PLD) means all diabetes types including type 1. But from line 116, they have stated that PLD were 18 years and above and not known to have type 1 diabetes. This means they are	There is a fine line between various types of diabetes and often it is clinically difficult to distinguish between for example latent autoimmune diabetes in adults and type 2 diabetes. Given that we did not

	taking about people with type 2 diabetes. This should be used instead of people living with diabetes, Gestational diabetes is also a form of diabetes. Thus, authors should be specific right from the outset of the paper about the specific type of diabetes patients being referred to.	do formal testing we decided it was best to avoid classifying patients as type 2 diabetes. The inclusion criteria was self-reported diabetes and we excluded those known to have type 1 diabetes. We have now included this in our limitation "our findings may not be generalised to people known to have type 1 diabetes"
	7. Lines 261 and 262, authors state "PLD receive DSME from nurses, doctors, and or nutritionists. It is un-structured, didactic, group based and delivered in-person prior to consultations". A clear distinction needs to be made between the diabetes education given to patients during routine clinical care and diabetes self-management education program, which is a behavior change intervention designed and delivered to improve patients' elf-efficacy in self-managing their conditions. Diabetes self-management education programs are underpinned by behavior change theories and models such as Bandura's Self-Efficacy Theory. Thus, the routine education on self-care given to patients during clinical visits could not be classified as a self-management education program, although it is self-management education. Authors therefore need to be clear whether they are referring to self-management education given to patients during routine clinic visits or DSME which is a new model of diabetes care and which aims at empowering patients to engage in effective self-care behaviors. The majority of DSMEs are delivered in non-clinical settings and some are led by laypersons or peer educators (people with diabetes trained as educators).	Thank you we have replaced dsme program with dsme intervention throughout the manuscript..
	8. This takes me back to lines 77 and 78. The statement that "Sustainability and by extension availability of DSME is influenced by patient-, provider- and facility-level factors" holds for DSMEs	Thank-you. This statement has been modified We have now specified facility-based DSME.

	delivered in clinic-settings. In other settings, DSMEs are provided by NGOs and these have nothing to do with providers and facility-based factors. Some DSMEs are even delivered I churches. And it must be stated that the facilitator of DSME is not always a clinician. DSME is separate from clinical care. The current literature calls for its integration in routine clinical care.	"Additionally, sustainability of facility-based structured DSME interventions are influenced by facility-, patient-, and provider level factors.[13]"
	. Conclusion: The first sentence reads "Existing DSME services are under-resourced and there are no structured DSME programs available." Are they talking about the two study sites or the entire country? I think being specific rathe than providing general statements will help.	Thank you<br>We have re-written the entire concluding paragraph . Those findings are limited to the two study sites.<br>"The DSME interventions studied were under-resourced and were not structured"
	10. Authors may do thorough proofreading as there are some identified typos in the work.	Thank you this has been done
	Reviewer #3: I would like to thank the authors for their important work on assessing management of diabetes and educational needs in LMICs, this is a critical topic and this work is an important contribution to the field. The research question and methods need to be articulated clearly from the beginning however, the rest of the manuscript is clear.	Thank you
	Abstract<br>• Overall, Adequately described<br>• Clarify aim regarding what exactly will you be studying from the provider group<br>• Line 41: Designate the location of the study as Accra, Ghana	The aim has been re-stated for clarity and the study location included in the Abstract<br>"We sought to characterise DSME interventions in two urban low-resource primary settings, and to explore diabetes self-management knowledge and behaviours of persons living with diabetes (PLD).<br>"

	Introduction<br>• Overall: a brief overview of diabetes self-management (DSME)<br>• Minor grammatical errors<br>• Authors should clarify early on that this study is reviewing the existing DSME in Accra, Ghana in two hospitals and its beliefs of and impact on patients, experts, and health care providers (HCPs). This should clearly be stated at the end of the introduction as the research question.<br>• Similarly, the methods do not describe the exisiting DSME intervention as what is being studied<br>o This is in the results section◊ existing DSME programs that are unstructured and they are reviewing whether the programs were are helpful to the patients, HCP, and experts - -- tlead with this and that you are observing its efficacy, usage, and public reaction<br>• Line 81: Designate the location with the city - Accra, Ghana	The entire introduction has been re-written for clarity and to improve congruency with the other sections of the manuscript thank you.<br>The aim has been re-stated at the end of the introduction<br><br><br><br>Our aim was rather to describe and characterise the existing DSME interventions<br><br>The location within the city has been designated
	Methods<br>• Overall: Provided a thorough list of methods with minimal grammatical errors and information regarding ethical considerations<br>• Actual intervention being studied is missing, it is critical to describe what is being evaluated so the reader understands the setting. Thorough descriptions of necessary sample size and methods of recruitment is there but what is being assessed is not included here and needs to be.<br>• Ethical considerations found in Data collection section: written informed consent, non-disclosure statements; codes assigned to maintain confidentiality<br>• Transcripts and analysis were shared with informants to check for accuracy and provide feedback- sounds excellent!<br>• Ethical approval: IRB from KBTH and Ethics review committee of Ghana Health Service<br>Line Edits	Our aim was rather to describe and characterise the existing DSME interventions. The aim has been re-written. Thank you<br><br>The abbreviations in the Figures have been corrected. Thank you.<br>The section on eligibility criteria has now been clearly labelled.<br><br>Figure 1 reorganised as suggested<br>Figure 2- Abreviations have been corrected<br>Figure 3 has been re-drawn; the major theme in that circle is stigma<br>The section on quantitative analysis has been titled to maintain formatting with the subsequent section

	• Line 93: Figure 1 (found on pg. 32 of PDF) – missing information in square boxes?, Top left oval box could be organized a bit more clearly<br>• Line 115-116: are these the eligibility criteria of the study participants?<br>o What makes participants ineligible?<br>• Line 126: Capitalize Declaration<br>• Line 128-129: Clarify this sentence<br>• Line 152: Figure 2 (found on pg. 32 of PDF) – no label for HCW but label for HCP which is not in the boxes; boxes with HCW letters are spelled differently in either box; FGD box on the left spelled FDG<br>• Line 155: title the section quantitative analysis to maintain formatting with next section<br>• Line 162: Change analysis to analyses<br>• Line 248: Figure 3 (found on pg. 33 on PDF) – "Norms stigma seasons" are these individual themes in this section?	Analysis has been changes to analyses
	Results<br>• Overall: fully inclusive results section with both quant and qual data<br>• Authors include exclusion criteria here in the quant section. Maybe consider moving up or also including in methods?<br>• Line 200: Says they included 427 participants? Differs from Abstract. Please confirm which one is correct<br>• Page 11: Table 1 could be organized a bit more clearly/create subsections for each section. May just be because of the formatting change.<br>• Line 233: Capitalize T in Table 2.	The number of included participants have been corrected to 425 thank you<br><br>Table 1 has been re-formatted for clarity. The variables are now readily identifiable<br><br>T in table 2 has been capitalised
	Discussion<br>• Overall: makes appropriate connections to quant and qual results and other studies that have been reviewed with individual components<br>• Line 380: remove second "had"	This sentence has been rephrased. Thank you.
	Conclusion	

	• Pulls everything together, does not over-emphasize any of the results/data	
	additional requirements.<br><br>1. Please ensure that your manuscript meets PLOS ONE's style requirements, including those for file naming. The PLOS ONE style templates can be found at https://journals.plos.org/plosone/s/file?id=wjVg/PLOSOne_formatting_sample_main_body.pdf and https://journals.plos.org/plosone/s/file?id=ba62/PLOSOne_formatting_sample_title_authors_affiliations.pdf	This has been done.
	2. Thank you for stating the following in the Acknowledgments Section of your manuscript:<br><br>RL is supported by the UMC Utrecht Global Health Support PhD programme. It had no role in the study design, collection, analysis, interpretation of data, writing of the report or decision to submit the article for publication.<br><br>We note that you have provided funding information that is not currently declared in your Funding Statement. However, funding information should not appear in the Acknowledgments section or other areas of your manuscript. We will only publish funding information present in the Funding Statement section of the online submission form.<br>Please remove any funding-related text from the manuscript and let us know how you would like to update your Funding Statement. Currently, your Funding Statement reads as follows:<br><br>The author(s) received no specific funding for this work.<br><br>Please include your amended statements within your cover letter; we will change	We have updated our funding statement .

	the online submission form on your behalf.	
	3. We note that you have stated that you will provide repository information for your data at acceptance. Should your manuscript be accepted for publication, we will hold it until you provide the relevant accession numbers or DOIs necessary to access your data. If you wish to make changes to your Data Availability statement, please describe these changes in your cover letter and we will update your Data Availability statement to reflect the information you provide.	This is well noted with thanks
	4a. Please include captions for your Supporting Information files at the end of your manuscript,<br><br>4b.and update any in-text citations to match accordingly. Please see our Supporting Information guidelines for more information: http://journals.plos.org/plosone/s/supporting-information.	This is not applicable thank you<br><br>This has been done . thank you

We trust that you will find our revised paper suitable for publication in Plos One.

Sincere regards,

Dr Roberta Lamptey
On behalf of all co-authors
roberta.lamptey@yahoo.com
