# Supplementary material for: Diabetes self-management education interventions and self-management in low-resource settings; a mixed methods study
Source: PLoS One. 2023 Jul 14;18(7):e0286974. doi: 10.1371/journal.pone.0286974 (PMC10348576; doi:10.1371/journal.pone.0286974)
Supplement: Attachment — Submitted filename: REVIEW REPORT 2.docx [file pone.0286974.s020.docx]

Thank you for the opportunity to review this paper. It is a very interesting and well written paper. It addresses a very important topic.

I provide my suggestions below which the authors may use for further improvement.

1. Abstract: Lines 50-52, the authors may revise the statement to include the percentage increase in the sentence, then the CI and the range are put in parentheses.
2. The introduction is not sufficiently grounded in the literature. The authors have not demonstrated their knowledge of what has been done already on the topic. Also, they have not provided enough justification for a need for the study. What is the research gap? And why is the study important for policy and practice. Self-Management Education is a behavior change intervention that is lees known and implemented in Africa. Authors should therefore provide sufficient information so readers will not confuse it with the normal self-management education that is provided to patients with diabetes when they seek routine clinical care. The topic is about diabetes self-management education (DSME). What is this DSME? Such background information is necessary.
3. The diabetes prevalence rate of 26% among the adult population in Ghana attributed to Jie Li et al., 2018 (lines 68 and 69) as the source may be incorrect. Jie Li et al. were talking about the prevalence of cardiovascular disease (CVD) risk factors and 26.1% was attributed to diabetes mellitus. They were not talking about diabetes prevalence among adult population in Ghana. They were talking about diabetes mellitus being a risk factor of CVD.
4. Lines 70-78, the entire two paragraphs have no single citation. Are the assertions based on the authors opinion? If the answer is no, then they need to credit the sources.
5. I am a little bit confused about the study aim and the conclusion drawn. The aim of the study is to understand DSME needs of patients and care providers. The conclusion is that "Diabetes self-management education tailored to resource-constrained settings are needed". What exact DSME needs of patients and providers did they find? It is already a known fact that DSMEs are needed for patients so they could self-manage their conditions. If the authors want to justify the need of DSME for patients, then the study aim has to be reconstructed. Also, what is understanding DSME needs of providers? Knowledge? resources? Some clarity is needed. It should also be noted that self-management needs are not the same as self-management education needs. Self-management education is an intervention provided to patients to build their capacity to self-manage their disease or engage in effective self-care behaviors.
6. Authors need to be specific about the type of diabetes patients being studied. People living with diabetes (PLD) means all diabetes types including type 1. But from line 116, they have stated that PLD were 18 years and above and not known to have type 1 diabetes. This means they are taking about people with type 2 diabetes. This should be used instead of people living with diabetes, Gestational diabetes is also a form of diabetes. Thus, authors should be specific right from the outset of the paper about the specific type of diabetes patients being referred to.
7. Lines 261 and 262, authors state “PLD receive DSME from nurses, doctors, and or nutritionists. It is un-structured, didactic, group based and delivered in-person prior to consultations”. A clear distinction needs to be made between the diabetes education given to patients during routine clinical care and diabetes self-management education program, which is a behavior change intervention designed and delivered to improve patients' elf-efficacy in self-managing their conditions. Diabetes self-management education programs are underpinned by behavior change theories and models such as Bandura’s Self-Efficacy Theory. Thus, the routine education on self-care given to patients during clinical visits could not be classified as a self-management education program, although it is self-management education. Authors therefore need to be clear whether they are referring to self-management education given to patients during routine clinic visits or DSME which is a new model of diabetes care and which aims at empowering patients to engage in effective self-care behaviors. The majority of DSMEs are delivered in non-clinical settings and some are led by laypersons or peer educators (people with diabetes trained as educators).
8. This takes me back to lines 77 and 78. The statement that “Sustainability and by extension availability of DSME is influenced by patient-, provider- and facility-level factors” holds for DSMEs delivered in clinic-settings. In other settings, DSMEs are provided by NGOs and these have nothing to do with providers and facility-based factors. Some DSMEs are even delivered I churches. And it must be stated that the facilitator of DSME is not always a clinician. DSME is separate from clinical care. The current literature calls for its integration in routine clinical care.
9. Conclusion: The first sentence reads “Existing DSME services are under-resourced and there are no structured DSME programs available.” Are they talking about the two study sites or the entire country? I think being specific rathe than providing general statements will help.
10. Authors may do thorough proofreading as there are some identified typos in the work.
