# Supplementary material for: Diabetes self-management education interventions and self-management in low-resource settings; a mixed methods study
Source: PLoS One. 2023 Jul 14;18(7):e0286974. doi: 10.1371/journal.pone.0286974 (PMC10348576; doi:10.1371/journal.pone.0286974)
Supplement: Attachment — Submitted filename: Response to reviewers.docx [file pone.0286974.s021.docx]

31^st^ October, 2022

The Editorial Office

PLOS ONE

Dear Jamie Males,

**RESPONSE TO REVIEWERS’ COMMENTS**

**PONE-D-22-15096: DIABETES SELF-MANAGEMENT EDUCATION IN LOW-RESOURCE SETTINGS: A MIXED METHODS NEEDS ASSESSMENT OF PROVIDERS AND PEOPLE LIVING WITH DIABETES**

We are deeply indebted to the reviewers for their time. We highly appreciate the time you and the team of editors have already committed to helping us improve our paper. Please find a point-by-point response to each comment in the table below. Our paper has been revised with these responses.

We have also modified the funding statement as follows:

Funding

The study was funded in part by the UMC Utrecht Global Health Support PhD program. It had no role in the study design, collection, analysis, interpretation of data, writing of the report or decision to submit the article for publication.

| No | Reviewer’s comment | Response to reviewer’s comment |
| --- | --- | --- |
|  |  |  |
|  | 1. Is the manuscript technically sound, and do the data support the conclusions?  The manuscript must describe a technically sound piece of scientific research with data that supports the conclusions. Experiments must have been conducted rigorously, with appropriate controls, replication, and sample sizes. The conclusions must be drawn appropriately based on the data presented.  Reviewer #1: No  Reviewer #2: Partly  Reviewer #3: Partly | Thank you.  We have reviewed the entire manuscript for clarity in communicating our processes and procedures. We were meticulous in following research methods, and we believe our work is technically sound. |
|  | 2. Has the statistical analysis been performed appropriately and rigorously?  Reviewer #1: I Don't Know  Reviewer #2: Yes  Reviewer #3: I Don't Know | We believe our analyses have been conducted appropriately and with rigour. |
|  | 3. Have the authors made all data underlying the findings in their manuscript fully available?  The [PLOS Data policy](http://www.plosone.org/static/policies.action#sharing) requires authors to make all data underlying the findings described in their manuscript fully available without restriction, with rare exception (please refer to the Data Availability Statement in the manuscript PDF file). The data should be provided as part of the manuscript or its supporting information, or deposited to a public repository. For example, in addition to summary statistics, the data points behind means, medians and variance measures should be available. If there are restrictions on publicly sharing data—e.g. participant privacy or use of data from a third party—those must be specified.  Reviewer #1: Yes  Reviewer #2: Yes  Reviewer #3: Yes | -- |
|  | 4. Is the manuscript presented in an intelligible fashion and written in standard English?  PLOS ONE does not copyedit accepted manuscripts, so the language in submitted articles must be clear, correct, and unambiguous. Any typographical or grammatical errors should be corrected at revision, so please note any specific errors here.  Reviewer #1: No  Reviewer #2: Yes  Reviewer #3: Yes | In response to reviewer 1, We have further reviewed the entire manuscript to ensure that the language used is suitable for a scientific paper. We have endeavoured to correct all language use errors. |
|  | Reviewer 1 |  |
|  | Reviewer #1: a. I applaud the authors for conducting an interesting article. However, is still needed to improve the quality of this paper. Please revise the manuscript to address the expressed concerns. After thorough review, I am recommending some revisions. In this regard, kindly address the following comments and suggestions to further improve your manuscript | Thank you. |
|  | b. There are some spelling and grammatical errors in the text. Please correct them | We have reviewed the manuscript and endeavoured to correct all language use errors. |
|  | c. Explain about the qualitative method in the abstract method section and mention the important qualitative results in the abstract results section | These were previously done. In our original submission  we stated the following:  1.A convergent parallel—This explains the mixed method both qualitative and quantitative. In response to the reviewer’s comment, in addition to the reference already provided, and Figure 1, we have modified the section 2.1 design and included this further explanation:  “Thus, we merged the two research methods (quantitative and qualitative) to answer our research questions and achieve our study aims. In addition, the two methods converged at the point of analysing the results and interpretating the data. Data for the quantitative study and qualitative study were collected simultaneously, in parallel. Moreover, we placed equal emphasis on qualitative and quantitative data in all aspects of the study.”  We believe this increases the reproducibility of our method. Thank you.  2. In the abstract we stated that “we employed inductive content analysis of informants’ experiences and context”.  In the main manuscript we explained further by stating “Data was analysed independently by RL, BB and a research assistant using an inductive thematic approach manually”-These explain the qualitative method  2.In the abstract we stated, “Financial constraints, conflicting messages, beliefs, and stigma were themes underpinning behaviours.”- These are the quantitative results  We then went on to describe the qualitative results in detail in the main manuscript. |
|  | d. Please write the type of study, sample size, sampling strategy and date and country of study in abstract | These were previously done.  We stated the following in our original submission:  1. A convergent parallel mixed-methods study was conducted . We went on to describe this method further in the main manuscript. In response to the reviewers comment we have provided further and better particulars of this type of study in addition to the reference already provided  2. sample size: In total 425 PLD…..  Two managers, five professionals, two diabetes experts and 16 PLD participated in in-depth interviews. Finally, 24 PLD  3. We have now provided further clarity to our sampling strategy.  , a total enumeration of all eligible clients seen at both study sites from December 2020 to January 2021 was done. **These dates formed the frame and we included everyone within the frame, who met the eligibility criteria.** **The attendance records of each study site were used in retrieving the relevant information on potential participants.** “  “PLD were identified through convenient sampling and snowballing for the qualitative study. Managers and healthcare professionals (HCPs) were sampled purposively, and judgemental sampling were used in identifying experts.”  4.date and country of the study  Thank you we have included this “January to February 2021 in Accra, Ghana” |
|  | e. The introduction section need some revision. You could summarize this section a bit more for readers. Write about the problems, the novelty of your study, the limitations of prior research might also be mentioned by the authors as further support for their present investigation and your study goals within the introduction. In this section, you can use the following articles: 1- “ Application of the Social Cognitive Theory to Predict Self-Care Behavior among Type 2 Diabetes Patients with Limited Health Literacy” 2- “The Relationship Between Health Literacy and Health Promoting Behaviors in Patients with Type2 Diabetes” | We have re-written the entire introduction section.  We have modified our title to ensure that the title, new introduction, aims, and conclusions are congruent  We have found it useful to include the suggested references in the introduction and discussion sections of the manuscript respectively.  Thank you for helping us improve our manuscript. |
|  | f. In the introduction, you should fully explain why you used the qualitative method? | This was previously done.  We stated that “We employed qualitative methods to deepen our understanding (of generalizable) outcomes from the quantitative study” |
|  | g. The materials & methods section is relatively immature. You could expand it a bit more clearly for readers. For example, Where have you collected samples? Write the year and the name of place in which you had done this survey. Furthermore, write about all applied exclusion and inclusion criteria a bit more clearly by which you selected samples for this survey. | These were previously done  1. Name and place where we did the study : We stated that “The study was conducted in Korle Bu Teaching Hospital polyclinic (KBTH) and Weija Gbawe Municipal hospital (WGMH),  2.where samples were collected: two public primary facilities located in Accra, Ghana.  Interviews were conducted at the study sites either in offices or large open spaces whilst observing prescribed COVID-19 protocols. Experts were interviewed virtually.  ”  3. year study conducted: We also stated that “Participant recruitment and data collection occurred between January and February 2021”  4. applied exclusion and inclusion criteria: We also stated that “HCP and PLD were staff and attendants at the study sites respectively. Managers were the respective heads. PLD were 18 years or older, not known to have type 1 diabetes, cognitive or psychiatric impairment and ambulant.  ” |
|  | h. Discuss more about your sampling strategy in both qualitative and quantitative section? The structure of your sampling is so vague and understandable. Did you have sampling frame? how did you access to this frame | This was previously done.  We stated that “a total enumeration of all eligible clients seen at both study sites from December 2020 to January 2021 was done.” For clarity we have added this sentence: “These dates formed the frame and we included everyone within the frame, who met the eligibility criteria “ |
|  | What are the data extract’s center characteristics? is it governmental or private, is it referral or not referral and so on, discuss more about it | This was previously done.  We stated that “The study was conducted in Korle Bu Teaching Hospital polyclinic (KBTH) and Weija Gbawe Municipal hospital (WGMH), two public primary facilities located in Accra, Ghana”. The facilities were government primary care facilities |
|  | j. How many observers did you have? if you had more than one observer, you must mention agreement | Thank you. We mentioned in our original submission that discrepancies were resolved through dialogue. We had 3 observers engaging the PLD . One observer engaged both managers  2 observers engaged the HCW.  2 observers engaged the Experts. |
|  | k. The methods need to be improved by providing more detail information related to participant’s selection (e.g. respond rate; necessary permissions from who? How did the researcher contact the potential participants?) | Response rate: We have now included the non-response rate “21%”  Permissions: We had stated the following in our original submission”The head of each facility granted permission for the study after having obtained ethical clearance”  We previously stated the following in our original submission “Trained staff called all potential participants meeting eligibility criteria and invited them to participate. For each individual, three attempts were made to reach them.” |
|  | l. Please prepare a method section based on consolidated criteria for reporting qualitative research (COREQ) guidelines | This was previously done . We stated in our original submission that “The Good Reporting of a Mixed-Methods Study (GRAMMS)(5) and Consolidated Criteria for REporting Qualitative research (COREQ)(6) checklists were followed.  ”  In response to the reviewer, we now have added the COREQ checklist as supporting material.” Thank you |
|  | m. Please write the type of Qualitative study (for example Grounded Theory, Content analysis, …) sample size, sampling strategy in qualitative method section | This was previously done in the abstract as well as in the main manuscript.  We stated the following and provided details of our method  1.”using an inductive content analysis”  2.“using an inductive thematic approach manually” |
|  | n. Sampling in qualitative section was done until redundancy in data was reached? the types and levels of participation of the participants should also be described | Thank you. The following statements have now been included “Our informants were fully engaged in all phases of our study. We selected participants who could best provide answers to our research question.” |
|  | o. The researcher's role(s), level of participation and relationship with participants also needs to be described in qualitative section, as they can influence the findings | Thank you . The following statement has been added “Some of the PLD recruited from the KBTH study site might have known RL as a staff of that facility. All other PLD involved in the study did not have any prior relationship with the data collectors. Experts and Health Care Professionals were colleagues of RL. The roles of the researchers were to facilitate the FGD and conduct the interviews” |
|  | p. You could increase the number of more recently studies in the reference section. You should have comprehensive and reliable comparisons between your findings with the other previous studies. In the discussion, you did not include related previous studies in relation to the findings of the current study. Please search and cite related studies and include them in your discussion | Our original discussion section included 14 references 9 which were published within the last 3 years and all the 14 references were published within the last 7 years.  We have in addition significantly increased the number of references in the introduction. Thank you. |
|  | q. In the discussion section, more interpretations are needed | This has been done . Thank you. We have done a thorough review of the entire discussion section |
|  | Reviewer #2: Thank you for the opportunity to review this paper. It is a very interesting and well written paper. It addresses a very important topic. I provide my suggestions below which the authors may use for further improvement. | Thank you |
|  | 1. Abstract: Lines 50-52, the authors may revise the statement to include the percentage increase in the sentence, then the CI and the range are put in parentheses. | We tried to rephrase the sentence to accommodate this suggestion however the sentence did not read very well . |
|  | 2. The introduction is not sufficiently grounded in the literature. The authors have not demonstrated their knowledge of what has been done already on the topic. Also, they have not provided enough justification for a need for the study. What is the research gap? And why is the study important for policy and practice. | We have increased the number of references in the introduction thank you.  The introduction has been re-written form clarity thank you. |
|  | Self-Management Education is a behavior change intervention that is lees known and implemented in Africa. Authors should therefore provide sufficient information so readers will not confuse it with the normal self-management education that is provided to patients with diabetes when they seek routine clinical care. The topic is about diabetes self-management education (DSME). What is this DSME? Such background information is necessary. | Additional background information has been provided in the introduction for clarity. We have also replaced self-management program with self-management intervention. Thank you |
|  | 3. The diabetes prevalence rate of 26% among the adult population in Ghana attributed to Jie Li et al., 2018 (lines 68 and 69) as the source may be incorrect. Jie Li et al. were talking about the prevalence of cardiovascular disease (CVD) risk factors and 26.1% was attributed to diabetes mellitus. They were not talking about diabetes prevalence among adult population in Ghana. They were talking about diabetes mellitus being a risk factor of CVD. | Thank you this has now been corrected to 6.5% prevalence. |
|  | 4. Lines 70-78, the entire two paragraphs have no single citation. Are the assertions based on the authors opinion? If the answer is no, then they need to credit the sources. | Thank you. This entire section has been re-written |
|  | 5. I am a little bit confused about the study aim and the conclusion drawn. The aim of the study is to understand DSME needs of patients and care providers. The conclusion is that "Diabetes self-management education tailored to resource-constrained settings are needed". What exact DSME needs of patients and providers did they find? It is already a known fact that DSMEs are needed for patients so they could self-manage their conditions. If the authors want to justify the need of DSME for patients, then the study aim has to be reconstructed. Also, what is understanding DSME needs of providers? Knowledge? resources? Some clarity is needed. It should also be noted that self-management needs are not the same as self-management education needs. Self-management education is an intervention provided to patients to build their capacity to self-manage their disease or engage in effective self-care behaviors. | The title, study aim, and conclusions have been re-written for clarity and is now more focused and congruent. Thank you |
|  | 6. Authors need to be specific about the type of diabetes patients being studied. People living with diabetes (PLD) means all diabetes types including type 1. But from line 116, they have stated that PLD were 18 years and above and not known to have type 1 diabetes. This means they are taking about people with type 2 diabetes. This should be used instead of people living with diabetes, Gestational diabetes is also a form of diabetes. Thus, authors should be specific right from the outset of the paper about the specific type of diabetes patients being referred to. | There is a fine line between various types of diabetes and often it is clinically difficult to distinguish between for example latent autoimmune diabetes in adults and type 2 diabetes. Given that we did not do formal testing we decided it was best to avoid classifying patients as type 2 diabetes. The inclusion criteria was self-reported diabetes and we excluded those known to have type 1 diabetes. We have now included this in our limitation “our findings may not be generalised to people known to have type 1 diabetes” |
|  | 7. Lines 261 and 262, authors state “PLD receive DSME from nurses, doctors, and or nutritionists. It is un-structured, didactic, group based and delivered in-person prior to consultations”. A clear distinction needs to be made between the diabetes education given to patients during routine clinical care and diabetes self-management education program, which is a behavior change intervention designed and delivered to improve patients' elf-efficacy in self-managing their conditions. Diabetes self-management education programs are underpinned by behavior change theories and models such as Bandura’s Self-Efficacy Theory. Thus, the routine education on self-care given to patients during clinical visits could not be classified as a self-management education program, although it is self-management education. Authors therefore need to be clear whether they are referring to self-management education given to patients during routine clinic visits or DSME which is a new model of diabetes care and which aims at empowering patients to engage in effective self-care behaviors. The majority of DSMEs are delivered in non-clinical settings and some are led by laypersons or peer educators (people with diabetes trained as educators). | Thank you we have replaced dsme program with dsme intervention throughout the manuscript.. |
|  | 8. This takes me back to lines 77 and 78. The statement that “Sustainability and by extension availability of DSME is influenced by patient-, provider- and facility-level factors” holds for DSMEs delivered in clinic-settings. In other settings, DSMEs are provided by NGOs and these have nothing to do with providers and facility-based factors. Some DSMEs are even delivered I churches. And it must be stated that the facilitator of DSME is not always a clinician. DSME is separate from clinical care. The current literature calls for its integration in routine clinical care. | Thank-you. This statement has been modified We have now specified facility-based DSME.  “Additionally, sustainability of facility-based structured DSME interventions are influenced by facility-, patient-, and provider level factors.[13]” |
|  | . Conclusion: The first sentence reads “Existing DSME services are under-resourced and there are no structured DSME programs available.” Are they talking about the two study sites or the entire country? I think being specific rathe than providing general statements will help. | Thank you  We have re-written the entire concluding paragraph . Those findings are limited to the two study sites.  “The DSME interventions studied were under-resourced and were not structured” |
|  | 10. Authors may do thorough proofreading as there are some identified typos in the work. | Thank you this has been done |
|  | Reviewer #3: I would like to thank the authors for their important work on assessing management of diabetes and educational needs in LMICs, this is a critical topic and this work is an important contribution to the field. The research question and methods need to be articulated clearly from the beginning however, the rest of the manuscript is clear. | Thank you |
|  | Abstract • Overall, Adequately described • Clarify aim regarding what exactly will you be studying from the provider group • Line 41: Designate the location of the study as Accra, Ghana | The aim has been re-stated for clarity and the study location included in the Abstract  “We sought to characterise DSME interventions in two urban low-resource primary settings, and to explore diabetes self-management knowledge and behaviours of persons living with diabetes (PLD).  ” |
|  | Introduction • Overall: a brief overview of diabetes self-management (DSME) • Minor grammatical errors • Authors should clarify early on that this study is reviewing the existing DSME in Accra, Ghana in two hospitals and its beliefs of and impact on patients, experts, and health care providers (HCPs). This should clearly be stated at the end of the introduction as the research question. • Similarly, the methods do not describe the exisiting DSME intervention as what is being studied o This is in the results section◊ existing DSME programs that are unstructured and they are reviewing whether the programs were are helpful to the patients, HCP, and experts - -- tlead with this and that you are observing its efficacy, usage, and public reaction • Line 81: Designate the location with the city - Accra, Ghana | The entire introduction has been re-written for clarity and to improve congruency with the other sections of the manuscript thank you.  The aim has been re-stated at the end of the introduction  Our aim was rather to describe and characterise the existing DSME interventions  The location within the city has been designated |
|  | Methods • Overall: Provided a thorough list of methods with minimal grammatical errors and information regarding ethical considerations • Actual intervention being studied is missing, it is critical to describe what is being evaluated so the reader understands the setting. Thorough descriptions of necessary sample size and methods of recruitment is there but what is being assessed is not included here and needs to be. • Ethical considerations found in Data collection section: written informed consent, non-disclosure statements; codes assigned to maintain confidentiality • Transcripts and analysis were shared with informants to check for accuracy and provide feedback- sounds excellent! • Ethical approval: IRB from KBTH and Ethics review committee of Ghana Health Service Line Edits • Line 93: Figure 1 (found on pg. 32 of PDF) – missing information in square boxes?, Top left oval box could be organized a bit more clearly • Line 115-116: are these the eligibility criteria of the study participants? o What makes participants ineligible? • Line 126: Capitalize Declaration • Line 128-129: Clarify this sentence • Line 152: Figure 2 (found on pg. 32 of PDF) – no label for HCW but label for HCP which is not in the boxes; boxes with HCW letters are spelled differently in either box; FGD box on the left spelled FDG • Line 155: title the section quantitative analysis to maintain formatting with next section • Line 162: Change analysis to analyses • Line 248: Figure 3 (found on pg. 33 on PDF) – “Norms stigma seasons” are these individual themes in this section? | Our aim was rather to describe and characterise the existing DSME interventions. The aim has been re-written. Thank you  The abbreviations in the Figures have been corrected. Thank you.  The section on eligibility criteria has now been clearly labelled.  Figure 1 reorganised as suggested  Figure 2- Abreviations have been corrected  Figure 3 has been re-drawn; the major theme in that circle is stigma  The section on quantitative analysis has been titled to maintain formatting with the subsequent section  Analysis has been changes to analyses |
|  | Results • Overall: fully inclusive results section with both quant and qual data • Authors include exclusion criteria here in the quant section. Maybe consider moving up or also including in methods? • Line 200: Says they included 427 participants? Differs from Abstract. Please confirm which one is correct • Page 11: Table 1 could be organized a bit more clearly/create subsections for each section. May just be because of the formatting change. • Line 233: Capitalize T in Table 2. | The number of included participants have been corrected to 425 thank you  Table 1 has been re-formatted for clarity. The variables are now readily identifiable  T in table 2 has been capitalised |
|  | Discussion • Overall: makes appropriate connections to quant and qual results and other studies that have been reviewed with individual components • Line 380: remove second "had" | This sentence has been rephrased. Thank you. |
|  | Conclusion • Pulls everything together, does not over-emphasize any of the results/data |  |
|  | additional requirements.  1. Please ensure that your manuscript meets PLOS ONE's style requirements, including those for file naming. The PLOS ONE style templates can be found at  https://journals.plos.org/plosone/s/file?id=wjVg/PLOSOne_formatting_sample_main_body.pdf and  https://journals.plos.org/plosone/s/file?id=ba62/PLOSOne_formatting_sample_title_authors_affiliations.pdf | This has been done. |
|  | 2. Thank you for stating the following in the Acknowledgments Section of your manuscript:  RL is supported by the UMC Utrecht Global Health Support PhD programme. It had no role in the study design, collection, analysis, interpretation of data, writing of the report or decision to submit the article for publication.  We note that you have provided funding information that is not currently declared in your Funding Statement. However, funding information should not appear in the Acknowledgments section or other areas of your manuscript. We will only publish funding information present in the Funding Statement section of the online submission form.  Please remove any funding-related text from the manuscript and let us know how you would like to update your Funding Statement. Currently, your Funding Statement reads as follows:  The author(s) received no specific funding for this work.  Please include your amended statements within your cover letter; we will change the online submission form on your behalf. | We have updated our funding statement . |
|  | 3. We note that you have stated that you will provide repository information for your data at acceptance. Should your manuscript be accepted for publication, we will hold it until you provide the relevant accession numbers or DOIs necessary to access your data. If you wish to make changes to your Data Availability statement, please describe these changes in your cover letter and we will update your Data Availability statement to reflect the information you provide. | This is well noted with thanks |
|  | 4a. Please include captions for your Supporting Information files at the end of your manuscript,  4b.and update any in-text citations to match accordingly. Please see our Supporting Information guidelines for more information: http://journals.plos.org/plosone/s/supporting-information. | This is not applicable thank you  This has been done . thank you |

We trust that you will find our revised paper suitable for publication in Plos One.

Sincere regards,

Dr Roberta Lamptey

On behalf of all co-authors

roberta.lamptey@yahoo.com
