# Supplementary material for: Diabetes self-management education interventions and self-management in low-resource settings; a mixed methods study
Source: PLoS One. 2023 Jul 14;18(7):e0286974. doi: 10.1371/journal.pone.0286974 (PMC10348576; doi:10.1371/journal.pone.0286974)
Supplement: Attachment — Submitted filename: 30-03 Response to reviewers.docx [file pone.0286974.s023.docx]

31^st^ March, 2023

The Editorial Office

PLOS ONE

Dear Margaret Williams,

**RESPONSE TO EDITOR’S COMMENTS**

**PONE-D-22-15096: DIABETES SELF-MANAGEMENT EDUCATION IN LOW-RESOURCE SETTINGS: A MIXED METHODS NEEDS ASSESSMENT OF PROVIDERS AND PEOPLE LIVING WITH DIABETES**

We are deeply indebted to the reviewers for their time. We highly appreciate the time you and the team of editors have already committed to helping us improve our paper. Please find a point-by-point response to each comment in the table below. Our paper has been revised with these responses.

| Editor’s Comment | Response to comment |
| --- | --- |
| I agree with the reviewers that this article should be published in PLOS ONE. | Thank you for this feedback |
| I also agree that the manuscript requires the attention of an editor. I note that there is an attempt at language editing but there are numerous basic errors that require attention, sentence structure and punctuation. | Thank you for drawing our attention to this. We have edited the entire paper and we have endeavoured to correct all language use errors. |
| The reviewers have highlighted one or two other areas of concern, one of which was a review of the statistical analysis. In my opinion the statistical analysis is adequate. | Thank you |

We sincerely appreciate your feedback and believe our paper reads much better.

We trust that you will find our revised paper suitable for publication in Plos One.

Sincere regards,

Dr Roberta Lamptey

On behalf of all co-authors

roberta.lamptey@yahoo.com
