# Supplementary material for: Diabetes self-management education interventions and self-management in low-resource settings; a mixed methods study
Source: PLoS One. 2023 Jul 14;18(7):e0286974. doi: 10.1371/journal.pone.0286974 (PMC10348576; doi:10.1371/journal.pone.0286974)
Supplement: Peer review history

# Peer review: Decision Letter 0

**DOI:** 10.1371/journal.pone.0286974.r001

**Authors:** Jamie Males

19 Sep 2022

PONE-D-22-15096DIABETES SELF-MANAGEMENT EDUCATION IN LOW-RESOURCE SETTINGS: A MIXED METHODS NEEDS ASSESSMENT OF PROVIDERS AND PEOPLE LIVING WITH DIABETESPLOS ONE

Dear Dr. Lamptey,

Thank you for submitting your manuscript to PLOS ONE. After careful consideration, we feel that it has merit but does not fully meet PLOS ONE’s publication criteria as it currently stands. Therefore, we invite you to submit a revised version of the manuscript that addresses the points raised during the review process.

Three external reviewers have now evaluated your submission. They have identified a number of concerns that need to be addressed, including a need for important clarifications about the study design and methods. Please refer to the reviewers' comments below for further detail, and ensure that you respond carefully to all of the points they have raised when preparing your revision.

Please submit your revised manuscript by Nov 01 2022 11:59PM. If you will need more time than this to complete your revisions, please reply to this message or contact the journal office at plosone@plos.org. When you're ready to submit your revision, log on to https://www.editorialmanager.com/pone/ and select the 'Submissions Needing Revision' folder to locate your manuscript file.

Please include the following items when submitting your revised manuscript:

- A rebuttal letter that responds to each point raised by the academic editor and reviewer(s). You should upload this letter as a separate file labeled 'Response to Reviewers'.
- A marked-up copy of your manuscript that highlights changes made to the original version. You should upload this as a separate file labeled 'Revised Manuscript with Track Changes'.
- An unmarked version of your revised paper without tracked changes. You should upload this as a separate file labeled 'Manuscript'.

If you would like to make changes to your financial disclosure, please include your updated statement in your cover letter. Guidelines for resubmitting your figure files are available below the reviewer comments at the end of this letter.

If applicable, we recommend that you deposit your laboratory protocols in protocols.io to enhance the reproducibility of your results. Protocols.io assigns your protocol its own identifier (DOI) so that it can be cited independently in the future. For instructions see: https://journals.plos.org/plosone/s/submission-guidelines#loc-laboratory-protocols. Additionally, PLOS ONE offers an option for publishing peer-reviewed Lab Protocol articles, which describe protocols hosted on protocols.io. Read more information on sharing protocols at https://plos.org/protocols?utm_medium=editorial-email&utm_source=authorletters&utm_campaign=protocols.

We look forward to receiving your revised manuscript.

Kind regards,

Jamie Males

Editorial Office

PLOS ONE

Journal Requirements:

When submitting your revision, we need you to address these additional requirements.

1. Please ensure that your manuscript meets PLOS ONE's style requirements, including those for file naming. The PLOS ONE style templates can be found at 

https://journals.plos.org/plosone/s/file?id=wjVg/PLOSOne_formatting_sample_main_body.pdf and 

https://journals.plos.org/plosone/s/file?id=ba62/PLOSOne_formatting_sample_title_authors_affiliations.pdf

2. Thank you for stating the following in the Acknowledgments Section of your manuscript: 

RL is supported by the UMC Utrecht Global Health Support PhD programme. It had no role in the study design, collection, analysis, interpretation of data, writing of the report or decision to submit the article for publication. 

We note that you have provided funding information that is not currently declared in your Funding Statement. However, funding information should not appear in the Acknowledgments section or other areas of your manuscript. We will only publish funding information present in the Funding Statement section of the online submission form. 

Please remove any funding-related text from the manuscript and let us know how you would like to update your Funding Statement. Currently, your Funding Statement reads as follows: 

The author(s) received no specific funding for this work.

Please include your amended statements within your cover letter; we will change the online submission form on your behalf.

3. We note that you have stated that you will provide repository information for your data at acceptance. Should your manuscript be accepted for publication, we will hold it until you provide the relevant accession numbers or DOIs necessary to access your data. If you wish to make changes to your Data Availability statement, please describe these changes in your cover letter and we will update your Data Availability statement to reflect the information you provide.

4. Please include captions for your Supporting Information files at the end of your manuscript, and update any in-text citations to match accordingly. Please see our Supporting Information guidelines for more information: http://journals.plos.org/plosone/s/supporting-information. 

[Note: HTML markup is below. Please do not edit.]

Reviewers' comments:

Reviewer's Responses to Questions

**Comments to the Author**

1. Is the manuscript technically sound, and do the data support the conclusions?

The manuscript must describe a technically sound piece of scientific research with data that supports the conclusions. Experiments must have been conducted rigorously, with appropriate controls, replication, and sample sizes. The conclusions must be drawn appropriately based on the data presented. 

Reviewer #1: No

Reviewer #2: Partly

Reviewer #3: Partly

**********

2. Has the statistical analysis been performed appropriately and rigorously? 

Reviewer #1: I Don't Know

Reviewer #2: Yes

Reviewer #3: I Don't Know

**********

3. Have the authors made all data underlying the findings in their manuscript fully available?

The PLOS Data policy requires authors to make all data underlying the findings described in their manuscript fully available without restriction, with rare exception (please refer to the Data Availability Statement in the manuscript PDF file). The data should be provided as part of the manuscript or its supporting information, or deposited to a public repository. For example, in addition to summary statistics, the data points behind means, medians and variance measures should be available. If there are restrictions on publicly sharing data—e.g. participant privacy or use of data from a third party—those must be specified.

Reviewer #1: Yes

Reviewer #2: Yes

Reviewer #3: Yes

**********

4. Is the manuscript presented in an intelligible fashion and written in standard English?

PLOS ONE does not copyedit accepted manuscripts, so the language in submitted articles must be clear, correct, and unambiguous. Any typographical or grammatical errors should be corrected at revision, so please note any specific errors here.

Reviewer #1: No

Reviewer #2: Yes

Reviewer #3: Yes

**********

5. Review Comments to the Author

Please use the space provided to explain your answers to the questions above. You may also include additional comments for the author, including concerns about dual publication, research ethics, or publication ethics. (Please upload your review as an attachment if it exceeds 20,000 characters)

Reviewer #1: a. I applaud the authors for conducting an interesting article. However, is still needed to improve the quality of this paper. Please revise the manuscript to address the expressed concerns. After thorough review, I am recommending some revisions. In this regard, kindly address the following comments and suggestions to further improve your manuscript

b. There are some spelling and grammatical errors in the text. Please correct them

c. Explain about the qualitative method in the abstract method section and mention the important qualitative results in the abstract results section

d. Please write the type of study, sample size, sampling strategy and date and country of study in abstract

e. The introduction section need some revision. You could summarize this section a bit more for readers. Write about the problems, the novelty of your study, the limitations of prior research might also be mentioned by the authors as further support for their present investigation and your study goals within the introduction. In this section, you can use the following articles:

1- “ Application of the Social Cognitive Theory to Predict Self-Care Behavior among Type 2 Diabetes Patients with Limited Health Literacy”

2- “The Relationship Between Health Literacy and Health Promoting Behaviors in Patients with Type2 Diabetes”

f. In the introduction, you should fully explain why you used the qualitative method?

g. The materials & methods section is relatively immature. You could expand it a bit more clearly for readers. For example, Where have you collected samples? Write the year and the name of place in which you had done this survey. Furthermore, write about all applied exclusion and inclusion criteria a bit more clearly by which you selected samples for this survey.

h. Discuss more about your sampling strategy in both qualitative and quantitative section? The structure of your sampling is so vague and understandable. Did you have sampling frame? how did you access to this frame

i. What are the data extract’s center characteristics? is it governmental or private, is it referral or not referral and so on, discuss more about it

j. How many observers did you have? if you had more than one observer, you must mention agreement

k. The methods need to be improved by providing more detail information related to participant’s selection (e.g. respond rate; necessary permissions from who? How did the researcher contact the potential participants?)

l. Please prepare a method section based on consolidated criteria for reporting qualitative research (COREQ) guidelines

m. Please write the type of Qualitative study (for example Grounded Theory, Content analysis, …) sample size, sampling strategy in qualitative method section

n. Sampling in qualitative section was done until redundancy in data was reached? the types and levels of participation of the participants should also be described

o. The researcher's role(s), level of participation and relationship with participants also needs to be described in qualitative section, as they can influence the findings

p. You could increase the number of more recently studies in the reference section. You should have comprehensive and reliable comparisons between your findings with the other previous studies. In the discussion, you did not include related previous studies in relation to the findings of the current study. Please search and cite related studies and include them in your discussion

q. In the discussion section, more interpretations are needed

Reviewer #2: Thank you for the opportunity to review this paper. It is a very interesting and well written paper. It addresses a very important topic.

I provide my suggestions below which the authors may use for further improvement.

1. Abstract: Lines 50-52, the authors may revise the statement to include the percentage increase in the sentence, then the CI and the range are put in parentheses.

2. The introduction is not sufficiently grounded in the literature. The authors have not demonstrated their knowledge of what has been done already on the topic. Also, they have not provided enough justification for a need for the study. What is the research gap? And why is the study important for policy and practice. Self-Management Education is a behavior change intervention that is lees known and implemented in Africa. Authors should therefore provide sufficient information so readers will not confuse it with the normal self-management education that is provided to patients with diabetes when they seek routine clinical care. The topic is about diabetes self-management education (DSME). What is this DSME? Such background information is necessary.

3. The diabetes prevalence rate of 26% among the adult population in Ghana attributed to Jie Li et al., 2018 (lines 68 and 69) as the source may be incorrect. Jie Li et al. were talking about the prevalence of cardiovascular disease (CVD) risk factors and 26.1% was attributed to diabetes mellitus. They were not talking about diabetes prevalence among adult population in Ghana. They were talking about diabetes mellitus being a risk factor of CVD.

4. Lines 70-78, the entire two paragraphs have no single citation. Are the assertions based on the authors opinion? If the answer is no, then they need to credit the sources.

5. I am a little bit confused about the study aim and the conclusion drawn. The aim of the study is to understand DSME needs of patients and care providers. The conclusion is that "Diabetes self-management education tailored to resource-constrained settings are needed". What exact DSME needs of patients and providers did they find? It is already a known fact that DSMEs are needed for patients so they could self-manage their conditions. If the authors want to justify the need of DSME for patients, then the study aim has to be reconstructed. Also, what is understanding DSME needs of providers? Knowledge? resources? Some clarity is needed. It should also be noted that self-management needs are not the same as self-management education needs. Self-management education is an intervention provided to patients to build their capacity to self-manage their disease or engage in effective self-care behaviors.

6. Authors need to be specific about the type of diabetes patients being studied. People living with diabetes (PLD) means all diabetes types including type 1. But from line 116, they have stated that PLD were 18 years and above and not known to have type 1 diabetes. This means they are taking about people with type 2 diabetes. This should be used instead of people living with diabetes, Gestational diabetes is also a form of diabetes. Thus, authors should be specific right from the outset of the paper about the specific type of diabetes patients being referred to.

7. Lines 261 and 262, authors state “PLD receive DSME from nurses, doctors, and or nutritionists. It is un-structured, didactic, group based and delivered in-person prior to consultations”. A clear distinction needs to be made between the diabetes education given to patients during routine clinical care and diabetes self-management education program, which is a behavior change intervention designed and delivered to improve patients' elf-efficacy in self-managing their conditions. Diabetes self-management education programs are underpinned by behavior change theories and models such as Bandura’s Self-Efficacy Theory. Thus, the routine education on self-care given to patients during clinical visits could not be classified as a self-management education program, although it is self-management education. Authors therefore need to be clear whether they are referring to self-management education given to patients during routine clinic visits or DSME which is a new model of diabetes care and which aims at empowering patients to engage in effective self-care behaviors. The majority of DSMEs are delivered in non-clinical settings and some are led by laypersons or peer educators (people with diabetes trained as educators).

8. This takes me back to lines 77 and 78. The statement that “Sustainability and by extension availability of DSME is influenced by patient-, provider- and facility-level factors” holds for DSMEs delivered in clinic-settings. In other settings, DSMEs are provided by NGOs and these have nothing to do with providers and facility-based factors. Some DSMEs are even delivered I churches. And it must be stated that the facilitator of DSME is not always a clinician. DSME is separate from clinical care. The current literature calls for its integration in routine clinical care.

9. Conclusion: The first sentence reads “Existing DSME services are under-resourced and there are no structured DSME programs available.” Are they talking about the two study sites or the entire country? I think being specific rathe than providing general statements will help.

10. Authors may do thorough proofreading as there are some identified typos in the work.

Reviewer #3: I would like to thank the authors for their important work on assessing management of diabetes and educational needs in LMICs, this is a critical topic and this work is an important contribution to the field. The research question and methods need to be articulated clearly from the beginning however, the rest of the manuscript is clear. Abstract

• Overall, Adequately described

• Clarify aim regarding what exactly will you be studying from the provider group

• Line 41: Designate the location of the study as Accra, Ghana

Introduction

• Overall: a brief overview of diabetes self-management (DSME)

• Minor grammatical errors

• Authors should clarify early on that this study is reviewing the existing DSME in Accra, Ghana in two hospitals and its beliefs of and impact on patients, experts, and health care providers (HCPs). This should clearly be stated at the end of the introduction as the research question.

• Similarly, the methods do not describe the exisiting DSME intervention as what is being studied

o This is in the results section� existing DSME programs that are unstructured and they are reviewing whether the programs were are helpful to the patients, HCP, and experts - -- tlead with this and that you are observing its efficacy, usage, and public reaction

• Line 81: Designate the location with the city - Accra, Ghana

Methods

• Overall: Provided a thorough list of methods with minimal grammatical errors and information regarding ethical considerations

• Actual intervention being studied is missing, it is critical to describe what is being evaluated so the reader understands the setting. Thorough descriptions of necessary sample size and methods of recruitment is there but what is being assessed is not included here and needs to be.

• Ethical considerations found in Data collection section: written informed consent, non-disclosure statements; codes assigned to maintain confidentiality

• Transcripts and analysis were shared with informants to check for accuracy and provide feedback- sounds excellent!

• Ethical approval: IRB from KBTH and Ethics review committee of Ghana Health Service

Line Edits

• Line 93: Figure 1 (found on pg. 32 of PDF) – missing information in square boxes?, Top left oval box could be organized a bit more clearly

• Line 115-116: are these the eligibility criteria of the study participants?

o What makes participants ineligible?

• Line 126: Capitalize Declaration

• Line 128-129: Clarify this sentence

• Line 152: Figure 2 (found on pg. 32 of PDF) – no label for HCW but label for HCP which is not in the boxes; boxes with HCW letters are spelled differently in either box; FGD box on the left spelled FDG

• Line 155: title the section quantitative analysis to maintain formatting with next section

• Line 162: Change analysis to analyses

• Line 248: Figure 3 (found on pg. 33 on PDF) – “Norms stigma seasons” are these individual themes in this section?

Results

• Overall: fully inclusive results section with both quant and qual data

• Authors include exclusion criteria here in the quant section. Maybe consider moving up or also including in methods?

• Line 200: Says they included 427 participants? Differs from Abstract. Please confirm which one is correct

• Page 11: Table 1 could be organized a bit more clearly/create subsections for each section. May just be because of the formatting change.

• Line 233: Capitalize T in Table 2.

Discussion

• Overall: makes appropriate connections to quant and qual results and other studies that have been reviewed with individual components

• Line 380: remove second "had"

Conclusion

• Pulls everything together, does not over-emphasize any of the results/data

**********

6. PLOS authors have the option to publish the peer review history of their article (what does this mean?). If published, this will include your full peer review and any attached files.

If you choose “no”, your identity will remain anonymous but your review may still be made public.

**Do you want your identity to be public for this peer review?** For information about this choice, including consent withdrawal, please see our Privacy Policy.

Reviewer #1: **Yes: **Dr. Hadi Tehrani

Reviewer #2: **Yes: **Dr. Emmanuel Kumah

Reviewer #3: No

**********

[NOTE: If reviewer comments were submitted as an attachment file, they will be attached to this email and accessible via the submission site. Please log into your account, locate the manuscript record, and check for the action link "View Attachments". If this link does not appear, there are no attachment files.]

While revising your submission, please upload your figure files to the Preflight Analysis and Conversion Engine (PACE) digital diagnostic tool, https://pacev2.apexcovantage.com/. PACE helps ensure that figures meet PLOS requirements. To use PACE, you must first register as a user. Registration is free. Then, login and navigate to the UPLOAD tab, where you will find detailed instructions on how to use the tool. If you encounter any issues or have any questions when using PACE, please email PLOS at figures@plos.org. Please note that Supporting Information files do not need this step.

Attachment Submitted filename: REVIEW REPORT 2.docx Click here for additional data file.

# Author comment: Author response to Decision Letter 0

**DOI:** 10.1371/journal.pone.0286974.r002

23 Jan 2023

We are highly indebted to the reviewers for their time. We deeply appreciate the time they have already committed to helping us improve our paper. We have uploaded a file with our point by point response in tabular form. We provide here a summary only.

We have reviewed the entire manuscript for clarity in communicating our processes and procedures. We were meticulous in our methods and we believe our work is technically sound. 

We believe our analyses have been conducted appropriately and with rigour.

We have reviewed the entire manuscript to ensure that the language used is suitable for a scientific paper. We have endeavoured to correct all language use errors.

In our original submission, we addressed these issues. Please find our itemised responses below:

1. We stated that we used a convergent parallel design, a recognised type of mixed methods design for which we provided a reference. We also depicted the design graphically with Fig 1. 

To explain the method in more detail we have now added this statement :

“Thus, we merged the two research methods (quantitative and qualitative) to answer our research questions and achieve our study aims. In addition, the two methods converged at the point of analysing the results, and interpretating the data. Data for the quantitative study and qualitative study were collected simultaneously, in parallel. Moreover, we placed equal emphasis on qualitative and quantitative data in all aspects of the study.” 

We believe this increases the reproducibility of our method. Thank you.

2. In the abstract we stated that “we employed inductive content analysis of informants’ experiences and context”. 

In the main manuscript we explained further by stating “Data was analysed independently by RL, BB and a research assistant using an inductive thematic approach manually”-These explain the qualitative method

2.In the abstract we stated, “Financial constraints, conflicting messages, beliefs, and stigma were themes underpinning behaviours.”- These themes are our quantitative results

We then went on to describe the qualitative results in detail in the main manuscript.

Thank you. In our original submission, we addressed these issues. Please find our itemised responses below:

:

Type of study

1. A convergent parallel mixed-methods study was conducted as earlier described

Sample size

2. sample size: In total 425 PLD…..

Two managers, five healthcare professionals, two diabetes experts and 16 PLD participated in in-depth interviews. Finally, 24 PLD participated in four FGD

Sampling strategy

3. sampling strategy: we stated the following in our original submission in the abstract “A total enumeration was done for the cross-sectional study whilst purposive or judgemental sampling was used in selecting participants for the qualitative study”.

4.date and country of the study

Thank you we have now included this “January to February 2021 in Accra, Ghana”

Thank you. We have re-written the entire introduction section.

We have modified our title to ensure that the title, new introduction, aims, and conclusions are congruent

Thank you. In our original submission, we provided an explanation for our choice. 

We stated that “We employed qualitative methods to deepen our understanding (of generalizable) outcomes from the quantitative study”

Thank you. In our original submission, we addressed these issues. Please find our itemised responses below:

1.where we collected samples for the study : We stated that “The study was conducted in Korle Bu Teaching Hospital polyclinic (KBTH) and Weija Gbawe Municipal hospital (WGMH), 

2. we stated that these two facilities were public primary care facilities located in Accra, Ghana.

 Interviews were conducted at the study sites either in offices or large open spaces whilst observing prescribed COVID-19 protocols. Experts were interviewed virtually. 

”

3. year study conducted

 We also stated that “Participant recruitment and data collection occurred between January and February 2021”

4.

 exclusion and inclusion criteria: We also stated that “HCP and PLD were staff and attendants at the study sites respectively. Managers were the respective heads. PLD were 18 years or older, not known to have type 1 diabetes, cognitive or psychiatric impairment and ambulant. 

” This section is now labelled clearly.

Thank you. In our original submission, we addressed these issues. Please find our itemised responses below:

We stated that “a total enumeration of all eligible clients seen at both study sites from December 2020 to January 2021 was done.” Thus the sampling strategy for the qualitative section was total enumeration.

We further explained that “Trained staff called all potential participants meeting eligibility criteria and invited them to participate.” This was how we accessed the sampling frame

We also stated that “PLD were identified through convenient sampling and snowballing for the qualitative study. Managers and healthcare professionals (HCPs) were sampled purposively, and judgemental sampling were used in identifying experts”. This explains the sampling method for the qualitative study.

Thank you. In our original submission, we addressed these issues. Please find our itemised responses below:

We stated that “The study was conducted in Korle Bu Teaching Hospital polyclinic (KBTH) and Weija Gbawe Municipal hospital (WGMH), two public primary facilities located in Accra, Ghana”. Thus, the facilities were government primary care facilities. They were not referral facilities.

In our original submission, we addressed these issues. Please find our itemised responses below:

Thank you. 

We mentioned in our original submission that discrepancies were resolved through dialogue. 

For the qualitative study, the interviews were one-on -one and for the FGD we had more than one facilitator per group including field note takers. 

Response rate

Thank you. we have now included the non-response rate “21%”

Permissions

We had stated the following in our original submission” The head of each facility granted permission for the study after having obtained ethical clearance”

Participant recruitment

We stated the following in our original submission “Trained staff called all potential participants meeting eligibility criteria and invited them to participate. For each individual, three attempts were made to reach them.”

In our original submission, we addressed these issues. Please find our itemised responses below:

Thank you. 

We stated in our original submission that “The Good Reporting of a Mixed-Methods Study (GRAMMS)(5) and Consolidated Criteria for REporting Qualitative research (COREQ)(6) checklists were followed.

”

In response to the reviewer’s comment, we now have added the COREQ checklist as supporting material.” Thank you

In our original submission, we addressed these issues. Please find our itemised responses below:

Thank you. 

We stated the following and provided details of our method

1.”using an inductive content analysis”

2.“using an inductive thematic approach manually”

Thank you. The following statements have now been included “Our informants were fully engaged in all phases of our study. We selected participants who could best provide answers to our research question.”

Thank you . The following statement has been added “Some of the PLD recruited from the KBTH study site might have known RL as a staff of that facility. All other PLD involved in the study did not have any prior relationship with the data collectors. Experts and Health Care Professionals were colleagues of RL. The roles of the researchers were to facilitate the FGD and conduct the interviews”

Our original discussion section included 14 references 9 which were published within the last 3 years and all the 14 references were published within the last 7 years.

We have in addition significantly increased the number of references in the introduction. Thank you.

2

We tried to rephrase the sentence to accommodate this suggestion however the sentence did not read well . We have therefore maintained the original sentence as is.

We have increased the number of references in the introduction thank you.

The entire introduction has been re-written form clarity thank you.

Additional background information has been provided in the introduction for clarity. We have also replaced self-management program with self-management intervention. Thank you

Thank you this has now been corrected by quoting a 6.5% prevalence.

Thank you. This entire section has been re-written

The title, study aim and conclusions have been re-written for clarity. Our manuscript is now more focused and congruent. Thank you

There is a fine line between various types of diabetes with some overlap, and often it is difficult to clinically distinguish between them. For example making a distinction between latent autoimmune diabetes in adults and type 2 diabetes or between type 1 diabetes in an adult and type 2 diabetes which is burnt out. Given that we did not do formal diagnostic testing e.g autoantibodies, c-peptide etc, we decided it was best to avoid classifying patients as type 2 diabetes. 

The inclusion criteria was self-reported diabetes and we excluded those known to have type 1 diabetes. We have now included this statement in our limitation “our findings may not be generalised to people known to have type 1 diabetes”

Thank you we have replaced DSME program with DSME intervention throughout the manuscript..

Thank-you. This statement has been modified We have now specified facility based DSME interventions.

“Additionally, sustainability of facility-based structured DSME interventions are influenced by facility-, patient-, and provider level factors.[13]”

Thank you

We have re-written the entire concluding paragraph . Those findings are limited to the two study sites.

“The DSME interventions studied were under-resourced and were not structured”

The aim has been re-stated for clarity and the study location included in the Abstract

“We sought to characterise DSME interventions in two urban low-resource primary settings, and to explore diabetes self-management knowledge and behaviours of persons living with diabetes (PLD). 

”

The entire introduction has been re-written for clarity and to improve congruency with the other sections of the manuscript thank you. 

The aim has been re-stated at the end of the introduction

Our aim was rather to describe and characterise the existing DSME interventions

The location within the city has been stated. “KBTH is located within the Ablekumah South Metropolitan district and WGMH is located in Ga West Municipal district.”

Our aim was rather to describe and characterise the existing DSME interventions. The aim has been re-written. Thank you

The abbreviations in the Figures have been corrected. Thank you.

The section on eligibility criteria has now been clearly labelled. 

Figure 1 has been reorganised as suggested

Figure 2- Abbreviations have been corrected 

Figure 3 has been re-drawn; the major theme in that circle is stigma

The section on quantitative analysis has been titled to maintain formatting with the subsequent section

Analysis has been changes to analyses

The number of included participants have been corrected to 425. Thank you

Table 1 has been re-formatted for clarity. The variables are now readily identifiable

T in table 2 has been capitalised

We have updated our funding statement . Our amended funding statement is as follows:

“This study was funded in part by the UMC Utrecht Global Health Support PhD program. It had no role in the study design, collection, analysis, interpretation of data, writing of the report or decision to submit the article for publication. 

”

Attachment Submitted filename: Response to reviewers.docx Click here for additional data file.

# Peer review: Decision Letter 1

**DOI:** 10.1371/journal.pone.0286974.r003

**Authors:** Margaret Williams

20 Mar 2023

PONE-D-22-15096R1Diabetes self-management education interventions and self-management in low-resource settings; a mixed methods studyPLOS ONE

Dear Dr. Lamptey,

Thank you for submitting your manuscript to PLOS ONE. After careful consideration, we feel that it has merit but does not fully meet PLOS ONE’s publication criteria as it currently stands. Therefore, we invite you to submit a revised version of the manuscript that addresses the points raised during the review process.

==============================I agree with the reviewers that this article should be published in PLOS ONE. I also agree that the manuscript requires the attention of an editor. I note that there is an attempt at language editing but there are numerous basic errors that require attention, sentence structure and punctuation. The reviewers have highlighted one or two other areas of concern, one of which was a review of the statistical analysis. In my opinion the statistical analysis is adequate. 

==============================

Please submit your revised manuscript by May 04 2023 11:59PM as soon as you are able to access the services of a language editor. If you will need more time than this to complete your revisions, please reply to this message or contact the journal office at plosone@plos.org. When you're ready to submit your revision, log on to https://www.editorialmanager.com/pone/ and select the 'Submissions Needing Revision' folder to locate your manuscript file.

Please include the following items when submitting your revised manuscript:

- A rebuttal letter that responds to each point raised by the academic editor and reviewer(s). You should upload this letter as a separate file labeled 'Response to Reviewers'.
- A marked-up copy of your manuscript that highlights changes made to the original version. You should upload this as a separate file labeled 'Revised Manuscript with Track Changes'.
- An unmarked version of your revised paper without tracked changes. You should upload this as a separate file labeled 'Manuscript'.

If you would like to make changes to your financial disclosure, please include your updated statement in your cover letter. Guidelines for resubmitting your figure files are available below the reviewer comments at the end of this letter.

If applicable, we recommend that you deposit your laboratory protocols in protocols.io to enhance the reproducibility of your results. Protocols.io assigns your protocol its own identifier (DOI) so that it can be cited independently in the future. For instructions see: https://journals.plos.org/plosone/s/submission-guidelines#loc-laboratory-protocols. Additionally, PLOS ONE offers an option for publishing peer-reviewed Lab Protocol articles, which describe protocols hosted on protocols.io. Read more information on sharing protocols at https://plos.org/protocols?utm_medium=editorial-email&utm_source=authorletters&utm_campaign=protocols.

We look forward to receiving your revised manuscript.

Kind regards,

Margaret Williams, Ph.D

Academic Editor

PLOS ONE

Journal Requirements:

Please review your reference list to ensure that it is complete and correct. If you have cited papers that have been retracted, please include the rationale for doing so in the manuscript text, or remove these references and replace them with relevant current references. Any changes to the reference list should be mentioned in the rebuttal letter that accompanies your revised manuscript. If you need to cite a retracted article, indicate the article’s retracted status in the References list and also include a citation and full reference for the retraction notice.

Additional Editor Comments:

There are a few comments from 2 of the reviewers who proposed minor revision. I am of the opinion that the statistical analysis appears to have been conducted with accuracy; there is evidence of statistical support. I do not think it necessary for the authors to provide more statistical evidence or review the analysis of the data for this article. I do agree that the document requires language editing to ensure that grammar is correct (I have noted numerous grammatical errors) and punctuation. This requires review and adjustment by an editor.

[Note: HTML markup is below. Please do not edit.]

Reviewers' comments:

Reviewer's Responses to Questions

**Comments to the Author**

1. If the authors have adequately addressed your comments raised in a previous round of review and you feel that this manuscript is now acceptable for publication, you may indicate that here to bypass the “Comments to the Author” section, enter your conflict of interest statement in the “Confidential to Editor” section, and submit your "Accept" recommendation.

Reviewer #1: All comments have been addressed

Reviewer #2: (No Response)

Reviewer #4: All comments have been addressed

**********

2. Is the manuscript technically sound, and do the data support the conclusions?

The manuscript must describe a technically sound piece of scientific research with data that supports the conclusions. Experiments must have been conducted rigorously, with appropriate controls, replication, and sample sizes. The conclusions must be drawn appropriately based on the data presented. 

Reviewer #1: Yes

Reviewer #2: Yes

Reviewer #4: Yes

**********

3. Has the statistical analysis been performed appropriately and rigorously? 

Reviewer #1: Yes

Reviewer #2: Yes

Reviewer #4: Yes

**********

4. Have the authors made all data underlying the findings in their manuscript fully available?

The PLOS Data policy requires authors to make all data underlying the findings described in their manuscript fully available without restriction, with rare exception (please refer to the Data Availability Statement in the manuscript PDF file). The data should be provided as part of the manuscript or its supporting information, or deposited to a public repository. For example, in addition to summary statistics, the data points behind means, medians and variance measures should be available. If there are restrictions on publicly sharing data—e.g. participant privacy or use of data from a third party—those must be specified.

Reviewer #1: Yes

Reviewer #2: Yes

Reviewer #4: Yes

**********

5. Is the manuscript presented in an intelligible fashion and written in standard English?

PLOS ONE does not copyedit accepted manuscripts, so the language in submitted articles must be clear, correct, and unambiguous. Any typographical or grammatical errors should be corrected at revision, so please note any specific errors here.

Reviewer #1: Yes

Reviewer #2: Yes

Reviewer #4: Yes

**********

6. Review Comments to the Author

Please use the space provided to explain your answers to the questions above. You may also include additional comments for the author, including concerns about dual publication, research ethics, or publication ethics. (Please upload your review as an attachment if it exceeds 20,000 characters)

Reviewer #1: Thank you for the revised manuscript and detailed responses to my previous suggestions. I find the revised manuscript to be much more clear and more comprehensible. Because you addressed my previous suggestions, I find the manuscript ready to be published.

Reviewer #2: Thank you for the opportunity to review the revised version of this paper. The current version is a major improvement over the previous version. Thus, I have no major comment. My only comment relates to the title: authors may use a colon instead of a semi-colon. I.e., Diabetes self-management education interventions and self- management in low-resource settings: a mixed methods study

I would advise the authors that in their future publications, they should endeavor to present the responses to reviewer comments in such a way that it would be easy for reviewers to verify/check the extent to which issues raised have been addressed. Normally, you have to state the reviewer comment first, then you indicate your rebuttal beneath it and also indicate the page and section of the revised paper where changes have been made. The authors gave me extra work going through their responses to see whether they had addressed all the issues I raised. I had to use my earlier review report to do this. Because, they used only the numbers of the reviewer comments. I see this extra work put on reviewers to be unfair.

Thank you

Reviewer #4: This is a well conducted study.

It presents a topic of global concerns and identifies the gaps in African health system.

You have been able to project towards the path to quality DMSE in Africa.

Though the transferability of the study is subject to criticisms, i think this study is a good addition to existing studies.

I congratulate you for bridging the knowledge gap at the right time.

My few comments is as in the attached. Thank you.

**********

7. PLOS authors have the option to publish the peer review history of their article (what does this mean?). If published, this will include your full peer review and any attached files.

If you choose “no”, your identity will remain anonymous but your review may still be made public.

**Do you want your identity to be public for this peer review?** For information about this choice, including consent withdrawal, please see our Privacy Policy.

Reviewer #1: **Yes: **Hadi Tehrani

Reviewer #2: No

Reviewer #4: **Yes: **Adeloye Amoo Adeniji

**********

[NOTE: If reviewer comments were submitted as an attachment file, they will be attached to this email and accessible via the submission site. Please log into your account, locate the manuscript record, and check for the action link "View Attachments". If this link does not appear, there are no attachment files.]

While revising your submission, please upload your figure files to the Preflight Analysis and Conversion Engine (PACE) digital diagnostic tool, https://pacev2.apexcovantage.com/. PACE helps ensure that figures meet PLOS requirements. To use PACE, you must first register as a user. Registration is free. Then, login and navigate to the UPLOAD tab, where you will find detailed instructions on how to use the tool. If you encounter any issues or have any questions when using PACE, please email PLOS at figures@plos.org. Please note that Supporting Information files do not need this step.

Attachment Submitted filename: PONE-D-22-15096_R1.pdf Click here for additional data file.

# Author comment: Author response to Decision Letter 1

**DOI:** 10.1371/journal.pone.0286974.r004

31 Mar 2023

comment:I also agree that the manuscript requires the attention of an editor. I note that there is an attempt at language editing but there are numerous basic errors that require attention, sentence structure and punctuation

Response:Thank you for drawing our attention to this. We have edited the entire paper and we have endeavoured to correct all language use errors

Attachment Submitted filename: 30-03 Response to reviewers.docx Click here for additional data file.

# Peer review: Decision Letter 2

**DOI:** 10.1371/journal.pone.0286974.r005

**Authors:** Edward Zimbudzi

29 May 2023

Diabetes self-management education interventions and self-management in low-resource settings; a mixed methods study

PONE-D-22-15096R2

Dear Dr. Lamptey,

We’re pleased to inform you that your manuscript has been judged scientifically suitable for publication and will be formally accepted for publication once it meets all outstanding technical requirements.

Within one week, you’ll receive an e-mail detailing the required amendments. When these have been addressed, you’ll receive a formal acceptance letter and your manuscript will be scheduled for publication.

An invoice for payment will follow shortly after the formal acceptance. To ensure an efficient process, please log into Editorial Manager at http://www.editorialmanager.com/pone/, click the 'Update My Information' link at the top of the page, and double check that your user information is up-to-date. If you have any billing related questions, please contact our Author Billing department directly at authorbilling@plos.org.

If your institution or institutions have a press office, please notify them about your upcoming paper to help maximize its impact. If they’ll be preparing press materials, please inform our press team as soon as possible -- no later than 48 hours after receiving the formal acceptance. Your manuscript will remain under strict press embargo until 2 pm Eastern Time on the date of publication. For more information, please contact onepress@plos.org.

Kind regards,

Edward Zimbudzi

Academic Editor

PLOS ONE

Additional Editor Comments (optional):

Reviewers' comments:

Reviewer's Responses to Questions

**Comments to the Author**

1. If the authors have adequately addressed your comments raised in a previous round of review and you feel that this manuscript is now acceptable for publication, you may indicate that here to bypass the “Comments to the Author” section, enter your conflict of interest statement in the “Confidential to Editor” section, and submit your "Accept" recommendation.

Reviewer #1: (No Response)

Reviewer #2: All comments have been addressed

Reviewer #4: All comments have been addressed

**********

2. Is the manuscript technically sound, and do the data support the conclusions?

The manuscript must describe a technically sound piece of scientific research with data that supports the conclusions. Experiments must have been conducted rigorously, with appropriate controls, replication, and sample sizes. The conclusions must be drawn appropriately based on the data presented. 

Reviewer #1: (No Response)

Reviewer #2: Yes

Reviewer #4: Yes

**********

3. Has the statistical analysis been performed appropriately and rigorously? 

Reviewer #1: (No Response)

Reviewer #2: (No Response)

Reviewer #4: Yes

**********

4. Have the authors made all data underlying the findings in their manuscript fully available?

The PLOS Data policy requires authors to make all data underlying the findings described in their manuscript fully available without restriction, with rare exception (please refer to the Data Availability Statement in the manuscript PDF file). The data should be provided as part of the manuscript or its supporting information, or deposited to a public repository. For example, in addition to summary statistics, the data points behind means, medians and variance measures should be available. If there are restrictions on publicly sharing data—e.g. participant privacy or use of data from a third party—those must be specified.

Reviewer #1: (No Response)

Reviewer #2: (No Response)

Reviewer #4: Yes

**********

5. Is the manuscript presented in an intelligible fashion and written in standard English?

PLOS ONE does not copyedit accepted manuscripts, so the language in submitted articles must be clear, correct, and unambiguous. Any typographical or grammatical errors should be corrected at revision, so please note any specific errors here.

Reviewer #1: (No Response)

Reviewer #2: (No Response)

Reviewer #4: Yes

**********

6. Review Comments to the Author

Please use the space provided to explain your answers to the questions above. You may also include additional comments for the author, including concerns about dual publication, research ethics, or publication ethics. (Please upload your review as an attachment if it exceeds 20,000 characters)

Reviewer #1: Thank you for the revised manuscript and detailed responses to my previous suggestions. I find the revised manuscript to be much more clear and more comprehensible. Because you addressed my previous suggestions, I find the manuscript ready to be published.

Reviewer #2: (No Response)

Reviewer #4: I have read through the reviewed fashion, and I can submit that it is scientifically sound and stands the pedigree of its original social value.

I think the paper now worth its value as a scientific writing.

All papers a succumbed to critical analysis in the world of science, but I am of the opinion that this study will receive minimal criticisms.

I think all the mistakes and errors that may question the integrity of the paper has been corrected.

**********

7. PLOS authors have the option to publish the peer review history of their article (what does this mean?). If published, this will include your full peer review and any attached files.

If you choose “no”, your identity will remain anonymous but your review may still be made public.

**Do you want your identity to be public for this peer review?** For information about this choice, including consent withdrawal, please see our Privacy Policy.

Reviewer #1: No

Reviewer #2: No

Reviewer #4: **Yes: **Adeloye Amoo Adeniji (MBBS; MMed; FCFP; FACRRM)

**********

# Editor report: Acceptance letter

**DOI:** 10.1371/journal.pone.0286974.r006

**Authors:** Edward Zimbudzi

7 Jul 2023

PONE-D-22-15096R2 

Diabetes self-management education interventions and self-management in low-resource settings; a mixed methods study 

Dear Dr. Lamptey:

I'm pleased to inform you that your manuscript has been deemed suitable for publication in PLOS ONE. Congratulations! Your manuscript is now with our production department. 

If your institution or institutions have a press office, please let them know about your upcoming paper now to help maximize its impact. If they'll be preparing press materials, please inform our press team within the next 48 hours. Your manuscript will remain under strict press embargo until 2 pm Eastern Time on the date of publication. For more information please contact onepress@plos.org.

If we can help with anything else, please email us at plosone@plos.org. 

Thank you for submitting your work to PLOS ONE and supporting open access. 

Kind regards, 

PLOS ONE Editorial Office Staff

on behalf of

Dr. Edward Zimbudzi 

Academic Editor

PLOS ONE
